# Mechanistic insights into reductive deamination with hydrosilanes catalyzed by B(C_6_F_5_)_3_: A DFT study

**DOI:** 10.3389/fchem.2022.1025135

**Published:** 2022-11-16

**Authors:** Miaomiao Zhou, Ting Wang, Gui-Juan Cheng

**Affiliations:** ^1^ Warshel Institute for Computational Biology, School of Medicine, The Chinese University of Hong Kong (Shenzhen), Shenzhen, China; ^2^ Department of Chemistry, City University of Hong Kong, KowloonTong, Hong Kong SAR, China; ^3^ School of Life and Health Sciences, School of Medicine, The Chinese University of Hong Kong (Shenzhen), Shenzhen, China; ^4^ Shenzhen Key Laboratory of Steroid Drug Development, School of Medicine, The Chinese University of Hong Kong (Shenzhen), Shenzhen, China

**Keywords:** B(C_6_F_5_)_3_, reductive deamination, reaction mechanism, substituent effect, DFT

## Abstract

Selective defunctionalization of synthetic intermediates is a valuable approach in organic synthesis. Here, we present a theoretical study on the recently developed B(C_6_F_5_)_3_/hydrosilane-mediated reductive deamination reaction of primary amines. Our computational results provide important insights into the reaction mechanism, including the active intermediate, the competing reactions of the active intermediate, the role of excess hydrosilane, and the origin of chemoselectivity. Moreover, the study on the substituent effect of hydrosilane indicated a potential way to improve the efficiency of the reductive deamination reaction.

## Introduction

In the search for renewable alternatives, biomass feedstock is usually a promising sustainable carbon source to produce fuels, chemicals, and materials. In general, biomass-derived feedstocks, such as sugars, alcohol, phenol, and amines, are over-functionalized. Therefore, defunctionalization has become an important way to produce useful downstream chemicals, which has attracted wide attention. (Huber et al., 2006; Corma et al., 2007; Zhou et al., 2011; Zhang et al., 2017). Over last 2 decades, the deoxygenation of alcohols or derivatives has been well developed to access simple hydrocarbons. (Adlington et al., 1976; Doyle et al., 1976; Orfanopoulos and Smonou, 1988; Yasuda et al., 2001; Nimmagadda and McRae, 2006; McLaughlin et al., 2013; Dai and Li, 2016). Conversely, although amines are also one of the most common feedstock chemicals accessible by biomass conversion, the deaminative transformation of amines is poorly developed, which highlights great challenges, particularly in the development of deaminative strategies for alkyl amines and primary amines to construct C–X (X = C, O, S, B, P, H, etc.) bonds.

**SCHEME 1:**
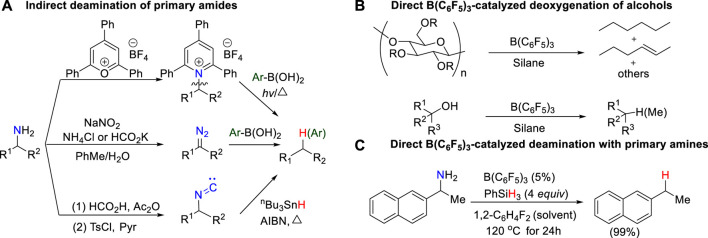
**(A)** Indirect deamination of primary amine; **(B)** Direct B(C_6_F_5_)_3_-catalyzed deoxygenation of primary alcohols; **(C)** Direct B(C_6_F_5_)_3_-catalyzed deamination of primary amines.

Deaminases, such as L-amino acid deaminases (LAAD), are essential biocatalyst in living cells. In the human body, deamination, as a common metabolic process, usually takes place to break down amino acids for the generation of their corresponding α-keto acids and ammonia by deaminases, involving in nucleotide sequence, immunity and cancer. (Massad et al., 1995; Petersen-Mahrt et al., 2009; Vesely et al., 2012; Molla et al., 2017; Nshimiyimana et al., 2019). In contrast, deamination in laboratory is very rare and difficult. Although the challenges are daunting, systematic efforts toward deamination of primary amines have recently begun to emerge, and some progress has been made. Earlier work on C–NH_2_ bond activation usually requires to pre-activate primary amines into reactive intermediates, such as active pyridinium salts (Katritzky salts) (Hu et al., 2018; Ni et al., 2019; Wang et al., 2019; Correia et al., 2020; Pang et al., 2020), electron-rich diazos (Mitsuhashi et al., 2000; Geoffroy et al., 2001; Barluenga et al., 2009; Peng et al., 2009; Wu et al., 2014), and isonitrile compounds (Barton et al., 1980; Barton et al., 1992), for further deamination, which results in a more complicated and expensive reaction process (Scheme 1A). Based on the principles of green chemistry and sustainable development, the direct activation of C–N bond of primary amines without pre-activation affords a very attractive approach to obtain valuable functionalized building blocks even though it is more challenging.

**FIGURE 1:**
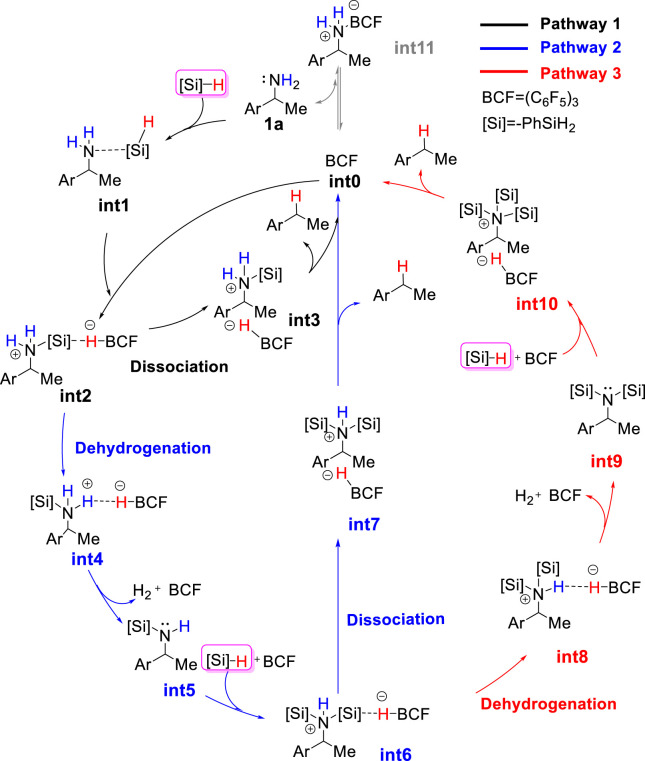
The proposed catalytic cycle for B(C_6_F_5_)_3_-catalyzed reductive deamination with hydrosilanes.

The combination of B(C_6_F_5_)_3_ and hydrosilanes was recently discovered for selective deoxygenations of 1,2-diols and polyols by the Gagné (Adduci et al., 2014; Adduci et al., 2015; Bender et al., 2016a; Bender et al., 2016b; Seo and Gagné, 2018; Seo et al., 2019), Morandi (Drosos and Morandi, 2015; Nikolaos Drosos and Morandi, 2015; Drosos et al., 2016), Yamamoto (Gevorgyan et al., 1999; Gevorgyan et al., 2000; Gevorgyan et al., 2001), and Oestreich group (Chatterjee et al., 2017; Fang and Oestreich, 2020a; Richter and Oestreich, 2021) (Scheme 1B). However, the reaction of B(C_6_F_5_)_3_ and hydrosilanes with amines generally does not give deamination product; instead, it affords N-silylation product which is formed *via* B(C_6_F_5_)_3_-catalyzed dehydrogenative coupling of amines and hydrosilanes. (Hermeke et al., 2013; Greb et al., 2014; Zhang et al., 2018; Zhou et al., 2020). Recently, the Oestreich group reported the first metal-free, B(C_6_F_5_)_3_/hydrosilane-mediated deamination reaction of primary amines, which was a breakthrough for the direct C–NH_2_ bond defunctionalization (Scheme 1C). (Fang and Oestreich, 2020b) Their study showed that the amount of silane reagent is essential to the reaction: 4 equivalents of PhSiH_3_ were required to obtain high yields, and less PhSiH_3_ would result in poor yields. Additionally, they found the substitution degree of benzylamines significantly affects the reactivity. But the role of excess hydrosilane and substituent effect on reactivity remain elusive. In addition, the stoichiometric experiments indicated the existence of silylammonium borohydride which was proposed to undergo C–N cleavage to afford deamination product. However, it is unclear which species among mono-, di-, or tri-silylammonium borohydrides (i.e., **int2**, **int6** or **int10 s**hown in Figure 1) is the active intermediate. Moreover, it remains unknown how does deamination compete over the dehydrogenative coupling to successfully afford desired product. A deeper understanding of the reaction mechanism may provide useful information for the optimization and development of deamination reactions.

Our group is particularly interested in the diverse catalytic capabilities of B(C_6_F_5_)_3_ system. In previous computational study on B(C_6_F_5_)_3_-catalyzed deoxygenation of polyols with silanes, we rationalized the special role of the cyclic siloxane intermediate in promoting reactivity and selectivity, as well as the different product distributions obtained with different silanes. (Drosos et al., 2017; Cheng et al., 2018).

Herein, we disclose the reaction mechanism of B(C_6_F_5_)_3_-catalyzed deamination by theoretical studies, thereby expanding the understanding of B(C_6_F_5_)_3_ catalytic system. The present work provides a detailed mechanistic picture for B(C_6_F_5_)_3_-catalyzed deamination reaction, unveils the role of excess hydrosilane, and explains the substituent effect on reactivity.

## Computational details

All the calculations were performed with Gaussian 16 (Frisch et al., 2016) package. All molecular geometries were optimized with B3LYP-D3/def2SVP method in gas phase. (Lee et al., 1988; Schafer et al., 1992; Becke, 1993; Schafer et al., 1994; Grimme et al., 2011). Optimized geometries were verified by frequency computations as minima (zero imaginary frequencies) or transition state (a single imaginary frequency) at the same level of theory. The transition states (TSs) were also confirmed by viewing normal mode vibrational vector and by intrinsic reaction coordinate (IRC) calculation. (Gonzalez and Gonzalez, 1990). All the single point energy calculations in solution phase were carried out by SMD(Marenich et al., 2009) model with 1,2-diflurobenzene as the solvent and B3LYP-D3 method with the def2-TZVP basis set. All of Gibbs energies were corrected at 393.15K. Both relative Gibbs energies and electronic energies were reported in kcal/mol. The 3D structures were generated by CYLview. (Legault, 2009). The conformational space of the system has been extensively explored manually by rotating the torsional angles of the molecule and automatically by using Crest program. (Grimme, 2019).

## Results and discussion

### Reaction mechanism of B(C_6_F_5_)_3_-catalyzed reductive deamination with PhSiH_3_ and 1a

**FIGURE 2:**
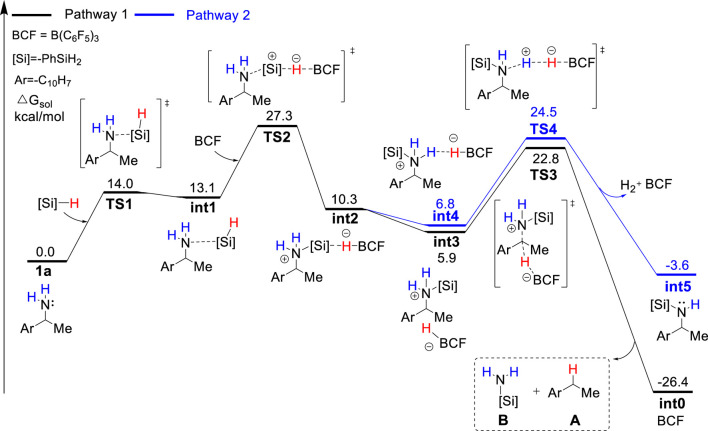
Gibbs energy profile for the B(C_6_F_5_)_3_-catalyzed reductive deamination with PhSiH_3_ and **1a** *via* pathway 1 and 2.

As discussed in the introduction, silylammonium borohydride was detected in the stoichiometric experiment and proposed to undergo C–N cleavage to form deamination product. Because it is unknown which one among mono-, bi-, and tri-silylammonium borohydrides (i.e., **int2**, **int6** or **int10** shown in Figure 1) is the active intermediate that leads to deamination product, we calculated three possible pathways that involve different silylammonium borohydrides. As shown in Figure 2, the reaction is initiated by the association of substrate **1a** with one PhSiH_3_. This step needs to overcome a Gibbs energy barrier of 14.0 kcal/mol (**TS1**), generating a thermodynamically unstable amine-silane complex **int1**. Then, the Lewis acid B(C_6_F_5_)_3_ abstracts a hydride from **int1**, which is accompanied by the N–Si bond formation to afford the monosilylammonium borohydride species (**int2**). The activation energy barrier for the silylation process is 27.3 kcal/mol (**TS2**). The monosilylammonium borohydride intermediate then undergoes C–N bond dissociation (pathway 1), where borohydride (C_6_F_5_)_3_BH^−^ acts as a nucleophile to attack the benzylic carbon of silylammonium moiety *via* an *S*_*N*_ *2-type* transition state (**TS3**) to afford the desired deamination product **A** and monosilazane **B**. The rate-determining step of pathway one is the silylation step (**TS2**) and overall reaction barrier is 27.3 kcal/mol.

**FIGURE 3:**
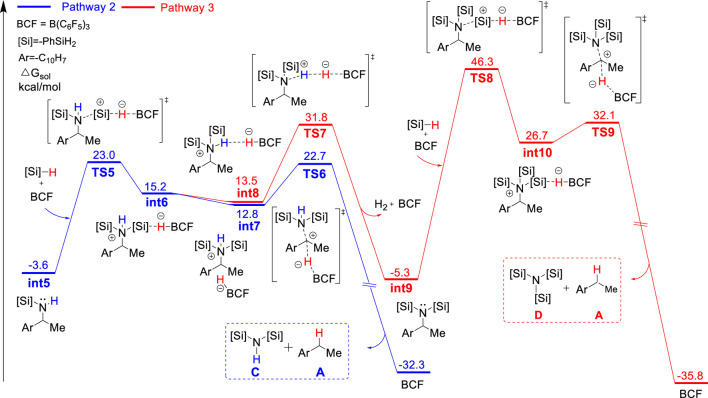
Gibbs energy profile for the B(C_6_F_5_)_3_-catalyzed reductive deamination with PhSiH_3_ and **1a** *via* pathway 2 and 3.

Alternatively, the monosilylammonium borohydride intermediate may occur dehydrogenative reaction (pathway 2, blue color), in which (C_6_F_5_)_3_BH^−^ accepts a proton from the amine group (**TS4**), which releases a H_2_ molecule, B(C_6_F_5_)_3_ and mono-silylated amine **int5**. The Gibbs energy barrier of **TS4** is 1.7 kcal/mol higher than that of **TS3**, indicating that the monosilylammonium borohydride intermediate prefers C–N dissociation than dehydrogenation. Follow the dehydrogenative step, **int5** further reacts with another PhSiH_3_ molecule and B(C_6_F_5_)_3_ catalyst to give the disilylammonium borohydride intermediate, **int6** (Figure 3). Like the monosilylammonium borohydride, the disilylammonium borohydride intermediate can undergo an *S*_*N*_ *2-type* C–N cleavage (**TS6**, ΔG^‡^ = 26.3 kcal/mol) to yield the deamination species **A** and disilazane **C**, completing pathway 2. Pathways one and two bifurcate at the monosilylammonium borohydride intermediate and pathway two is less favorable since the monosilylammonium borohydride intermediate favors C–N dissociation.

**FIGURE 4:**
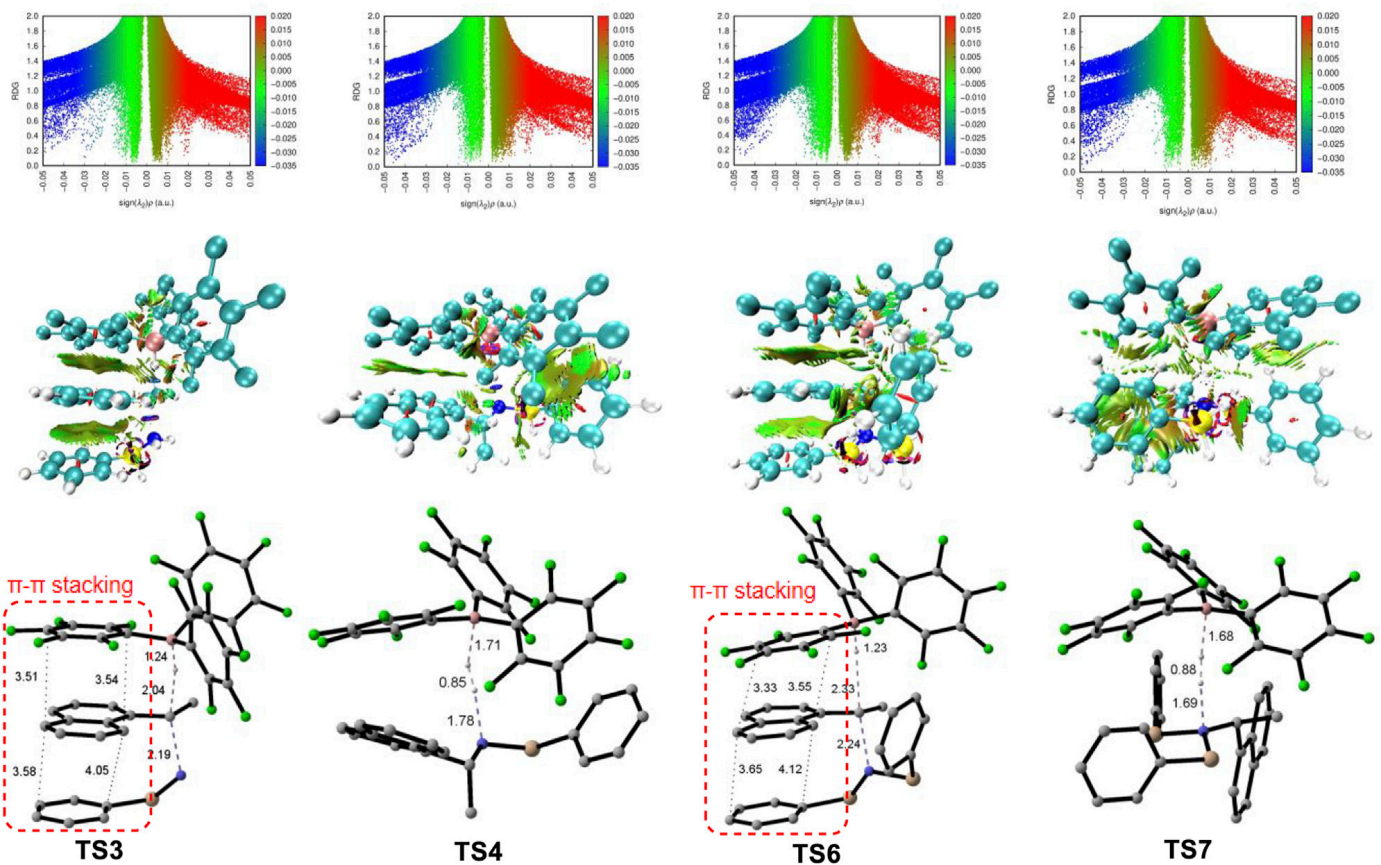
Top: Overlapping troughs in s(*ρ*) plots can be distinguished when sign (*λ*_*2*_)*ρ* is used as the ordinate. Favorable interactions appear on the left, unfavorable on the right, and van der Waals near zero; Medium: NCI surfaces of **TS3**, **TS4**, **TS6 and TS7** correspond to s = 0.5 au and a colour scale of −0.05 < *ρ* < 0.05 au for SCF densities; Bottom: structures of **TS3**, **TS4**, **TS6 and TS7** (Non-reacting hydrogen atoms are omitted for clarity).

In the alternative pathway 3 (in red color), the dehydrogenation of disilylammonium borohydride intermediate *via* **TS7** (ΔG^‡^ = 35.4 kcal/mol) releases one H_2_ molecule and a disilyated amine (**int9**). **int9** then reacts with another molecule of PhSiH_3_ and B(C_6_F_5_)_3_ for further silylation. The silylation step proceeds *via* a very high energy barrier transition state, **TS8**, (ΔG^‡^ = 51.6 kcal/mol) and yields a highly unstable trisilylammonium borohydride species **int10**. Finally, the **C**–**N** dissociation of the trisilylammonium borohydride *via* **TS9** releases product **A** and trisilazane **D**. In summary, the computational results suggest the direct C–N dissociation of the monosilylammonium borohydride to generate monosilazane and deamination product (pathway 1) is the most favorable pathway for this reductive deamination reaction. The first silylation step *via* **TS2** is the rate-limiting step and the overall energy barrier is 27.5 kcal/mol. Moreover, the TSs of dehydrogenation of both mono- and di-silylammonium borohydride are higher in energy than their corresponding TSs of C–N dissociation energy (**TS3** vs*.* **TS4** and **TS6** vs*.* **TS7**), suggesting the deamination is more favorable than dehydrogenation reaction which is consistent with experimental results. The lower activation barrier of deamination can be attributed to the weaker bond strength of C–N bond compared with the N–H bond. The calculated bond dissociation energy of C–N bond is lower than that of N–H bond by 8.5 and 13.2 kcal/mol for mono- and di-silylammonium borohydride, respectively (Supplementary Table S1). Thus, the C–N bond is easier to be cleavage than the N–H bond. In addition, well-ordered π-π stacking interactions between the naphthyl ring of substrate, the phenyl ring of silane and the pentafluorophenyl group of the catalyst were identified in **TS3** and **TS6** (Figure 4), which help to stabilize the deamination TSs. The NCI analysis (Humphrey et al., 1996; Lu and Chen, 2012) supports the existence of attractive π-π stacking interactions in **TS3** and **TS6**.

**FIGURE 5:**
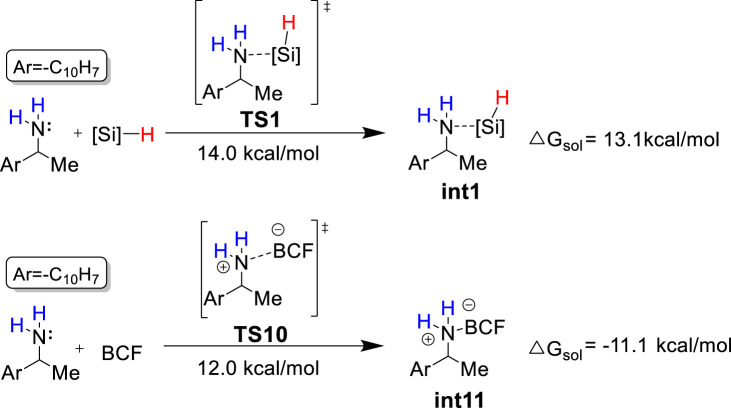
Competition between the formation of **int1** and **int11**. BCF = B(C_6_F_5_)_3_; [Si] = −PhSiH_2_.

Based on the theoretical calculation, the most favorable pathway (i.e., pathway 1) only consumes one equivalent of PhSiH_3_ to afford the deamination product **A**, which contradicts with the experimental observation that four equivalents of PhSiH_3_ are required to achieve good yields. Because both PhSiH_3_ and B(C_6_F_5_)_3_ are Lewis acids, we envision that PhSiH_3_ and B(C_6_F_5_)_3_ may compete to bind with the amine substrate to form amine-silane ([N-Si]) and amine-boron ([N-B]) Lewis adducts, respectively. As shown in Figure 5, the binding of **1a** with B(C_6_F_5_)_3_ has a Gibbs energy barrier lower by 2.0 kcal/mol than with PhSiH_3_ and leads to a very stable [N-B] Lewis adduct **int11** (Δ*G* ^o^ = −11.1 kcal/mol). Thus, computational results suggest that the formation of [N–B] Lewis adduct (**int11**) is kinetically and thermodynamically more favorable than [N–Si] Lewis adduct (**int1**). The favorable formation of [N–B] Lewis adduct can be attributed to the stronger Lewis acidity of B(C_6_F_5_)_3_. Frontier molecular orbital analysis supports that B(C_6_F_5_)_3_ is a much stronger Lewis acid than PhSiH_3_ and thus easier to accept a lone pair electron from **1a** (Supplementary Figure S2).

The computational results demonstrate that when B(C_6_F_5_)_3_ and PhSiH_3_ were added with a ratio of 1:1, the amine substrate prefers to bind with B(C_6_F_5_)_3_ and form a very stable Lewis adduct (**int11**) which is the resting state of catalyst and substrate. Taking the resting state **int11** as the start point for the deamination reaction, the overall reaction barrier is as high as 38.6 kcal/mol (**int11** to **TS2**), which is difficult to overcome. This result is in line with the experimental observation that **int11** rather than the deamination product was obtained as main product in the stoichiometric experiments with stepwise addition of B(C_6_F_5_)_3_ and PhSiH_3_.

In the catalytic reaction with 20% mol of B(C_6_F_5_)_3_, the use of four equivalents of PhSiH_3_ afforded deamination product as main product. We speculate this is related to the concentration effect which influences the competition between the formation of **int1** and **int11**. Based on the reaction rate equation, the reaction rate (*r*_1_ and *r*_2_) for the formation of **int1** and **int11** can be calculated by (Eqs 1, 2), respectively. Thus, their ratio (*r*_1_/*r*_2_) is determined by Eq. 3 which is affected by the ratio of rate constants (*k*_1_/*k*_2_) and the concentration ([PhSiH_3_]/[BCF]). *k*_1_/*k*_2_ is calculated based on the Erying equation (Eq. 4)

$$r_{1}=k_{1}\left[1a\right]\left[PhSiH_{3}\right]$$ (1)

$$r_{2}=k_{2}\left[1a\right]\left[BCF\right]$$ (2)

$$\frac{r_{1}}{r_{2}}=\frac{k_{1}}{k_{2}}\;\frac{\left[PhSiH_{3}\right]}{\left[BCF\right]}$$ (3)

$$\frac{k_{1}}{k_{2}}={\frac{\frac{k_{B}T}{h}e^{-\frac{{∆G}_{1}^{\neq }}{RT}}}{\frac{k_{B}T}{h}e^{-\frac{∆G_{2}^{\neq }}{RT}}}=e}^{-\frac{∆G_{1}^{\neq }-∆G_{2}^{\neq }}{RT}}$$ (4)

As the activation energy difference between **TS1** and **TS10** is 2.0 kcal/mol (Figure 5), *k*_1_/*k*_2_ is calculated to be 0.077 at 120°C. Therefore, *r*_1_/*r*_2_ for the reaction with equivalent amount of PhSiH_3_ and B(C_6_F_5_)_3_ is as small as 0.077, suggesting the formation of **int11** is dominant. However, under the catalytic reaction condition with 20% mol of B(C_6_F_5_)_3_ and 4 equivalents of PhSiH_3_, the concentration ratio [PhSiH_3_]/[ BCF] increases to 20. As a result, *r*_1_/*r*_2_ is increased by 20 folds compared to that of the stoichiometric reaction with equivalent of PhSiH_3_ and B(C_6_F_5_)_3_, and thus the formation of **int1** is 1.54 times faster than **int11**. This result is in good agreement with the experimental observation that increasing the equivalent of PhSiH_3_ gradually increases the yields of silylammonium borohydride intermediate and deamination product in the stoichiometric reaction with 1 equivalent of B(C_6_F_5_)_3_. Therefore, the excess PhSiH_3_ plays a crucial role to maintain a high [PhSiH_3_]/[BCF] ratio so that PhSiH_3_ can compete with B(C_6_F_5_)_3_ for binding with the amine substrate, avoiding the deactivation of catalyst and substrate.

### Reactivity of amines with different α-substitutions

**SCHEME 2:**
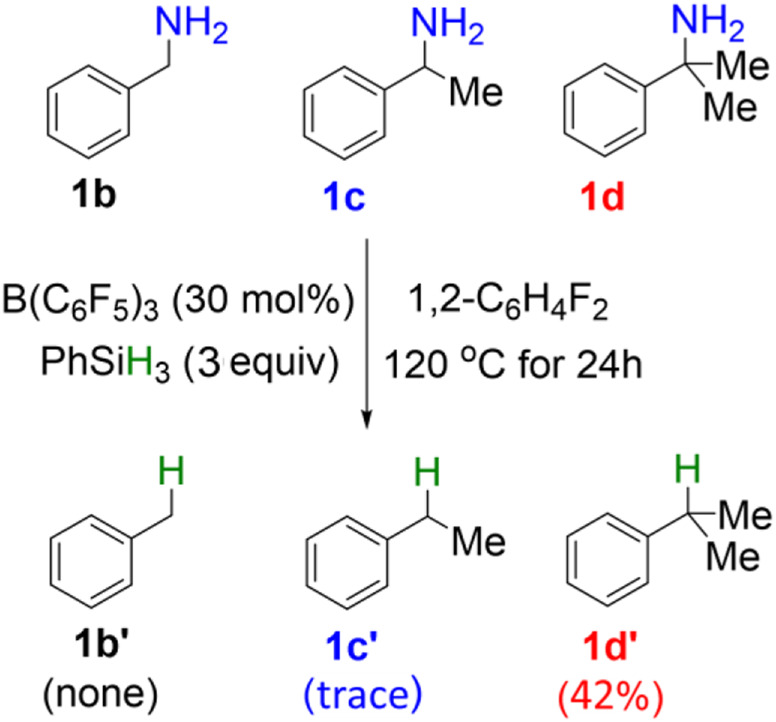
Reactivity study on B(C_6_F_5_)_3_-catalyzed reductive deamination with equimolar mixture of benzylamines **(1b**, **1c**, **1d)** and PhSiH_3_.

**FIGURE 6:**
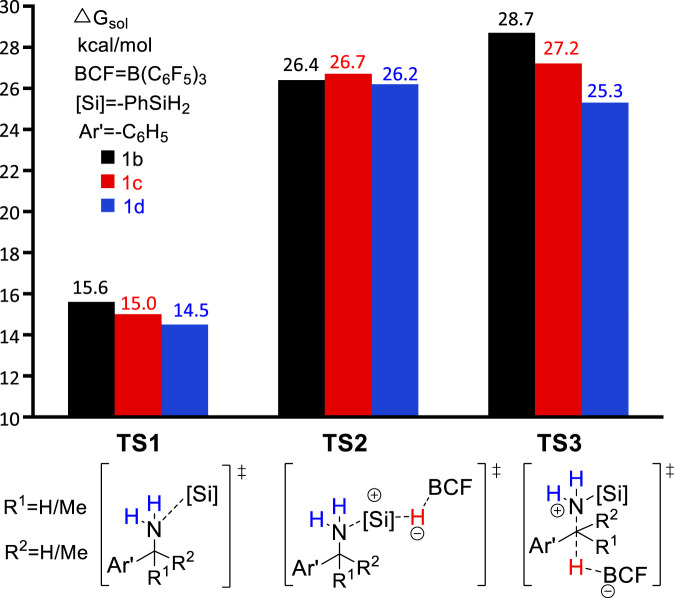
Gibbs energy of transition states **TS1, TS2,** and **TS3** for different substrates.

In the original experimental work, control experiments were performed to assess the relative reactivity of a variety of benzylamines. As shown in Scheme 2, the competition reaction with one equivalent of an equimolar benzylamines mixture (**1b**, **1c**, **1d**) demonstrates that the relative reactivity of benzylamines follows the order: **1b** < **1c** < **1d**. To understand the relative reactivity of benzyl amines with different degrees of substitution at the α-carbon atom, we calculated the reaction pathway one for all substrates. Pathway one involves three main steps, i.e., the amine-silane binding, amine silylation, and deamination (Figure 6). It is worth noting that, for substrate **1d**, the silylation step (**TS2d**, Δ*G* ^‡^ = 26.2 kcal/mol) remains as the rate-limiting step, like the reaction with **1a**. However, for substrate **1b** and **1c**, the C–N bond cleavage of monosilylammonium borohydride (i.e., deamination) *via* **TS3b** (Δ*G* ^‡^ = 28.7 kcal/mol) and **TS3c (**Δ*G* ^‡^ = 27.2 kcal/mol**)** becomes the rate-determining step. Thus, the overall reaction energy barriers for the deamination of **1b/1c/1d** are calculated to be 28.7, 27.2 and 26.2 kcal/mol, respectively, which is consistent with the experimental observed reactivity order. Figure 6 clearly shows that the C–N bond cleavage (**TS3**) of **1d** is more favorable than **1b** and **1c**, which is because the reacting benzylic carbon in **TS3d** is stabilized by more methyl substituents, leading to stronger stabilization effect on the transition state.

### Substituent effect of hydrosilanes

**FIGURE 7:**
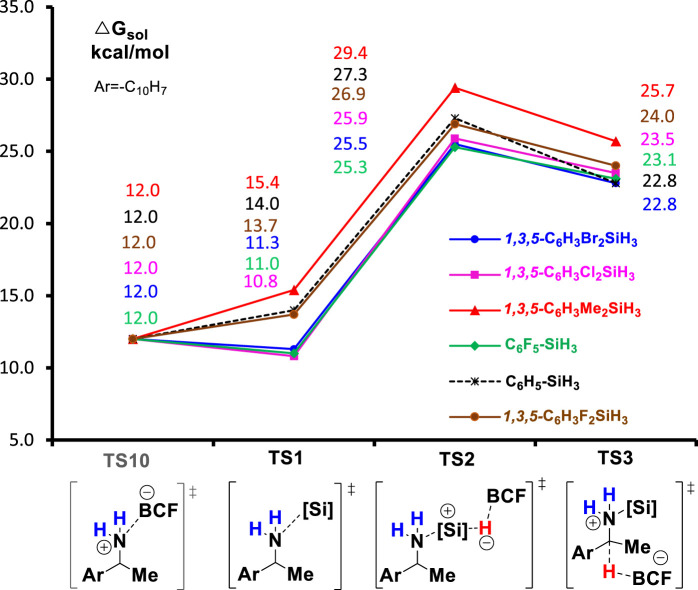
The B(C_6_F_5_)_3_-catalyzed reductive deamination with different hydrosilanes.

In the end, we turn our attention to the reactivity of hydrosilanes, another important reactant for the deamination reaction. To study the substituent effect of PhSiH_3_, we calculated the reaction of a series of PhSiH_3_ derivatives that carry electron-withdrawing groups (EWGs) or electron-donating groups (EDGs) at the phenyl ring. Figure 7 summarizes the Gibbs energies of all TSs of deamination reaction with different silanes (PhSiH_3_, C_6_F_5_SiH_3_, *1,3,5-*C_6_H_3_F_2_SiH_3_, *1,3,5-*C_6_H_3_Cl_2_SiH_3_, *1,3,5-*C_6_H_3_Br_2_SiH_3_, and *1,3,5-*C_6_H_3_Me_2_SiH_3_). In all reactions, the silylation step (**TS2**) is the rate-determining step. Compared to unsubstituted PhSiH_3_, hydrosilanes with EWGs, such as F, Cl, and Br substituents, lower the barrier of **TS2** by 0.4–2.0 kcal/mol. Moreover, the formation of amine-silane complex (**TS1**) becomes more favorable than the generation of amine-boron Lewis product as **TS1** for the EWG-substituted hydrosilanes are lower in energy than **TS10** by 0.7–1.2 kcal/mol. This indicates that the reaction with these hydrosilanes may not need excess amount of silane reagent. The increased reactivity and binding affinity of hydrosilanes caused by the EWGs may because the EWGs increases the acidity of hydrosilanes which makes them more reactive toward amine substrates. On the contrary, EDGs will decrease the acidity of hydrosilane and thus lower the reactivity. Indeed, the potential energy surface of *1,3,5-*C_6_H_3_Me_2_SiH_3_ lies above the energy surface of PhSiH_3_.

## Conclusion

In the present study, we perform DFT calculations on the reaction of B(C_6_F_5_)_3_-catalyzed reductive deamination of benzylic amines with hydrosilanes. Three possible reaction pathways (shown in Figure 1) involving mono-, bi- or tri-silylammonium borohydride as active intermediate were explored. The computational results reveal that the pathway one which includes the deamination of monosilylammonium borohydride is most favorable. Pathway one consists of three steps: first, amine and silane associate to form an amine-silane Lewis adduct; then, the amine-silane complex is catalyzed by B(C_6_F_5_)_3_ to undergo silylation reaction, affording monosilylammonium borohydride intermediate; finally, the C–N dissociation of monosilylammonium borohydride intermediate generates the desired deamination product. The second step (silylation process) is the rate-determining step. The monosilylammonium borohydride prefers to undergo *S*_*N*_ *2* C–N bond dissociation rather than the dehydrogenation, probably because the C–N is weaker than the N–H bond and the π-π stacking interaction stabilizes the transition state for C–N bond dissociation.

Our computational results suggest that B(C_6_F_5_)_3_ acts as stronger Lewis acid than hydrosilane to bind with amine substrate, which will deactivate the catalyst and substrate. The excess silanes used in the experiment play an essential role to maintain a high concentration of silane which enables PhSiH_3_ to compete with B(C_6_F_5_)_3_ for binding with amine substrate, avoiding the deactivation of catalyst and substrate. Furthermore, the calculated relative reactivity of benzylamines with different degrees of substitution agrees well with the experimental observed reactivity order. In addition, our DFT studies on the substituent effect of silanes indicate that the introduction of electron-withdrawing groups on the phenyl ring of PhSiH_3_ could lower the reaction energy barrier of the reductive deamination reaction, which may improve the reaction efficiency. Overall, this work promotes the understanding of mechanism of deamination reaction and lays the theoretical foundation for the development of new deamination methodology.

## Data Availability

The original contributions presented in the study are included in the article/Supplementary Material, further inquiries can be directed to the corresponding author.

## Supplementary material

Supplementary Figure S1 Gibbs energy profile for the B(C6F5)3-catalyzed reductive deamination with PhSiH3 and 1b/1c/1d by pathway 2.

Supplementary Figure S2 Frontier molecular orbital analysis of 1a, B(C6F5) and PhSiH3 (top) and the NCI surfaces of int1 and int11 (bottom).

Supplementary Figure S3 The formation of int12 and int13.

Supplementary Figure S4 Gibbs energy profile for the structural rearrangement between int2 and int4.

Supplementary Figure S5 Gibbs energy profile for the structural rearrangement between int6 and int8.

Supplementary Table S1 Corrections to zero point energies, enthalpies, free energies and electronic potential energies (in Hartree) and imaginary frequencies (IF) (cm-1) of optimized structures which were calculated at B3LYP-D3/def2-SVP//B3LYP-D3/def2-TZVP level in solvent (1,2- diflurobenzene) at 298.15 K and 393.15K and 1 atm.
