# Supplementary material for: Mechanistic insights into reductive deamination with hydrosilanes catalyzed by B(C6F5)3: A DFT study
Source: Front Chem. 2022 Nov 16;10:1025135. doi: 10.3389/fchem.2022.1025135 (PMC9709212; doi:10.3389/fchem.2022.1025135)
Supplement: Supplementary file 1 [file DataSheet1.docx]

*Supporting Information*

**Mechanistic Insights into Reductive Deamination with Hydrosilanes Catalyzed by B(C_6_F_5_)_3_: A DFT Study**

Miaomiao Zhou, ^a,^ ^c, +^ Ting Wang,^a,^ ^+^ and Gui-Juan Cheng^a, b,^ *

^a^ Warshel Institute for Computational Biology, School of Life and Health Sciences, The Chinese University of Hong Kong (Shenzhen), Shenzhen 518172, China

Email: chengguijuan@cuhk.edu.cn

^b^ Shenzhen Key Laboratory of Steroid Drug Development, School of Life and Health Sciences, The Chinese University of Hong Kong (Shenzhen), Shenzhen 518172, China.

^c^ Department of Chemistry, City University of Hong Kong, Tat Chee Avenue, KowloonTong, Hong Kong, China.

^+^  These authors contributed equally to this work

1. **Computational Results**

**Figure S1.** Gibbs energy profile for the B(C_6_F_5_)_3_-catalyzed reductive deamination with PhSiH_3_ and **1b**/**1c**/**1d** by pathway 2.

| **Substrate** | **Substrate group** | **TS10** | **Int11** | **TS1** | **Int1** | **TS2** | **Int2** | **Int4** | **TS4** | **Int5** | **TS5** | **Int6** | **Int7** | **TS6** |
| --- | --- | --- | --- | --- | --- | --- | --- | --- | --- | --- | --- | --- | --- | --- |
| **1b** | R_1_=H R_2_=H | 13.0 | -10.7 | 15.6 | 12.0 | 26.4 | 11.6 | 5.2 | 25.9 | -2.9 | 22.4 | 14.7 | 12.9 | 31.1 |
| **1c** | R_1_=CH_3_ R_2_=H | 14.5 | -10.7 | 15.0 | 13.1 | 26.7 | 10.5 | 6.0 | 24.4 | -4.6 | 23.7 | 16.4 | 13.4 | 27.3 |
| **1d** | R_1_=CH_3_ R_2_=CH_3_ | 13.2 | -5.2 | 14.5 | 12.7 | 26.2 | 13.6 | 8.1 | 28.4 | -1.9 | 24.9 | 20.6 | 16.6 | 26.4 |

**Figure S2.** Frontier molecular orbital analysis of **1a**, B(C_6_F_5_) and PhSiH_3_ (top) and the NCI surfaces of **int1** and **int11** (bottom).

 **Figure S3.** The formation of **int12** and **int13**.

**Figure S4.** Gibbs energy profile for the structural rearrangement between **int2** and **int4**.

**Figure S5.** Gibbs energy profile for the structural rearrangement between **int6** and **int8**.

In pathways 1 and 2, the monosilylammonium borohydride specie (**int2**) can be converted to **int4** via structural rearrangement, which need overcome a very small and negligible energy barrier (shown in Figure S4). Similarly, it could also be negligible for the rearrangement from **int6** to **int8** due to the very small energy barrier (shown in Figure S5). Based on the result, we infer the energy barrier for rearrangement between **int2** and **int3** as well as that between **int6** and **int7** should also be negligible

**Table S1. Corrections to zero point energies, enthalpies, free energies and electronic potential energies (in Hartree) and imaginary frequencies (IF) (cm-1) of optimized structures which were calculated at B3LYP-D3/def2-SVP//B3LYP-D3/def2-TZVP level in solvent (1,2-diflurobenzene) at 298.15 K and 393.15K and 1 atm**.

| **Geometry** | | **cZPE_298,gas_** | | **cH_298,gas_** | | **cG_298,gas_** | **cG_393,gas_** | **E_0,sol_** | **IF** | |
| --- | --- | --- | --- | --- | --- | --- | --- | --- | --- | --- |
| **1a** | | 0.221640 | | 0.233270 | | 0.185714 | 0.169450 | -520.091843049 | - | |
| **PhSiH_3_** | | 0.115342 | | 0.123295 | | 0.083030 | 0.069107 | -523.071216833 | - | |
| **H_2_** | | 0.009971 | | 0.013275 | | -0.001561 | -0.006440 | -1.17993204965 | - | |
| **A** | | 0.204136 | | 0.214663 | | 0.169377 | 0.153945 | -464.714277384 | - | |
| **B** | | 0.133923 | | 0.143227 | | 0.100607 | 0.086217 | -578.492534549 | - | |
| **C** | | 0.233181 | | 0.249241 | | 0.186770 | 0.165429 | -1100.396816600 | - | |
| **D** | | 0.333679 | | 0.355955 | | 0.282206 | 0.256642 | -1622.30931472 | - | |
| **int0 (BCF)** | | 0.154802 | | 0.184539 | | 0.093893 | 0.062534 | -2209.25863052 | - | |
| **int1** | | 0.338445 | | 0.358741 | | 0.288804 | 0.264650 | -1043.16823439 | - | |
| **int2** | | 0.498256 | | 0.547458 | | 0.414752 | 0.368098 | -3252.47225076 | - | |
| **int3** | | 0.499690 | | 0.548868 | | 0.414516 | 0.367350 | -3252.47848362 | - | |
| **int4** | | 0.500034 | | 0.548899 | | 0.417944 | 0.371864 | -3252.48161441 | - | |
| **int5** | | 0.319635 | | 0.338356 | | 0.271486 | 0.248416 | -1041.99233487 | - | |
| **int6** | | 0.597176 | | 0.653300 | | 0.503734 | 0.451061 | -3774.36323085 | - | |
| **int7** | | 0.597461 | | 0.653457 | | 0.505031 | 0.452717 | -3774.3686957 | - | |
| **int8** | | 0.598873 | | 0.654646 | | 0.507279 | 0.455322 | -3774.37015039 | - | |
| **int9** | | 0.418495 | | 0.444165 | | 0.359360 | 0.329937 | -1563.89219735 | - | |
| **int10** | | 0.696467 | | 0.759394 | | 0.596094 | 0.538381 | -4296.24795837 | - | |
| **int11** | | 0.381925 | | 0.422457 | | 0.311317 | 0.272292 | -2729.40847637 | - | |
| **int12** | | 0.340302 | | 0.371945 | | 0.276666 | 0.243485 | -2001.87252333 | - | |
| **int13** | | 0.158588 | | 0.175416 | | 0.113447 | 0.092249 | -1250.60877693 | - | |
| **TS1** | | 0.337800 | | 0.357473 | | 0.289543 | 0.266066 | -1043.16832282 | -38.13 | |
| **TS2** | | 0.495354 | | 0.544759 | | 0.412330 | 0.365763 | -3252.44288307 | -33.51 | |
| **TS3** | | 0.494401 | | 0.543992 | | 0.410469 | 0.363539 | -3252.44780024 | -275.40 | |
| **TS4** | | 0.487670 | | 0.536911 | | 0.404343 | 0.357711 | -3252.43931323 | 224.50 | |
| **TS5** | | 0.594512 | | 0.650452 | | 0.501777 | 0.449399 | -3774.34911216 | -19.45 | |
| **TS6** | | 0.593955 | | 0.650079 | | 0.502156 | 0.450003 | -3774.35016907 | -241.12 | |
| **TS7** | | 0.592364 | | 0.647762 | | 0.502529 | 0.451251 | -3774.33695073 | -107.33 | |
| **TS8** | | 0.695821 | | 0.757765 | | 0.598656 | 0.542326 | -4296.22056344 | -50.18 | |
| **TS9** | | 0.694262 | | 0.757155 | | 0.593126 | 0.535199 | -4296.23619406 | -231.82 | |
| **TS10** | | 0.378303 | | 0.419632 | | 0.304721 | 0.264484 | -2729.36376405 | -29.30 | |
| **TS11** | 0.496963 | | 0.546108 | | 0.414423 | | 0.368104 | -3252.39290082 | | -187.35 |
| **1b** | 0.146096 | | 0.154074 | | 0.114456 | | 0.101127 | -327.056396692 | | - |
| **1b’** | 0.127562 | | 0.133825 | | 0.098637 | | 0.086862 | -271.681038319 | | - |
| **int1b** | 0.262992 | | 0.279693 | | 0.217064 | | 0.195648 | -850.133961221 | | - |
| **int2b** | 0.423694 | | 0.468951 | | 0.343636 | | 0.299755 | -3059.43478421 | | - |
| **int3b** | 0.423806 | | 0.469367 | | 0.342859 | | 0.298594 | -3059.4391402 | | - |
| **int4b** | 0.424652 | | 0.469741 | | 0.345864 | | 0.303092 | -3059.44830832 | | - |
| **int5b** | 0.244464 | | 0.259232 | | 0.201226 | | 0.181395 | -848.957070287 | | - |
| **int6b** | 0.522080 | | 0.574190 | | 0.433499 | | 0.384071 | -3581.32981493 | | - |
| **int7b** | 0.522486 | | 0.574883 | | 0.431355 | | 0.381103 | -3581.32982514 | | - |
| **int11b** | 0.306429 | | 0.343243 | | 0.238577 | | 0.202024 | -2536.37044197 | | - |
| **TS1b** | 0.262859 | | 0.278546 | | 0.219405 | | 0.199143 | -850.131643559 | | -37.06 |
| **TS2b** | 0.419832 | | 0.465609 | | 0.337903 | | 0.293251 | -3059.40456419 | | -30.53 |
| **TS3b** | 0.419581 | | 0.465028 | | 0.338789 | | 0.294605 | -3059.40239091 | | -434.99 |
| **TS4b** | 0.413589 | | 0.458920 | | 0.333878 | | 0.290057 | -3059.4023288 | | -147.82 |
| **TS5b** | 0.518019 | | 0.570751 | | 0.427114 | | 0.376742 | -3581.3102213 | | -16.09 |
| **TS6b** | 0.518903 | | 0.571007 | | 0.429716 | | 0.380185 | -3581.29988643 | | -457.25 |
| **TS10b** | 0.302402 | | 0.340264 | | 0.230398 | | 0.192176 | -2536.32276459 | | -17.69 |
| **1c** | 0.174058 | | 0.183328 | | 0.140818 | | 0.126438 | -366.390371609 | | - |
| **1c’** | 0.156568 | | 0.164782 | | 0.124434 | | 0.110859 | -311.011976713 | | - |
| **int1c** | 0.290844 | | 0.308695 | | 0.244311 | | 0.222204 | -889.467363386 | | - |
| **int2c** | 0.451495 | | 0.498179 | | 0.369905 | | 0.324949 | -3098.77033752 | | - |
| **int3c** | 0.451730 | | 0.498702 | | 0.366424 | | 0.320200 | -3098.77164817 | | - |
| **int4c** | 0.452621 | | 0.499067 | | 0.372474 | | 0.328064 | -3098.78059644 | | - |
| **int5c** | 0.271802 | | 0.288192 | | 0.226601 | | 0.205486 | -888.292421939 | | - |
| **int6c** | 0.549882 | | 0.603455 | | 0.459058 | | 0.408313 | -3620.65999633 | | - |
| **int7c** | 0.550267 | | 0.603964 | | 0.457223 | | 0.405668 | -3620.66223195 | | - |
| **int11c** | 0.334344 | | 0.372512 | | 0.265526 | | 0.228103 | -2575.70520503 | | - |
| **TS1c** | 0.290729 | | 0.307753 | | 0.245697 | | 0.224375 | -889.4665549845 | | -19.76 |
| **TS2c** | 0.447818 | | 0.494957 | | 0.365011 | | 0.319512 | -3098.73903198 | | -14.45 |
| **TS3c** | 0.447130 | | 0.494329 | | 0.363775 | | 0.318073 | -3098.73694397 | | -286.79 |
| **TS4c** | 0.440052 | | 0.486880 | | 0.358444 | | 0.313405 | -3098.73660400 | | -183.70 |
| **TS5c** | 0.546429 | | 0.600166 | | 0.455087 | | 0.404125 | -3620.64420050 | | -15.59 |
| **TS6c** | 0.545912 | | 0.599879 | | 0.454123 | | 0.402935 | -3620.63736674 | | -219.63 |
| **TS10c** | 0.330909 | | 0.369850 | | 0.258325 | | 0.219450 | -2575.65637323 | | -34.38 |
| **1d** | 0.201868 | | 0.212313 | | 0.167704 | | 0.152518 | -405.722580156 | | - |
| **1d’** | 0.184641 | | 0.194135 | | 0.151134 | | 0.136584 | -350.343420934 | | - |
| **int1d** | 0.318679 | | 0.337765 | | 0.269766 | | 0.246370 | -928.798275732 | | - |
| **int2d** | 0.479544 | | 0.527398 | | 0.397113 | | 0.351384 | -3138.09800235 | | - |
| **int3d** | 0.479588 | | 0.527625 | | 0.396082 | | 0.349943 | -3138.10291953 | | - |
| **int4d** | 0.480747 | | 0.528317 | | 0.400071 | | 0.355000 | -3138.11032114 | | - |
| **int5d** | 0.300056 | | 0.317447 | | 0.254975 | | 0.233446 | -927.622319694 | | - |
| **int6d** | 0.574557 | | 0.630670 | | 0.481153 | | 0.428594 | -3659.97978623 | | - |
| **int7d** | 0.578085 | | 0.633137 | | 0.483833 | | 0.431383 | -3659.98899866 | | - |
| **int11d** | 0.362582 | | 0.401722 | | 0.293958 | | 0.256151 | -2615.03064080 | | - |
| **TS1d** | 0.318647 | | 0.336987 | | 0.271455 | | 0.248889 | -928.797968729 | | -5.7 |
| **TS2d** | 0.475489 | | 0.523904 | | 0.391277 | | 0.344787 | -3138.07134626 | | -21.21 |
| **TS3d** | 0.473058 | | 0.522469 | | 0.387471 | | 0.340184 | -3138.06818642 | | -78.11 |
| **TS4d** | 0.467685 | | 0.515537 | | 0.386046 | | 0.340541 | -3138.06348879 | | -525.04 |
| **TS5d** | 0.573419 | | 0.629020 | | 0.478588 | | 0.425768 | -3659.97007349 | | -25.65 |
| **TS6d** | 0.574210 | | 0.629363 | | 0.482479 | | 0.430795 | -3659.97276295 | | -200.28 |
| **TS10d** | 0.358118 | | 0.398451 | | 0.283264 | | 0.243080 | -2614.98823655 | | -12.67 |
| **C_6_F_5_-SiH_3_** | | 0.074886 | | 0.086379 | | 0.038532 | 0.022326 | -1019.45193551 | - | |
| **TS1(C_6_F_5_-SiH_3_)** | | 0.298066 | | 0.322104 | | 0.243546 | 0.216364 | -1539.55087866 | -23.22 | |
| **TS2(C_6_F_5_-SiH_3_)** | | 0.455377 | | 0.508980 | | 0.368111 | 0.318534 | -3748.82634612 | -26.23 | |
| **TS3(C_6_F_5_-SiH_3_)** | | 0.454932 | | 0.508559 | | 0.367636 | 0.318036 | -3748.82937776 | -278.27 | |
| **TS10(C_6_F_5_-SiH_3_)** | | 0.378303 | | 0.419632 | | 0.304721 | 0.264484 | -2729.36376405 | -29.30 | |
| **1,3,5-C_6_H_3_**Br_2_**SiH_3_** | | 0.095019 | | 0.105885 | | 0.055905 | 0.039119 | -5670.23392799 | - | |
| **TS1(1,3,5-C_6_H_3_Br_2_SiH_3_)** | | 0.317371 | | 0.340166 | | 0.263493 | 0.237044 | -6190.33627864 | -49.94 | |
| **TS2(1,3,5-C_6_H_3_Br_2_SiH_3_)** | | 0.475331 | | 0.527687 | | 0.387715 | 0.338566 | -8399.61118500 | -15.43 | |
| **TS3(1,3,5-C_6_H_3_Br_2_SiH_3_)** | | 0.474320 | | 0.526740 | | 0.385762 | 0.336277 | -8399.61327839 | -281.88 | |
| **TS10(1,3,5-C_6_H_3_Br_2_SiH_3_)** | | 0.378303 | | 0.419632 | | 0.304721 | 0.264484 | -2729.36376405 | -29.30 | |
| **1,3,5-C_6_H_3_Cl_2_SiH_3_** | | 0.096191 | | 0.106536 | | 0.059210 | 0.043286 | -1442.32588998 | - | |
| **TS1(1,3,5-C_6_H_3_Cl_2_SiH_3_)** | | 0.318578 | | 0.340855 | | 0.266419 | 0.240700 | -1962.4285139 | -47.48 | |
| **TS2(1,3,5-C_6_H_3_Cl_2_SiH_3_)** | | 0.476515 | | 0.528257 | | 0.390721 | 0.342365 | -4171.70223593 | -26.35 | |
| **TS3(1,3,5-C_6_H_3_Cl_2_SiH_3_)** | | 0.47570 | | 0.527475 | | 0.389518 | 0.341014 | -4171.70468417 | -281.33 | |
| **TS10(1,3,5-C_6_H_3_Cl_2_SiH_3_)** | | 0.378303 | | 0.419632 | | 0.304721 | 0.264484 | -2729.36376405 | -29.30 | |
| **1,3,5-C_6_H_3_F_2_SiH_3_** | | 0.099044 | | 0.108615 | | 0.064017 | 0.048998 | -721.635834317 | - | |
| **TS1(1,3,5-C_6_H_3_F_2_SiH_3_)** | | 0.321807 | | 0.342973 | | 0.272452 | 0.248022 | -1241.73542085 | -23.14 | |
| **TS2(1,3,5-C_6_H_3_F_2_SiH_3_)** | | 0.479271 | | 0.530226 | | 0.394597 | 0.346886 | -3451.00928513 | -37.37 | |
| **TS3(1,3,5-C_6_H_3_F_2_SiH_3_)** | | 0.478426 | | 0.529492 | | 0.393282 | 0.345369 | -3451.01242234 | -281.95 | |
| **TS10(1,3,5-C_6_H_3_F_2_SiH_3_)** | | 0.378303 | | 0.419632 | | 0.304721 | 0.264484 | -2729.36376405 | -29.30 | |
| **1,3,5-C_6_H_3_Me_2_SiH_3_** | | 0.169413 | | 0.181181 | | 0.129037 | 0.111460 | -601.740198806 | - | |
| **TS1(1,3,5-C_6_H_3_Me_2_SiH_3_)** | | 0.392681 | | 0.415675 | | 0.341274 | 0.315459 | -1121.84205832 | -3.11 | |
| **TS2(1,3,5-C_6_H_3_Me_2_SiH_3_)** | | 0.550345 | | 0.603125 | | 0.464146 | 0.415214 | -3331.11559235 | -19.04 | |
| **TS3(1,3,5-C_6_H_3_Me_2_SiH_3_)** | | 0.548759 | | 0.601867 | | 0.460940 | 0.411369 | -3331.11764203 | -280.20 | |
| **TS10(1,3,5-C_6_H_3_Me_2_SiH_3_)** | | 0.378303 | | 0.419632 | | 0.304721 | 0.264484 | -2729.36376405 | -29.30 | |
| **int2-4** | | 0.498839 | | 0.548258 | | 0.414612 | 0.367657 | -3252.4755898 | - | |
| **Int6-8** | | 0.59751 | | 0.653509 | | 0.505958 | 0.453923 | -3774.36669778 | - | |
| **TS_dissociation-1_** | | 0.498476 | | 0.547218 | | 0.4147 | 0.368143 | -3252.47159206 | -55.18 | |
| **TS _association-1_** | | 0.499349 | | 0.547439 | | 0.417799 | 0.372185 | -3252.48034278 | -17.43 | |
| **TS _dissociation-2_** | | 0.597513 | | 0.652709 | | 0.507583 | 0.456364 | -3774.3655008 | -35.17 | |
| **TS _association-2_** | | 0.598698 | | 0.653539 | | 0.508773 | 0.457692 | -3774.37010631 | -4.66 | |

1. **Cartesian coordinates and structures of all the compounds optimized in this study**

**
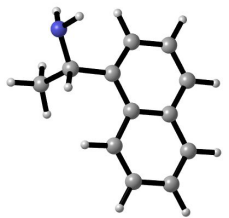
**

**1a**

**G_gas_ = -519.331073 a.u.**

**E_gas_ = -519.516787812 a.u.**

C 2.10314200 -0.77765900 -0.23807600

H 1.78842400 -1.54977300 -0.96156800

C 0.95783700 0.23912100 -0.12684600

C -0.41472600 -0.19394900 -0.10375100

C 1.22945400 1.59051900 -0.01400700

C -0.80304600 -1.56075800 -0.23046200

C -1.45774800 0.78612700 0.04606000

C 0.20250700 2.55468000 0.13631700

H 2.26654200 1.93093200 -0.04535100

C -2.13047200 -1.93839100 -0.20594800

H -0.04166900 -2.33153100 -0.35304100

C -2.81647500 0.36270400 0.06864100

C -1.11545000 2.16191200 0.16785900

H 0.46647400 3.61165700 0.22566200

C -3.15091600 -0.96891900 -0.05232400

H -2.39634000 -2.99369900 -0.30772000

H -3.59696200 1.12007300 0.18400000

H -1.91592400 2.89769600 0.28316600

H -4.19880600 -1.27877100 -0.03290200

C 2.34419700 -1.46997800 1.11264600

H 2.67720300 -0.72970400 1.85973900

H 1.42788300 -1.93773900 1.49991600

H 3.12691000 -2.23665900 1.00945200

N 3.37796400 -0.26416800 -0.72713300

H 3.25304100 0.27167300 -1.58560300

H 3.80764000 0.36256200 -0.04646500

**
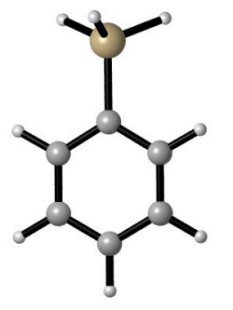
**

**PhSiH_3_**

**G_gas_ = -522.630150 a.u.**

**E_gas_ = -522.712865948 a.u.**

C -1.65448400 1.20960100 0.00347500

C -0.25640300 1.20734700 -0.01083000

C 0.46863500 0.00010700 -0.01545100

C -0.25630100 -1.20729900 -0.01081200

C -1.65428000 -1.20974000 0.00347100

C -2.35594900 -0.00007200 0.01133700

H -2.19822700 2.15806800 0.00515800

H 0.27436000 2.16449500 -0.02310300

H 0.27449300 -2.16446300 -0.02310700

H -2.19795800 -2.15824500 0.00515700

H -3.44913800 -0.00017900 0.02000900

Si 2.35267700 0.00003100 0.00644800

H 2.88060200 -0.01732000 1.40445600

H 2.86634800 -1.21121300 -0.69792200

H 2.86473300 1.22876400 -0.66806900

**
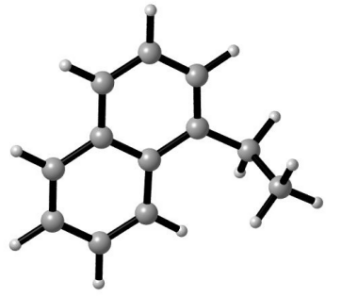
**

**A**

**G_gas_ = -464.0398570 a.u.**

**E_gas_ = -464.209233536 a.u.**

C 1.26218900 0.18612600 -0.30166400

C -0.11530900 -0.20327300 -0.16962300

C 1.58852500 1.52804600 -0.19853100

C -0.54933800 -1.55780400 -0.26890600

C -1.11161600 0.80708500 0.06736300

C 0.60773500 2.52249400 0.03531000

H 2.63513200 1.82974500 -0.30055800

C -1.88061100 -1.89762200 -0.13961400

H 0.18236700 -2.34550500 -0.45304000

C -2.47615700 0.42227800 0.19602800

C -0.71647500 2.17013600 0.16730900

H 0.91017200 3.57026000 0.10916200

C -2.85679800 -0.89878300 0.09709000

H -2.18519500 -2.94418900 -0.22148900

H -3.22326300 1.20057800 0.37546000

H -1.48141500 2.93015700 0.34838200

H -3.90846500 -1.17855600 0.19844400

C 2.75770100 -1.55744100 0.79994900

H 3.12819100 -0.83400500 1.54341800

H 1.90152600 -2.08265400 1.25115000

H 3.55351100 -2.29727100 0.61605300

C 2.35634500 -0.84158100 -0.50017500

H 2.04805800 -1.58693600 -1.25239700

H 3.24223300 -0.33959700 -0.92180700

**
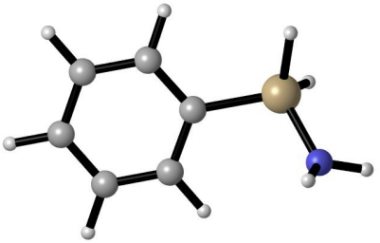
**

**B**

**G_gas_ = -577.958168 a.u.**

**E_gas_ = -578.058774406 a.u.**

N 2.75761100 -0.84429000 0.38551200

H 2.60156900 -1.16334000 1.33766000

Si 1.91141800 0.55471800 -0.20442000

H 2.16606900 1.84793100 0.51312000

H 2.37284700 0.73934300 -1.61334800

C 0.06428300 0.21790500 -0.07910600

C -0.86009200 1.26351600 0.10297600

C -0.43031900 -1.09638600 -0.18400900

C -2.23343900 1.00895200 0.16751700

H -0.50447600 2.29430400 0.20262400

C -1.80225500 -1.35629000 -0.11482800

H 0.27391000 -1.92362500 -0.31238800

C -2.70635400 -0.30292400 0.05819900

H -2.93645900 1.83436500 0.30899500

H -2.16831400 -2.38353500 -0.19576500

H -3.77964700 -0.50462100 0.11195300

H 3.72043800 -1.00548100 0.10594700

**
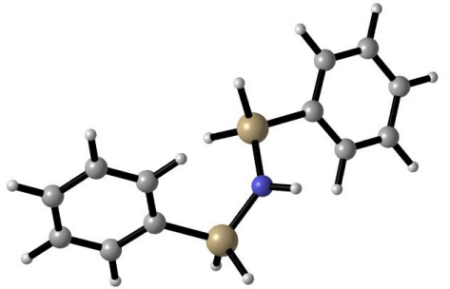
**

**C**

**G_gas_ = -1099.424683 a.u.**

**E_gas_ = -1099.61145310 a.u.**

Si -1.32282900 -1.61672000 0.37092200

H -1.11747400 -2.40902200 -0.88000200

C -2.81087100 -0.49930200 0.08567500

C -3.81044000 -0.83809800 -0.84531700

C -2.95779100 0.69802800 0.81176100

C -4.92466000 -0.01583500 -1.03823400

H -3.71664400 -1.75443300 -1.43728700

C -4.06800500 1.52559900 0.62001700

H -2.18750800 0.99168500 1.53145900

C -5.05473500 1.16803000 -0.30463600

H -5.69018700 -0.29485100 -1.76732700

H -4.16302400 2.45314800 1.19106600

H -5.92286400 1.81479000 -0.45761000

N 0.08410500 -0.68035700 0.81852400

H 0.51345200 -0.93438400 1.70554000

H -1.55869600 -2.57117700 1.49583600

Si 0.84252300 0.63508200 -0.04664000

H 0.23018900 0.63783500 -1.40756100

H 0.59814200 1.97617300 0.56999600

C 2.70537200 0.37143100 -0.10046300

C 3.24519700 -0.92894400 -0.10464700

C 3.59580900 1.46015100 -0.15585100

C 4.62635200 -1.13570200 -0.16795800

H 2.57338800 -1.79096200 -0.05058500

C 4.97774700 1.25876400 -0.22277300

H 3.20760100 2.48385900 -0.14024600

C 5.49486100 -0.04093000 -0.22940500

H 5.02703400 -2.15305600 -0.16886300

H 5.65375500 2.11707500 -0.26461600

H 6.57537600 -0.20039600 -0.27844700

**
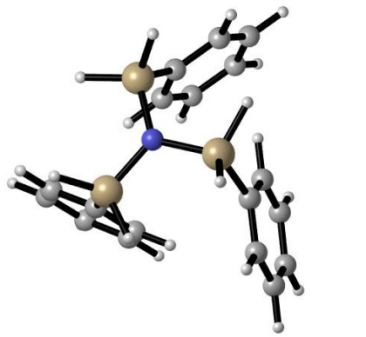
**

**D**

**G_gas_ = -1620.891175 a.u.**

**E_gas_ = -1621.17338036 a.u.**

Si 1.18886700 -1.29546700 2.07179700

H 2.53168800 -0.67664600 2.26844800

C 1.08540400 -2.11715600 0.38059600

C 0.45272500 -3.36503900 0.22408100

C 1.52351500 -1.44385400 -0.77759600

C 0.24883200 -3.91746600 -1.04526600

H 0.09959300 -3.91095500 1.10441200

C 1.32252900 -1.99242500 -2.04632800

H 2.01073000 -0.46948400 -0.69745100

C 0.68019800 -3.22837900 -2.18319400

H -0.25068100 -4.88464500 -1.14649200

H 1.66143400 -1.44683200 -2.93077900

H 0.51719600 -3.65524000 -3.17649800

N -0.00100500 -0.00010800 2.21687800

H 0.94476600 -2.34196800 3.10609400

Si 0.52590200 1.67797400 2.07138400

H -0.68156600 2.53155100 2.26676800

H 1.55344700 1.99042900 3.10630300

C 1.29054500 1.99849500 0.38060300

C 0.48954400 2.03988200 -0.77846300

C 2.68766900 2.07559500 0.22545300

C 1.06637100 2.13960300 -2.04666900

H -0.59795400 1.97389300 -0.69950700

C 3.26927800 2.17483700 -1.04336000

H 3.33610200 2.04389500 1.10649000

C 2.45799000 2.20233300 -2.18214900

H 0.42534100 2.15913800 -2.93181200

H 4.35669700 2.22666400 -1.14349900

H 2.91012600 2.27421600 -3.17504500

Si -1.71763700 -0.38287900 2.07056600

H -1.85305200 -1.85535000 2.26612200

C -2.37654900 0.11855700 0.37927600

C -2.01056000 -0.59601600 -0.77923200

C -3.14221300 1.28960000 0.22310300

C -2.38453800 -0.14702400 -2.04790500

H -1.40925300 -1.50442700 -0.69935000

C -3.51812100 1.74302500 -1.04619800

H -3.43995900 1.86720700 1.10368100

C -3.13511100 1.02638600 -2.18441400

H -2.08003500 -0.71218300 -2.93258100

H -4.10699400 2.65857400 -1.14716400

H -3.42284500 1.38147900 -3.17766900

H -2.50263000 0.35092800 3.10490300

**
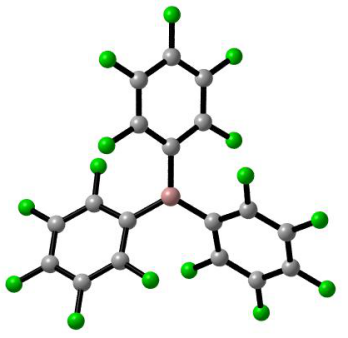
**

**int0**

**G_gas_ = -2206.554092 a.u.**

**E_gas_ = -2206.64798533 a.u.**

B -0.00012000 -0.00058900 0.00025300

C 0.27280600 1.54551900 0.00010700

C -0.54656400 2.44818100 0.69966000

C 1.35178800 2.11292700 -0.69942000

C -0.31494500 3.82301100 0.71895400

C 1.60533600 3.48387700 -0.71840800

C 0.76678100 4.34142700 0.00038300

C 1.20256900 -1.00983200 0.00003800

C 1.15473300 -2.22841200 -0.69885700

C 2.39434700 -0.75058800 0.69868700

C 2.21565700 -3.13297500 -0.71783200

C 3.46962000 -1.63804400 0.71787600

C 3.37788100 -2.83452700 0.00008400

C -1.47566300 -0.53696900 -0.00001900

C -1.84864000 -1.69711400 0.70044400

C -2.50599900 0.11365900 -0.70059400

C -3.15527100 -2.18343300 0.71929700

C -3.82019800 -0.35181300 -0.72001000

C -4.14440000 -1.50608600 -0.00046300

F 2.52886200 0.37464300 1.40522200

F 4.57228100 -1.36064300 1.40467900

F 4.39182800 -3.68513200 0.00020100

F 2.13436500 -4.26712600 -1.40456500

F 0.07016100 -2.55650300 -1.40559000

F -0.94266700 -2.37566900 1.40909300

F -3.46796400 -3.27559800 1.40770300

F -5.38839600 -1.95781500 -0.00061400

F -4.76065000 0.28532700 -1.40857200

F -2.24632900 1.21566200 -1.40881700

F 2.17727900 1.33730500 -1.40681300

F 2.62783800 3.98016800 -1.40595500

F 0.99709200 5.64472400 0.00054300

F -1.10556400 4.63950400 1.40655600

F -1.58796500 2.00227300 1.40683700

**
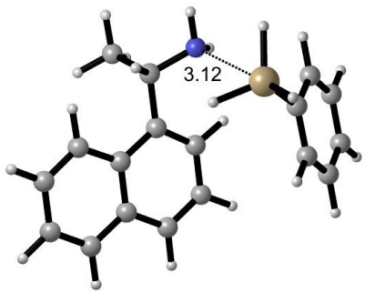
**

**Int1**

**G_gas_ = -1041.953145 a.u.**

**E_gas_ = -1042.24194847 a.u.**

Si -1.95902400 -1.35692700 2.08341700

H -2.38796400 -1.00457500 3.47686500

C -2.77437300 -0.12954800 0.90542900

C -3.53099200 -0.55791800 -0.20063400

C -2.60215000 1.25393100 1.08960400

C -4.08138300 0.35994600 -1.10026100

H -3.68161200 -1.62758000 -0.37085600

C -3.14964700 2.17788300 0.19392600

H -2.01669800 1.62469100 1.93675400

C -3.88716800 1.73242700 -0.90720400

H -4.66458300 0.00531800 -1.95472400

H -2.99468600 3.24845700 0.35311300

H -4.31377400 2.45203100 -1.61110800

N -0.78024300 -2.26454800 -0.65557400

H -1.23401200 -1.93988700 -1.51069400

H -0.93139200 -3.27258200 -0.62346300

C 0.65725100 -1.98445300 -0.68449000

H 1.06450100 -2.46050200 0.22293300

C 1.36062200 -2.59907700 -1.91269400

H 0.94644600 -2.16322100 -2.83688800

H 2.44407600 -2.40599200 -1.91038000

H 1.20416800 -3.69042400 -1.94369700

C 0.95039700 -0.49024200 -0.59205700

C 2.22895100 -0.03046400 -0.11666500

C 0.01618400 0.44267900 -1.00318600

C 3.24948800 -0.90734600 0.35661500

C 2.50285700 1.38192300 -0.09841200

C 0.28906200 1.83258100 -0.98364000

H -0.97293300 0.12369800 -1.33087300

C 4.46197700 -0.42756300 0.80947500

H 3.07611200 -1.98379300 0.37151200

C 3.76400600 1.84349300 0.37254100

C 1.50735200 2.29503100 -0.54547100

H -0.48698100 2.52814100 -1.31025700

C 4.72735500 0.96295000 0.81576900

H 5.22171800 -1.12632800 1.16905500

H 3.95532100 2.92029800 0.37833600

H 1.72636900 3.36615200 -0.52701000

H 5.69086500 1.33304800 1.17507400

H -2.42261800 -2.74608400 1.81117700

H -0.47303100 -1.26945200 2.06445700

**
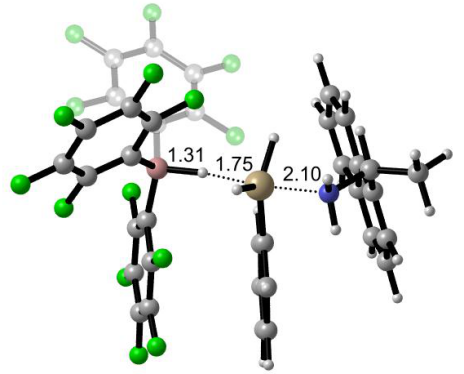
**

**Int2**

**G_gas_ = -3248.521746 a.u.**

**E_gas_ = -3248.93649833 a.u.**

Si 0.86350400 -0.39044000 1.78138300

H -0.38417600 -0.02372500 0.60430300

C 1.74976500 -1.51622700 0.57338300

C 1.88093700 -2.88080700 0.89632100

C 2.21465600 -1.07711100 -0.67811600

C 2.46537600 -3.78033200 0.00166500

H 1.48200000 -3.26726200 1.84042900

C 2.79568200 -1.97687700 -1.57508500

H 2.11592500 -0.03048900 -0.96345500

C 2.92093900 -3.32796100 -1.24039000

H 2.53483500 -4.83951100 0.25979300

H 3.14267900 -1.61927300 -2.54754300

H 3.35548500 -4.03230300 -1.95392600

B -1.43824600 0.02778000 -0.16373300

C -2.66479500 -0.24459200 0.86275300

C -2.69446000 0.39695000 2.10261000

C -3.75564400 -1.07453500 0.59255600

C -3.71224900 0.22401600 3.04198900

C -4.80009400 -1.27773500 1.49880700

C -4.77837200 -0.62219800 2.73189700

C -1.44146400 1.54895000 -0.73457400

C -2.62178600 2.16841000 -1.15613900

C -0.28652500 2.31802400 -0.87363800

C -2.66732400 3.47419800 -1.65336100

C -0.28023300 3.62497500 -1.36175600

C -1.48527200 4.21104800 -1.75151600

C -1.10944700 -1.13380700 -1.24379100

C -0.82958600 -0.92202500 -2.59672900

C -0.94002300 -2.45120700 -0.80456600

C -0.38734300 -1.93741900 -3.45297000

C -0.49736700 -3.49126900 -1.61612000

C -0.21226300 -3.22800000 -2.95679600

N 2.29912700 -0.63813400 3.30037600

H 2.68827800 -1.58156400 3.20032700

H 1.80247000 -0.63392800 4.19727700

C 3.42309300 0.36125500 3.35025900

H 2.92573600 1.33373700 3.44215000

C 4.28872300 0.15495600 4.59572700

H 4.77496400 -0.83301500 4.59775800

H 5.08073400 0.91742800 4.62948100

H 3.68831800 0.24535000 5.51611300

C 4.20229200 0.30598800 2.04284500

C 4.12695600 1.36075700 1.07045800

C 4.94428600 -0.82992800 1.76136100

C 3.41027900 2.57822100 1.26490500

C 4.79097900 1.17356600 -0.19395800

C 5.61822600 -0.99524900 0.53209000

H 5.00175000 -1.64095700 2.49288100

C 3.30911800 3.52212000 0.26698300

H 2.90633300 2.77268200 2.21158500

C 4.66632800 2.17029600 -1.20323400

C 5.53500400 -0.01312000 -0.42892300

H 6.18074700 -1.91175400 0.34304500

C 3.92937800 3.31305600 -0.98783200

H 2.72038900 4.42626900 0.43067800

H 5.16065000 2.00297200 -2.16392400

H 6.03021800 -0.14015100 -1.39501200

H 3.81391800 4.05741100 -1.77832300

H -0.14447700 -1.04147400 2.64718500

H 1.07368400 1.06325200 1.85238700

F -5.81877200 -2.08224900 1.19713900

F -5.76513500 -0.80528200 3.60686500

F -3.67721500 0.85433800 4.21829200

F -1.68926200 1.23157500 2.44698800

F -3.84561300 -1.71584400 -0.57905400

F -1.15347600 -2.74778900 0.49160600

F -0.30249300 -4.71495200 -1.12088200

F 0.24577300 -4.19824600 -3.74600600

F -0.11179200 -1.67583400 -4.73190100

F -0.94904900 0.29591800 -3.14654900

F 0.91583300 1.79857200 -0.54852700

F 0.86073200 4.31850200 -1.46067900

F -1.50511000 5.45680500 -2.21992200

F -3.82032900 4.01897300 -2.03855100

F -3.78284900 1.50020200 -1.11873000

**
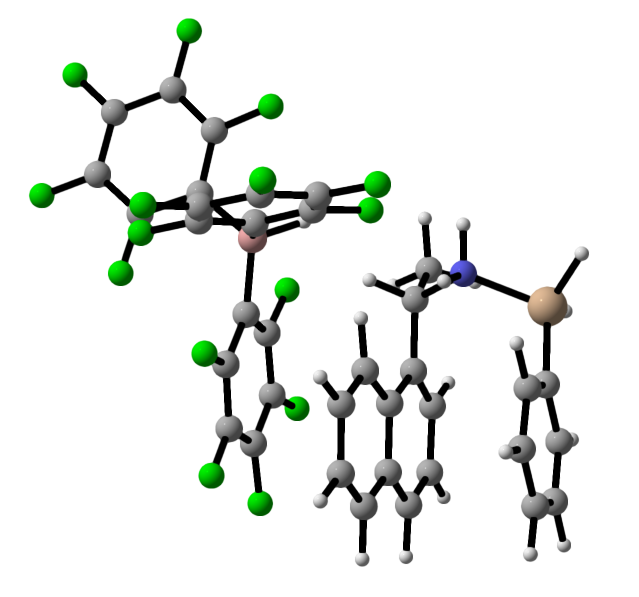
**

**Int3**

**G_gas_ = -3248.517922 a.u.**

**E_gas_ = -3248.93243873 a.u.**

Si -3.75034700 0.93025800 3.21410700

H -3.96409800 -0.26082900 4.06264100

C -4.86962100 1.03757500 1.74831400

C -5.79686200 0.01345100 1.47827000

C -4.75171900 2.10900800 0.83948000

C -6.58535000 0.05914300 0.32725700

H -5.88898000 -0.83992000 2.15468400

C -5.52958500 2.14441100 -0.31776100

H -4.03989900 2.92108300 1.02042200

C -6.44688200 1.11954300 -0.57277100

H -7.29530200 -0.74538900 0.12315500

H -5.41462700 2.96742000 -1.02623900

H -7.05095100 1.14528400 -1.48283200

C -1.46109900 -0.57909000 2.06998400

H -0.51675300 -0.32490300 1.56784400

C -2.43492100 -1.16397300 1.06510400

C -2.63264000 -0.51174900 -0.20263600

C -3.10935300 -2.34266000 1.33362000

C -1.93589200 0.66374000 -0.59709800

C -3.55117600 -1.08844500 -1.14305300

C -3.99561000 -2.91874400 0.39346100

H -2.94489000 -2.86728700 2.27466000

C -2.14697000 1.25409000 -1.82677900

H -1.15877300 1.08138700 0.03702500

C -3.74961100 -0.45788200 -2.40106500

C -4.21758600 -2.30051500 -0.81491000

H -4.49022400 -3.86261100 0.63203100

C -3.06873900 0.69267000 -2.73808200

H -1.56200800 2.13208100 -2.10747900

H -4.44070900 -0.91862800 -3.11115300

H -4.89393000 -2.74381400 -1.54966500

H -3.21525800 1.15285300 -3.71753100

C -1.10834400 -1.44010200 3.27288900

H -1.97250900 -1.66987700 3.91387600

H -0.67200700 -2.37917100 2.91169600

H -0.33557200 -0.94172800 3.87914000

N -1.98417000 0.78554900 2.56605600

H -1.89257300 1.44296200 1.77889900

H -3.63027500 2.17894300 4.00824200

B 1.81641100 -0.03659200 0.33740700

C 0.93195000 -1.07535800 -0.57819700

C 0.51239900 -2.27690100 0.00222000

C 0.44191400 -0.86132400 -1.86904400

C -0.38355300 -3.16685500 -0.59068100

C -0.43729900 -1.73379200 -2.51769300

C -0.86925800 -2.88803900 -1.86733100

C 1.74497800 1.52864100 -0.15326700

C 2.50644000 2.03196100 -1.21526700

C 0.94554100 2.47803900 0.47604500

C 2.46012200 3.36343800 -1.63592000

C 0.84588700 3.81680200 0.09237100

C 1.62021300 4.26610800 -0.97607700

C 3.36433100 -0.50515200 0.54289000

C 4.05442500 -0.11177000 1.69320500

C 4.09476500 -1.28485900 -0.35680400

C 5.37864600 -0.46928500 1.95763900

C 5.42071800 -1.66971700 -0.13417400

C 6.06653900 -1.25736600 1.03259500

F 0.14418700 2.12858900 1.53222800

F 0.02294700 4.65560800 0.73466700

F 1.55604500 5.53829100 -1.36735900

F 3.19932500 3.78577700 -2.66090900

F 3.31190900 1.21569400 -1.90048200

F 3.44182300 0.65559700 2.61360000

F 5.99070700 -0.06967100 3.07673900

F 7.33175300 -1.61213600 1.26236200

F 6.07631300 -2.41920000 -1.02399400

F 3.53683200 -1.70241000 -1.50354000

F 0.74193300 0.26029900 -2.54394700

F -0.91852900 -1.43907200 -3.72882200

F -1.77010400 -3.68966500 -2.44025700

F -0.82564700 -4.23939000 0.07642100

F 0.92064700 -2.59473100 1.24961800

H 1.31086200 -0.06727000 1.45193600

H -1.30162100 1.14745600 3.24436800

**
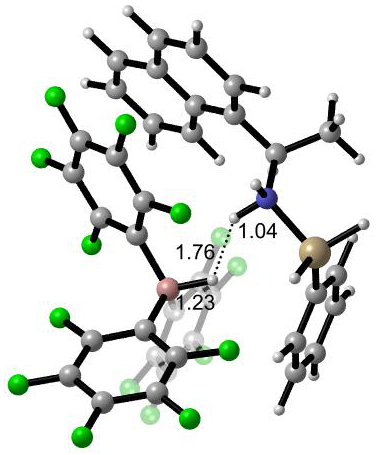
**

**Int4**

**G_gas_ = -3248.534459 a.u.**

**E_gas_ = -3248.95240306 a.u.**

H 0.80629900 -0.54451000 1.46412800

B -1.02633000 0.49319000 0.05275600

C -1.43786500 -0.76225500 -0.90811100

C -2.59696500 -0.90379000 -1.67554300

C -0.59565100 -1.87026200 -0.93735200

C -2.89669500 -2.06596100 -2.39925400

C -0.82890600 -3.03919600 -1.65022300

C -2.00825500 -3.14222400 -2.38757500

C 0.38233400 1.22475100 -0.37213300

C 1.09877300 1.95756000 0.57729800

C 0.93698300 1.23175600 -1.65628300

C 2.25970900 2.68002500 0.30175900

C 2.10567200 1.92613900 -1.98114300

C 2.76430100 2.66406500 -0.99759500

C -2.18615500 1.61525500 0.26746000

C -2.95821700 1.68457100 1.42618700

C -2.45507400 2.59827800 -0.68892400

C -3.93641300 2.65918300 1.64506400

C -3.41992900 3.59238200 -0.51973900

C -4.16451200 3.62351400 0.66281200

F 0.54360300 -1.84821300 -0.19383600

F 0.03266400 -4.06153900 -1.59658400

F -2.29388700 -4.26473100 -3.04782100

F -4.02935500 -2.15689400 -3.09699700

F -3.50524700 0.07835900 -1.74346300

F 0.35153800 0.55804500 -2.65359900

F 2.60622000 1.88234300 -3.21601500

F 3.89517700 3.30945200 -1.28755600

F 2.89785300 3.35177900 1.26354600

F 0.68206900 1.97258000 1.87180700

F -2.79559700 0.77732400 2.41735700

F -1.78141400 2.59478600 -1.84946000

F -3.64137700 4.50616000 -1.46531600

F -5.09028600 4.56354700 0.84766200

F -4.64650000 2.67798700 2.77578700

H -0.84160900 -0.01140900 1.15697500

N 1.26387300 -0.82038300 2.35376400

H 1.51120800 0.08643400 2.77120500

Si -0.17519700 -1.54077100 3.35858900

H -0.95924400 -0.33912700 3.69904800

H 0.50956200 -2.10544800 4.54657800

C 2.54211300 -1.58657700 2.02111000

H 2.23326200 -2.30958000 1.26155400

C -1.07224000 -2.78677300 2.32364500

C -2.36257300 -2.48524100 1.84292700

C -0.48863600 -4.02239900 1.97807000

C -3.04980900 -3.40110600 1.04261500

H -2.82140700 -1.52234200 2.07502000

C -1.16922500 -4.92618700 1.16139400

H 0.51155700 -4.28462500 2.33693400

C -2.45191800 -4.61536900 0.69525900

H -4.04492000 -3.15207600 0.66740200

H -0.69711400 -5.86911900 0.87755200

H -2.97770200 -5.31591400 0.04182600

C 3.55501300 -0.60563200 1.44368400

C 3.92978900 -0.61486500 0.05646400

C 4.11171800 0.33612700 2.29617400

C 3.40967800 -1.52680100 -0.90820300

C 4.87359100 0.37414600 -0.39940700

C 5.04436900 1.29494300 1.84386700

H 3.82912100 0.35235600 3.35314700

C 3.76302000 -1.44026800 -2.23851600

H 2.69549000 -2.29298100 -0.61716100

C 5.22050800 0.42919000 -1.77809100

C 5.41862600 1.30966500 0.52027700

H 5.44770000 2.02883600 2.54405100

C 4.67085300 -0.44974600 -2.68352200

H 3.32698300 -2.13885400 -2.95640700

H 5.91401100 1.20531000 -2.10948100

H 6.11983200 2.06096400 0.15080200

H 4.92442700 -0.38010400 -3.74342800

C 3.07944700 -2.35760800 3.22687000

H 4.04170700 -2.81091600 2.94984500

H 3.25301900 -1.70735100 4.09741500

H 2.40118800 -3.16660700 3.53508800

**
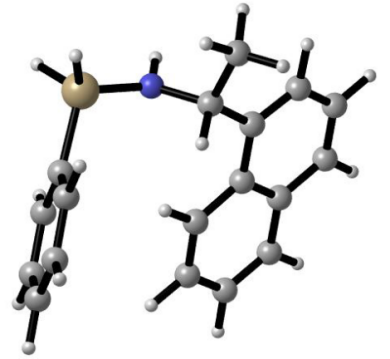
**

**Int5**

**G_gas_ = -1040.795746 a.u.**

**E_gas_ = -1041.06723177 a.u.**

N 0.16740900 -1.83375800 -0.84710300

H -0.44584000 -2.10020600 -1.61177300

Si 1.88384000 -1.95300700 -1.09546300

H 2.06296300 -2.04227300 -2.57255200

H 2.51374700 -3.14376200 -0.43632600

C -0.52330800 -1.78275700 0.44617500

H 0.13246200 -1.22432300 1.13765400

C 2.76573000 -0.44539900 -0.38976700

C 2.71333200 0.79853600 -1.05034200

C 3.43499200 -0.50919200 0.84644800

C 3.30358600 1.93615200 -0.49473400

H 2.19048400 0.88574600 -2.00761200

C 4.02197100 0.62862800 1.41170200

H 3.49968900 -1.46263000 1.38048300

C 3.95623100 1.85305900 0.74111200

H 3.25359300 2.89135500 -1.02444300

H 4.53194700 0.55904000 2.37625000

H 4.41476000 2.74350400 1.17938900

C -1.81663100 -0.97931800 0.30413800

C -1.76319200 0.45197500 0.15509500

C -3.04966200 -1.60630800 0.26835100

C -0.54378100 1.18721800 0.18252400

C -2.98566600 1.18588900 -0.02825000

C -4.25405500 -0.88113900 0.08396800

H -3.11434900 -2.68909700 0.38378800

C -0.52799300 2.55938800 0.03934200

H 0.40103100 0.66455000 0.30747700

C -2.93559000 2.60133600 -0.17207700

C -4.22445000 0.48594300 -0.06176300

H -5.20530000 -1.41900700 0.06056000

C -1.73487100 3.27802200 -0.14068500

H 0.42788700 3.08891500 0.06239800

H -3.87428400 3.14560400 -0.30913400

H -5.14866600 1.05299200 -0.20322900

H -1.71170200 4.36500800 -0.25346200

C -0.70973500 -3.18051300 1.05540000

H -1.23811100 -3.13934600 2.02112500

H -1.27199400 -3.84159900 0.37715300

H 0.27461500 -3.64519300 1.21862400

**
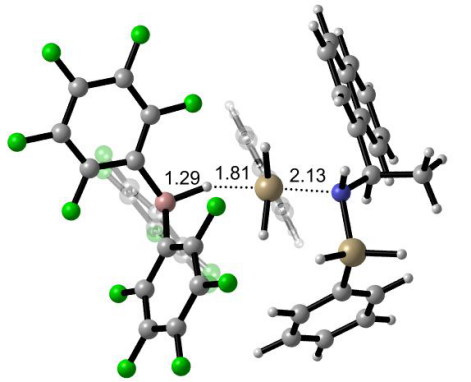
**

**Int6**

**G_gas_ = -3769.974816 a.u.**

**E_gas_ = -3770.47855030 a.u.**

Si 0.68323600 0.26682900 -1.26475500

H -0.87312400 -0.29777100 -0.54043800

C 1.62802700 -0.44131800 0.17872800

C 2.34738800 0.37590200 1.06909300

C 1.54623500 -1.82120600 0.44444400

C 2.97721600 -0.17086400 2.18913000

H 2.40145100 1.45266500 0.91341200

C 2.17889000 -2.36673400 1.56283000

H 0.97708400 -2.47349100 -0.22062100

C 2.89479800 -1.54303700 2.43765200

H 3.51793300 0.48296200 2.87683600

H 2.10777900 -3.43970100 1.75594100

H 3.38314500 -1.97154300 3.31611900

B -1.98262400 -0.23192900 0.10874500

C -2.59983300 1.22721500 -0.27130100

C -2.50863500 1.71389700 -1.57739600

C -3.21914800 2.08931000 0.63833000

C -2.92806100 2.98703300 -1.96698300

C -3.67386200 3.36576000 0.29339700

C -3.52551000 3.81962600 -1.01949400

C -2.87016900 -1.44097200 -0.50710200

C -4.25030300 -1.49107800 -0.29425800

C -2.34029900 -2.49292600 -1.25253700

C -5.06714800 -2.50576900 -0.79726500

C -3.11489200 -3.52942900 -1.77853300

C -4.49166000 -3.53276200 -1.54958500

C -1.48480500 -0.42209400 1.64354900

C -1.67552000 -1.56982200 2.41910500

C -0.67243500 0.55002000 2.23298900

C -1.08819900 -1.74915600 3.67767100

C -0.05637500 0.41422000 3.47210300

C -0.26539100 -0.75434400 4.20496000

H 0.10144600 1.60407100 -1.11459300

H 0.30816600 -0.61044800 -2.38396700

F -4.24821600 4.15406400 1.20203600

F -3.94535700 5.03596200 -1.36226100

F -2.75798600 3.40915300 -3.22300400

F -1.94820600 0.95125900 -2.54143900

F -3.38713100 1.72236800 1.91392800

F -0.39817500 1.68714300 1.55722400

F 0.76219800 1.36472200 3.93487200

F 0.33088900 -0.92469100 5.38386800

F -1.29204400 -2.87227400 4.36768700

F -2.42609000 -2.58811700 1.97452400

F -1.01253200 -2.56256000 -1.49248900

F -2.55482900 -4.51029800 -2.49007600

F -5.25047000 -4.51061200 -2.04080100

F -6.37969300 -2.50921800 -0.56602700

F -4.84282600 -0.54290300 0.44712200

N 2.25201500 1.12577300 -2.42302500

H 2.01176500 0.79118400 -3.36270500

Si 1.96006300 2.95737500 -2.43603600

H 0.56342300 3.11732300 -2.90446500

H 2.89998600 3.54658800 -3.42373100

C 3.65065000 0.65442800 -2.11134500

H 3.85632900 1.05874400 -1.11297800

C 2.28668000 3.58201200 -0.71078700

C 1.24327400 3.77427900 0.21603900

C 3.61639200 3.74662800 -0.26822100

C 1.51901300 4.07986100 1.55121100

H 0.20238300 3.64532000 -0.08766100

C 3.89310300 4.05782400 1.06506000

H 4.45003600 3.62365700 -0.96640400

C 2.84306300 4.21366200 1.97766300

H 0.69768900 4.17634100 2.26345300

H 4.92851700 4.17358000 1.39494700

H 3.05824600 4.43688400 3.02553600

C 3.74247500 -0.86528700 -2.03636600

C 4.61926600 -1.48112900 -1.07851300

C 3.02769200 -1.66485200 -2.90886300

C 5.44337000 -0.74352900 -0.18092200

C 4.67088000 -2.91596900 -1.01068500

C 3.07666900 -3.07783900 -2.83762400

H 2.37792900 -1.21892900 -3.66567600

C 6.26622500 -1.37867900 0.72613800

H 5.43006000 0.34648900 -0.19671400

C 5.51927100 -3.53963900 -0.05467200

C 3.87230300 -3.69025100 -1.89761000

H 2.47125300 -3.67156100 -3.52587800

C 6.30358700 -2.79118100 0.79648900

H 6.88504000 -0.78556500 1.40383500

H 5.54003000 -4.63175500 -0.00940400

H 3.90883300 -4.78027600 -1.82567600

H 6.95276200 -3.28354400 1.52451100

C 4.70026700 1.19125300 -3.09529700

H 5.68147500 0.76355500 -2.84332300

H 4.45944800 0.88447700 -4.12594300

H 4.79103400 2.28675800 -3.06970500

**
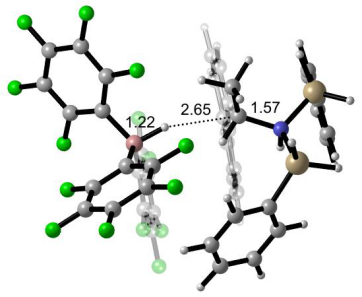
**

**Int7**

**G_gas_ = -3769.972964 a.u.**

**E_gas_ = -3770.47799461 a.u.**

Si 4.18687600 0.58697800 -2.94553200

H 3.60930600 -0.29321900 -3.97927600

C 5.21336400 -0.32876300 -1.69953900

C 5.42547300 -1.71308200 -1.82955800

C 5.73112800 0.33296900 -0.56685600

C 6.13999900 -2.41651600 -0.85649500

H 5.00988400 -2.25559500 -2.68220800

C 6.42933500 -0.37277200 0.41262500

H 5.58611800 1.41070800 -0.43491000

C 6.63583100 -1.74913500 0.26594600

H 6.29112800 -3.49281500 -0.96647700

H 6.80873600 0.14789900 1.29463000

H 7.17906600 -2.30281600 1.03552600

C 1.46486000 0.50046300 -1.91174500

H 0.79495600 1.05046900 -1.23777500

C 1.83650400 -0.82344700 -1.26498200

C 2.32849900 -0.82736200 0.08639100

C 1.77223900 -2.01683000 -1.96245600

C 2.31478900 0.32584100 0.92083100

C 2.86323200 -2.04190600 0.63008900

C 2.22082500 -3.23051100 -1.38921200

H 1.36244700 -2.03981000 -2.97225100

C 2.87513800 0.30525500 2.18293800

H 1.79113000 1.22709400 0.60577500

C 3.44195800 -2.02689600 1.92836400

C 2.78116700 -3.23791700 -0.13241400

H 2.13231100 -4.15684900 -1.96100600

C 3.46769500 -0.87555700 2.68603200

H 2.81368000 1.19660900 2.81100300

H 3.84300700 -2.95990700 2.33059500

H 3.15891100 -4.16620000 0.30307200

H 3.88864300 -0.88651700 3.69325800

C 0.78436200 0.42036100 -3.26781400

H 1.39987300 -0.07397700 -4.03169500

H -0.14780900 -0.14511300 -3.14371100

H 0.50373200 1.41793000 -3.62837400

N 2.77068400 1.36683500 -1.97608200

H 3.12835200 1.29575600 -1.00786100

H 4.86029600 1.76206700 -3.55332800

Si 2.50484100 3.23448600 -2.11738400

H 3.90041000 3.72903000 -2.21717300

H 1.73159000 3.45123300 -3.35795400

C 1.68873300 3.72970200 -0.53358000

C 2.46467300 3.76170100 0.64550300

C 0.29293300 3.89860400 -0.43317000

C 1.85343600 3.89654900 1.89353800

H 3.55325900 3.65181900 0.59679900

C -0.31387800 4.03023800 0.81721500

H -0.33100100 3.89360500 -1.32742900

C 0.46121200 4.00580200 1.98089700

H 2.46024800 3.89941300 2.80227600

H -1.39975300 4.10925700 0.88476600

H -0.02327900 4.04789200 2.95815300

B -1.76787800 -0.33771400 -0.05124300

C -1.08434800 -0.42441100 1.43420300

C -0.38269200 -1.57816000 1.80790400

C -1.01229400 0.61379000 2.36584300

C 0.29966600 -1.72063700 3.01408300

C -0.29506100 0.53905300 3.56422900

C 0.36457200 -0.64083100 3.89506900

C -2.70354000 0.98424800 -0.28876400

C -3.87889900 1.20295400 0.43651900

C -2.39923400 1.97335700 -1.21758600

C -4.68026800 2.33663900 0.28720300

C -3.15800500 3.13218500 -1.39960900

C -4.31089300 3.31828400 -0.63847700

C -2.62290500 -1.66210800 -0.49000800

C -2.73831900 -1.98130100 -1.84518000

C -3.28891000 -2.52971700 0.37964600

C -3.44370000 -3.08594100 -2.32743900

C -4.00780000 -3.64837400 -0.05332000

C -4.08812300 -3.92689700 -1.41868100

F -1.29097200 1.88271500 -1.99326800

F -2.76299700 4.07269300 -2.27197300

F -5.05328900 4.41522200 -0.79238800

F -5.78318700 2.50376800 1.01867800

F -4.25597800 0.30987900 1.36060800

F -2.14918500 -1.20126000 -2.77954000

F -3.51044000 -3.34339600 -3.63828300

F -4.77082800 -4.98869000 -1.85174800

F -4.61832800 -4.45161800 0.82147800

F -3.24622100 -2.32926300 1.70513900

F -1.62467800 1.79601500 2.14240500

F -0.20246400 1.60703700 4.37069800

F 1.09927800 -0.72294400 5.00874000

F 0.95641900 -2.84954100 3.30293300

F -0.31761500 -2.62455700 0.96647500

H -0.82686000 -0.27581900 -0.82348700

**
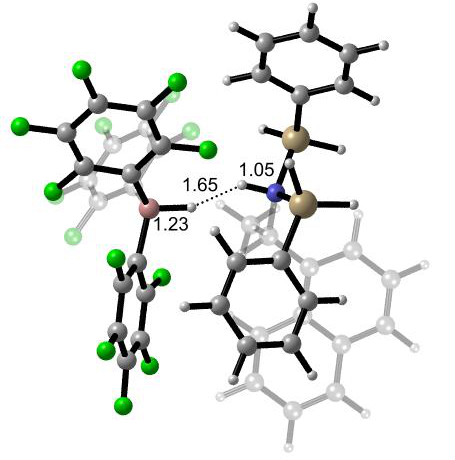
**

**Int8**

**G_gas_ = -3769.990383 a.u.**

**E_gas_ = -3770.49766249 a.u.**

H 0.08887000 1.51669400 0.41386700

B 1.07784100 -1.09615000 0.02696300

C 0.00110600 -2.24859400 -0.37770900

C -0.13993000 -2.80074400 -1.65191800

C -0.98347000 -2.64470700 0.53053500

C -1.16568000 -3.68242200 -2.00688500

C -2.01145900 -3.53659500 0.23380900

C -2.11924700 -4.04806200 -1.05939000

C 1.74208100 -1.24670200 1.51618700

C 2.23130600 -0.10313000 2.14333600

C 1.90896200 -2.42755800 2.24453900

C 2.80022700 -0.07399400 3.41511300

C 2.48228000 -2.46081900 3.52097200

C 2.93145300 -1.27672300 4.11192200

C 2.31115200 -0.85354300 -1.01158500

C 2.55591200 0.37447800 -1.61712000

C 3.27678900 -1.83561900 -1.24658100

C 3.68484500 0.64829800 -2.39264200

C 4.41245900 -1.62371300 -2.03036000

C 4.62169200 -0.36386300 -2.60182800

F -1.00622200 -2.10790300 1.77188600

F -2.93535900 -3.84641700 1.15117400

F -3.13194600 -4.85182800 -1.38251700

F -1.28120400 -4.10810200 -3.26804600

F 0.68249400 -2.43885400 -2.65386600

F 1.49586300 -3.59793100 1.74539000

F 2.60646600 -3.61100200 4.18137300

F 3.47371400 -1.29423700 5.32789800

F 3.20610500 1.07648700 3.96024600

F 2.14454000 1.09460200 1.50037600

F 1.67715200 1.39781500 -1.46971800

F 3.12386000 -3.04801000 -0.69639800

F 5.30376600 -2.59398800 -2.22925400

F 5.70618500 -0.13343000 -3.34004800

F 3.87928500 1.86323700 -2.92065200

H 0.41497900 -0.05943100 0.05068500

N -0.68377000 2.18491900 0.63638000

Si -1.20966000 2.75826900 -1.10629700

H 0.06284300 3.13019600 -1.74430600

H -2.06483000 3.94301000 -0.87393600

C -1.71168600 1.24886400 1.31687700

H -1.62269100 0.33844000 0.71335200

C -1.30107200 0.89357400 2.74553300

H -1.38846800 1.75583000 3.42433500

H -1.97961300 0.11095100 3.11235400

H -0.28064600 0.48854800 2.78293100

C -3.15185400 1.73274100 1.23121700

C -4.19873300 0.77633500 0.97749200

C -3.48806600 3.06336500 1.40553500

C -3.97159700 -0.62414000 0.86164600

C -5.54555100 1.25239300 0.81044800

C -4.82053300 3.52390100 1.27246300

H -2.71527000 3.79616200 1.63073000

C -4.99478800 -1.49613600 0.54989700

H -2.98128400 -1.03362200 1.04218200

C -6.57775100 0.32912000 0.48405700

C -5.82622300 2.63863900 0.96227200

H -5.03613900 4.58647000 1.40551500

C -6.30968900 -1.01558700 0.34384700

H -4.78162400 -2.56305800 0.47045000

H -7.59403000 0.70910600 0.34887500

H -6.85494100 2.98609700 0.83537600

H -7.11026700 -1.71454200 0.09001500

C -2.08698500 1.37223700 -1.97264400

C -1.35223700 0.33118200 -2.57729900

C -3.49314400 1.35494100 -2.06677800

C -2.01037500 -0.70031900 -3.25160800

H -0.26211900 0.31902800 -2.51865300

C -4.14903200 0.31492300 -2.72727700

H -4.08399800 2.14940100 -1.60459200

C -3.40711900 -0.71319000 -3.31834300

H -1.43021000 -1.49962300 -3.71486200

H -5.24052400 0.30209700 -2.76952300

H -3.91749300 -1.53270800 -3.83083000

Si 0.19899800 3.52281900 1.64101700

H -0.86823900 4.43044200 2.12017200

H 0.87344200 2.83149400 2.75360000

C 1.31745500 4.41333900 0.45389500

C 2.52237600 3.84278600 -0.00442800

C 0.90539700 5.64998200 -0.08503600

C 3.27234500 4.47254000 -0.99899600

H 2.86773900 2.89030100 0.39740200

C 1.66591100 6.28768400 -1.06767300

H -0.02312500 6.11748900 0.25641700

C 2.84336900 5.69234000 -1.53253000

H 4.18090100 3.99663700 -1.37317700

H 1.33570300 7.24530700 -1.47780400

H 3.42984200 6.18141400 -2.31452600


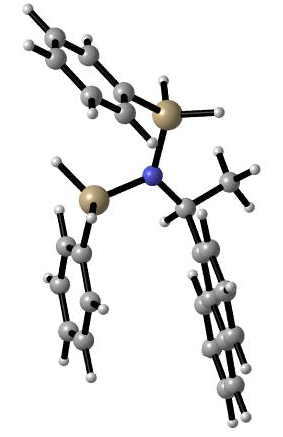


**Int9**

**G_gas_ = -1562.255285 a.u.**

**E_gas_ = -1562.61464532 a.u.**

N 1.07197900 -0.03161100 -0.91786400

Si 1.12960300 1.08321800 0.46438400

H 2.24055900 2.05233300 0.25279900

H 1.34929100 0.36788600 1.75512900

C -0.27946600 -0.38587500 -1.42098500

H -0.81385400 0.56153700 -1.58446100

C -0.26431600 -1.10743200 -2.77841000

H 0.18219000 -2.10918200 -2.70300800

H -1.29690500 -1.23566300 -3.13587500

H 0.28608200 -0.52379600 -3.53427300

C -1.09322500 -1.19464100 -0.40654100

C -2.52200700 -1.05656400 -0.33041800

C -0.45750800 -2.07854200 0.44627500

C -3.26605000 -0.16479500 -1.15674700

C -3.24612300 -1.82688500 0.64526200

C -1.17363900 -2.84782300 1.39482500

H 0.62864300 -2.17015300 0.39544500

C -4.63344800 -0.03622600 -1.02926300

H -2.74951500 0.44787800 -1.89544500

C -4.65726600 -1.67185600 0.74990700

C -2.54051000 -2.72235200 1.49653500

H -0.63147400 -3.53465900 2.04968900

C -5.34070300 -0.79701400 -0.06700400

H -5.17363500 0.66488900 -1.67034300

H -5.19308000 -2.26339600 1.49762900

H -3.10190400 -3.30417200 2.23264100

H -6.42410300 -0.68702500 0.02625600

C -0.50819600 2.00193100 0.58513700

C -0.79296400 3.09591800 -0.25498000

C -1.51202700 1.56893700 1.47145400

C -2.03797100 3.73092200 -0.21578900

H -0.03315500 3.45856800 -0.95505100

C -2.75895500 2.19864700 1.51362200

H -1.32713100 0.71036200 2.12305200

C -3.02382500 3.28016400 0.66870800

H -2.24046500 4.57845900 -0.87628400

H -3.53050700 1.83348700 2.19600700

H -4.00080500 3.77007600 0.69592400

Si 2.56133500 -0.63059000 -1.63182900

H 2.44507500 -2.08885900 -1.92649900

H 2.92936800 0.06739800 -2.90331900

C 3.96834100 -0.36637800 -0.41014900

C 4.99976100 0.55449200 -0.67247300

C 4.01823300 -1.09028600 0.79785000

C 6.04170400 0.75052300 0.23906500

H 4.98803200 1.13216500 -1.60160500

C 5.05555600 -0.89668200 1.71345300

H 3.23249200 -1.81505300 1.03315700

C 6.06945900 0.02610300 1.43463600

H 6.83259700 1.47209700 0.01756300

H 5.07425200 -1.46619000 2.64638400

H 6.88183400 0.17978700 2.14995700

**
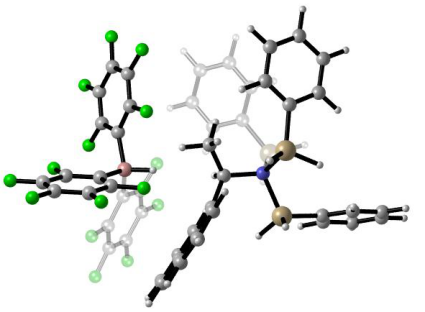
**

**int10**

**G_gas_ = -4291.403931 a.u.**

**E_gas_ = -4292.00002491 a.u.**

Si -3.90027600 2.43844500 0.28875000

C -5.45299800 2.53957900 -0.75406400

C -6.74603500 2.52851000 -0.19176800

C -5.33822700 2.72297800 -2.14824000

C -7.88114800 2.66046100 -0.99464900

H -6.87502800 2.42920500 0.88896500

C -6.47200700 2.85166400 -2.95408700

H -4.35113500 2.77269500 -2.61570600

C -7.74561700 2.81321700 -2.37854600

H -8.87435700 2.65126500 -0.53886300

H -6.36035400 2.98947900 -4.03229900

H -8.63368000 2.91519100 -3.00741200

N -3.34952300 0.62857600 0.55007100

C -1.80069200 0.56741700 0.91888700

H -1.30240800 0.44620200 -0.04848800

H -4.13997200 2.95037400 1.65665400

H -2.79546400 3.08316100 -0.43902800

Si -4.50108300 -0.06984900 1.85828000

H -3.96336000 0.36421300 3.16268000

Si -3.54226000 -0.25749400 -1.12305200

H -3.04333300 0.78631700 -2.05081800

H -5.00330800 -0.46764500 -1.23949300

C -2.54241100 -1.80450300 -1.30375200

C -1.23006700 -1.71909400 -1.81492500

C -3.06372900 -3.07682900 -0.98851700

C -0.45573800 -2.86798800 -1.98624200

H -0.78578900 -0.75935800 -2.08692000

C -2.28318400 -4.22204400 -1.15188700

H -4.08129000 -3.17989700 -0.61070000

C -0.97732200 -4.11884500 -1.64321300

H 0.55967200 -2.77193500 -2.37068200

H -2.69505900 -5.19940900 -0.88928600

H -0.35865600 -5.01294900 -1.74834500

C -4.84127400 -1.89780600 1.73424800

C -4.03800000 -2.88811900 2.33372700

C -6.00116400 -2.30447400 1.04040600

C -4.36935800 -4.23958500 2.22235300

H -3.13898500 -2.61408600 2.88797100

C -6.33274300 -3.65721400 0.92788600

H -6.65328900 -1.55888000 0.57667800

C -5.51280200 -4.62628000 1.51492700

H -3.72894400 -4.99389100 2.68515800

H -7.23096200 -3.95472100 0.38154600

H -5.76721400 -5.68525200 1.42452100

H -5.76598800 0.63858000 1.56201200

C -1.25890200 1.83643700 1.55735400

C -0.38713200 2.70880500 0.81456800

C -1.45898600 2.07272500 2.90941800

C -0.14818800 2.59164400 -0.58380800

C 0.28950300 3.76588400 1.51636900

C -0.82241600 3.13523000 3.58493400

H -2.09133500 1.40181800 3.49161600

C 0.70650400 3.44771300 -1.24255900

H -0.63385100 1.80758700 -1.16391800

C 1.19648400 4.60869400 0.81301800

C 0.05253800 3.95143200 2.90398600

H -0.99891200 3.27857300 4.65303400

C 1.40724500 4.45545000 -0.53830200

H 0.87077500 3.32304700 -2.31389200

H 1.72964200 5.38182500 1.37218900

H 0.58222200 4.75311600 3.42512100

H 2.12628800 5.08586900 -1.06552000

C -1.46021600 -0.67657100 1.72866300

H -1.73095200 -1.59811900 1.19876900

H -0.37458800 -0.68469600 1.87235400

H -1.91824500 -0.66859900 2.72789100

B 2.37799700 -0.23873400 -0.09556600

C 2.76370400 0.75206000 -1.34462000

C 2.22917600 0.52155800 -2.61566100

C 3.52059000 1.92413300 -1.25248200

C 2.43966400 1.33750200 -3.72703300

C 3.75618300 2.78183200 -2.33263200

C 3.21947700 2.48672000 -3.58384700

C 3.19225900 0.07744800 1.28641300

C 4.56808700 -0.14757600 1.38671900

C 2.58876100 0.56057600 2.44440900

C 5.31036700 0.10255300 2.54179400

C 3.28475000 0.83147800 3.62620600

C 4.65827700 0.59650400 3.67537000

C 2.55075300 -1.83821700 -0.43528700

C 1.81606500 -2.77120300 0.30052900

C 3.35568600 -2.38921700 -1.43765900

C 1.80647900 -4.14039300 0.03512900

C 3.37626400 -3.75442100 -1.74681500

C 2.58537200 -4.63617400 -1.00858100

F 1.26221400 0.80733600 2.48077600

F 2.64728500 1.30867000 4.70258300

F 5.34521400 0.84432000 4.79267300

F 6.62651900 -0.11975600 2.58032200

F 5.24066300 -0.59890800 0.31591000

F 1.01202300 -2.36462000 1.30368200

F 1.00682400 -4.96952900 0.71924400

F 2.54799000 -5.93709400 -1.31356100

F 4.14010700 -4.22207600 -2.73678600

F 4.13895100 -1.60749300 -2.19312100

F 4.03075700 2.33360900 -0.07975000

F 4.44082400 3.91824800 -2.15872100

F 3.42009600 3.30365400 -4.62046600

F 1.89509400 1.04267900 -4.91216800

F 1.43354000 -0.55389200 -2.82583300

H 1.17929400 -0.05911300 0.10396100

**
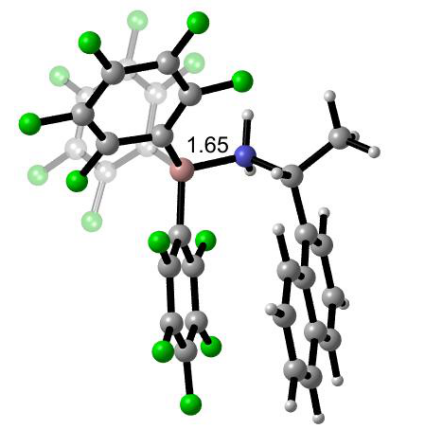
**

**int11**

**G_gas_ = -2725.930048 a.u.**

**E_gas_ = -2726.24136521 a.u.**

C -0.97609500 0.65620600 2.38999000

H -0.88282600 1.60995700 1.86569200

N 0.18276600 -0.18065200 1.88640300

H 1.00349400 0.02976900 2.45980500

H -0.02625300 -1.17012800 2.04992600

C 1.41486600 1.33953800 0.00860500

C 1.76482500 2.32924300 0.92522100

C 1.88487500 1.55816600 -1.29376200

C 2.48772300 3.47988100 0.59394800

C 2.60573900 2.68716800 -1.67867900

C 2.91279200 3.65907500 -0.72133000

C -0.66937300 -0.39479300 -0.56707300

C -1.31012800 -1.62867300 -0.41487300

C -1.33082300 0.50542500 -1.40467000

C -2.50657400 -1.97533200 -1.03267300

C -2.52680800 0.20050600 -2.06300800

C -3.12478400 -1.04111900 -1.86637600

C 1.85704400 -1.23470500 0.17012800

C 1.97858500 -2.13429800 -0.89681200

C 2.90567200 -1.28476100 1.09535900

C 3.04449500 -3.03234300 -1.02084600

C 3.98705300 -2.16241600 1.01858400

C 4.05503500 -3.04812100 -0.05831100

F 2.77784000 4.39009800 1.52208700

F 1.41120000 2.21838500 2.22884200

F 3.60256900 4.74222400 -1.06310900

F 3.01139400 2.84424700 -2.93577400

F 1.63086400 0.64657100 -2.24228000

F 2.91392100 -0.42563100 2.14809700

F 4.94366600 -2.15574300 1.94430400

F 5.07111100 -3.89702200 -0.16454900

F 3.10472700 -3.87009100 -2.05228500

F 1.06628400 -2.17831700 -1.87158200

F -0.77741500 -2.54360300 0.42712000

F -3.08835100 -3.15089800 -0.79917700

F -4.29812800 -1.31986700 -2.42633900

F -3.13547300 1.11859900 -2.81179700

F -0.88190200 1.75814900 -1.57451300

B 0.67377000 -0.08987600 0.31272700

C -2.31003100 -0.00081800 2.06852100

C -3.29723700 0.65233100 1.25663600

C -2.57350400 -1.26253400 2.57630900

C -3.13235500 1.95552400 0.70273100

C -4.50461500 -0.06331700 0.93708800

C -3.76498700 -1.95655400 2.27008800

H -1.83597700 -1.76224000 3.21152700

C -4.08341300 2.50963100 -0.12711300

H -2.22478200 2.52446800 0.90289700

C -5.46238200 0.53522200 0.07032200

C -4.70817400 -1.36984000 1.45706400

H -3.92176400 -2.96204900 2.66592400

C -5.25873200 1.79163800 -0.45491000

H -3.91588400 3.50040200 -0.55490700

H -6.36121500 -0.03202700 -0.18364500

H -5.62155600 -1.90663000 1.18954000

H -5.99389100 2.23234400 -1.13181000

C -0.80920000 0.91392400 3.89044700

H 0.12740000 1.45199500 4.09708200

H -0.81593800 -0.02700800 4.46286900

H -1.64673300 1.52806800 4.25159500

**
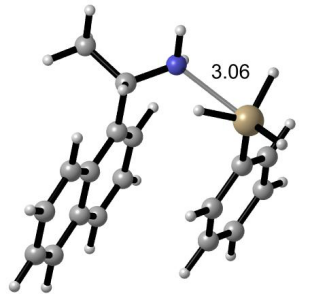
**

**TS1**

**G_gas_ = -1041.951512 a.u.**

**E_gas_ = -1042.241055200 a.u.**

Si 2.75336700 1.17205300 1.73040500

H 3.32164200 0.85335400 3.08309200

C 2.27658500 -0.44043900 0.88553900

C 3.05493000 -0.97243500 -0.15756100

C 1.13574700 -1.15681100 1.29164500

C 2.71196700 -2.18233300 -0.76939900

H 3.94105900 -0.43260900 -0.50542600

C 0.78786200 -2.36495700 0.68258800

H 0.49216600 -0.76345600 2.08445700

C 1.57769700 -2.88214700 -0.34811600

H 3.33080200 -2.57881900 -1.57934900

H -0.11286400 -2.89391900 1.00278500

H 1.30170200 -3.82351100 -0.83059200

N 1.51535300 2.19027700 -0.88092400

H 1.78044100 1.82600900 -1.79553900

H 1.90355800 3.13229000 -0.82639300

C 0.05969800 2.24397100 -0.71816900

H -0.09769200 2.56118600 0.32265800

C -0.63235900 3.27676200 -1.62954000

H -0.48625100 3.01185400 -2.68966300

H -1.71671700 3.31517500 -1.43804000

H -0.21782600 4.28542900 -1.46253200

C -0.58395600 0.87394300 -0.90739100

C -1.65120600 0.42626100 -0.05446500

C -0.19632600 0.07066400 -1.96507700

C -2.12567000 1.16937900 1.06667800

C -2.27772800 -0.84150700 -0.32592100

C -0.81540800 -1.17195600 -2.23234600

H 0.61409000 0.38929300 -2.62476900

C -3.13847500 0.69322200 1.87403000

H -1.67893400 2.13497100 1.30426700

C -3.32063700 -1.30300400 0.52588000

C -1.84035200 -1.61894100 -1.43190200

H -0.46593400 -1.77547100 -3.07325400

C -3.74449000 -0.55799300 1.60481200

H -3.47396100 1.28556800 2.72912000

H -3.78217600 -2.26997400 0.30665100

H -2.32280400 -2.58053700 -1.62625500

H -4.54455400 -0.92725300 2.25128900

H 3.81780600 1.89054100 0.97614100

H 1.57456100 2.04912500 1.98443800


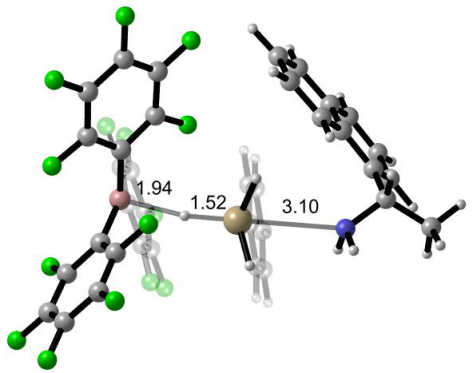


**TS2**

**G_gas_ = -3248.510976 a.u.**

**E_gas_ = -3248.92330581 a.u.**

Si 0.56536900 -0.21245800 1.76511900

H -0.74262100 -0.09028000 0.99948800

C 1.67451900 -1.28741300 0.70039600

C 1.80548500 -2.65852600 0.99770400

C 2.34945700 -0.78361200 -0.42665500

C 2.58441200 -3.49865600 0.19711500

H 1.27991100 -3.08384600 1.85800500

C 3.12235600 -1.62395300 -1.23123500

H 2.28398500 0.27405600 -0.68085700

C 3.23954000 -2.98301100 -0.92506200

H 2.66290500 -4.56161700 0.43761500

H 3.64041800 -1.21055900 -2.09935400

H 3.83703000 -3.63963500 -1.56215200

B -2.03694200 0.04793600 -0.44320200

C -3.19235000 -0.55252300 0.46019700

C -3.45965400 -0.04797300 1.74020800

C -4.02455900 -1.59211600 0.02596100

C -4.46883500 -0.55073800 2.56033100

C -5.05435700 -2.11469600 0.81020200

C -5.27540600 -1.58918800 2.08566100

C -1.95674100 1.62751800 -0.58372100

C -3.10166400 2.43686100 -0.52841700

C -0.74438500 2.31084100 -0.75692200

C -3.05527900 3.82976900 -0.63045700

C -0.65418200 3.69733500 -0.85088400

C -1.82143600 4.46357900 -0.78626800

C -1.20843300 -0.89874500 -1.39630700

C -0.68943500 -0.49312000 -2.63684700

C -0.89130400 -2.21450100 -1.01560100

C 0.10697000 -1.31654100 -3.43368700

C -0.11873300 -3.07333000 -1.79198900

C 0.40102400 -2.61107800 -3.00154100

N 3.22702800 -0.42448600 3.34042500

H 3.27710000 -1.37343000 2.96565300

H 2.87638800 -0.50424400 4.29653800

C 4.57658500 0.17114800 3.34355400

H 4.46047400 1.21133600 3.69343100

C 5.52427300 -0.52009600 4.33350500

H 5.62246800 -1.59597100 4.11643100

H 6.53012100 -0.07167600 4.31946800

H 5.12513100 -0.42786500 5.35653600

C 5.09871500 0.23193300 1.90957800

C 4.56292900 1.19954200 0.98946000

C 6.05662600 -0.65724500 1.45663800

C 3.55547900 2.13768400 1.35312500

C 5.03456700 1.21523500 -0.36818700

C 6.52774800 -0.63424400 0.12018000

H 6.47300000 -1.40381900 2.13416000

C 3.02043900 3.01208100 0.43066000

H 3.16962700 2.13997100 2.37115300

C 4.46527500 2.13362400 -1.29502600

C 6.03107100 0.28513700 -0.77323900

H 7.28656900 -1.35566600 -0.19283300

C 3.46980300 3.00668000 -0.91175700

H 2.22846700 3.70252300 0.72552800

H 4.82470000 2.12341900 -2.32788800

H 6.38420600 0.30579600 -1.80791700

H 3.01557400 3.68792800 -1.63404700

H 0.21006900 -0.91320200 3.02869800

H 0.98402100 1.18698700 1.98049200

F -5.82587500 -3.09805300 0.35565300

F -6.24795400 -2.07517400 2.84567500

F -4.67595200 -0.05133200 3.77574500

F -2.71681700 0.95193400 2.23272500

F -3.86187300 -2.12269000 -1.19082800

F -1.29859700 -2.68678300 0.16813200

F 0.15588700 -4.30652200 -1.37895500

F 1.17654400 -3.39785000 -3.73428800

F 0.59406900 -0.87820900 -4.59198800

F -0.95211800 0.72540400 -3.12030800

F 0.40298000 1.62432200 -0.82752200

F 0.52455700 4.30390900 -0.99937500

F -1.75559800 5.78414600 -0.87968100

F -4.17018200 4.55190200 -0.58143200

F -4.31488300 1.89276900 -0.39364400

**
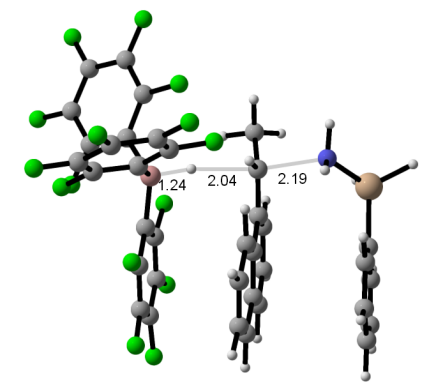
**

**TS3**

**G_gas_ = -3248.493212 a.u.**

**E_gas_ = -3248.90368096 a.u.**

Si -3.94039200 0.85285400 -3.58239700

H -3.67346100 2.30472400 -3.40336900

C -4.96910800 0.17506000 -2.18024500

C -5.73098400 1.04261400 -1.37665700

C -4.99904800 -1.20331100 -1.88870200

C -6.50117000 0.54992200 -0.31927100

H -5.71298900 2.12013400 -1.56261400

C -5.75681500 -1.69658000 -0.82581500

H -4.41985400 -1.91443400 -2.48636800

C -6.51140700 -0.81890900 -0.04058300

H -7.08272100 1.23976900 0.29722700

H -5.75098700 -2.76577300 -0.60302800

H -7.10090600 -1.20391300 0.79508300

C -0.97097400 0.67616200 -1.97841100

H -0.54107900 -0.30655100 -2.10363300

C -1.86011200 0.90169900 -0.85932300

C -2.29039100 -0.19377500 -0.02066400

C -2.28805500 2.19993900 -0.56351400

C -1.87356300 -1.53675700 -0.21034100

C -3.15061400 0.09410800 1.09065800

C -3.13617900 2.46797900 0.52226100

H -1.94138000 3.03555500 -1.17083100

C -2.26623500 -2.53388400 0.66153500

H -1.19221300 -1.79350500 -1.01795500

C -3.54568300 -0.95566900 1.96359200

C -3.55750200 1.43215700 1.33320400

H -3.43190600 3.49546900 0.73924300

C -3.10808600 -2.24592400 1.76007800

H -1.89809400 -3.55137500 0.51287500

H -4.17686700 -0.71242100 2.82150400

H -4.19271700 1.63585800 2.19867200

H -3.39026800 -3.04000800 2.45429200

C -0.29452900 1.79216300 -2.71104200

H -1.01501900 2.51441300 -3.12196900

H 0.34607700 2.33342600 -1.99625100

H 0.35242700 1.40790900 -3.50825800

N -2.34964900 0.01682400 -3.54149300

H -2.41457800 -0.97182400 -3.29285200

H -4.54622200 0.63214400 -4.92743100

B 1.48204700 0.02265000 0.06960800

C 0.64238000 0.34669800 1.42039800

C 0.16647400 1.64619800 1.63048700

C 0.17044300 -0.59893700 2.33692800

C -0.68775900 2.00705500 2.66917400

C -0.69221100 -0.28578400 3.39220400

C -1.12806100 1.02610100 3.55813900

C 2.03841900 -1.49980200 -0.10113400

C 3.00756100 -1.99567300 0.77664000

C 1.67304000 -2.36707000 -1.12622500

C 3.56273500 -3.27131500 0.67225200

C 2.19614300 -3.65461100 -1.27802600

C 3.15289500 -4.10820500 -0.37045000

C 2.68476700 1.05860400 -0.29916300

C 3.08089600 1.18652700 -1.63365800

C 3.40244200 1.83696500 0.61291900

C 4.10138100 2.03666600 -2.06338900

C 4.43459400 2.70029300 0.23200000

C 4.78712600 2.79943600 -1.11580700

F 0.74963400 -2.00436000 -2.05783900

F 1.79367000 -4.44498200 -2.27778100

F 3.66885100 -5.33065000 -0.49298700

F 4.47511900 -3.70067500 1.54399400

F 3.42388700 -1.22549500 1.79141700

F 2.45962300 0.46009400 -2.58917600

F 4.42671500 2.12496200 -3.35612500

F 5.76766300 3.61812300 -1.49566800

F 5.08750900 3.42797000 1.13909500

F 3.11416400 1.79006000 1.92001400

F 0.48341400 -1.89896500 2.21360300

F -1.15824700 -1.24433400 4.19767300

F -2.01929800 1.32741800 4.50645900

F -1.15266100 3.25609500 2.77387600

F 0.48427100 2.62462200 0.75691500

H 0.63829100 0.20850700 -0.81702500

H -1.78841800 0.08116300 -4.39285700


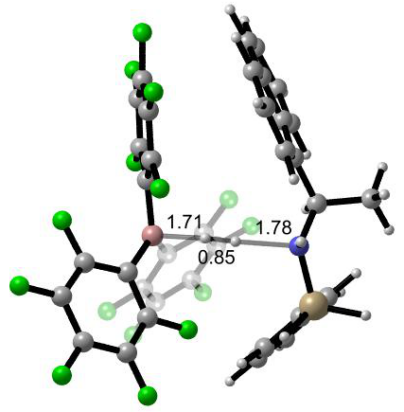


**TS4**

**G_gas_ = -3248.514832 a.u.**

**E_gas_ = -3248.91917421 a.u.**

H 0.15849300 -0.04172900 -1.56364500

B 0.53921600 1.14685600 0.56999700

C 1.18171400 -0.14773100 1.23093500

C 2.39658000 -0.14271600 1.92728000

C 0.52314600 -1.38010000 1.18136400

C 2.91091400 -1.28182900 2.55248500

C 0.99702400 -2.53810600 1.78730100

C 2.20047500 -2.48078400 2.49115800

C -0.98904100 1.48444900 0.84989500

C -1.72040300 2.28384100 -0.04145800

C -1.71312300 0.98099500 1.94084900

C -3.06947500 2.58016100 0.12751200

C -3.07520700 1.22766400 2.12688000

C -3.75278700 2.03464700 1.21470000

C 1.52286500 2.28704200 0.05771500

C 2.61690400 1.97988600 -0.76046600

C 1.37519800 3.63757500 0.39451000

C 3.50872600 2.93714500 -1.23905600

C 2.25090300 4.62921300 -0.05552400

C 3.32320000 4.27494600 -0.87734100

F -0.63791700 -1.47291000 0.52264000

F 0.32837300 -3.68612400 1.68634100

F 2.69166600 -3.57374800 3.06454600

F 4.08273900 -1.24152400 3.18455600

F 3.11558600 0.98002600 2.04025000

F -1.11707700 0.22362200 2.86876500

F -3.73286900 0.68784700 3.14983600

F -5.05998400 2.24107800 1.34711400

F -3.72446400 3.32362000 -0.76243600

F -1.12830000 2.76918900 -1.13965300

F 2.83361400 0.70704600 -1.13157700

F 0.37408100 4.03207600 1.18836300

F 2.07706200 5.90061200 0.29464000

F 4.16345200 5.20454400 -1.31458700

F 4.52667600 2.59349100 -2.02469200

H 0.21706000 0.60808500 -1.01560500

N -0.07114700 -1.23913700 -2.86663500

H -0.34035000 -0.57033700 -3.58930800

Si 1.53598900 -1.91555900 -3.22601900

H 2.39426500 -0.73460100 -3.50062700

H 1.48501000 -2.78561800 -4.44077500

C -1.22745700 -2.05018900 -2.43155100

H -0.88473800 -2.57430900 -1.53038700

C 2.20202200 -2.91866700 -1.79191300

C 3.31534300 -2.46624700 -1.05629200

C 1.66518300 -4.18348000 -1.47741100

C 3.88154900 -3.26025900 -0.05465900

H 3.74440300 -1.48362300 -1.26347100

C 2.22021700 -4.97057600 -0.46563200

H 0.80596500 -4.57077700 -2.03357300

C 3.33763200 -4.51410200 0.23982900

H 4.73988800 -2.89125200 0.51281300

H 1.78193000 -5.94268200 -0.22760800

H 3.77145200 -5.12528600 1.03395500

C -2.42215500 -1.17240400 -2.05667300

C -3.28949400 -1.52588700 -0.96560200

C -2.72412000 -0.05212100 -2.81242700

C -3.08933400 -2.66969900 -0.13769300

C -4.41153900 -0.67510200 -0.66668700

C -3.84664800 0.76286400 -2.53840900

H -2.08814000 0.23547500 -3.65356200

C -3.90769600 -2.92842800 0.94157300

H -2.25394600 -3.34197500 -0.33073400

C -5.22890400 -0.96277000 0.46304000

C -4.67343500 0.45875300 -1.48243700

H -4.03388000 1.64740100 -3.15039800

C -4.98125500 -2.06022500 1.25736700

H -3.71515700 -3.80290700 1.56792900

H -6.04975100 -0.28126800 0.69912300

H -5.51948000 1.10409100 -1.23515500

H -5.60745200 -2.26201900 2.12956900

C -1.65967700 -3.10033400 -3.47115200

H -2.51336400 -3.68330500 -3.09311200

H -1.97255500 -2.60953400 -4.40648300

H -0.84235300 -3.80049700 -3.70723900


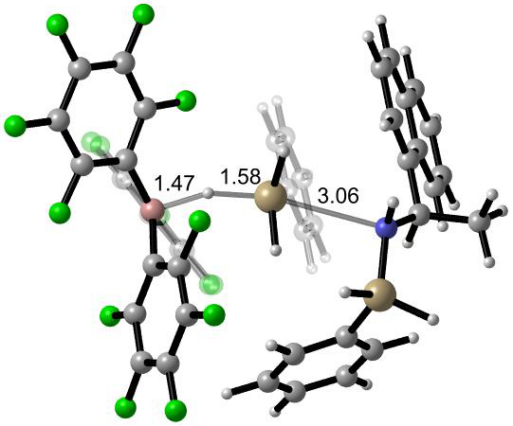


**TS5**

**G_gas_ = -3769.972273 a.u.**

**E_gas_ = -3770.47404961 a.u.**

Si -0.34411100 -0.02910700 -1.17711500

H 1.04293100 0.37874900 -0.54544300

C -1.54799500 0.67008000 0.05281000

C -2.28461700 -0.16995700 0.90867700

C -1.65205600 2.06509900 0.21533000

C -3.10585200 0.37051200 1.89883000

H -2.20178100 -1.25293800 0.82019200

C -2.47059600 2.60229600 1.21068500

H -1.08860800 2.73870200 -0.43318300

C -3.19674400 1.75661500 2.05463200

H -3.66705900 -0.29526500 2.55773900

H -2.54731900 3.68615400 1.32436500

H -3.84068100 2.17963500 2.82895500

B 2.25477100 0.29028000 0.28485800

C 2.62707700 -1.26314900 0.08691400

C 2.62127100 -1.82586600 -1.19393100

C 2.89618000 -2.15386800 1.13046000

C 2.80459300 -3.18552500 -1.44393200

C 3.07718600 -3.52383100 0.92821400

C 3.03875800 -4.04349300 -0.36692400

C 3.13599000 1.33724300 -0.55385200

C 4.49348400 1.13272200 -0.81653300

C 2.58555800 2.50963700 -1.08002400

C 5.26396400 2.03035000 -1.56227700

C 3.31218400 3.42916900 -1.83321500

C 4.66700600 3.18320700 -2.07561300

C 1.61231100 0.76841700 1.67612400

C 1.87192200 2.00336800 2.28562600

C 0.63683400 -0.01075100 2.31319400

C 1.19650100 2.44455600 3.42844300

C -0.05644100 0.38446700 3.45255400

C 0.22133400 1.63366200 4.01054100

H -0.15433300 -1.48066200 -1.18660400

H -0.25872400 0.65085700 -2.48770000

F 3.25566000 -4.34298800 1.96434400

F 3.19440700 -5.34660300 -0.57057200

F 2.73264300 -3.67020900 -2.68204800

F 2.35959100 -1.04585600 -2.25864500

F 2.95478400 -1.72436100 2.39517600

F 0.30030900 -1.20369800 1.79393100

F -0.98530600 -0.40066400 3.99461700

F -0.43559100 2.04476400 5.08893300

F 1.47553200 3.63139300 3.96346100

F 2.79601800 2.83582100 1.79290200

F 1.28768100 2.79046600 -0.85679700

F 2.73732300 4.52852000 -2.31698200

F 5.38335400 4.04638700 -2.78732600

F 6.55537100 1.79769100 -1.78377900

F 5.12008600 0.05039700 -0.33776000

N -2.59932000 -1.53408500 -2.59210600

H -2.38860700 -1.22630800 -3.54210500

Si -2.12141600 -3.22777600 -2.35837000

H -0.78936600 -3.35583400 -3.01048100

H -3.08457100 -4.17644900 -2.99865900

C -3.90790200 -1.02782700 -2.14620000

H -4.01455000 -1.37407500 -1.10687500

C -1.98478800 -3.63560300 -0.52905600

C -0.72318100 -3.81350800 0.07205700

C -3.12482600 -3.72129800 0.29501900

C -0.59632600 -4.03253600 1.44623700

H 0.18213300 -3.76652100 -0.53778900

C -3.00374400 -3.92847600 1.67261400

H -4.12564300 -3.61959300 -0.13660400

C -1.73803500 -4.07489300 2.25113200

H 0.39602600 -4.15104400 1.88796200

H -3.89938900 -3.97658100 2.29746300

H -1.64206600 -4.22428400 3.32931200

C -3.97678400 0.49724500 -2.11734800

C -4.85929100 1.16060300 -1.19634200

C -3.23229000 1.26197100 -2.99604900

C -5.69265300 0.46408100 -0.27328200

C -4.90708900 2.59752300 -1.18644000

C -3.28397600 2.67658800 -2.98919700

H -2.55980200 0.78015500 -3.70802100

C -6.51868700 1.13701500 0.60299500

H -5.68047900 -0.62558200 -0.24603700

C -5.75974100 3.26166600 -0.26147400

C -4.09828800 3.33298500 -2.09586600

H -2.66602200 3.23989800 -3.69259100

C -6.55161700 2.55177600 0.61535000

H -7.14465900 0.57383300 1.29966400

H -5.77715000 4.35499000 -0.26080300

H -4.13792000 4.42521800 -2.07485600

H -7.20414900 3.07603200 1.31791300

C -5.08225300 -1.58880300 -2.97416900

H -6.04484200 -1.18969200 -2.62119400

H -4.96728500 -1.29809700 -4.03092500

H -5.12236100 -2.68810300 -2.92182700


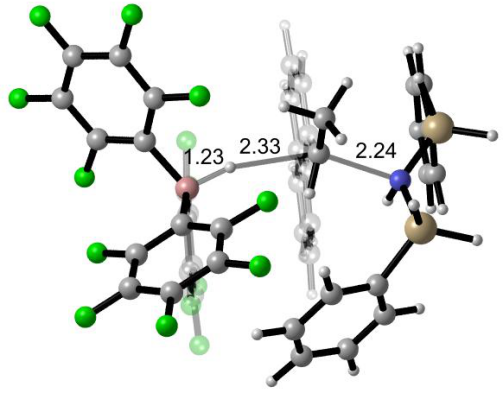


**TS6**

**G_gas_ = -3769.958568 a.u.**

**E_gas_ = -3770.46072361 a.u.**

Si -4.26431900 0.68806300 2.93516700

H -3.60399500 -0.19660700 3.93176400

C -5.25060000 -0.34411100 1.72443400

C -5.53445600 -1.69479800 1.99356800

C -5.69468100 0.20057900 0.50241600

C -6.24418200 -2.47580500 1.07601500

H -5.18459400 -2.15288100 2.92297500

C -6.39555600 -0.57848900 -0.41923900

H -5.48790500 1.24636500 0.25297000

C -6.67364500 -1.91904300 -0.13129600

H -6.45241000 -3.52484700 1.30083800

H -6.71960200 -0.14217900 -1.36682200

H -7.22018500 -2.53096000 -0.85334100

C -1.17502600 0.31199100 1.81347700

H -0.79138900 1.08986300 1.15854900

C -1.74531600 -0.85835200 1.19390100

C -2.27445400 -0.80181100 -0.15218200

C -1.81185600 -2.06194300 1.90600100

C -2.21507600 0.35552600 -0.96992700

C -2.91862600 -1.96860900 -0.68223300

C -2.36977700 -3.21848500 1.34220700

H -1.39313100 -2.11724600 2.91104300

C -2.79911700 0.37089600 -2.22328300

H -1.66766500 1.23914200 -0.64628200

C -3.51643700 -1.91753300 -1.97093500

C -2.93046600 -3.16457600 0.07978200

H -2.37165800 -4.15138000 1.90905700

C -3.46936000 -0.76710800 -2.72669800

H -2.70803200 1.26725600 -2.83921100

H -3.99744100 -2.81744100 -2.35968700

H -3.39456900 -4.05386900 -0.35358800

H -3.90879100 -0.74221400 -3.72553900

C -0.65398200 0.34065000 3.20365200

H -1.29968800 -0.18538700 3.91788700

H 0.32982000 -0.15680800 3.18646400

H -0.47856600 1.36914000 3.53825200

N -3.02347400 1.57091900 1.98242000

H -3.21631800 1.39149700 0.99161500

H -5.07531700 1.70731300 3.65953100

Si -2.63714900 3.33218000 2.12611200

H -3.91294400 4.06768200 2.35390000

H -1.72406200 3.50375900 3.28436800

C -1.83924900 3.76045100 0.49500000

C -2.62666500 3.82993300 -0.67277600

C -0.43725100 3.79918700 0.36179400

C -2.02908600 3.88171100 -1.93535900

H -3.71973200 3.82038600 -0.60323300

C 0.15958200 3.83692800 -0.90132000

H 0.20004200 3.76575200 1.24623100

C -0.63481900 3.85589200 -2.05203800

H -2.65217000 3.92422700 -2.83252000

H 1.24644400 3.81540500 -0.98817700

H -0.16478800 3.83070800 -3.03744100

B 1.81270600 -0.31799100 0.06019700

C 1.10209800 -0.48628100 -1.40087700

C 0.38762800 -1.65793800 -1.68437800

C 1.02716600 0.48618700 -2.40147000

C -0.30733800 -1.88246400 -2.87035300

C 0.30231100 0.32771800 -3.58665700

C -0.36815000 -0.86902300 -3.82676300

C 2.71852000 1.02906200 0.24034800

C 3.86569800 1.25021300 -0.52775300

C 2.43191700 2.02618700 1.16651200

C 4.65530500 2.39651300 -0.42281300

C 3.18092000 3.19731900 1.30674700

C 4.30456200 3.38535000 0.50280300

C 2.70027700 -1.60598000 0.53900700

C 2.88490200 -1.84315300 1.90276000

C 3.34722800 -2.50858100 -0.30981000

C 3.63575400 -2.90218900 2.41627600

C 4.11046700 -3.58483600 0.15414000

C 4.25759400 -3.78186700 1.52821200

F 1.35470300 1.92541300 1.98253400

F 2.81105700 4.13924200 2.18389300

F 5.03826300 4.49181500 0.61658100

F 5.73037900 2.56670100 -1.19296100

F 4.22307600 0.34352500 -1.44703600

F 2.32229100 -1.01671300 2.81496800

F 3.76681100 -3.07961400 3.73431900

F 4.98164000 -4.80229300 1.98919100

F 4.70030700 -4.42357100 -0.69986100

F 3.24740600 -2.38327500 -1.64099700

F 1.64147100 1.67789700 -2.26498000

F 0.20846500 1.33313600 -4.46638800

F -1.10799100 -1.02570600 -4.92717300

F -0.98389600 -3.01997000 -3.06445000

F 0.31893800 -2.63832900 -0.76558400

H 0.87317800 -0.24676300 0.85071200

**
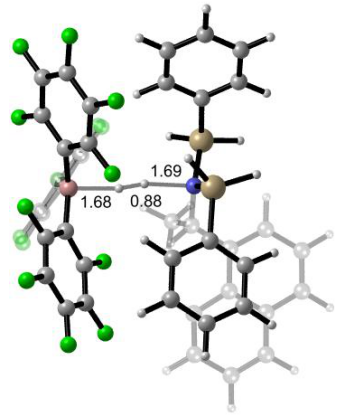
**

**TS7**

**G_gas_ = -3769.966044 a.u.**

**E_gas_ = -3770.46857304 a.u.**

H 0.27418500 0.60050100 0.34881600

B 1.61770100 -1.50628200 -0.14625900

C 0.53461200 -2.27357400 -1.02312200

C 0.77367500 -2.69503300 -2.33826400

C -0.76382500 -2.49622700 -0.54527200

C -0.21520400 -3.26837100 -3.14281300

C -1.77808100 -3.05959500 -1.31263000

C -1.49971000 -3.44143900 -2.62634500

C 1.78163900 -1.86517900 1.39358200

C 2.25286700 -0.90954600 2.30332900

C 1.41529000 -3.09711700 1.95069400

C 2.31542200 -1.11974200 3.67850800

C 1.48002800 -3.35886900 3.32181400

C 1.92812200 -2.36141000 4.19100900

C 2.77712400 -0.69805300 -0.87659800

C 2.48442700 0.20215700 -1.90905900

C 4.11788100 -0.75190300 -0.47726400

C 3.43386500 1.04530200 -2.47997000

C 5.10571700 0.06608200 -1.03134100

C 4.75455800 0.97379900 -2.03171800

F -1.09259400 -2.11105800 0.69840700

F -3.01203100 -3.19325900 -0.82892500

F -2.46779100 -3.92989400 -3.39353900

F 0.05206500 -3.63626700 -4.39160100

F 1.98351200 -2.55724800 -2.88771200

F 0.97828600 -4.09170000 1.17129900

F 1.12147600 -4.54483400 3.80447200

F 1.98841600 -2.59147600 5.49567300

F 2.73207200 -0.15779800 4.49712400

F 2.63805000 0.29763200 1.86255700

F 1.22574700 0.31684100 -2.35704000

F 4.50466900 -1.59718800 0.48413300

F 6.36492700 -0.00354200 -0.61022200

F 5.66752800 1.80247000 -2.52997500

F 3.09409500 1.93668000 -3.40530100

H 0.64268700 -0.15408100 0.07948100

N -0.75021500 1.86125300 0.83198500

Si -1.08469100 2.56770100 -0.81053400

H 0.19239700 2.54378100 -1.55975800

H -1.55777800 3.97070600 -0.65222400

C -1.80539500 0.93762000 1.36970300

H -1.86281700 0.12535800 0.63634700

C -1.40243500 0.28959900 2.69959400

H -1.34294600 1.02877300 3.51378300

H -2.16478400 -0.45038300 2.98276000

H -0.43824600 -0.23167600 2.61407600

C -3.20170700 1.54915500 1.46214200

C -4.35423000 0.73568400 1.17709100

C -3.38369800 2.87499400 1.81000300

C -4.26985400 -0.63554500 0.79875500

C -5.65974000 1.33576900 1.23255700

C -4.67247600 3.45949600 1.87694500

H -2.51795600 3.50078400 2.02482800

C -5.39687000 -1.36493200 0.48098100

H -3.30439000 -1.13328100 0.74169400

C -6.80292700 0.55311400 0.90873400

C -5.78777900 2.70743800 1.58957300

H -4.76991900 4.51257600 2.15188600

C -6.67886300 -0.76842000 0.53639000

H -5.28917000 -2.40771400 0.17512100

H -7.78842300 1.02493500 0.95521200

H -6.78597800 3.15186900 1.62868500

H -7.56523900 -1.35559000 0.28335400

C -2.41602200 1.57509000 -1.68204400

C -2.15702500 0.32686100 -2.28338800

C -3.73338700 2.07152700 -1.71964000

C -3.18680600 -0.40168700 -2.88781800

H -1.14365300 -0.08252600 -2.28237500

C -4.76225300 1.34512100 -2.32207600

H -3.96803200 3.02691500 -1.24268900

C -4.48989400 0.10413400 -2.90276900

H -2.98296300 -1.37223600 -3.34345100

H -5.78101700 1.73949000 -2.31990600

H -5.29449300 -0.47438200 -3.36345900

Si 0.32761600 2.81395100 1.91084900

H -0.45862400 3.79900600 2.70861300

H 1.03258600 1.89796200 2.83588700

C 1.51174600 3.77611100 0.81931800

C 2.63410200 3.17917200 0.20858500

C 1.25936600 5.13791300 0.56210000

C 3.46534200 3.91850300 -0.63683200

H 2.86006600 2.12578500 0.38847600

C 2.08914400 5.87893000 -0.28428900

H 0.39816100 5.62931500 1.02445600

C 3.19157800 5.26760500 -0.88673500

H 4.32708700 3.44736300 -1.11285200

H 1.87374800 6.93350700 -0.47437700

H 3.84179500 5.84134600 -1.55207700

**
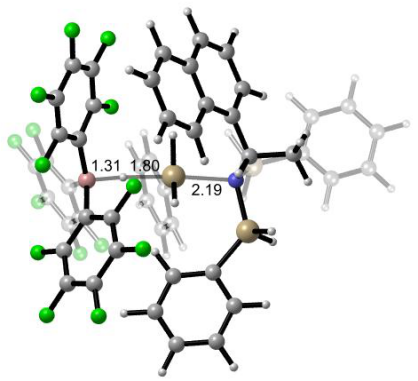
**

**TS8**

**G_gas_ = -4291.395867 a.u.**

**E_gas_ = -4291.99452278 a.u.**

Si 0.84127900 -0.23368000 -0.17637100

H -0.92634300 0.05834400 -0.31948800

C 0.76221800 -1.39802900 -1.64639500

C 0.83695300 -2.80351600 -1.62454300

C 0.55484300 -0.76439300 -2.88975500

C 0.70275300 -3.54786800 -2.79709200

H 0.98740700 -3.34030000 -0.68789700

C 0.42142200 -1.50901200 -4.06419300

H 0.47852600 0.32235300 -2.94229500

C 0.49080400 -2.90353700 -4.01966100

H 0.74413600 -4.63833200 -2.75220600

H 0.24730300 -0.99646800 -5.01331700

H 0.36398400 -3.48956200 -4.93313600

B -2.22553600 0.24751400 -0.27018100

C -2.63158000 -0.44066400 1.15066100

C -1.95082400 -0.07078900 2.31355100

C -3.59403700 -1.43799100 1.33646500

C -2.12835700 -0.66964100 3.55877400

C -3.80132900 -2.07988700 2.56197900

C -3.06407800 -1.69647500 3.68261000

C -2.32284700 1.87097600 -0.27457500

C -3.10691000 2.61324700 0.61341000

C -1.51270400 2.63251200 -1.12253100

C -3.05852900 4.00921100 0.69366200

C -1.43151400 4.02165400 -1.08026700

C -2.21300000 4.71538500 -0.15749400

C -2.67927100 -0.53687900 -1.61722900

C -3.19823800 0.07086400 -2.76541500

C -2.43486300 -1.91000900 -1.74381900

C -3.42686500 -0.61973400 -3.96193000

C -2.65649800 -2.64106400 -2.90658100

C -3.14920200 -1.98383700 -4.03458200

N 2.91823700 -0.54814900 0.45524000

C 3.29550800 0.69910200 1.32475800

H 2.65887900 0.58115900 2.20845600

C 4.75028100 0.70919900 1.82040600

H 5.45948800 0.97466200 1.02847500

H 4.83101100 1.47261600 2.60917900

H 5.06477600 -0.25305500 2.24664100

C 2.97169700 2.05027900 0.69215100

C 1.97494500 2.92568000 1.25424300

C 3.68072800 2.47192700 -0.41963600

C 1.21966200 2.62783600 2.42358100

C 1.70422900 4.17700600 0.59203200

C 3.39561000 3.68914400 -1.07921900

H 4.48724500 1.85524100 -0.81318100

C 0.26259500 3.49582500 2.90811400

H 1.35426400 1.67982000 2.94285000

C 0.71180200 5.04925800 1.11702200

C 2.41378600 4.51950400 -0.59077700

H 3.95968700 3.96144300 -1.97425400

C 0.00180500 4.71963700 2.25093000

H -0.31861800 3.21352600 3.78834000

H 0.49203200 5.97473500 0.58028700

H 2.16885400 5.45541300 -1.09794000

H -0.78462900 5.38312600 2.61827500

H 0.50395200 -0.55529800 1.21403800

H 1.06243000 1.15323600 -0.58212200

F -4.68621400 -3.07366400 2.66438400

F -3.24304700 -2.31219400 4.84901600

F -1.41257500 -0.27339100 4.61409000

F -1.04801400 0.92838900 2.26235600

F -4.36649800 -1.84738400 0.32202800

F -1.93536100 -2.59938700 -0.69772300

F -2.37980200 -3.94532900 -2.96407700

F -3.34559800 -2.65421300 -5.16847600

F -3.90788300 0.01619900 -5.03089400

F -3.50606000 1.37510400 -2.77907900

F -0.74284700 2.01574300 -2.04070300

F -0.58420300 4.68947200 -1.86866100

F -2.10033600 6.04040700 -0.04733800

F -3.78182500 4.66742800 1.60151500

F -3.94567500 2.00279900 1.46323800

Si 2.91173300 -2.03933500 1.57800800

H 3.45787700 -1.54624900 2.86497900

H 3.90145000 -2.97263800 0.97703200

C 1.41299500 -3.09007500 2.07313000

C 1.83574300 -4.03559600 3.03511900

C 0.06108100 -3.12116400 1.68857900

C 0.95071800 -4.96428700 3.58613500

H 2.87810200 -4.05255000 3.37449900

C -0.82728300 -4.05595800 2.23075500

H -0.34513200 -2.42428700 0.96281900

C -0.38859400 -4.97638300 3.18386100

H 1.30872900 -5.67755000 4.33275900

H -1.86834300 -4.05059900 1.90144900

H -1.08668300 -5.69863800 3.61400600

Si 3.87348200 -0.88063300 -1.09533500

H 3.39640400 0.06729700 -2.12654800

H 3.52732800 -2.27448600 -1.44209800

C 5.73859000 -0.71087200 -0.95237900

C 6.40747500 0.23140300 -1.75758500

C 6.51634500 -1.53105700 -0.11023500

C 7.79871800 0.36810900 -1.70884300

H 5.83800400 0.86983900 -2.43990100

C 7.90483300 -1.39668600 -0.05690300

H 6.03695300 -2.28573100 0.51833100

C 8.54884100 -0.44217300 -0.85285900

H 8.29610000 1.10807900 -2.34090000

H 8.48889200 -2.03986900 0.60627300

H 9.63571300 -0.33558800 -0.80908700

**
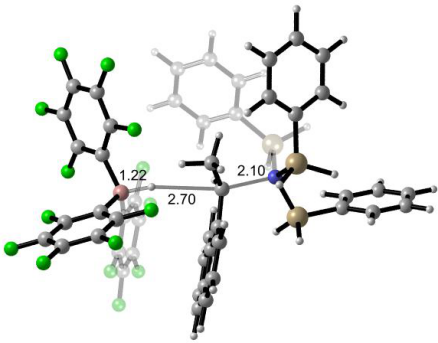
**

**TS9**

**G_gas_ = -4291.396941 a.u.**

**E_gas_ = -4291.99006722 a.u.**

Si -4.02171400 2.02998400 0.35104300

C -5.79455400 2.18560500 -0.24422500

C -6.87070900 2.31018900 0.65727300

C -6.07814800 2.22363100 -1.62485600

C -8.18292900 2.44824100 0.19769200

H -6.68519100 2.30861200 1.73483700

C -7.39000600 2.35574700 -2.08772100

H -5.26626700 2.15634400 -2.35450200

C -8.44476300 2.46459700 -1.17614400

H -9.00312300 2.54700800 0.91326100

H -7.58842200 2.38081200 -3.16213300

H -9.47110600 2.57114700 -1.53664400

N -3.46284100 0.26970300 0.41005100

C -1.39653300 0.22924400 0.75868900

H -1.18985300 0.14547200 -0.30491400

H -3.89814600 2.52501600 1.74571500

H -3.13807600 2.73862000 -0.59180000

Si -4.30580700 -0.52280100 1.83203300

H -3.51029700 -0.20026600 3.03979000

Si -3.63837700 -0.52810000 -1.24656100

H -3.31856100 0.58113200 -2.18537900

H -5.06051100 -0.93296000 -1.37542200

C -2.46803900 -1.94740400 -1.54017600

C -1.18844000 -1.70617800 -2.08436400

C -2.80730400 -3.27055100 -1.19076300

C -0.26213000 -2.74094200 -2.22929000

H -0.88331900 -0.70399900 -2.39677500

C -1.88138400 -4.30540100 -1.33895200

H -3.79473600 -3.49897900 -0.78703800

C -0.60378700 -4.04079200 -1.84298200

H 0.72981200 -2.51781800 -2.62334300

H -2.15575700 -5.32196400 -1.04670600

H 0.12930300 -4.84571100 -1.92250700

C -4.60979700 -2.35594300 1.63373700

C -3.75057800 -3.33877800 2.16214600

C -5.76683200 -2.77944900 0.94694400

C -4.02140900 -4.69761600 1.98546100

H -2.85422000 -3.05057100 2.71513400

C -6.03783300 -4.13825300 0.76557200

H -6.46582700 -2.04124300 0.54324700

C -5.16072300 -5.09893500 1.28020800

H -3.33841400 -5.44504100 2.39600800

H -6.93488100 -4.44795200 0.22386400

H -5.36890400 -6.16220400 1.13686400

H -5.62666000 0.14855400 1.92557900

C -0.98603000 1.49684300 1.33966900

C -0.55336400 2.59448600 0.49761000

C -0.92103300 1.63462900 2.72907100

C -0.59371400 2.57304000 -0.92227200

C -0.06377300 3.78741900 1.13032900

C -0.42713300 2.80023800 3.33467100

H -1.22865200 0.80763900 3.36736900

C -0.23068400 3.67625200 -1.66689900

H -0.92686500 1.68091100 -1.45076300

C 0.34498500 4.89470400 0.33314000

C -0.00082300 3.85226300 2.54689800

H -0.36633900 2.86137500 4.42281700

C 0.24771000 4.85037800 -1.03858700

H -0.28122800 3.63101700 -2.75642100

H 0.74210500 5.78151300 0.83256100

H 0.39395600 4.76033000 3.01037200

H 0.57811900 5.69409100 -1.64733100

C -1.19711300 -1.06343500 1.46742000

H -1.66809400 -1.89775400 0.93777900

H -0.11072800 -1.23918700 1.43489100

H -1.48772000 -1.05209300 2.52331000

B 2.40339600 -0.15917800 -0.00228200

C 2.51619900 1.10305900 -1.03872400

C 2.03663600 0.99310200 -2.34816600

C 2.97707900 2.38017500 -0.70366700

C 2.05705600 2.02443800 -3.28380900

C 3.02111600 3.44688200 -1.60545400

C 2.56115300 3.26878900 -2.90701400

C 3.24441000 0.02526000 1.38993100

C 4.63668500 0.15437800 1.38225900

C 2.66279100 0.02410600 2.65529800

C 5.40810800 0.29838400 2.53615100

C 3.38773300 0.16684800 3.84254200

C 4.77394100 0.30203600 3.78181600

C 2.83622300 -1.60272300 -0.65822500

C 2.28040300 -2.78135700 -0.15464900

C 3.73625600 -1.78591100 -1.71353700

C 2.54925800 -4.05046100 -0.66829100

C 4.03548600 -3.03689200 -2.26481800

C 3.43013300 -4.18023800 -1.74117200

F 1.32549600 -0.11158200 2.80555200

F 2.76592300 0.17191400 5.02798400

F 5.48773400 0.43774400 4.90067300

F 6.73437400 0.43150100 2.46879600

F 5.28782800 0.17133200 0.20922800

F 1.39934900 -2.73885600 0.86711000

F 1.93071200 -5.13569700 -0.18185100

F 3.68189700 -5.38239900 -2.26282800

F 4.88825100 -3.14922600 -3.28508000

F 4.34604300 -0.73682600 -2.28160200

F 3.37511500 2.66792200 0.54761200

F 3.41431500 4.66066000 -1.20716100

F 2.50174200 4.30498600 -3.74928700

F 1.52533100 1.86492100 -4.50135100

F 1.47560400 -0.16574000 -2.76661500

H 1.21386700 -0.23919200 0.26888300

**
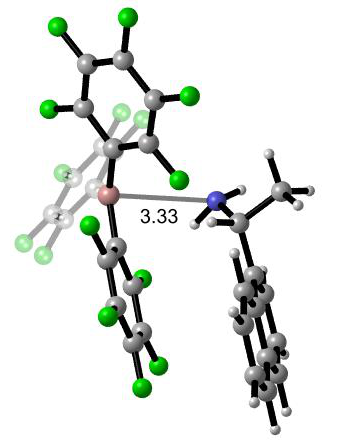
**

**TS10**

**G_gas_ = -2725.889798 a.u.**

**E_gas_ = -2726.19451894 a.u.**

C -1.15293200 -0.10739100 -2.25249300

H -0.97297500 -0.69876900 -1.34854300

N -0.01613900 0.80623800 -2.36255700

H 0.09003900 1.16944300 -3.30894400

H -0.12140900 1.60427500 -1.74041000

C 1.91329200 -1.40601300 0.28726200

C 1.25823100 -2.52391100 -0.25557600

C 3.29537700 -1.56415500 0.49423100

C 1.91513800 -3.70237600 -0.60429300

C 3.98705800 -2.73380400 0.17807300

C 3.29076300 -3.80806600 -0.38089400

C -0.27636100 -0.01922900 1.19254200

C -1.15955100 1.05585000 0.97231000

C -0.80994700 -1.07906500 1.95217600

C -2.47432600 1.07880100 1.42920500

C -2.11960700 -1.09157400 2.42840700

C -2.95644200 -0.00571200 2.16379500

C 1.97436700 1.29921100 0.39693200

C 1.87717100 2.37803500 1.28942100

C 2.81381600 1.49324400 -0.71361900

C 2.54780600 3.58554100 1.09843700

C 3.48313900 2.69440900 -0.94877000

C 3.35319200 3.74260500 -0.03294800

F 1.24827700 -4.72241700 -1.13456800

F -0.06160600 -2.50047300 -0.48014600

F 3.93025700 -4.92421400 -0.69440000

F 5.29258800 -2.83493600 0.40305600

F 4.01536200 -0.57701900 1.03416400

F 2.99686100 0.52137700 -1.60479100

F 4.25234400 2.84715800 -2.02288900

F 3.99512800 4.88374200 -0.23652400

F 2.43205300 4.57889200 1.97444600

F 1.12290100 2.27345100 2.38818400

F -0.77502500 2.12013100 0.25555800

F -3.27431400 2.09921400 1.14831400

F -4.20778900 -0.01546800 2.59011500

F -2.58648800 -2.13184500 3.11145300

F -0.05596500 -2.13694300 2.26166800

B 1.18870300 -0.04540600 0.61967400

C -2.52541800 0.54094100 -2.06892300

C -3.56368200 -0.16228400 -1.36281800

C -2.78135200 1.81435200 -2.54052200

C -3.38938900 -1.47159300 -0.82237800

C -4.82657700 0.49031300 -1.14548200

C -4.03577900 2.44421800 -2.35147600

H -1.99023800 2.35946100 -3.06067500

C -4.38878100 -2.08816400 -0.09651000

H -2.44564300 -1.99785700 -0.96119900

C -5.83726400 -0.17742000 -0.39875100

C -5.03664400 1.79871700 -1.66298700

H -4.19638100 3.45183200 -2.74306900

C -5.62701300 -1.43691800 0.11951900

H -4.21833400 -3.08275000 0.32315400

H -6.78857500 0.33564300 -0.23370400

H -6.00107000 2.28474100 -1.49377300

H -6.40756700 -1.93067600 0.70308800

C -1.13392400 -1.08247300 -3.43527900

H -0.16020900 -1.59374900 -3.48717500

H -1.29940000 -0.54200600 -4.38260500

H -1.92619500 -1.83860000 -3.34091700

**
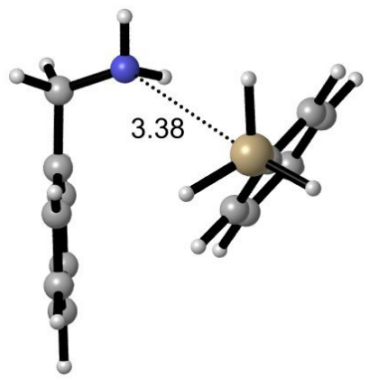
**

**int1b**

**G_gas_ = -849.195610 a.u.**

**E_gas_ = -849.412674146 a.u.**

Si 1.69213100 2.05424400 1.11366000

H 2.53701800 2.28392600 2.32966300

C 1.87944100 0.26377300 0.54813600

C 3.00249500 -0.14129200 -0.19999200

C 0.90100200 -0.70267900 0.84768400

C 3.14560900 -1.46366700 -0.63164300

H 3.77934800 0.58583300 -0.45837400

C 1.04129800 -2.02644100 0.41892900

H 0.00053000 -0.42057900 1.39877600

C 2.16331900 -2.41048400 -0.32108900

H 4.02438700 -1.75583700 -1.21311600

H 0.26017100 -2.75332900 0.65436800

H 2.27127700 -3.44432000 -0.66017000

N 0.01207000 1.57336300 -1.77603800

H 0.62403900 0.76455200 -1.66622700

H 0.39358500 2.13725200 -2.53547500

C -1.35398600 1.14547700 -2.07716700

C -1.95100700 0.40192200 -0.90104300

C -2.23957100 1.08680800 0.29034900

C -2.17008700 -0.98003200 -0.95041800

C -2.73696900 0.40645100 1.40281200

C -2.66760500 -1.66827100 0.16222900

H -1.93786000 -1.52828200 -1.86806600

C -2.95008600 -0.97693700 1.34289000

H -2.83005000 -2.74795900 0.10647500

H -3.33692900 -1.51140800 2.21438600

H 2.18128000 2.98930000 0.06120800

H 0.28108200 2.34397800 1.47910400

H -2.05275100 2.16203900 0.34294000

H -2.95559400 0.95414100 2.32328100

H -1.95564400 2.04986300 -2.27423100

H -1.44134400 0.51011100 -2.98358000

**
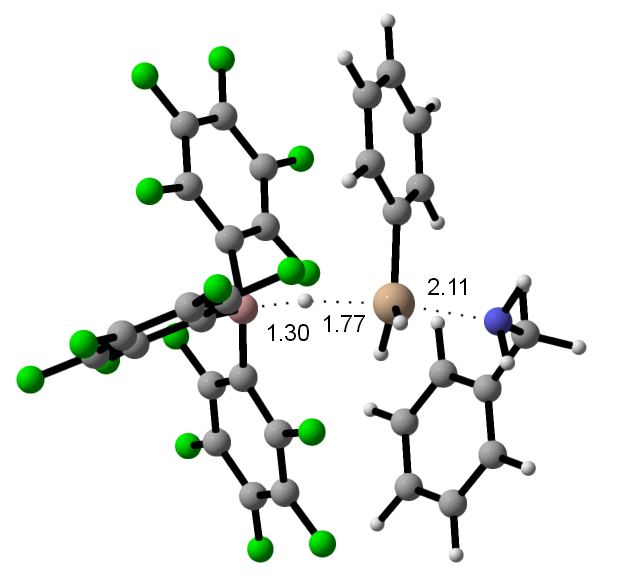
**

**Int2b**

**G_gas_ = -3055.75893 a.u.**

**E_gas_ = -3056.10256642 a.u.**

Si 0.98544700 -0.73574800 -1.98038400

H -0.28469300 -0.03013200 -0.97805000

C 0.31671000 -2.46537400 -1.71476300

C 1.08011000 -3.48465400 -1.11398300

C -1.02969900 -2.73618000 -2.02774100

C 0.52321300 -4.73629000 -0.84034800

H 2.10663300 -3.29221200 -0.79600200

C -1.58936400 -3.98668600 -1.75198300

H -1.65715600 -1.95286400 -2.45956200

C -0.81475600 -4.98800900 -1.15756700

H 1.12475200 -5.50380400 -0.34830700

H -2.64011100 -4.17387700 -1.98667800

H -1.25881500 -5.95912400 -0.92522800

B -0.77906300 0.45954100 0.12379300

C 0.42357400 1.35528500 0.75429600

C 1.17955300 2.18828600 -0.07514000

C 0.80317300 1.35217300 2.09939300

C 2.27791800 2.92845600 0.36151100

C 1.89412200 2.07956300 2.58501500

C 2.63494900 2.87190200 1.70766400

C -2.00402000 1.37988300 -0.40106000

C -2.57824500 2.33908900 0.43695500

C -2.56559400 1.27802500 -1.67249000

C -3.63471500 3.16340100 0.04493400

C -3.62092000 2.08044100 -2.11229400

C -4.15710500 3.03320300 -1.24444300

C -1.18922200 -0.88782200 0.93102700

C -2.50057500 -1.32293700 1.14833800

C -0.19901600 -1.78702500 1.33991500

C -2.81125600 -2.56714600 1.71077800

C -0.45799200 -3.03779100 1.89102500

C -1.78313600 -3.43480200 2.07569000

N 2.59790100 -1.30570300 -3.20891700

H 2.77463400 -0.46787200 -3.77205900

H 2.25628500 -2.01539700 -3.86506800

C 3.89648500 -1.75842500 -2.60201400

C 4.40483600 -0.76431700 -1.58512400

C 4.32107300 -1.03018400 -0.21101600

C 4.67066100 -0.05122100 0.72225800

H 3.93916900 -1.99281800 0.13814400

C 5.10649100 1.20498700 0.29132800

H 4.57379700 -0.26119800 1.78984100

H 5.34001300 1.98415100 1.01956000

H 1.94328500 -0.13233900 -1.05440500

H 0.41778300 0.01607400 -3.11947500

F 2.23867400 2.01849600 3.87146900

F 3.70863600 3.53588500 2.14302900

F 3.00650800 3.65268600 -0.49245400

F 0.88799600 2.26196400 -1.39055400

F 0.13264200 0.61758300 2.99405800

F 1.10232200 -1.47768500 1.14975900

F 0.53971800 -3.87068800 2.19799400

F -2.05940100 -4.63619600 2.57866300

F -4.08095600 -2.93632100 1.88150100

F -3.54905600 -0.56412200 0.79972700

F -2.10494300 0.36176000 -2.55188200

F -4.11851200 1.94462900 -3.34271100

F -5.16347500 3.80913100 -1.64111500

F -4.15046200 4.06348500 0.88122200

F -2.12793800 2.47761100 1.69311800

C 5.21151400 1.47340500 -1.07741200

H 5.54389800 2.45638700 -1.41690400

C 4.87067200 0.49003000 -2.00846800

H 4.95474700 0.70987300 -3.07819500

H 4.63135400 -1.90459000 -3.41149300

H 3.71575000 -2.73712600 -2.13939800

**
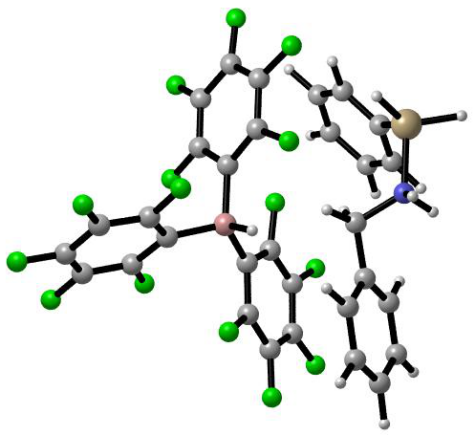
**

**Int3b**

**G_gas_ = -3055.749619 a.u.**

**E_gas_ = -3056.09247786 a.u.**

C -2.01735700 0.77299100 1.59780100

H -1.13050900 0.24979300 1.96797500

C -1.86214500 2.26537800 1.65012700

C -0.84807300 2.85167000 2.42146700

C -2.71216400 3.08513200 0.89007200

C -0.68537200 4.23849800 2.42813100

C -2.55585200 4.47288900 0.90523900

H -3.46365000 2.63229500 0.23484600

C -1.53900200 5.04902500 1.67301000

H -3.19765200 5.10031500 0.28321200

H -1.39376100 6.13145100 1.66014500

N -3.20444700 0.31756800 2.44723900

H -2.96121400 0.43759200 3.43880400

Si -3.89718200 -1.43746600 2.14428400

H -2.94054900 -2.34460000 2.80563700

H -5.17922300 -1.30786600 2.88564200

C -3.98123200 -1.60480900 0.30642700

C -3.42893600 -2.74609000 -0.30500200

C -4.45513800 -0.55717800 -0.51258400

C -3.31420400 -2.82161300 -1.69559700

H -3.06447200 -3.57505500 0.30297300

C -4.33130300 -0.62931900 -1.89862700

H -4.89914100 0.34289300 -0.07268000

C -3.75196400 -1.75931700 -2.48867500

H -2.84671400 -3.69916600 -2.14816400

H -4.65722800 0.20684300 -2.51959100

H -3.62778500 -1.80074400 -3.57313100

B 1.15915400 -0.01502800 0.42670600

C 0.43491200 1.13935500 -0.48862300

C -0.65952700 0.97805800 -1.33950600

C 0.83251700 2.47233400 -0.31796400

C -1.32422800 2.03459100 -1.97012300

C 0.20950100 3.55942000 -0.92857400

C -0.88698800 3.33910700 -1.76293900

C 2.78524800 -0.07133700 0.27527600

C 3.53065300 0.41886100 -0.79900600

C 3.52951600 -0.69260100 1.28338200

C 4.92463600 0.32752200 -0.86931500

C 4.92096500 -0.80975300 1.25630400

C 5.62429900 -0.29413300 0.16565200

C 0.58005800 -1.52919700 0.18565100

C -0.09218900 -2.26861800 1.15509700

C 0.75110700 -2.18034700 -1.04101900

C -0.58314600 -3.55758100 0.93669100

C 0.25811500 -3.45642200 -1.31579500

C -0.42206000 -4.14869200 -0.31250100

F 2.90001500 -1.22046900 2.34836500

F 5.58193500 -1.41030300 2.25055800

F 6.95368200 -0.39476900 0.11465800

F 5.59113700 0.81998500 -1.91685100

F 2.92342800 1.01898100 -1.83589100

F -0.36908000 -1.74401500 2.37553500

F -1.31997100 -4.18210000 1.87343800

F -0.99746600 -5.32674400 -0.57415800

F 0.37628000 -4.00340300 -2.52724800

F 1.36838100 -1.54136100 -2.04163100

F 1.83415900 2.76128800 0.52553900

F 0.60073100 4.81001600 -0.66995800

F -1.54950400 4.37015300 -2.29295700

F -2.42361500 1.80709200 -2.70682500

F -1.22044600 -0.23879600 -1.53251500

H 0.92705400 0.29885100 1.58984700

H -3.96630600 0.99113900 2.29276700

H -0.14836700 2.21383300 2.96698700

H 0.12968200 4.68507700 3.00147900

H -2.21513100 0.41709500 0.58169500

**
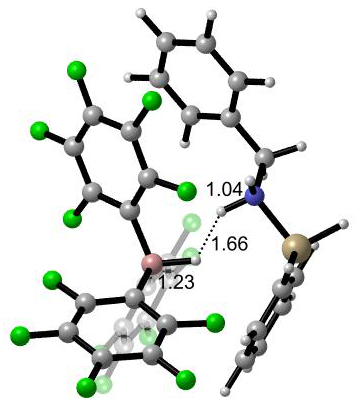
**

**Int4b**

**G_gas_ = -3055.775260 a.u.**

**E_gas_ = -3056.12112389 a.u.**

H 1.02350900 -0.84147300 1.35911600

B -0.53802200 0.55021700 0.02161700

C -1.18731700 -0.62597900 -0.90978700

C -2.41037000 -0.58165900 -1.58481600

C -0.53085000 -1.85255400 -0.99667000

C -2.94740400 -1.67922500 -2.26985700

C -1.00930100 -2.97082100 -1.66926500

C -2.24465900 -2.88332100 -2.31067500

C 0.94075300 1.05473700 -0.47835000

C 1.84925000 1.59058900 0.43679900

C 1.39714000 1.01336300 -1.79988900

C 3.12185400 2.05376000 0.10396100

C 2.66317100 1.46361800 -2.18534100

C 3.52693800 1.99583100 -1.22804400

C -1.49498300 1.83303400 0.31589200

C -2.15412200 2.01243300 1.53128800

C -1.67923600 2.85397400 -0.62048300

C -2.95126800 3.12395000 1.82034900

C -2.46404800 3.98308300 -0.38204900

C -3.10290900 4.11893300 0.85392200

F 0.66173400 -2.01696600 -0.35432700

F -0.33376500 -4.12379400 -1.65869900

F -2.76281000 -3.95036400 -2.91938500

F -4.13458200 -1.58954300 -2.86993500

F -3.15718200 0.53031100 -1.58788900

F 0.62122200 0.51599600 -2.77015800

F 3.07017300 1.36216000 -3.45147200

F 4.75134700 2.39302500 -1.57478100

F 3.95760000 2.50942000 1.04037300

F 1.52400800 1.64984200 1.75747400

F -2.05345400 1.08537700 2.51346800

F -1.10033000 2.75666200 -1.82660700

F -2.61266800 4.92791200 -1.31084100

F -3.85606800 5.18850100 1.10506700

F -3.56036300 3.24366900 3.00248500

H -0.37991300 0.01349900 1.11343500

N 1.49712700 -1.15126400 2.23179500

H 1.90496800 -0.27742700 2.59018900

Si 0.07857600 -1.70901300 3.35021900

H -0.47020000 -0.44407200 3.87597500

H 0.82101000 -2.49263300 4.37190300

C 2.58633800 -2.14044800 1.91073000

H 2.11011100 -2.96239400 1.36105100

C -1.09231500 -2.71715300 2.33203800

C -2.31043700 -2.15706900 1.89543800

C -0.77054400 -4.03484100 1.94601800

C -3.18100000 -2.90001400 1.09521700

H -2.56803300 -1.13081000 2.16291900

C -1.63413400 -4.76706000 1.13021900

H 0.16419400 -4.49769000 2.27793400

C -2.84046100 -4.19856500 0.70590400

H -4.11657700 -2.45234800 0.75280700

H -1.36386100 -5.77713900 0.81528400

H -3.50753200 -4.76455200 0.05109700

C 3.69338200 -1.50346900 1.10926900

C 3.72375400 -1.60683600 -0.28961900

C 4.68671700 -0.75923300 1.76519000

C 4.73876100 -0.98007800 -1.01628000

C 5.69677300 -0.12816100 1.03652500

H 4.67306600 -0.67314200 2.85654200

C 5.72615300 -0.24225400 -0.35631100

H 6.45760700 0.45867200 1.55543100

H 6.50445600 0.26394000 -0.93042100

H 2.94179300 -2.16287100 -0.80882700

H 4.74423900 -1.04682700 -2.10671200

H 2.96380700 -2.53004800 2.86769100

**
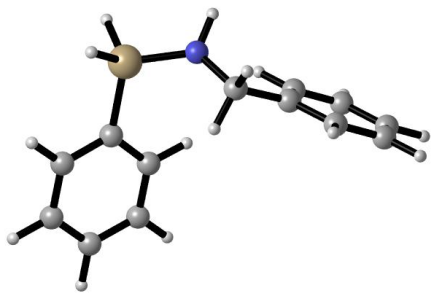
**

**Int5b**

**G_gas_ = -848.036123 a.u.**

**E_gas_ = -848.237349058 a.u.**

N 0.21196400 1.91623600 -0.25274000

H 0.78389900 2.54985300 -0.80362100

Si -1.51529200 2.02698900 -0.40339200

H -1.75613200 2.58526100 -1.76761800

H -2.19820800 2.93429600 0.57738500

C 0.93014700 1.27635200 0.83131300

H 0.22155000 0.62443100 1.37371400

C -2.26140700 0.31675800 -0.15315900

C -1.56356000 -0.84393200 -0.54039600

C -3.53785700 0.16342500 0.41997300

C -2.12458300 -2.11239800 -0.36672100

H -0.56287900 -0.75698800 -0.97218900

C -4.10643500 -1.10292600 0.58811000

H -4.09742700 1.04579900 0.74710500

C -3.39919600 -2.24354700 0.19455500

H -1.56443900 -3.00157600 -0.66832200

H -5.10013200 -1.20115800 1.03352300

H -3.83920100 -3.23520500 0.33037600

C 2.10917200 0.41941300 0.39147300

C 2.26973200 0.00683900 -0.93766100

C 3.05282900 0.00004100 1.34312400

C 3.34373500 -0.81098400 -1.30761700

C 4.12288500 -0.81915400 0.97850900

H 2.94584200 0.32144500 2.38413200

C 4.27253000 -1.22904300 -0.35173400

H 4.84694600 -1.13635900 1.73381100

H 5.11177400 -1.86731600 -0.63978600

H 1.54487300 0.33700200 -1.68454300

H 3.45347400 -1.12164800 -2.35020700

H 1.29244900 2.00560700 1.58429800

**
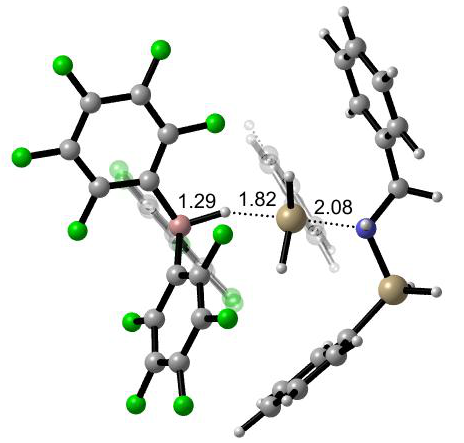
**

**Int6b**

**G_gas_ = -3577.217286 a.u.**

**E_gas_ = -3577.65078493 a.u.**

Si 1.26154300 -1.16810600 -0.36957500

H -0.34546700 -0.32753700 -0.22372900

C 1.07332900 -1.78227700 1.39267700

C 2.04549300 -1.53956600 2.37899100

C -0.12256500 -2.41714300 1.78120700

C 1.83818300 -1.91519600 3.70905200

H 2.96971900 -1.01709000 2.12820100

C -0.33395800 -2.79371900 3.10974100

H -0.90633200 -2.59620800 1.04282000

C 0.64421900 -2.54081100 4.07734500

H 2.59799200 -1.69418100 4.46198800

H -1.27606700 -3.26839800 3.39482200

H 0.46830000 -2.81546500 5.12026100

B -1.16773200 0.64470400 -0.03906300

C -0.46816600 1.90959100 -0.78770700

C 0.09601500 1.73246700 -2.05421700

C -0.33790000 3.19336000 -0.25160500

C 0.79514500 2.72665500 -2.73832600

C 0.35156900 4.22075900 -0.90150900

C 0.91875500 3.98413300 -2.15235200

C -2.51688400 0.17487200 -0.80695000

C -3.52376800 1.10066200 -1.09290500

C -2.76795300 -1.13134600 -1.22279000

C -4.70429500 0.76853000 -1.75965400

C -3.93176700 -1.51355500 -1.89480400

C -4.90633100 -0.55272800 -2.16739800

C -1.25550500 0.71435700 1.58219400

C -2.37067200 0.35670400 2.34574500

C -0.10484100 0.99388300 2.32616500

C -2.34349500 0.25811300 3.74253900

C -0.02428600 0.89743400 3.71123500

C -1.15937800 0.51950100 4.42963900

H 1.79620200 0.16823000 -0.65240700

H 0.61274900 -1.94518000 -1.43695200

F 0.52368100 5.40971000 -0.31880200

F 1.65919700 4.92733200 -2.74150000

F 1.39354100 2.47454600 -3.90645300

F 0.02536600 0.52648300 -2.65410300

F -0.85181300 3.48753000 0.94837300

F 1.03864700 1.32210400 1.68664700

F 1.13127300 1.11458600 4.34608200

F -1.10275300 0.38868900 5.75410500

F -3.43337600 -0.10923600 4.41826700

F -3.53979000 0.05191800 1.76389700

F -1.87557600 -2.11551300 -0.97542800

F -4.11922600 -2.77984700 -2.27389200

F -6.02382100 -0.89384800 -2.80647900

F -5.63644600 1.68837600 -2.00752300

F -3.38357000 2.37450800 -0.69543000

N 3.12222800 -1.99770200 -0.79555800

H 3.06324400 -1.92503700 -1.81913200

Si 4.66106100 -1.06272400 -0.34085900

H 5.65124600 -1.51578100 -1.35630900

H 5.01390300 -1.56413200 1.00866800

C 3.25340800 -3.46851600 -0.47973500

C 4.35619600 0.76674900 -0.39026400

C 4.32197900 1.45127100 -1.62320900

C 4.02193600 1.47292700 0.78174100

C 3.94338500 2.79310400 -1.68258900

H 4.57194000 0.93123100 -2.55355900

C 3.64600900 2.81651100 0.72301800

H 4.02113100 0.97169400 1.75302200

C 3.60214300 3.47487500 -0.50831800

H 3.87771600 3.30350900 -2.64579300

H 3.35798500 3.33957100 1.63726700

H 3.27489700 4.51564200 -0.56212200

C 2.22099200 -4.29664100 -1.20618700

C 1.10517000 -4.81434900 -0.53454000

C 2.33878200 -4.49907600 -2.59033100

C 0.11120000 -5.49949900 -1.23840000

C 1.34609100 -5.18268100 -3.29547400

H 3.21630100 -4.11886200 -3.12450700

C 0.22599000 -5.67897600 -2.61967200

H 1.44627300 -5.33038200 -4.37348700

H -0.55622500 -6.20661900 -3.17053800

H 1.00162000 -4.65793400 0.54114200

H -0.76172000 -5.88482500 -0.70638200

H 3.14888100 -3.57444700 0.60872500

H 4.26895100 -3.80407500 -0.75565300

**
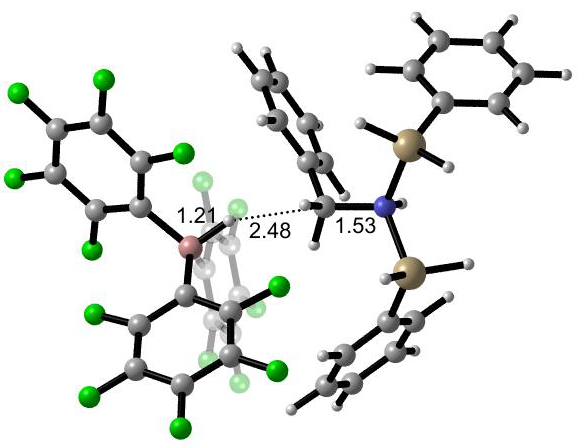
**

**Int7b**

**G_gas_ = -3577.207717 a.u.**

**E_gas_ = -3577.63907164 a.u.**

Si -3.98219600 0.14036700 -2.51831500

H -2.96391900 1.07405100 -3.03972300

C -5.46385200 0.90101800 -1.69913500

C -5.35009800 2.06472000 -0.90932300

C -6.72751200 0.28823200 -1.83044600

C -6.47139500 2.59462900 -0.26837700

H -4.38395000 2.55859000 -0.78497300

C -7.84706600 0.82122900 -1.18640800

C -7.71814900 1.97415500 -0.40503100

H -6.36822300 3.49461700 0.34229100

H -8.82130300 0.33890100 -1.29713400

H -8.59377800 2.39196300 0.09831000

C -1.76743900 -0.17956200 -0.73670200

H -1.25332700 -0.87841900 -0.06392300

C -2.04156900 1.12330900 -0.04015900

C -2.66978000 1.12008400 1.21478300

C -1.64147600 2.34090000 -0.60764800

C -2.91627300 2.31731800 1.88623600

C -1.89482900 3.54121600 0.06198300

H -1.08214400 2.34510500 -1.54549400

C -2.53591300 3.53143100 1.30413800

H -1.56421200 4.48482900 -0.37810700

H -2.71488700 4.46997700 1.83428000

N -3.06012400 -0.85717600 -1.20575800

H -3.69336100 -0.85911700 -0.39438000

H -4.37900600 -0.87866700 -3.52177000

Si -2.79913900 -2.68766600 -1.60584100

H -4.18480500 -3.11343000 -1.92981700

H -1.89832700 -2.68660700 -2.77624200

C -2.14177000 -3.43869400 -0.05012200

C -3.00856100 -3.59818700 1.05243000

C -0.77267200 -3.73370500 0.10879100

C -2.51322600 -4.01576500 2.28917900

H -4.08046500 -3.39400800 0.95119700

C -0.28221700 -4.15369900 1.34663400

H -0.08318200 -3.60459700 -0.72641700

C -1.14802100 -4.28512100 2.43666300

H -3.18934200 -4.12582100 3.14054400

H 0.78607600 -4.33145900 1.47259800

H -0.74681300 -4.57344800 3.41007800

B 1.69114500 0.30573500 -0.20394900

C 1.29910900 -0.13686600 1.32798500

C 0.55419800 0.74682300 2.12096900

C 1.52782400 -1.38309400 1.91712800

C 0.01910100 0.41967100 3.36736100

C 1.03999600 -1.74486100 3.17755100

C 0.25991400 -0.84737900 3.90270200

C 2.47537600 -0.85652600 -1.04616600

C 3.85781600 -1.05961000 -0.99500400

C 1.78689400 -1.77127300 -1.83927300

C 4.51226400 -2.09253100 -1.67307800

C 2.38461200 -2.83313600 -2.51794800

C 3.76720900 -2.99496500 -2.43663800

C 2.42275500 1.75975100 -0.29680300

C 2.11678600 2.62694400 -1.34799600

C 3.31760400 2.26360300 0.64943500

C 2.65000900 3.91231600 -1.47057600

C 3.88157500 3.53982800 0.56882900

C 3.54514700 4.37014200 -0.50242800

F 0.43181800 -1.70152900 -1.96649900

F 1.63821100 -3.70274900 -3.21348700

F 4.36901800 -3.99829600 -3.07575600

F 5.83466400 -2.24131200 -1.58470300

F 4.62424400 -0.26245900 -0.23955500

F 1.24623500 2.25150500 -2.31276900

F 2.31120400 4.70490100 -2.49387900

F 4.07101200 5.59297700 -0.59769400

F 4.73148400 3.97667400 1.50150300

F 3.66093300 1.52099500 1.71208800

F 2.23053700 -2.33864400 1.27554900

F 1.28412400 -2.96430600 3.67928300

F -0.24830200 -1.19392000 5.08768100

F -0.73467300 1.29610200 4.04185000

F 0.29855100 1.98813200 1.67688600

H 0.62325400 0.46495700 -0.75712500

H -6.84418800 -0.61003100 -2.44457000

H -3.37913800 2.30192800 2.87495100

H -2.93426200 0.16975600 1.69095700

H -1.12662700 -0.05205600 -1.61300100

**
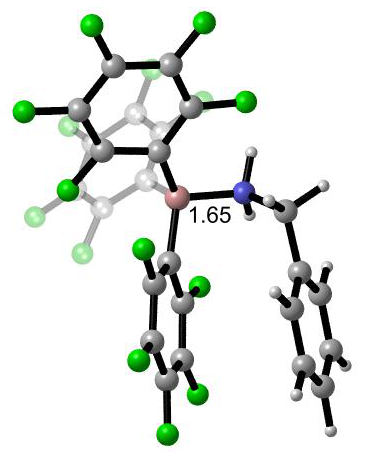
**

**int11b**

**G_gas_ = -2533.164928 a.u.**

**E_gas_ = -2533.40350446 a.u.**

C 1.17587600 -1.03100200 2.41678000

N 0.22421100 0.03555300 1.94264800

H -0.60845400 0.02578400 2.53582000

H 0.65573300 0.95583900 2.07120200

C -1.33411800 -1.24296700 0.15448900

C -1.88523600 -2.07173700 1.13153200

C -1.85185700 -1.42972600 -1.13396200

C -2.85031900 -3.04899800 0.86727500

C -2.81022700 -2.39046000 -1.45173900

C -3.31667400 -3.20844300 -0.43684900

C 1.08409900 -0.03582900 -0.49331500

C 1.98560700 1.02604800 -0.36726500

C 1.53796900 -1.09935700 -1.27553800

C 3.24238900 1.06498200 -0.96067000

C 2.78393100 -1.10041600 -1.91237300

C 3.64352800 -0.01634800 -1.74716100

C -1.19083800 1.37446200 0.18198100

C -1.11826200 2.22104100 -0.93136700

C -2.18588500 1.70931400 1.10656400

C -1.95457200 3.32956200 -1.10252700

C -3.04120200 2.80430200 0.98363300

C -2.92152900 3.62438700 -0.13993600

F -3.32777500 -3.81412200 1.84699400

F -1.50195700 -1.96445300 2.42747800

F -4.23407800 -4.12876500 -0.71371100

F -3.25332100 -2.52945300 -2.69813100

F -1.40944500 -0.65465400 -2.13355100

F -2.37008000 0.93111800 2.20438900

F -3.96058500 3.06474300 1.91019300

F -3.72003700 4.67495700 -0.29021200

F -1.83745800 4.10442100 -2.17715300

F -0.22991400 2.00489400 -1.90566300

F 1.66271500 2.06905000 0.43169000

F 4.07482200 2.08146500 -0.74684200

F 4.85558800 -0.03187400 -2.29231400

F 3.17978100 -2.15624600 -2.62178600

F 0.81769000 -2.22291300 -1.40805400

B -0.29722200 0.00311700 0.37963200

C 2.62727000 -0.68765100 2.17728400

C 3.44163700 -1.53339200 1.41272400

C 3.18212900 0.48528000 2.71276800

C 4.77468700 -1.19889900 1.16045700

C 4.50953000 0.82901800 2.45064800

H 2.57237700 1.15052200 3.33285800

C 5.30818000 -0.01132700 1.66843600

H 4.92104000 1.75659800 2.85494700

H 6.34295000 0.26252900 1.45079700

H 3.01928700 -2.44495000 0.98253400

H 5.38981800 -1.85566700 0.54087200

H 0.90363300 -1.96405200 1.91097200

H 0.99008700 -1.17561900 3.49213200

**
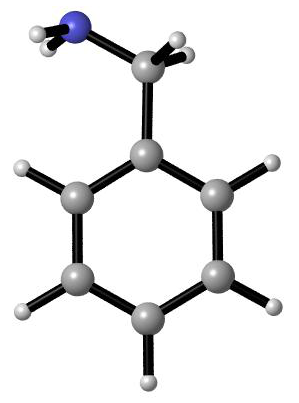
**

**1b**

**G_gas_ = -326.571905 a.u.**

**E_gas_ = -326.686360145 a.u.**

C 1.90770400 -0.66300800 -0.00022800

C 0.42772000 -0.28358600 -0.00005200

C -0.54404600 -1.29878800 -0.00000200

C -0.00301200 1.04816100 -0.00008600

C -1.90505800 -0.99116300 0.00011600

C -1.36768400 1.36295900 -0.00007500

H 0.73598300 1.85287200 -0.00011400

C -2.32352000 0.34549600 0.00001800

H -1.68198400 2.41023400 -0.00005700

H -3.38904700 0.58898200 0.00007400

N 2.89653000 0.39898300 0.00028100

H 2.79327800 0.99859000 -0.81795000

H 2.79259500 0.99840400 0.81855900

H -2.64524800 -1.79591500 0.00026000

H -0.22467300 -2.34612600 0.00013900

H 2.09539800 -1.30977600 -0.87648700

H 2.09536600 -1.31056600 0.87546300

**
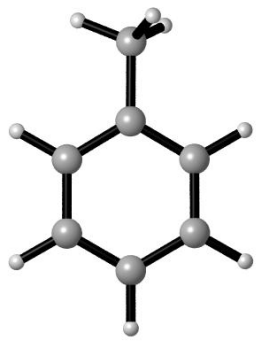
**

**1b’**

**G_gas_ = -271.282648 a.u.**

**E_gas_ = -271.381284923 a.u.**

C 2.42672800 0.00371000 0.00256200

C 0.91693400 0.00963000 -0.00342300

C 0.20166200 -1.20040700 -0.00257300

C 0.18878600 1.20855100 -0.00253500

C -1.19458100 -1.21176900 0.00059100

C -1.21054200 1.20283000 0.00057000

H 0.72506800 2.16207100 -0.00478500

C -1.90789300 -0.00754900 0.00237400

H -1.75646200 2.15012600 0.00062500

H -3.00089000 -0.01468900 0.00405200

H -1.73027800 -2.16494600 0.00065500

H 0.75080100 -2.14704800 -0.00503200

H 2.82738700 -0.59776600 -0.83021500

H 2.81857900 -0.43764400 0.93480500

H 2.83923000 1.01992500 -0.08550500

**
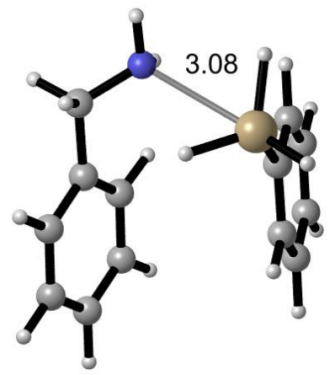
**

**TS1b**

**G_gas_ = -849.190326 a.u.**

**E_gas_ = -849.409731452 a.u.**

Si 1.33002400 2.01030600 1.34452600

H 1.96660300 2.04039300 2.70347200

C 1.75543100 0.34535900 0.56497100

C 2.35104400 0.25187900 -0.70618900

C 1.46741800 -0.85059700 1.24615100

C 2.63687300 -0.98947100 -1.28251900

H 2.58978100 1.16447800 -1.25976900

C 1.74884500 -2.09535200 0.67565900

H 1.00048000 -0.81823200 2.23552100

C 2.33200100 -2.16818600 -0.59274300

H 3.10117200 -1.03900300 -2.27151900

H 1.50451700 -3.01141400 1.21999000

H 2.55217400 -3.14036400 -1.04203400

N -0.21070600 2.19992500 -1.31538600

H 0.13897800 1.58446800 -2.04915400

H -0.07468700 3.15265200 -1.65232600

C -1.62682700 1.95543600 -1.04818300

C -1.90695700 0.56329600 -0.51499000

C -2.90638700 0.37344900 0.45213200

C -1.22141000 -0.56319100 -0.99178600

C -3.21798800 -0.90356300 0.92579700

C -1.52885500 -1.84216700 -0.52031300

H -0.41513200 -0.45609900 -1.72032600

C -2.52937000 -2.01894600 0.43880200

H -0.96800400 -2.70090700 -0.89607300

H -2.76637300 -3.01898600 0.81099700

H 1.90646200 3.13781900 0.56122000

H -0.13072000 2.19153800 1.56947400

H -3.44416000 1.24109300 0.84594600

H -3.99658300 -1.02660400 1.68335600

H -1.95819700 2.69044800 -0.29667600

H -2.27460100 2.11728500 -1.93849500

**
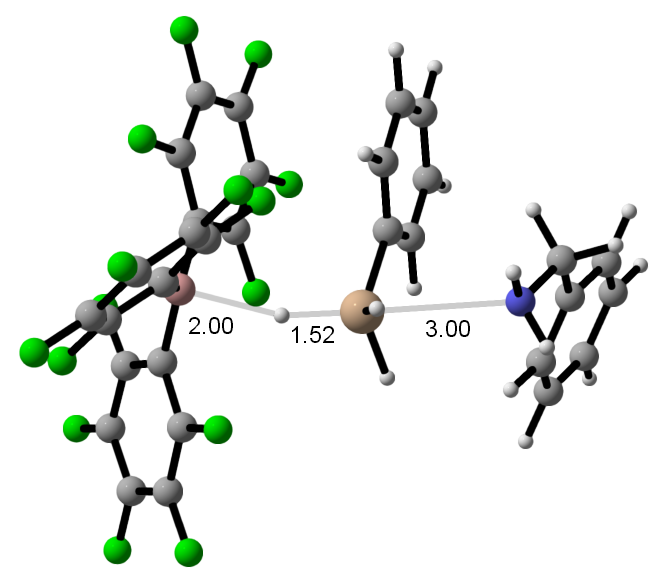
**

**TS2b**

**G_gas_ = -3055.747740 a.u.**

**E_gas_ = -3056.08564283 a.u.**

Si 1.04156500 -0.00358600 -1.66014100

H -0.24108300 0.21247500 -0.87850600

C 2.22009500 -0.85469400 -0.47265500

C 3.18979200 -0.10447700 0.21821500

C 2.14348400 -2.23851500 -0.21917200

C 4.05855200 -0.71383100 1.12739400

H 3.27942900 0.96949200 0.04306400

C 3.00335400 -2.84782000 0.69908100

H 1.40078000 -2.85030800 -0.73513900

C 3.96252500 -2.08569900 1.37620400

H 4.80501000 -0.11069400 1.64854500

H 2.92148000 -3.92090600 0.89151500

H 4.62906300 -2.56092300 2.10022100

B -1.72654900 0.03194700 0.45522200

C -1.99043400 1.59248800 0.46156800

C -2.15380800 2.31486200 -0.72887400

C -2.10064600 2.33191600 1.64656900

C -2.38606300 3.68938000 -0.75760500

C -2.34714200 3.70560700 1.66135000

C -2.48931800 4.38654800 0.44963500

C -2.62838200 -0.86862600 -0.48674100

C -3.96075600 -0.54027500 -0.77973300

C -2.14602700 -2.03196200 -1.10234000

C -4.76253200 -1.30552300 -1.63011400

C -2.90488900 -2.81429200 -1.96931700

C -4.22845600 -2.44742300 -2.23070800

C -0.78000400 -0.61471900 1.53794100

C -0.96034000 -1.91068600 2.04963700

C 0.35165200 0.06732200 2.01813000

C -0.07409100 -2.50297800 2.94990000

C 1.24629100 -0.48058900 2.93321400

C 1.04212800 -1.78512800 3.38433400

N 3.41211400 -0.64029900 -3.39362000

H 3.52653600 -0.09159800 -4.24742100

H 2.99199600 -1.52549500 -3.67418400

C 4.69983000 -0.87904600 -2.74908100

C 5.35619200 0.38037400 -2.21915600

C 6.59153600 0.27456500 -1.55922700

C 7.21688200 1.39993500 -1.02190000

H 7.06146500 -0.70843300 -1.45584900

C 6.61840100 2.66047200 -1.14136000

H 8.17502400 1.29539800 -0.50595600

H 7.10590500 3.54369100 -0.72099600

H 1.49237800 1.35839500 -2.03700300

H 0.61310900 -0.82933800 -2.81746800

F -2.45224900 4.36759600 2.80995600

F -2.72080600 5.69248700 0.44433200

F -2.51766400 4.33732600 -1.91179200

F -2.06984600 1.68827800 -1.90903600

F -1.98229300 1.72565300 2.83257700

F 0.64272000 1.29077300 1.56226400

F 2.30249400 0.20907900 3.35156200

F 1.90599600 -2.34001800 4.22266700

F -0.27794500 -3.74171200 3.39113100

F -2.02045800 -2.64227500 1.69225000

F -0.88576400 -2.43131600 -0.88054100

F -2.38993500 -3.89985000 -2.53983300

F -4.97338400 -3.18261200 -3.04432400

F -6.02352400 -0.95919900 -1.86926200

F -4.53323100 0.53450100 -0.22856000

C 5.39469100 2.77797700 -1.80319700

H 4.91721700 3.75645300 -1.90234300

C 4.76746900 1.64540800 -2.33707600

H 3.79833800 1.74622200 -2.82724400

H 5.42949000 -1.39920400 -3.40492300

H 4.52299100 -1.55450100 -1.89511500

**
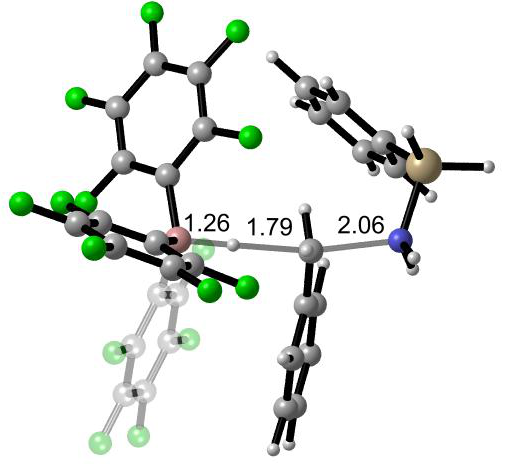
**

**TS3b**

**G_gas_ = -3055.727253 a.u.**

**E_gas_ = -3056.06604226 a.u.**

C -1.07309700 0.61763800 1.98358100

H -0.33177000 0.13430300 2.61001200

C -0.93658500 2.02700400 1.61818700

C 0.21796800 2.75035900 1.97220500

C -1.95103100 2.66053000 0.87285800

C 0.35655800 4.08040200 1.57706400

C -1.81509900 3.99491700 0.49591100

H -2.82918700 2.08793000 0.56440600

C -0.65770200 4.70380300 0.84089100

H -2.59212000 4.47403900 -0.10275600

H -0.53619000 5.73942200 0.51458000

N -2.42325800 0.75363200 3.53218600

H -1.85419400 0.77183900 4.38229500

Si -3.71094000 -0.52691400 3.49456300

H -3.01424700 -1.78706600 3.84872500

H -4.74080600 -0.16619300 4.50851500

C -4.37899600 -0.54633900 1.75567100

C -4.05545800 -1.60116700 0.88066800

C -5.15465500 0.52447500 1.26452800

C -4.49009400 -1.58484100 -0.44880600

H -3.44521400 -2.43898100 1.22333400

C -5.57533400 0.54809000 -0.06697200

H -5.43599700 1.35366800 1.92250000

C -5.24242300 -0.50800100 -0.92419600

H -4.23341400 -2.41602700 -1.10822000

H -6.16913900 1.38744300 -0.43747300

H -5.57266100 -0.49036900 -1.96571000

B 0.90671500 -0.28101900 -0.15761400

C 1.15555100 1.14176100 -0.89091800

C 0.07697800 1.81061100 -1.48191300

C 2.34817500 1.87281700 -0.85254700

C 0.14675000 3.10500500 -1.98868500

C 2.46786900 3.17368000 -1.35494800

C 1.35588700 3.79845400 -1.91813900

C 2.21220700 -0.97526400 0.51938700

C 3.07488000 -1.77809700 -0.23298700

C 2.56742300 -0.81959200 1.85745000

C 4.20550700 -2.40175400 0.29986200

C 3.68470800 -1.42071100 2.44043500

C 4.51059300 -2.22255900 1.65133600

C 0.01514700 -1.36833600 -0.97618900

C -0.79149000 -2.27509300 -0.28753000

C -0.03122100 -1.46861700 -2.36975700

C -1.60159900 -3.21894500 -0.91760200

C -0.84065200 -2.38671600 -3.04556300

C -1.64541900 -3.25872800 -2.31032100

F 1.82630600 -0.03289900 2.67991900

F 3.96919500 -1.23511400 3.73159200

F 5.58246700 -2.80818200 2.18168600

F 4.99561100 -3.15751500 -0.46189500

F 2.84368300 -1.95937200 -1.54143900

F -0.83940600 -2.25232100 1.06578800

F -2.39770700 -4.03166900 -0.20689700

F -2.46715000 -4.10946700 -2.92617600

F -0.86846200 -2.42602100 -4.37733900

F 0.71070500 -0.65494800 -3.12852900

F 3.45626000 1.35632300 -0.29977000

F 3.62743300 3.82934400 -1.27806100

F 1.43809000 5.05107600 -2.36623500

F -0.93783600 3.70623300 -2.48637600

F -1.12954800 1.21239300 -1.53492900

H 0.15123000 0.06095000 0.79548300

H -2.78296900 1.70146800 3.39645800

H 1.01967600 2.24438900 2.51399800

H 1.26782700 4.62871600 1.82603500

H -1.73729700 -0.01852800 1.40688500

**
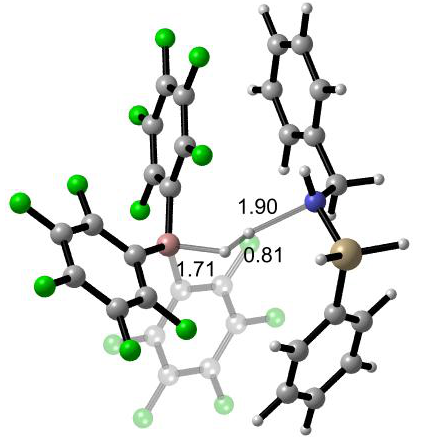
**

**TS4b**

**G_gas_ = -3055.751981 a.u.**

**E_gas_ = -3056.08585910 a.u.**

H -0.06318600 -0.32553400 -1.35039200

B 0.38061500 0.86668800 0.46655000

C 1.26254000 -0.25401000 1.16934300

C 2.52526000 -0.02709300 1.73231600

C 0.81734700 -1.58193300 1.20870000

C 3.30764500 -1.05063500 2.27410700

C 1.55887800 -2.62899200 1.74499300

C 2.82184600 -2.35858600 2.27373300

C -1.18865700 0.90181100 0.71548900

C -2.08015900 1.32554300 -0.27646700

C -1.75583100 0.59483100 1.95622100

C -3.45010300 1.46017500 -0.06404300

C -3.12221300 0.71955700 2.21133300

C -3.96896000 1.16920200 1.19820600

C 1.06149500 2.21470200 -0.04665100

C 2.23626500 2.20852900 -0.81043300

C 0.53437500 3.48293000 0.23649100

C 2.85322000 3.36200800 -1.28759700

C 1.12651800 4.66707300 -0.21341900

C 2.28990400 4.60488600 -0.98217500

F -0.37621800 -1.89709000 0.68397000

F 1.09595700 -3.87637100 1.72422800

F 3.56981000 -3.34922400 2.74577500

F 4.51214200 -0.79130300 2.77663200

F 3.04367200 1.20447700 1.77379900

F -0.98268500 0.17923500 2.96790100

F -3.62862200 0.39379400 3.39909400

F -5.27220700 1.28352800 1.42571200

F -4.26047000 1.85059700 -1.04453200

F -1.62062900 1.62527400 -1.50291300

F 2.81226400 1.03756000 -1.12254800

F -0.56606700 3.61772800 0.98366100

F 0.59400800 5.84766400 0.08781600

F 2.86109800 5.71912700 -1.41905100

F 3.96180500 3.29444800 -2.01925200

H 0.53400900 0.15240900 -1.08004400

N -1.08275600 -1.43171900 -2.50957100

H -1.66071600 -0.67659900 -2.87843900

Si 0.30534600 -1.89090700 -3.51377700

H 0.81400200 -0.63455900 -4.12639800

H -0.11674600 -2.86754300 -4.56472600

C -1.88434800 -2.51371600 -1.91085400

H -1.26383700 -3.02743600 -1.16285200

C 1.60587000 -2.66686900 -2.40534500

C 2.73518800 -1.92752600 -2.00407200

C 1.45348500 -3.97357900 -1.90020400

C 3.67242600 -2.46817000 -1.11919900

H 2.88384900 -0.91201900 -2.37485600

C 2.38597000 -4.51532200 -1.01124000

H 0.59418400 -4.58194600 -2.19932000

C 3.49702200 -3.76169800 -0.61847300

H 4.53477700 -1.87133300 -0.81126000

H 2.24159300 -5.52320400 -0.61531100

H 4.21540000 -4.17704100 0.09264100

C -3.16958000 -2.04369200 -1.26709000

C -3.41693100 -2.25235400 0.09725100

C -4.16784500 -1.43732400 -2.04744100

C -4.64018300 -1.88044100 0.66365500

C -5.38512900 -1.05364000 -1.48132500

H -3.99795600 -1.27556100 -3.11665100

C -5.62855500 -1.28348600 -0.12351100

H -6.14722800 -0.57574000 -2.10127700

H -6.57674900 -0.97879600 0.32385800

H -2.64282500 -2.70364300 0.72067800

H -4.81230700 -2.03564700 1.73184500

H -2.15016900 -3.27263700 -2.67682300

**
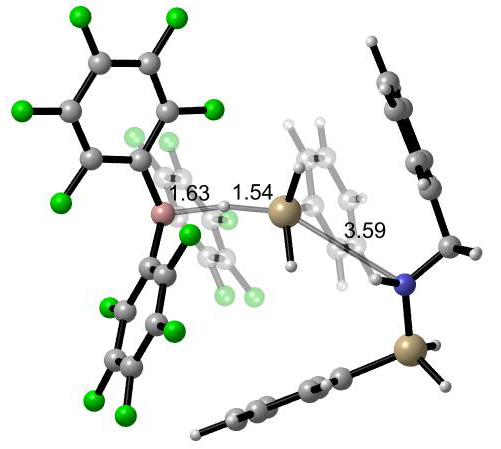
**

**TS5b**

**G_gas_ = -3577.215319 a.u.**

**E_gas_ = -3577.64243314 a.u.**

Si -0.81242400 -0.91154200 0.86468900

H 0.53856700 -0.29477600 0.45749000

C -1.38038500 -1.87459800 -0.63183500

C -2.52764500 -1.46420200 -1.33391800

C -0.64876800 -2.97373000 -1.12346600

C -2.93211000 -2.12426500 -2.49694100

H -3.09992000 -0.60967200 -0.97515000

C -1.04626800 -3.62814800 -2.29144300

H 0.24272500 -3.32000900 -0.59885400

C -2.18664900 -3.20223600 -2.98302800

H -3.81995900 -1.78338100 -3.03465200

H -0.46040900 -4.47019800 -2.66888200

H -2.48936400 -3.70828100 -3.90316700

B 1.72032900 0.51898900 -0.30979400

C 1.26458000 1.99002400 0.11108100

C 0.87674500 2.27659700 1.42437900

C 1.20530000 3.05719800 -0.79086900

C 0.41093500 3.52710100 1.82695000

C 0.75828900 4.32669700 -0.42512000

C 0.35537600 4.55785200 0.88966500

C 2.92789000 -0.11402200 0.52006200

C 3.96738300 0.67235900 1.03240500

C 3.01019000 -1.48199700 0.80111200

C 5.02165400 0.14006200 1.78068500

C 4.03494600 -2.05411300 1.55099900

C 5.05198400 -1.23074100 2.04376400

C 1.36993600 -0.01839300 -1.76672000

C 2.17992600 -0.89785200 -2.49949200

C 0.13664800 0.29071200 -2.36170900

C 1.78794000 -1.45150300 -3.72154500

C -0.28796500 -0.22926700 -3.58000000

C 0.54240200 -1.12220500 -4.25910000

H -1.65505500 0.26815600 1.15146800

H -0.49567400 -1.72404000 2.06224300

F 0.64917200 5.30117200 -1.32706700

F -0.15517800 5.73804500 1.22985300

F -0.02057400 3.73609700 3.06895800

F 0.90071600 1.30620700 2.35358700

F 1.56740300 2.88425200 -2.06677600

F -0.73301800 1.08346400 -1.71757000

F -1.48200800 0.08553500 -4.07615000

F 0.14616500 -1.65937300 -5.40553100

F 2.58610900 -2.29577300 -4.37062000

F 3.38737300 -1.25391900 -2.04933400

F 2.05736900 -2.31051300 0.34129500

F 4.05728500 -3.36133500 1.79726800

F 6.04352300 -1.75189900 2.75491400

F 5.99233400 0.92463400 2.23961500

F 3.99974200 1.98985900 0.80273400

N -4.24817300 -0.70374900 1.89491700

H -3.54225700 -0.25774100 2.47636900

Si -5.28637500 0.38201900 1.00006400

H -6.43427900 0.91102200 1.80769500

H -5.84989500 -0.37590700 -0.15550500

C -4.67254000 -1.99576500 2.43330100

C -4.23102100 1.81208500 0.38739300

C -3.67192700 2.73866100 1.29079100

C -3.94692100 1.97403000 -0.98184600

C -2.85754400 3.78240300 0.84377800

H -3.87390400 2.65276100 2.36375800

C -3.12979600 3.01437800 -1.43490500

H -4.36364400 1.27575300 -1.71352200

C -2.58715800 3.92231300 -0.52267700

H -2.42593300 4.48557800 1.56002400

H -2.90695900 3.10594800 -2.50056400

H -1.94804000 4.73609200 -0.87306800

C -3.48995900 -2.91594500 2.65598100

C -3.00980700 -3.73469600 1.62276500

C -2.80827600 -2.91102200 3.88280300

C -1.87822700 -4.53215200 1.81160200

C -1.67088800 -3.70161300 4.07333100

H -3.17547300 -2.28193600 4.69971500

C -1.20313100 -4.51527300 3.03632400

H -1.15014100 -3.68474300 5.03419600

H -0.31475500 -5.13482000 3.18260300

H -3.51768100 -3.73427500 0.65516600

H -1.51701000 -5.15930900 0.99316200

H -5.36507000 -2.45813000 1.71005800

H -5.23568200 -1.89397200 3.38350300

**
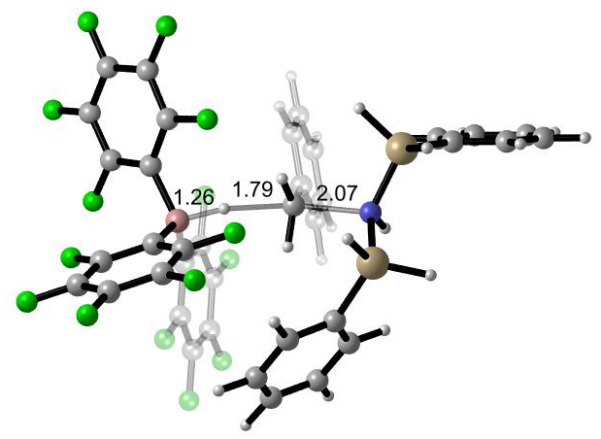
**

**TS6b**

**G_gas_ = -3577.188039 a.u.**

**E_gas_ = -3577.61775569 a.u.**

Si 4.12946500 0.36252200 -2.31034100

H 3.33051300 -0.79914800 -2.77535000

C 5.67466400 -0.11872500 -1.37818200

C 5.61201500 -1.02100000 -0.29586900

C 6.92122000 0.44648400 -1.71098600

C 6.76048400 -1.34372400 0.43049100

H 4.66264100 -1.48129700 -0.01264100

C 8.07129200 0.12029000 -0.98583000

H 6.99984400 1.14934700 -2.54579200

C 7.99123600 -0.77404800 0.08603100

H 6.69321500 -2.04449300 1.26641900

H 9.03158200 0.56579300 -1.25760400

H 8.88992900 -1.02846400 0.65384100

C 1.35513300 0.26352200 -0.67490800

C 1.88251600 -0.81396200 0.15660400

C 2.24053100 -0.55715500 1.49444900

C 2.00705100 -2.11859700 -0.35735700

C 2.71732700 -1.58643400 2.30353500

C 2.48157200 -3.14579500 0.45708800

H 1.69109900 -2.32063000 -1.38218300

C 2.83764200 -2.88100100 1.78456000

H 2.55975100 -4.16062700 0.06090400

H 3.19431000 -3.69143400 2.42466900

N 3.06675000 1.30532000 -1.17913700

H 3.50052400 1.27986600 -0.25111100

H 4.45844100 1.25353600 -3.45573000

Si 2.60379200 3.02237800 -1.56687900

H 3.87489900 3.79529200 -1.65796000

H 1.91215200 2.98363000 -2.87710600

C 1.48787600 3.59064800 -0.18351000

C 1.93045000 3.52256500 1.15403100

C 0.14940700 3.95305700 -0.43079900

C 1.05336900 3.77457400 2.21178500

H 2.96913700 3.25734200 1.38278800

C -0.72495400 4.21157300 0.62789700

H -0.22939100 3.99403600 -1.45426000

C -0.27924600 4.11067400 1.94843800

H 1.40340400 3.69933500 3.24447100

H -1.76884100 4.45591700 0.42228100

H -0.97624900 4.26422000 2.77379400

B -1.54001600 -0.40540000 -0.10658800

C -1.43877100 -0.14623400 1.49760300

C -0.75464100 -1.05796500 2.31385100

C -1.84767400 1.02118800 2.15315600

C -0.45922600 -0.83336800 3.65871500

C -1.58383200 1.28370400 3.50124900

C -0.86942600 0.35720700 4.25984100

C -2.44107000 0.65969600 -0.94716700

C -3.82709100 0.71371900 -0.76974100

C -1.92644600 1.58575400 -1.85063800

C -4.65034300 1.64404500 -1.40639600

C -2.70334100 2.54820200 -2.49971600

C -4.07929400 2.57845200 -2.27616900

C -1.84548600 -1.93166100 -0.57161700

C -1.25111400 -2.44581700 -1.72537700

C -2.67607400 -2.81964800 0.11827700

C -1.43803000 -3.75231600 -2.17936800

C -2.90477300 -4.13278600 -0.30272500

C -2.28059300 -4.60229400 -1.46099500

F -0.60319600 1.62119300 -2.13661300

F -2.13043200 3.44735800 -3.30974900

F -4.84142100 3.48474400 -2.88521600

F -5.96475100 1.66291600 -1.18787600

F -4.41141800 -0.13703800 0.08376900

F -0.41563500 -1.67465900 -2.46192900

F -0.81644100 -4.19328000 -3.27650900

F -2.48128300 -5.85333400 -1.87258400

F -3.70289900 -4.94455800 0.39099300

F -3.27509100 -2.43935600 1.25308100

F -2.51859400 1.98521200 1.49985600

F -1.98645800 2.42931200 4.06315200

F -0.58508000 0.60364000 5.53810700

F 0.24939000 -1.72489200 4.35829300

F -0.31051200 -2.21765000 1.80300400

H -0.35168800 -0.22146600 -0.47011000

H 2.11653800 0.45116800 1.89897700

H 2.96803900 -1.38921500 3.34751000

H 1.07521800 0.07372000 -1.70590600

H 0.95239400 1.15131600 -0.20145000

**
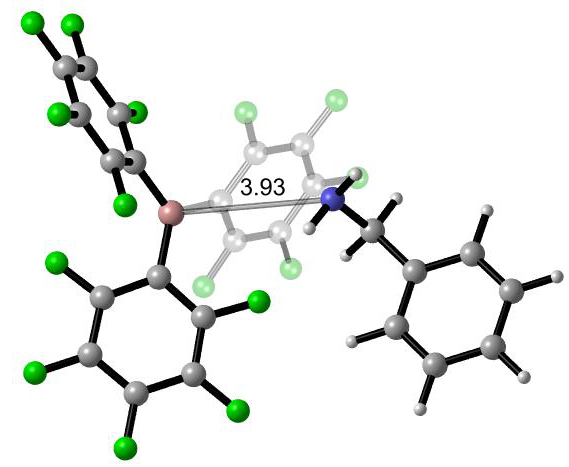
**

**TS10b**

**G_gas_ = -2533.122190 a.u.**

**E_gas_ = -2533.35258745 a.u.**

C 3.19760700 1.21854900 0.84425400

H 3.43477300 2.28703500 0.70697700

N 1.97229200 1.12730900 1.64541200

H 2.13786800 1.50559400 2.57904600

C -0.78131000 -1.53785900 -0.60863000

C 0.52782000 -2.02130500 -0.46908100

C -1.76241300 -2.51058700 -0.86012600

C 0.85922000 -3.37161600 -0.55494200

C -1.47100100 -3.87053500 -0.97385900

C -0.15050400 -4.30282100 -0.81490100

C -0.14655900 1.06602000 -1.04409500

C 0.09187400 2.27462600 -0.36715000

C 0.62427900 0.84208300 -2.19862000

C 1.05479600 3.18981100 -0.78680400

C 1.57425600 1.74807700 -2.66299000

C 1.80044500 2.92159100 -1.93536300

C -2.45190900 0.42326200 0.25195400

C -3.19887100 1.54026100 -0.16028600

C -2.96097600 -0.28174700 1.35570200

C -4.37987600 1.93413600 0.46782800

C -4.12843700 0.09147400 2.02028100

C -4.84242600 1.20527200 1.56780200

F 2.11600100 -3.77542800 -0.38992300

F 1.53641200 -1.17575400 -0.21136100

F 0.14215100 -5.59029200 -0.90916700

F -2.42958200 -4.75426200 -1.22665700

F -3.03804100 -2.15001400 -1.02027800

F -2.31040300 -1.34738000 1.83257900

F -4.56615000 -0.59421200 3.07093900

F -5.95719100 1.56997300 2.18154700

F -5.06456200 2.98789400 0.03695700

F -2.80238800 2.26455800 -1.20835300

F -0.58749500 2.57769800 0.73494900

F 1.30990000 4.28459000 -0.07640400

F 2.73256300 3.77530700 -2.33172700

F 2.27566900 1.50457100 -3.76634200

F 0.45273200 -0.27621600 -2.91183400

B -1.13123600 -0.00696800 -0.47737900

C 4.39672800 0.48987800 1.41979600

C 5.36540000 1.17256800 2.17032500

C 4.52572200 -0.90107600 1.26204200

C 6.43653700 0.48748900 2.75293500

C 5.59431700 -1.58835900 1.84296000

H 3.78183400 -1.44975900 0.67802200

C 6.55318300 -0.89611400 2.59122200

H 5.68266600 -2.66954600 1.70663300

H 7.39087800 -1.43363500 3.04296800

H 5.28167400 2.25676700 2.29526900

H 7.18436600 1.03658500 3.33132100

H 1.73143800 0.14466400 1.77875900

H 2.97066100 0.81823500 -0.15582500

**
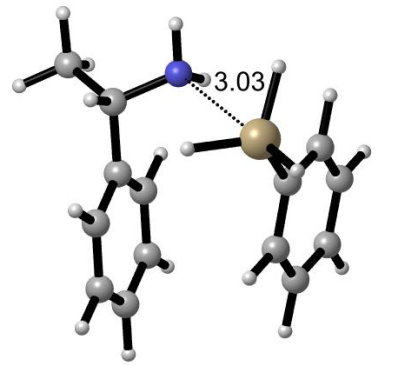
**

**Int1c**

**G_gas_ = -888.458964 a.u.**

**E_gas_ = -888.703274953 a.u.**

Si -1.12360700 -2.22142100 1.44123900

H -2.05974100 -2.37668200 2.60603000

C -1.75218900 -0.77742300 0.40219000

C -2.18640600 -0.95925300 -0.92406300

C -1.80742500 0.52037200 0.94028500

C -2.65500600 0.11501800 -1.68681600

H -2.15591400 -1.95712500 -1.37244400

C -2.27306200 1.59847700 0.18257100

H -1.46580500 0.70483900 1.96310400

C -2.69870600 1.39889600 -1.13364200

H -2.98929000 -0.05001300 -2.71503700

H -2.29202100 2.60033600 0.61886900

H -3.06171300 2.24167300 -1.72811400

N 0.94259200 -2.01747700 -0.76948100

H 0.48044000 -1.55569100 -1.55313500

H 1.20259900 -2.95267700 -1.08501600

C 2.13012500 -1.26806800 -0.34097500

C 3.29098600 -1.31840300 -1.35016100

H 2.99975500 -0.86195000 -2.31039900

H 4.17352100 -0.77759000 -0.97244500

H 3.58710900 -2.36253100 -1.54709600

C 1.74648700 0.16511300 0.00078700

C 1.87540600 0.65067100 1.30902800

C 1.26610000 1.03701400 -0.98907700

C 1.54043900 1.97199400 1.62237500

C 0.92559500 2.35502400 -0.68089500

H 1.14661200 0.68557200 -2.01772200

C 1.06599800 2.82948400 0.62693000

H 0.54119200 3.01261900 -1.46434100

H 0.79965500 3.86149100 0.86929100

H -1.16851100 -3.49815300 0.67675800

H 0.22040000 -1.97463900 2.03246100

H 2.23853400 -0.01709100 2.09482000

H 1.64885000 2.33020300 2.64955300

H 2.47663200 -1.74385900 0.59267200

**
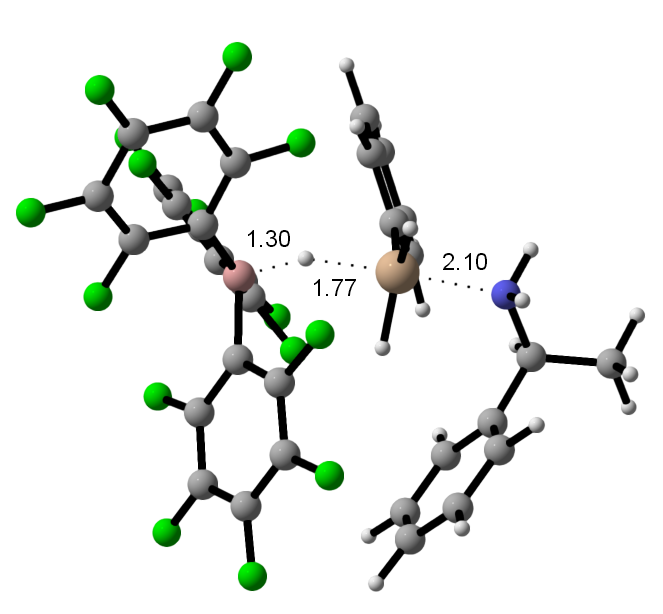
**

**Int2c**

**G_gas_ = -3095.025512 a.u.**

**E_gas_ = -3095.39541706 a.u.**

Si 1.13081400 -0.64565400 -1.77862500

H -0.30359900 -0.07688200 -0.91102200

C 0.66531600 -2.43604600 -1.48366900

C 1.47024300 -3.30539200 -0.72238400

C -0.57228100 -2.91384900 -1.95670100

C 1.06084500 -4.61301300 -0.45022800

H 2.39959300 -2.94660200 -0.27515800

C -0.98446200 -4.22114500 -1.68331300

H -1.23641800 -2.24919700 -2.51441400

C -0.16900800 -5.07243000 -0.93073300

H 1.68649200 -5.26159400 0.16700300

H -1.95443800 -4.57078700 -2.04550100

H -0.50011700 -6.08878100 -0.70328600

B -0.95384600 0.39095300 0.11313100

C 0.05867200 1.47342600 0.78430600

C 0.75904100 2.36653400 -0.03115400

C 0.32299100 1.58500100 2.15219700

C 1.70134600 3.27719700 0.44539500

C 1.26010800 2.48047200 2.67700800

C 1.95423400 3.32986600 1.81495200

C -2.23972500 1.10897800 -0.56151800

C -3.00828200 2.01794500 0.16993600

C -2.66819900 0.87193900 -1.86628800

C -4.12640900 2.66986700 -0.35352300

C -3.77815000 1.50008900 -2.43599000

C -4.51089500 2.40933000 -1.67131800

C -1.25348400 -0.95823900 0.96514600

C -2.51125100 -1.55287700 1.10928300

C -0.19343000 -1.69311200 1.50556200

C -2.70476700 -2.79483200 1.72662800

C -0.33402200 -2.93596800 2.11464100

C -1.60765000 -3.49562300 2.22524900

N 2.88460800 -1.05698900 -2.85344900

H 3.01748900 -0.23917500 -3.45774300

H 2.67826600 -1.83563300 -3.48799900

C 4.17632600 -1.33323400 -2.12601100

C 4.45146400 -0.21405600 -1.13667500

C 4.41085700 -0.46126500 0.24203900

C 4.54814700 0.58606700 1.15723300

H 4.23059900 -1.47532900 0.60862400

C 4.72736300 1.89499000 0.70243200

H 4.48621100 0.38126100 2.22844000

H 4.79153400 2.72125600 1.41337900

H 1.92132600 0.10559300 -0.80298700

H 0.56110500 -0.01468300 -2.98747300

F 1.50271000 2.52664800 3.98712500

F 2.88761600 4.15770500 2.29105800

F 2.38554500 4.06407100 -0.39058800

F 0.57253800 2.33669600 -1.36724500

F -0.31218900 0.80491100 3.03410500

F 1.06779800 -1.22186400 1.39707000

F 0.73440800 -3.61091900 2.54739900

F -1.76843700 -4.69271000 2.78567200

F -3.92624400 -3.32001500 1.82589800

F -3.61618300 -0.96109500 0.63420600

F -2.01350900 -0.01155500 -2.65098200

F -4.14273700 1.24013300 -3.69293100

F -5.57273400 3.02020800 -2.19273200

F -4.82927500 3.52833800 0.38459200

F -2.69321300 2.27513900 1.44844900

C 4.79040400 2.14956500 -0.67167500

H 4.91473800 3.17256400 -1.03119400

C 4.66035300 1.10013900 -1.58334100

H 4.69963700 1.32233100 -2.65436500

C 5.31586400 -1.57324700 -3.11831300

H 5.08546900 -2.40982600 -3.79833300

H 5.51511700 -0.67605700 -3.72617500

H 6.23978000 -1.81881300 -2.57448700

H 3.99855900 -2.25999500 -1.56299400

**
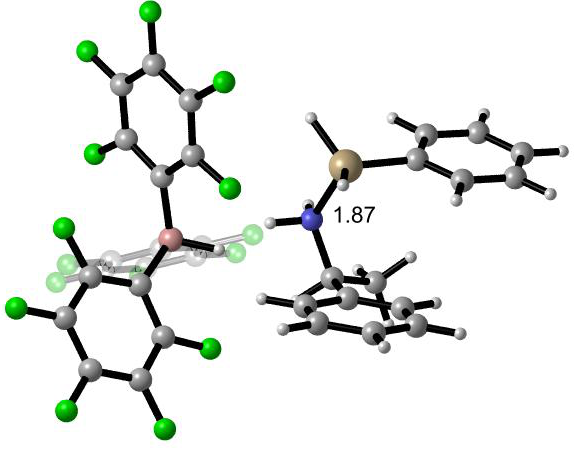
**

**Int3c**

**G_gas_ = -3095.030166 a.u.**

**E_gas_ = -3095.39658978 a.u.**

C 2.37527200 -0.55887600 -1.57826100

H 1.36378000 -0.69182100 -1.99020100

C 2.86067500 -1.91096500 -1.08797600

C 1.97175600 -2.71611400 -0.35382500

C 4.16682800 -2.36810800 -1.30993500

C 2.38884600 -3.94248000 0.16347000

C 4.58301100 -3.60084800 -0.79478400

H 4.87269000 -1.76622400 -1.88376900

C 3.69783100 -4.38765800 -0.05380400

H 5.60328700 -3.94717400 -0.97804300

H 4.02371600 -5.35009400 0.34818800

C 3.23694700 0.15019000 -2.61432900

H 4.23392700 0.40831300 -2.22985100

H 3.35959100 -0.49774000 -3.49386800

H 2.73724000 1.07367500 -2.94582600

N 2.15149700 0.35402300 -0.36946200

H 1.98692700 1.30382100 -0.72463400

Si 3.34992300 0.41907600 1.07097500

H 2.69324700 1.44545600 1.91259000

H 3.40245800 -0.92678100 1.66407800

C 4.98607700 0.98405600 0.38889300

C 5.16446500 2.31569400 -0.03716400

C 6.05546300 0.07962700 0.24023500

C 6.36902300 2.72692800 -0.61213900

H 4.35772500 3.04695700 0.07983600

C 7.26313200 0.49130100 -0.33012200

H 5.94119300 -0.95858700 0.56273000

C 7.41817400 1.81283200 -0.76128400

H 6.49240400 3.76180700 -0.94047600

H 8.08526900 -0.22039900 -0.43903100

H 8.36162700 2.13405300 -1.20983400

B -1.31999800 0.13341400 0.01641800

C -2.07338500 -1.31112800 0.01061000

C -1.72324700 -2.26459500 -0.94902800

C -3.06499100 -1.70595300 0.91161600

C -2.28808300 -3.53954800 -1.01799200

C -3.66251800 -2.96995900 0.88268500

C -3.27058300 -3.89230500 -0.09021300

C -1.16324800 0.80225200 1.50113400

C -1.95684300 1.82970000 2.02271400

C -0.15522500 0.36110100 2.35845800

C -1.74675400 2.39530700 3.28523700

C 0.10945800 0.89792500 3.61790900

C -0.70428000 1.92828300 4.08989200

C -1.89908700 1.14560500 -1.12831000

C -1.06004500 1.88917300 -1.95576500

C -3.26532000 1.30644100 -1.38530500

C -1.50421400 2.73521800 -2.97355000

C -3.76567900 2.13818000 -2.39050000

C -2.87642600 2.85979400 -3.19151000

F 0.67949600 -0.64144800 1.96067800

F 1.13214400 0.44992500 4.35235700

F -0.48462400 2.46532400 5.28796100

F -2.53004300 3.37624200 3.73027000

F -2.97602800 2.32746100 1.31455600

F 0.29670000 1.81975900 -1.81066500

F -0.63756400 3.41372800 -3.73065800

F -3.33368300 3.65868300 -4.15335000

F -5.07703200 2.25590600 -2.59220200

F -4.16599800 0.65014800 -0.64613300

F -3.49095700 -0.86174500 1.86062800

F -4.60314600 -3.30302400 1.76683100

F -3.82854100 -5.10132300 -0.13251900

F -1.90294100 -4.41550000 -1.94882400

F -0.77968400 -1.97594500 -1.87473200

H -0.18771900 -0.14860400 -0.35294600

H 1.19230100 0.10265400 -0.02049900

H 0.94939700 -2.37659300 -0.18140100

H 1.68647500 -4.55515700 0.73331500

**
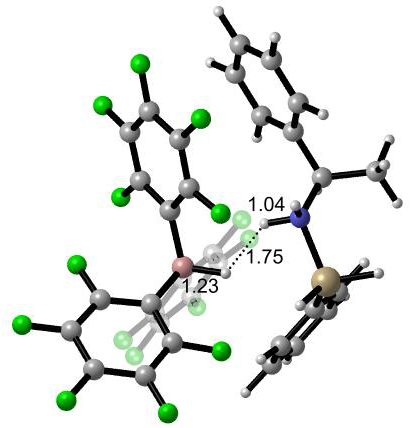
**

**Int4c**

**G_gas_ = -3095.038025 a.u.**

**E_gas_ = -3095.41049962 a.u.**

H -1.24464000 -0.58806900 -1.24553800

B 0.73027200 0.49388200 -0.04149200

C 1.15864100 -0.73736600 0.94505600

C 2.39971000 -0.93187600 1.55753800

C 0.25257000 -1.77630300 1.14789700

C 2.71898100 -2.08156700 2.29179000

C 0.50966200 -2.93418800 1.87189200

C 1.76980500 -3.09158600 2.44844000

C -0.59604600 1.31630700 0.46373100

C -1.42264200 1.95730300 -0.46088400

C -0.99207200 1.45490400 1.79815200

C -2.56201600 2.68698600 -0.12291400

C -2.12695500 2.17121400 2.18841100

C -2.91182000 2.79911200 1.22094400

C 1.92502800 1.53232900 -0.42265400

C 2.60386700 1.48247800 -1.63930500

C 2.32686600 2.55442900 0.44196500

C 3.61356200 2.37985300 -1.99907600

C 3.32851300 3.47556200 0.13146800

C 3.97496700 3.38776100 -1.10457200

F -0.98130800 -1.70032900 0.57450900

F -0.40609200 -3.90314900 1.97027700

F 2.07404600 -4.21245500 3.10357900

F 3.93128200 -2.22944300 2.82727100

F 3.37495000 -0.01976000 1.44734700

F -0.28428100 0.88087300 2.77807200

F -2.48550700 2.24176900 3.47108200

F -4.01484300 3.45940700 1.57444600

F -3.33245600 3.23743100 -1.06552000

F -1.15825400 1.85596500 -1.79209700

F 2.31275500 0.52520700 -2.55138700

F 1.75408700 2.66434100 1.65026100

F 3.67703000 4.43080700 0.99394300

F 4.93336900 4.25626200 -1.42353100

F 4.22895200 2.28375500 -3.18017400

H 0.41800700 -0.05823800 -1.09421100

N -1.78479700 -0.90559500 -2.07339400

H -2.08731000 -0.02175800 -2.50380300

Si -0.44440000 -1.69103100 -3.16108700

H 0.34432200 -0.53067100 -3.61240500

H -1.23093200 -2.28966600 -4.26676900

C -3.00126900 -1.68093100 -1.58443000

H -2.58719900 -2.45151300 -0.92213700

C 0.47153800 -2.91897500 -2.12073300

C 1.80099500 -2.65439600 -1.73388700

C -0.14337400 -4.10706500 -1.67515300

C 2.49433900 -3.55917100 -0.92674600

H 2.28713700 -1.72834400 -2.04582200

C 0.54424300 -4.99717400 -0.84893100

H -1.17169300 -4.34381000 -1.96622100

C 1.86505200 -4.72290700 -0.47637200

H 3.52046100 -3.33860100 -0.62441500

H 0.04915300 -5.90054000 -0.48638200

H 2.39719200 -5.40956700 0.18647600

C -3.87941400 -0.75126600 -0.76355400

C -3.90365600 -0.85300100 0.63503700

C -4.67486900 0.22232900 -1.38960700

C -4.71872300 -0.00573800 1.39041300

C -5.48809400 1.06893800 -0.63395700

H -4.66563900 0.33147400 -2.47778200

C -5.51687100 0.95146000 0.75856200

H -6.08923800 1.83183200 -1.13270000

H -6.13783900 1.62503500 1.35196100

C -3.74253100 -2.35009300 -2.74088900

H -4.66669300 -2.80170400 -2.35377800

H -4.02389700 -1.62919100 -3.52405700

H -3.14668600 -3.14603300 -3.20989700

H -3.26781800 -1.58533700 1.13516200

H -4.71435600 -0.08142300 2.48025600

**
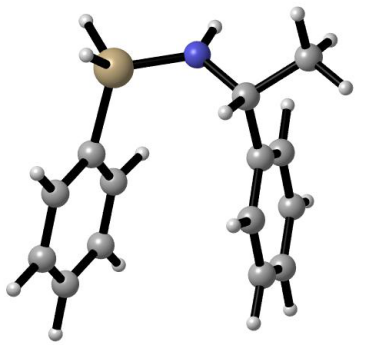
**

**Int5c**

**G_gas_ = -887.301778 a.u.**

**E_gas_ = -887.528379179 a.u.**

N -1.44590400 -1.88774300 -0.43275800

H -1.99503100 -1.89241000 -1.28638200

Si 0.17723800 -2.51216200 -0.48308400

H 0.28864900 -3.20648900 -1.79937900

H 0.41928300 -3.48403700 0.62834000

C -1.94607600 -0.97485700 0.59392900

H -1.48372700 -1.27816600 1.55062700

C 1.47524600 -1.15969500 -0.28067200

C 1.47136700 -0.02863100 -1.12026900

C 2.42505200 -1.20975900 0.75537100

C 2.37951100 1.01397200 -0.93189400

H 0.72741600 0.05670600 -1.91739700

C 3.34056000 -0.16880000 0.94842600

H 2.44732400 -2.07092800 1.43099600

C 3.31704400 0.94548000 0.10529300

H 2.34746000 1.88921200 -1.58568100

H 4.06926700 -0.22533700 1.76177600

H 4.02562900 1.76403300 0.25841600

C -1.54175800 0.48004400 0.34847600

C -0.79341800 1.19011000 1.29483600

C -1.87931000 1.12022600 -0.85390200

C -0.38204800 2.50374500 1.04790100

C -1.47603800 2.43295000 -1.10520800

H -2.45058200 0.57835700 -1.61343100

C -0.72162000 3.12969300 -0.15373100

H -1.74573900 2.91445900 -2.04912600

H -0.39690600 4.15430000 -0.35235500

C -3.46466600 -1.12098100 0.75061000

H -3.84474700 -0.46949300 1.55233200

H -3.97968500 -0.83442100 -0.18215400

H -3.72563600 -2.16554500 0.98007200

H -0.50365600 0.69949900 2.22855800

H 0.21760700 3.03374600 1.79228900

**
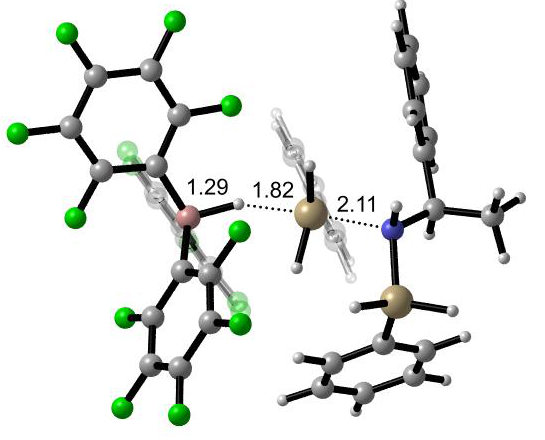
**

**Int6c**

**G_gas_ = -3616.478324 a.u.**

**E_gas_ = -3616.93738135 a.u.**

Si 1.09807400 -0.30523300 -1.15758900

H -0.59606100 -0.30495300 -0.49680100

C 1.71274700 -1.10509300 0.41368400

C 2.68035800 -0.50292000 1.23788700

C 1.11489800 -2.30512200 0.84179600

C 3.05291500 -1.09047100 2.44947400

H 3.13462900 0.44677300 0.95837900

C 1.48225300 -2.88879100 2.05565800

H 0.34559800 -2.78050300 0.22997800

C 2.45285100 -2.28393600 2.86157600

H 3.79138400 -0.59610300 3.08464900

H 0.99970200 -3.81401600 2.37980500

H 2.72654200 -2.73281900 3.81974900

B -1.65427100 0.16155700 0.07033500

C -1.75926200 1.69948400 -0.45568400

C -1.47554400 2.00742600 -1.78832600

C -2.09376500 2.79470500 0.34581900

C -1.44663700 3.30589500 -2.29980000

C -2.09907700 4.11170600 -0.12386700

C -1.77079700 4.37010400 -1.45700800

C -2.85558000 -0.75712700 -0.51344000

C -4.18685900 -0.35174300 -0.38765000

C -2.65948300 -1.98399600 -1.14515500

C -5.26407300 -1.09857200 -0.86894400

C -3.70273900 -2.76682300 -1.64505200

C -5.01663700 -2.31673800 -1.50676700

C -1.30415400 -0.03937600 1.64434400

C -1.87492800 -1.00149900 2.48299500

C -0.24328200 0.67122500 2.21215800

C -1.41366200 -1.25638300 3.78053600

C 0.25911900 0.44796500 3.48922900

C -0.33220300 -0.53462500 4.28393300

H 0.92479300 1.15103400 -1.16744900

H 0.52744300 -1.16086500 -2.21037900

F -2.41465300 5.12329400 0.68484100

F -1.76212300 5.61954300 -1.91749300

F -1.10809200 3.53311900 -3.57203600

F -1.16162000 1.01907400 -2.65405900

F -2.41041400 2.62076100 1.63409000

F 0.39986900 1.60470000 1.47801700

F 1.32053000 1.12812900 3.93410400

F 0.14655000 -0.79360700 5.49978900

F -1.98296800 -2.20210600 4.52944900

F -2.89488300 -1.76544300 2.06583400

F -1.41462300 -2.48825600 -1.29091600

F -3.45738900 -3.93308000 -2.24612500

F -6.02760900 -3.04600300 -1.97513100

F -6.51736100 -0.66897100 -0.72402000

F -4.47457500 0.79641300 0.24395400

N 2.87986100 -0.05593100 -2.26933600

H 2.55667500 -0.36210800 -3.19423000

Si 3.18100500 1.77023600 -2.42246100

H 1.92539800 2.31123900 -2.99378500

H 4.29823300 1.95064400 -3.38386100

C 4.04727700 -0.94144200 -1.89528800

H 4.37546300 -0.59262600 -0.90593600

C 3.61740300 2.41084500 -0.72980800

C 2.64643000 2.97154300 0.12313200

C 4.92194700 2.21672300 -0.22843500

C 2.95661200 3.28992200 1.44752400

H 1.62424800 3.12664700 -0.22781700

C 5.23510600 2.54263800 1.09323700

H 5.70348400 1.79803800 -0.87010700

C 4.24791200 3.06950300 1.93444600

H 2.17719600 3.67675000 2.10632100

H 6.24766100 2.37947200 1.47052000

H 4.48563600 3.30556700 2.97462500

C 3.62264600 -2.39644700 -1.75836400

C 4.05053200 -3.14031200 -0.65030500

C 2.85003400 -3.03292300 -2.74029400

C 3.69817300 -4.48370700 -0.51458000

C 2.48489800 -4.37498500 -2.60229100

H 2.50976800 -2.48564300 -3.62454600

C 2.90762900 -5.10369700 -1.48707800

H 1.86617400 -4.85002400 -3.36720000

H 2.61708400 -6.15094500 -1.37457500

C 5.21953700 -0.82562400 -2.87941000

H 6.00675100 -1.53495900 -2.58564000

H 4.90148600 -1.08775300 -3.90180800

H 5.66042600 0.18130900 -2.90022000

H 4.63031900 -2.65114300 0.13625900

H 4.02385700 -5.04143600 0.36645300

**
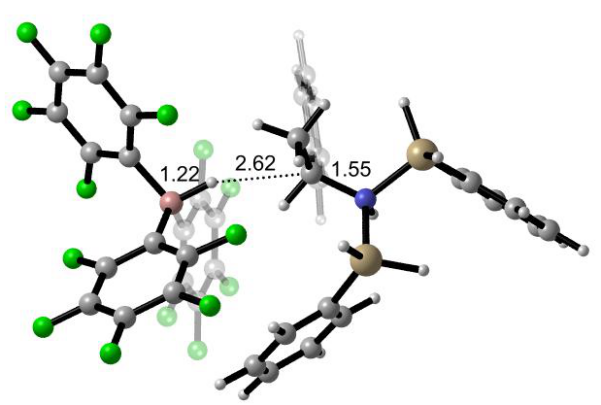
**

**Int7c**

**G_gas_ = -3616.473900 a.u.**

**E_gas_ = -3616.93107798 a.u.**

Si 4.46335300 -0.50194400 -2.55695500

H 3.95085900 -1.83266500 -2.93955200

C 5.93732100 -0.56392400 -1.42477400

C 5.91925400 -1.45539600 -0.33013200

C 7.04484100 0.29017100 -1.58722600

C 6.98012800 -1.48423400 0.57796100

H 5.07096500 -2.13139900 -0.17805600

C 8.10802600 0.25500000 -0.68050400

C 8.07455000 -0.62976300 0.40208300

H 6.95480400 -2.17771600 1.42202300

H 8.96317700 0.92129600 -0.81735400

H 8.90515100 -0.65450700 1.11193600

C 1.72389000 -0.38971400 -1.53483700

H 1.07828500 0.25995400 -0.92916100

C 1.83005600 -1.72604800 -0.82074000

C 2.21116900 -1.72912600 0.53337600

C 1.56524100 -2.94914700 -1.44688400

C 2.35834100 -2.92504300 1.23446800

C 1.69551200 -4.14922300 -0.73991800

H 1.23471100 -2.97708700 -2.48569800

C 2.09806000 -4.14181800 0.59642700

H 1.46889000 -5.09476400 -1.23812800

H 2.18365600 -5.07871400 1.15126900

C 1.17537800 -0.41267800 -2.95112700

H 1.76108700 -1.05538600 -3.62612600

H 0.14359900 -0.78782800 -2.91832600

H 1.12833000 0.60093800 -3.36991300

N 3.12798400 0.27547300 -1.49012200

H 3.47075100 0.06053900 -0.54388000

H 4.63519500 0.40753800 -3.71480000

Si 3.11358600 2.16229900 -1.46143300

H 4.56412200 2.45289000 -1.60832500

H 2.34561000 2.60481600 -2.64188300

C 2.38275000 2.61284200 0.17737000

C 2.89556700 2.05137600 1.36628600

C 1.25130100 3.44786800 0.25270700

C 2.26752800 2.28754700 2.59037000

H 3.79109100 1.41875400 1.34833800

C 0.63336300 3.69089100 1.48048900

H 0.82863400 3.88539500 -0.65313000

C 1.13157700 3.10285200 2.64557400

H 2.65128500 1.82623600 3.50345700

H -0.26576400 4.30842300 1.52117900

H 0.61480200 3.24770600 3.59511100

B -1.76530700 -0.11808700 -0.12621600

C -1.26302200 -0.36541400 1.41333200

C -0.78723000 -1.63581200 1.76237400

C -1.16284500 0.58732300 2.42954600

C -0.24482900 -1.95044600 3.00798400

C -0.60876900 0.32335300 3.68742000

C -0.13334300 -0.95322500 3.97831900

C -2.21845600 1.41853900 -0.45865600

C -3.41222000 1.96379500 0.02549700

C -1.45675900 2.28516500 -1.23574900

C -3.81741800 3.27813100 -0.21579000

C -1.80865800 3.61123800 -1.49777200

C -3.00006900 4.11630200 -0.98084800

C -2.93001300 -1.15404100 -0.61837000

C -3.03463800 -1.46857800 -1.97532700

C -3.86272000 -1.78259800 0.20962500

C -3.98160700 -2.35209700 -2.49742100

C -4.82996400 -2.67472200 -0.26417800

C -4.89129200 -2.95898500 -1.62942200

F -0.27413600 1.88816300 -1.77295100

F -0.98763700 4.40610400 -2.20478800

F -3.35619100 5.38012200 -1.21297500

F -4.96032800 3.75066900 0.28359000

F -4.21128200 1.21787700 0.79859700

F -2.18588800 -0.90928900 -2.86697400

F -4.02823000 -2.62011000 -3.80676500

F -5.80827500 -3.80594500 -2.10119200

F -5.69596100 -3.25449200 0.57077100

F -3.85774500 -1.55728200 1.53211500

F -1.59140100 1.85102200 2.24594000

F -0.50825500 1.29339800 4.60799700

F 0.41627200 -1.22108000 5.16579900

F 0.21266600 -3.18178600 3.27004600

F -0.82192300 -2.63040700 0.86012800

H -0.79419700 -0.37494600 -0.81315600

H 7.08123700 0.99114700 -2.42620800

H 2.62478000 -2.90762800 2.29253900

H 2.34722900 -0.78067700 1.06395200

**
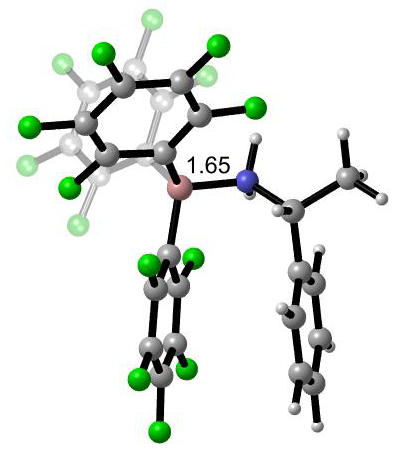
**

**Int11c**

**G_gas_ = -2572.431411 a.u.**

**E_gas_ = -2572.69693725 a.u.**

C 1.20350200 -1.09226300 2.32238400

H 0.93769900 -1.99826600 1.76334900

N 0.22394500 -0.03028700 1.86723700

H -0.60879800 -0.08321500 2.45912600

H 0.63015000 0.89410200 2.04123000

C -1.33663000 -1.22829500 0.00116700

C -1.87094300 -2.13350200 0.91592200

C -1.86307400 -1.32644300 -1.29400400

C -2.82844500 -3.09909000 0.58918700

C -2.81432300 -2.27138400 -1.67437300

C -3.30460900 -3.16731300 -0.71913900

C 1.05681400 0.03611000 -0.59133300

C 1.94185500 1.10700200 -0.43596200

C 1.51097700 -0.98124500 -1.43286100

C 3.18398700 1.19589300 -1.05416900

C 2.74141900 -0.92999600 -2.09673800

C 3.58570200 0.16066200 -1.89983700

C -1.22571200 1.37817500 0.19828100

C -1.17581000 2.29591700 -0.85866300

C -2.22209700 1.63499200 1.14642300

C -2.03237700 3.39818500 -0.95353600

C -3.09715100 2.72020600 1.09902900

C -2.99868300 3.61323800 0.03049400

F -3.28863500 -3.93923600 1.51452600

F -1.47587800 -2.11758500 2.21204900

F -4.21493000 -4.07461800 -1.05627300

F -3.26540100 -2.32329400 -2.92467900

F -1.43555200 -0.47811000 -2.23923400

F -2.38865700 0.78378500 2.19220100

F -4.01589600 2.90298800 2.04477200

F -3.81635300 4.65701000 -0.04764400

F -1.93540900 4.24256400 -1.97664600

F -0.29139600 2.15943000 -1.85060400

F 1.61896900 2.10572700 0.41705100

F 4.00392100 2.21596100 -0.80961800

F 4.78546800 0.19311000 -2.47115500

F 3.13873400 -1.94389300 -2.86451800

F 0.80653900 -2.11000500 -1.60382400

B -0.30825100 0.00925100 0.30531400

C 2.63763400 -0.71242700 1.99755200

C 3.42849700 -1.55742400 1.20837600

C 3.20578900 0.46769000 2.50248200

C 4.74978700 -1.21832400 0.90492600

C 4.52128200 0.81586600 2.19024900

H 2.61741300 1.14087900 3.13327400

C 5.29676600 -0.02635400 1.38754700

H 4.93986800 1.75112400 2.56856500

H 6.32147700 0.25122700 1.13066100

C 1.01258600 -1.35231500 3.81953700

H -0.00924200 -1.69595400 4.03549800

H 1.21887800 -0.44427800 4.40848500

H 1.71591500 -2.12963400 4.15089100

H 2.99529400 -2.47384500 0.79927200

H 5.34479100 -1.87626600 0.26713200

**
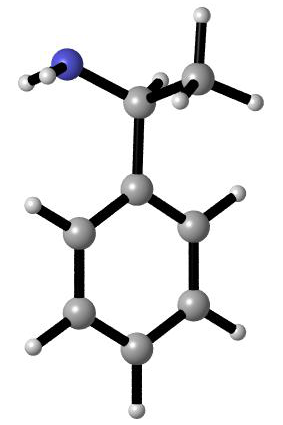
**

**1c**

**G_gas_ = -365.836922 a.u.**

**E_gas_ = -365.977739839 a.u.**

C 1.66775200 -0.24159400 -0.32314300

H 1.83903200 -0.93330300 -1.16878000

C 0.14941900 -0.08606900 -0.18467400

C -0.68826700 -1.19337300 -0.39686100

C -0.44076600 1.12991900 0.18472800

C -2.07188500 -1.09189800 -0.23831500

C -1.82645800 1.23799500 0.34698700

H 0.18925200 2.00905500 0.34010600

C -2.64754600 0.12732800 0.13707800

H -2.26548900 2.19691800 0.63559100

H -3.73047300 0.21091600 0.25938700

C 2.25374800 -0.90014200 0.93432400

H 2.08847200 -0.25511500 1.81438000

H 1.77257900 -1.86804800 1.13859700

H 3.33685200 -1.05354300 0.81235300

N 2.41831800 0.97486200 -0.61272400

H 1.99814400 1.48831900 -1.38717800

H 2.41907000 1.60318100 0.19126800

H -2.70537600 -1.96576000 -0.41272800

H -0.24627000 -2.14964700 -0.69467700

**
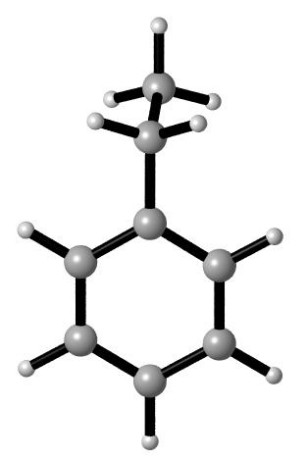
**

**1c’**

**G_gas_ = -310.545187 a.u.**

**E_gas_ = -310.66962162 a.u.**

C 1.92985500 0.00017200 -0.59153600

H 2.20034900 -0.88296600 -1.19484100

C 0.43835500 0.00007100 -0.33686700

C -0.26718700 -1.20482000 -0.18912300

C -0.26732100 1.20489700 -0.18900900

C -1.63563800 -1.20794000 0.09593200

C -1.63574400 1.20785800 0.09603600

H 0.26445000 2.15456900 -0.30296100

C -2.32542500 -0.00009200 0.24011400

H -2.16676700 2.15762900 0.20278900

H -3.39608000 -0.00011500 0.46022100

C 2.75071500 -0.00012700 0.70734900

H 2.52061800 0.88789100 1.31791900

H 2.52072100 -0.88847100 1.31747600

H 3.83261300 0.00000000 0.49701400

H -2.16651500 -2.15780100 0.20259300

H 0.26462800 -2.15445400 -0.30316100

H 2.20033000 0.88359800 -1.19441600

**
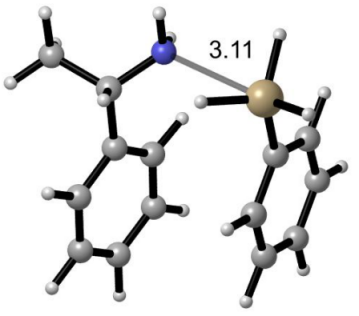
**

**TS1c**

**G_gas_ = -888.455887 a.u.**

**E_gas_ = -888.701584194 a.u.**

Si -1.83218200 -2.38605000 0.81157600

H -2.97229600 -2.72054200 1.72846300

C -1.98446200 -0.57877700 0.30691800

C -2.46088100 -0.21628900 -0.96556400

C -1.66494600 0.45085900 1.21127800

C -2.62520900 1.12696300 -1.31923700

H -2.70616000 -0.99273100 -1.69665400

C -1.82498300 1.79386300 0.86245300

H -1.27311800 0.20711600 2.20363700

C -2.31102000 2.13493500 -0.40324300

H -2.99911800 1.38728500 -2.31345600

H -1.55639600 2.57718900 1.57552300

H -2.43397900 3.18575500 -0.67876500

N 0.75990800 -2.18129600 -0.88987500

H 0.83953900 -1.91021800 -1.87125900

H 0.84429800 -3.19784400 -0.87679100

C 1.83941000 -1.58786500 -0.09925900

H 1.71532900 -1.97553900 0.92723900

C 3.24513900 -1.98902100 -0.58997900

H 3.41169700 -1.61427700 -1.61388900

H 4.03477000 -1.56896200 0.05279200

H 3.35596100 -3.08647100 -0.59910700

C 1.72359200 -0.07117800 -0.01759800

C 2.32067400 0.60349800 1.05962000

C 1.08838000 0.68964300 -1.00755200

C 2.29505400 1.99730500 1.14144100

C 1.06594400 2.08520000 -0.93308200

H 0.57043100 0.19904700 -1.83278900

C 1.67083000 2.74577300 0.13825200

H 0.55168100 2.65615400 -1.70948400

H 1.64593600 3.83682000 0.19889100

H -1.94556000 -3.29140800 -0.36537600

H -0.58891200 -2.65287600 1.58961200

H 2.81052800 0.02519400 1.84918800

H 2.76143700 2.50064100 1.99259900

**
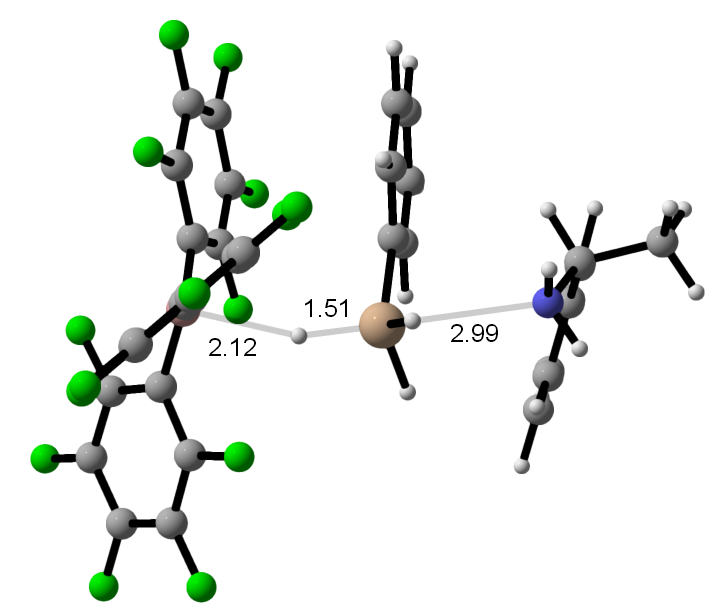
**

**TS2c**

**G_gas_ = -3095.012454 a.u.**

**E_gas_ = -3095.37746447 a.u.**

Si 1.07303900 -0.11524100 -1.54470700

H -0.25814300 0.18068000 -0.89094300

C 2.18375700 -0.78272500 -0.18354400

C 3.07891400 0.07606900 0.48029200

C 2.12869600 -2.13024800 0.22435400

C 3.89310900 -0.39264700 1.51521100

H 3.15488100 1.12433300 0.18306300

C 2.93531800 -2.59899000 1.26511500

H 1.44396700 -2.82383900 -0.26786600

C 3.81853300 -1.72928000 1.91573000

H 4.58089700 0.29389100 2.01331000

H 2.87003500 -3.64542200 1.57511500

H 4.44124200 -2.09308900 2.73681300

B -1.90141000 0.03086400 0.44623700

C -2.22434700 1.56775200 0.27046500

C -2.35337500 2.15374400 -0.99727200

C -2.41899700 2.42333300 1.36352100

C -2.63093700 3.50762600 -1.18243900

C -2.71389100 3.77967300 1.22045700

C -2.81831900 4.32308800 -0.06261900

C -2.68344200 -1.00348200 -0.46005300

C -4.00528200 -0.77802500 -0.87518600

C -2.10005900 -2.19035100 -0.92638900

C -4.70360100 -1.66090600 -1.70177500

C -2.75394100 -3.08878500 -1.76617100

C -4.07072400 -2.82144700 -2.15239500

C -0.97106800 -0.45225600 1.62008700

C -1.11291600 -1.69926100 2.25296600

C 0.10078000 0.33475100 2.07815300

C -0.24732300 -2.14916900 3.24976400

C 0.97004500 -0.06885700 3.08791700

C 0.80720100 -1.33081600 3.66031900

N 3.55817400 -0.91334400 -3.00912800

H 3.66345800 -0.50562600 -3.94060700

H 3.23308800 -1.86849300 -3.15861400

C 4.84481600 -0.93731100 -2.30694800

C 5.27776200 0.45702000 -1.87174500

C 6.30733000 0.58897900 -0.92496600

C 6.72721200 1.84526700 -0.48676000

H 6.76964300 -0.30948300 -0.50631200

C 6.12361400 3.00290200 -0.99231300

H 7.52323100 1.92290700 0.25853000

H 6.44699600 3.98853800 -0.64828100

H 1.54660900 1.18843500 -2.07115100

H 0.74189700 -1.09774200 -2.60859100

F -2.89946100 4.55402600 2.28550500

F -3.09406300 5.61078700 -0.21814900

F -2.72678900 4.02554700 -2.40373800

F -2.18916500 1.41250500 -2.09879900

F -2.33812600 1.95018800 2.61148300

F 0.35697700 1.51901700 1.51370400

F 1.96596900 0.71634600 3.48452700

F 1.65208900 -1.75093900 4.59085900

F -0.41074100 -3.34743700 3.80478300

F -2.11632500 -2.51846000 1.92330500

F -0.84255800 -2.49869900 -0.58264700

F -2.14586200 -4.19138400 -2.19422600

F -4.71616700 -3.66795400 -2.94204300

F -5.95872600 -1.40890600 -2.05980600

F -4.66783000 0.30978300 -0.47026800

C 5.10087200 2.88527300 -1.93559300

H 4.61658600 3.78102400 -2.33330800

C 4.68100500 1.62211800 -2.36992700

H 3.85727600 1.55103400 -3.08097700

C 5.95355100 -1.63732300 -3.11530400

H 5.65265200 -2.66605100 -3.37414400

H 6.15142500 -1.08727900 -4.05060100

H 6.89847900 -1.69068600 -2.55292200

H 4.67433500 -1.51472800 -1.38202300

**
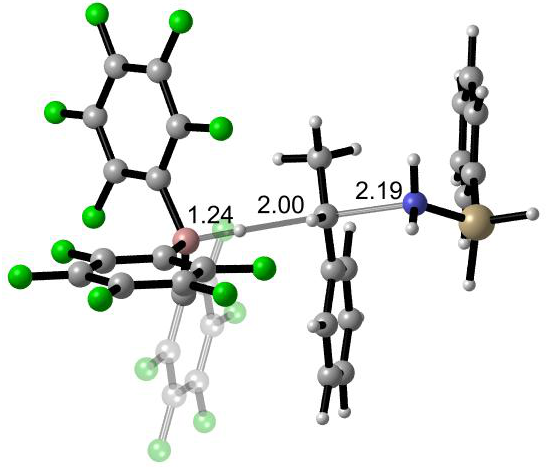
**

**TS3c**

**G_gas_ = -3094.988955 a.u.**

**E_gas_ = -3095.35273003 a.u.**

C -1.63115800 -0.24568200 1.25170400

H -0.84898700 -0.30460500 1.99888000

C -1.94072700 1.07466600 0.72568500

C -1.26666200 2.20162200 1.24576200

C -2.86183200 1.25424500 -0.32683100

C -1.51012800 3.46946300 0.72671400

C -3.11008700 2.52611400 -0.83657500

H -3.36648300 0.39496100 -0.76614200

C -2.43116800 3.63405200 -0.31606500

H -3.80472200 2.65170200 -1.66917100

H -2.60190500 4.62611700 -0.74087300

C -2.04200500 -1.50313300 0.55812400

H -3.12261100 -1.52933900 0.36257700

H -1.52927600 -1.53324200 -0.41648400

H -1.73637700 -2.39011000 1.12524200

N -2.98143000 -0.37054200 2.97716900

H -2.97155900 -1.37363600 3.17403300

Si -4.63087800 0.33777300 2.82962100

H -5.31602700 0.21184600 4.14846800

H -4.44439500 1.76554500 2.47637700

C -5.52184500 -0.60229900 1.48240700

C -5.72433100 -1.99323000 1.58697100

C -5.97282700 0.05266600 0.32049400

C -6.33806400 -2.70960500 0.55667200

H -5.39991100 -2.53525200 2.48188600

C -6.59288000 -0.66078000 -0.70985600

H -5.82681500 1.13044000 0.21030700

C -6.77050100 -2.04283300 -0.59511400

H -6.48124600 -3.78879200 0.65165700

H -6.93410500 -0.13736500 -1.60648500

H -7.24912600 -2.60208700 -1.40285800

B 1.29740800 -0.10352200 -0.11199600

C 1.04230500 1.19920200 -1.04421700

C -0.02856900 1.18781100 -1.94482300

C 1.69515400 2.43144900 -0.92985300

C -0.44637300 2.29474800 -2.67671500

C 1.31413700 3.57219800 -1.64616100

C 0.22748600 3.50678200 -2.51736400

C 2.47759000 0.03263000 1.00009100

C 3.82053100 -0.11260600 0.63978700

C 2.25058700 0.26837200 2.35246900

C 4.87422300 -0.03282400 1.55184800

C 3.26613100 0.35526500 3.30794500

C 4.59150100 0.20016600 2.90100000

C 1.39761400 -1.53658600 -0.87330400

C 1.07951800 -2.69943000 -0.16665000

C 1.77636500 -1.73148200 -2.20386500

C 1.10575900 -3.97907900 -0.72320300

C 1.82361600 -2.99292200 -2.80525400

C 1.48692300 -4.12472800 -2.05860500

F 0.98550200 0.44284500 2.82435900

F 2.98263500 0.58277400 4.59359800

F 5.58003000 0.27808300 3.79055000

F 6.13934300 -0.17379700 1.15711100

F 4.13906900 -0.31750400 -0.64619400

F 0.70564300 -2.61405200 1.13029500

F 0.77064200 -5.05180900 -0.00047600

F 1.52387100 -5.33372300 -2.61761900

F 2.18840900 -3.13003900 -4.08043300

F 2.11153400 -0.68717000 -2.97189600

F 2.72899300 2.58949400 -0.08965900

F 1.96224500 4.72729300 -1.48286000

F -0.17951600 4.59286900 -3.17553900

F -1.51872600 2.22832000 -3.47223500

F -0.75659200 0.06075600 -2.09994700

H 0.22610400 -0.18787800 0.51177200

H -2.40343700 0.07979500 3.69066100

H -0.52041400 2.05888800 2.02961500

H -0.96290700 4.33115400 1.11483200

**
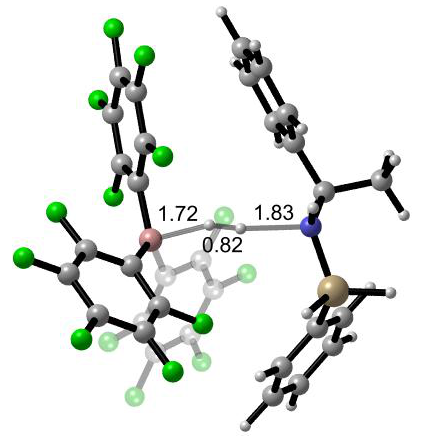
**

**TS4c**

**G_gas_ = -3095.017878 a.u.**

**E_gas_ = -3095.37632162 a.u.**

H 0.01200400 -0.33085000 -1.44914600

B -0.50717500 0.92287800 0.57205500

C 0.83681000 0.43268500 1.26096800

C 1.74367900 1.30739300 1.87345400

C 1.17376100 -0.92461300 1.32368400

C 2.89963800 0.86522900 2.52076700

C 2.31790900 -1.40709000 1.95131500

C 3.17973500 -0.50001000 2.56885100

C -1.87047900 0.18536900 0.91534500

C -2.93474200 0.17270300 0.00158700

C -2.08864300 -0.51533600 2.10925200

C -4.13676200 -0.48569500 0.24047300

C -3.27609800 -1.19920100 2.38055200

C -4.30158000 -1.18766500 1.43518900

C -0.54291900 2.37550200 -0.07231600

C 0.47504600 2.80939300 -0.93055000

C -1.56634400 3.29877500 0.17314800

C 0.48832400 4.06635800 -1.53049400

C -1.58792100 4.57336300 -0.39957000

C -0.55414800 4.95723200 -1.25667200

F 0.37049100 -1.83328600 0.75377700

F 2.59982200 -2.70806700 1.95383100

F 4.28672900 -0.93003300 3.16304700

F 3.74905900 1.73287100 3.06778600

F 1.52282300 2.62712800 1.87719600

F -1.14898100 -0.55184400 3.06111100

F -3.43089100 -1.86947100 3.52024500

F -5.41982500 -1.87131100 1.65173400

F -5.10334000 -0.50178300 -0.67513000

F -2.80354200 0.78664400 -1.18052700

F 1.49570500 1.98630900 -1.22022400

F -2.57554000 2.99010600 0.99407400

F -2.57665100 5.42199300 -0.13322600

F -0.56061000 6.16303100 -1.81019700

F 1.47439000 4.42434600 -2.34919700

H -0.42272100 0.17453800 -0.97066100

N 0.68241300 -1.44229200 -2.73701100

H 0.01032800 -1.15898800 -3.45136600

Si 2.31512700 -0.86893200 -3.14051400

H 2.14771300 0.56433200 -3.49368400

H 2.86981300 -1.60582100 -4.31814300

C 0.38475200 -2.79948600 -2.23645900

H 1.00559600 -2.93686400 -1.33752500

C 3.48846200 -1.08179700 -1.69484900

C 4.00915700 0.03851200 -1.01760900

C 3.93549400 -2.36096400 -1.30625600

C 4.95561700 -0.11578100 -0.00023700

H 3.67151600 1.04250600 -1.28312400

C 4.86813500 -2.51751600 -0.27800600

H 3.56022300 -3.25370600 -1.81605600

C 5.38845200 -1.39280300 0.36925400

H 5.34262500 0.76338800 0.52164000

H 5.19194100 -3.51825100 0.01777000

H 6.11472500 -1.51173600 1.17594400

C -1.07237700 -2.93851100 -1.80676100

C -1.39311200 -3.64828200 -0.64020100

C -2.12352300 -2.43685500 -2.58951400

C -2.72354200 -3.84540800 -0.26205200

C -3.45590700 -2.62841200 -2.21447800

H -1.91559600 -1.88217300 -3.50911400

C -3.76072100 -3.33596500 -1.04835600

H -4.25834200 -2.20784500 -2.82417300

H -4.80050500 -3.46067500 -0.73740400

C 0.73908700 -3.90414800 -3.24921100

H 0.51785500 -4.89731800 -2.82948500

H 0.14600500 -3.78622600 -4.17060800

H 1.80584300 -3.87522300 -3.52347200

H -0.58892100 -4.02966800 -0.00691000

H -2.95108700 -4.38426300 0.66134300

**
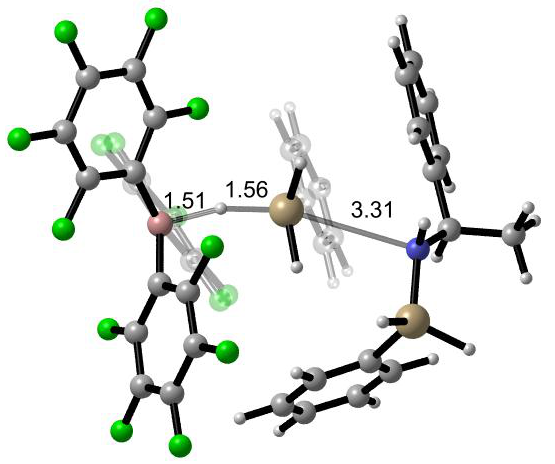
**

**TS5c**

**G_gas_ = -3616.476618 a.u.**

**E_gas_ = -3616.93170548 a.u.**

Si -0.70006100 0.39663500 -1.10543200

H 0.75693500 0.26534700 -0.55703000

C -1.57828500 1.31437200 0.25308800

C -2.51728900 0.64761300 1.06229600

C -1.23117000 2.64129300 0.56977700

C -3.09442300 1.28891300 2.15931800

H -2.78314400 -0.38814900 0.85203500

C -1.79904900 3.27690900 1.67612400

H -0.50617500 3.18168600 -0.04167600

C -2.72861600 2.60154900 2.47387500

H -3.81036500 0.75083600 2.78486500

H -1.51482300 4.30428000 1.91665200

H -3.16360400 3.09765100 3.34527900

B 1.97387200 -0.17747700 0.22541500

C 1.87228900 -1.76015500 -0.01994700

C 1.55721400 -2.26314700 -1.28579000

C 1.99359700 -2.71324100 0.99573200

C 1.28762700 -3.60847000 -1.53297900

C 1.74504000 -4.07103500 0.78992700

C 1.38677900 -4.52125500 -0.48199400

C 3.05096200 0.61582400 -0.65404100

C 4.26173500 0.04002400 -1.05236500

C 2.82898000 1.92583200 -1.09045500

C 5.19833400 0.71705700 -1.83989400

C 3.72891700 2.63577400 -1.88164600

C 4.92700400 2.02092300 -2.25857200

C 1.53096900 0.40740000 1.64855300

C 2.13327000 1.50866900 2.27239500

C 0.40911200 -0.10992900 2.31356700

C 1.64574400 2.07401800 3.45491300

C -0.10353300 0.41415500 3.49590800

C 0.51576500 1.52810400 4.06511000

H -1.07222200 -1.01604300 -1.24399700

H -0.49066300 1.12930800 -2.37387300

F 1.79638000 -4.93407700 1.80317900

F 1.09521400 -5.80187300 -0.67887500

F 0.89555600 -4.01890000 -2.73788500

F 1.42707000 -1.41794500 -2.32425000

F 2.31565000 -2.34011400 2.23875000

F -0.25974700 -1.14733700 1.78330000

F -1.18223600 -0.11640200 4.06761700

F 0.03166500 2.06311100 5.17953700

F 2.24818900 3.12844500 4.00018400

F 3.22498800 2.08161600 1.75406800

F 1.69213900 2.55531800 -0.74068400

F 3.46505900 3.87932600 -2.27602200

F 5.80437000 2.67760500 -3.00825100

F 6.34109800 0.13280400 -2.18999500

F 4.57986200 -1.20258100 -0.67058800

N -3.59521400 -0.36184000 -2.51803100

H -3.33443700 -0.07197500 -3.45983900

Si -3.69354000 -2.11885300 -2.38229300

H -2.62047200 -2.63942200 -3.27346300

H -5.02088700 -2.65174400 -2.82494900

C -4.57747900 0.55409200 -1.92401400

H -4.72883100 0.21813500 -0.88370000

C -3.38515200 -2.68680000 -0.61675600

C -2.17543700 -3.31854500 -0.26596300

C -4.32642600 -2.45008100 0.40458000

C -1.89856700 -3.66669200 1.05923400

H -1.43424500 -3.53937900 -1.03891600

C -4.04894400 -2.78539600 1.73362900

H -5.29059600 -1.98885300 0.16768900

C -2.82885100 -3.38427200 2.06357900

H -0.95276900 -4.15053900 1.31206100

H -4.78589600 -2.57873800 2.51414700

H -2.60399600 -3.63500100 3.10301600

C -4.07202600 1.99104900 -1.84362600

C -4.53291100 2.82252400 -0.81145600

C -3.19960800 2.53619400 -2.79491800

C -4.12486000 4.15339500 -0.72272300

C -2.78631400 3.87033200 -2.71081900

H -2.81600800 1.92022900 -3.61150100

C -3.24623700 4.68386200 -1.67327100

H -2.09780000 4.27156200 -3.45896100

H -2.91785100 5.72365600 -1.60126500

C -5.94239600 0.52382000 -2.64162100

H -6.65957400 1.20241900 -2.15464700

H -5.82617200 0.85184100 -3.68764700

H -6.36973000 -0.49126200 -2.64347300

H -5.20097600 2.41171100 -0.04969400

H -4.48006600 4.77471200 0.10287800

**
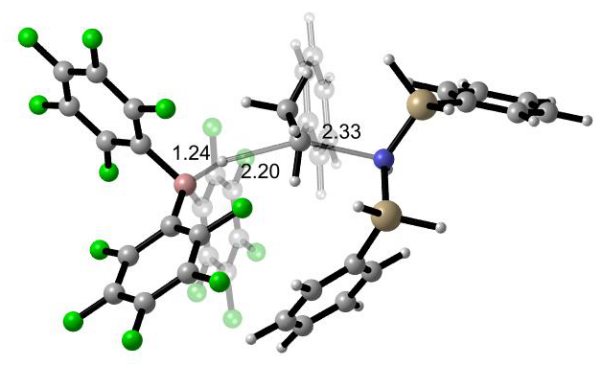
**

**TS6c**

**G_gas_ = -3616.454693 a.u.**

**E_gas_ = -3616.90881542 a.u.**

Si 4.65433500 0.23493400 -2.56922100

H 3.98354100 -0.88476500 -3.28423300

C 6.03957900 -0.41211200 -1.49209300

C 5.79068600 -1.50152700 -0.63202200

C 7.30939800 0.19388200 -1.45847000

C 6.78014600 -1.96719200 0.23705900

H 4.81505400 -1.99664600 -0.63535800

C 8.30239100 -0.27514200 -0.59251900

H 7.53058400 1.04163500 -2.11388100

C 8.03802300 -1.35437500 0.25649700

H 6.56999300 -2.81186300 0.89811400

H 9.28465000 0.20398400 -0.57921000

H 8.81391400 -1.71948900 0.93428600

C 1.40033100 -0.09448400 -1.41005300

H 1.08089000 0.77184200 -0.83701500

C 1.84468900 -1.23857700 -0.65134100

C 2.12362100 -1.07547600 0.72731100

C 2.02465400 -2.50949900 -1.24201400

C 2.58855800 -2.14559300 1.48271900

C 2.45324100 -3.58550400 -0.47044500

H 1.80007000 -2.65796800 -2.29884800

C 2.73686900 -3.40485900 0.88967500

H 2.56319600 -4.57208200 -0.92542300

H 3.06470200 -4.25293900 1.49511000

C 1.06996600 -0.11216200 -2.85430900

H 1.74288700 -0.75362800 -3.43716900

H 0.04554000 -0.50517800 -2.95791600

H 1.05740600 0.90470800 -3.26386700

N 3.45460700 1.01158900 -1.48698500

H 3.63569200 0.67531900 -0.53840100

H 5.12467100 1.25425700 -3.54514600

Si 3.14127400 2.78165700 -1.39894200

H 4.44762100 3.49884500 -1.47102900

H 2.29968400 3.15396600 -2.56471600

C 2.24637900 3.02815000 0.22370900

C 2.81471300 2.54100400 1.41970600

C 0.95159100 3.57907800 0.27401800

C 2.09584500 2.56608600 2.61752300

H 3.82856100 2.12420800 1.42374000

C 0.23358500 3.60551400 1.47284500

H 0.48030100 3.95827500 -0.63480900

C 0.79783900 3.08813700 2.64197900

H 2.54160400 2.16922700 3.53334000

H -0.78561400 3.99573600 1.48673700

H 0.21681700 3.06451600 3.56544400

B -1.75015400 -0.22703600 -0.12013700

C -1.27615200 -0.49253700 1.41842300

C -0.68932700 -1.72302400 1.74003900

C -1.30674100 0.42460800 2.47356400

C -0.17375600 -2.03878900 2.99625000

C -0.78073000 0.16126500 3.74297200

C -0.19783900 -1.07747200 4.00669600

C -2.39179700 1.24668200 -0.40391800

C -3.63726300 1.60120200 0.12467700

C -1.77826900 2.23255900 -1.16977700

C -4.22373600 2.85506200 -0.05356200

C -2.31377800 3.50747800 -1.36823300

C -3.54921300 3.82327600 -0.80482800

C -2.74232200 -1.37164100 -0.73387200

C -2.83308500 -1.52933000 -2.11828700

C -3.57056500 -2.21585000 0.01116800

C -3.65805800 -2.46300300 -2.74694300

C -4.41538400 -3.16740100 -0.56967700

C -4.46113300 -3.29055100 -1.95933700

F -0.57698800 2.01209900 -1.75623600

F -1.63554300 4.42694000 -2.06832000

F -4.08187200 5.03153900 -0.98022500

F -5.40600800 3.14664600 0.48867700

F -4.30160000 0.71967800 0.88420100

F -2.09085900 -0.74388400 -2.93349300

F -3.69078000 -2.56939200 -4.07784900

F -5.26001000 -4.19184800 -2.52981500

F -5.18066300 -3.95430100 0.18823700

F -3.58587900 -2.14986300 1.35005300

F -1.84928400 1.64497800 2.32213500

F -0.80928600 1.09808800 4.69799700

F 0.33502800 -1.33871500 5.20097500

F 0.41373800 -3.22086500 3.21742900

F -0.57223000 -2.67263500 0.79543200

H -0.69648800 -0.31756900 -0.77011200

H 1.96403800 -0.10057200 1.19418500

H 2.79306200 -2.01554100 2.54647700

**
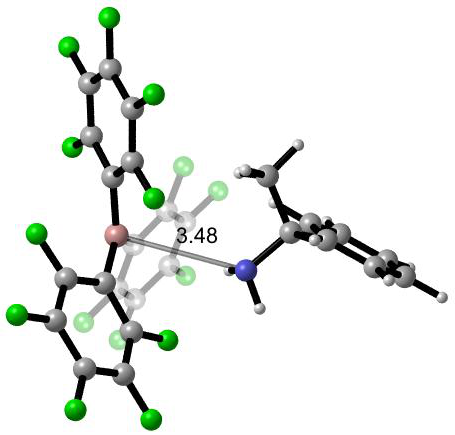
**

**TS10c**

**G_gas_ = -2572.387304 a.u.**

**E_gas_ = -2572.64562924 a.u.**

C 1.61171300 0.16871200 2.92532500

H 1.38889100 -0.07645900 3.97744000

N 0.92103200 -0.83474900 2.09337800

H 1.28463700 -0.78105700 1.14068200

C -1.68570400 -1.32928200 -0.29556100

C -1.04422000 -2.51318200 0.10823700

C -3.08884400 -1.34093200 -0.20145400

C -1.73022300 -3.62039300 0.60144500

C -3.81316300 -2.43761900 0.26614000

C -3.12570600 -3.58217000 0.67703800

C 0.45122800 -0.14579300 -1.54299000

C 1.46998200 0.81973600 -1.42414300

C 0.75788400 -1.23700900 -2.37853400

C 2.73008900 0.67399100 -2.00210600

C 1.99467600 -1.39936700 -3.00216500

C 2.99432000 -0.44386300 -2.79673300

C -1.48509700 1.37160700 -0.39238200

C -1.44339800 2.44623800 -1.29372600

C -2.03596200 1.64712000 0.86860300

C -1.90187300 3.72270400 -0.97351900

C -2.47366400 2.91842700 1.23765900

C -2.41470200 3.95866700 0.30623900

F -1.07471000 -4.70817400 0.99429500

F 0.28693300 -2.62506500 0.04802800

F -3.79365100 -4.63077200 1.13176700

F -5.14008300 -2.40327100 0.32557000

F -3.80033600 -0.27671400 -0.58256000

F -2.12047900 0.69038500 1.79351600

F -2.94112600 3.14634100 2.46079300

F -2.84140800 5.16853100 0.63526900

F -1.85298600 4.71087800 -1.86103700

F -0.94709500 2.26593700 -2.52227900

F 1.29035100 1.93166200 -0.69969600

F 3.68087500 1.57956000 -1.78929400

F 4.18138500 -0.59186100 -3.36043100

F 2.23283100 -2.44873400 -3.77976000

F -0.15596800 -2.17747400 -2.61820300

B -0.90857600 -0.04374400 -0.75532300

C 3.12676000 0.11399000 2.73864300

C 3.94839200 -0.51601200 3.68416800

C 3.72672500 0.61753200 1.57030500

C 5.32513300 -0.64778000 3.47008900

C 5.09850000 0.48349100 1.34803000

H 3.12209900 1.13094900 0.82096500

C 5.90488800 -0.15215000 2.29897200

H 5.53455500 0.88110900 0.42767900

H 6.97944200 -0.25683500 2.12891500

C 1.02085000 1.54231400 2.59423100

H -0.04411200 1.57029200 2.86069100

H 1.10974300 1.67067600 1.50847700

H 1.55612700 2.35931900 3.09952000

H 3.50346800 -0.91161900 4.60257400

H 5.94672300 -1.14095500 4.22225800

H 1.16177100 -1.77836200 2.39907600

**
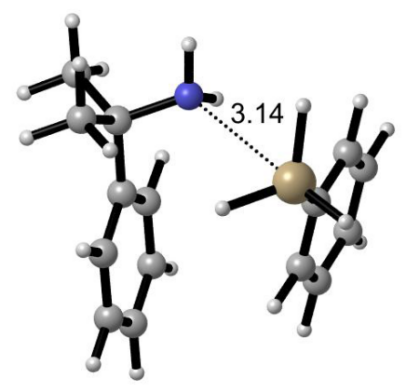
**

**Int1d**

**G_gas_ = -927.722210 a.u.**

**E_gas_ = -927.991976120 a.u.**

Si 1.01345500 -2.24815500 -1.52126700

H 1.92668200 -2.59766900 -2.66077200

C 1.86949800 -0.94108100 -0.46507600

C 2.41592000 -1.25682500 0.79337200

C 1.99087300 0.38279100 -0.92270800

C 3.05895200 -0.28569400 1.56770700

H 2.33473500 -2.27739300 1.18080600

C 2.63713600 1.35603200 -0.15552800

H 1.55617400 0.67324100 -1.88370400

C 3.17278300 1.02500800 1.09231000

H 3.47529100 -0.55281800 2.54309700

H 2.70774400 2.38088200 -0.52823900

H 3.67499600 1.78649600 1.69509300

N -0.95667100 -1.67644900 0.84995300

H -0.27484000 -1.22449200 1.45876500

H -1.23216800 -2.54889500 1.30400100

C -2.13735100 -0.81613200 0.63585000

C -2.94759000 -0.60335500 1.93739100

H -2.32974900 -0.16310900 2.73417900

H -3.80952800 0.06230100 1.77033700

H -3.32786900 -1.57065000 2.30604200

C -1.62444200 0.53949000 0.11371700

C -1.83490300 0.97637600 -1.20253400

C -0.89553500 1.38255400 0.97108000

C -1.34327700 2.21036300 -1.64322100

C -0.40438900 2.61446500 0.53781300

H -0.69243400 1.07482500 1.99948700

C -0.62942700 3.03804800 -0.77480200

H 0.16608500 3.24092400 1.22778800

H -0.24666500 4.00243600 -1.11852600

H 0.78559500 -3.49966600 -0.74851700

H -0.24029900 -1.73425200 -2.13430700

H -2.38542500 0.35429600 -1.90779900

H -1.52375400 2.52371700 -2.67499900

C -3.03749400 -1.54024000 -0.37631900

H -3.93675400 -0.95114800 -0.61095200

H -2.49549900 -1.75860400 -1.30610700

H -3.36851800 -2.50192800 0.04808600

**
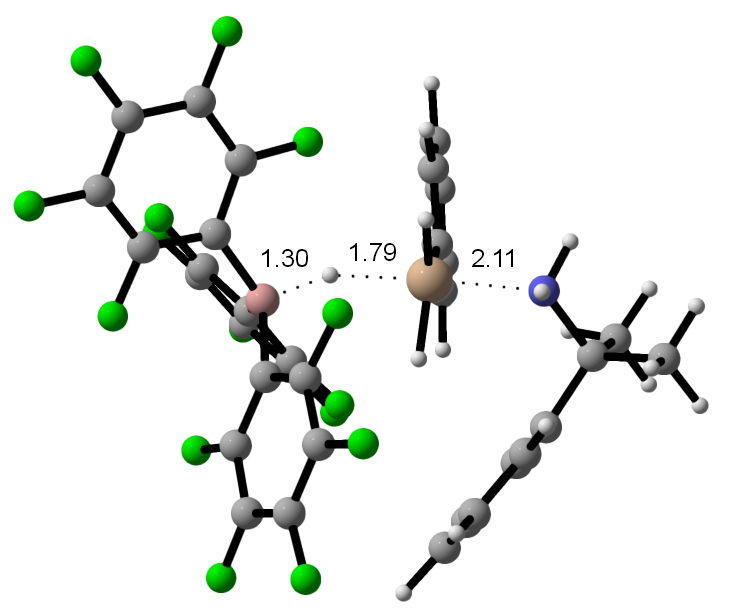
**

**Int2d**

**G_gas_ = -3134.285278 a.u.**

**E_gas_ =-3134.68239186 a.u.**

Si 1.11786900 0.48210900 1.69888200

H -0.37918500 0.11887200 0.79666900

C 0.89197700 2.31939300 1.44366500

C 1.47566700 3.01959100 0.37003500

C 0.02974600 3.02029600 2.30936100

C 1.21467600 4.37736500 0.17171700

H 2.11207000 2.49554200 -0.34480500

C -0.22454200 4.38168300 2.11756600

H -0.47496600 2.49420000 3.12373000

C 0.36668000 5.06182400 1.04833300

H 1.65430500 4.89092600 -0.68684900

H -0.90101500 4.90848900 2.79533500

H 0.15467400 6.12215700 0.88908100

B -1.10513200 -0.37160400 -0.15961800

C -0.26566100 -1.66216500 -0.69142000

C 0.31224600 -2.54082900 0.22827100

C 0.01247600 -1.94460600 -2.03229600

C 1.16736800 -3.58399500 -0.12321800

C 0.86991900 -2.97351300 -2.43298100

C 1.45440100 -3.79464000 -1.46962700

C -2.47572700 -0.78306500 0.59552200

C -3.36518200 -1.69850100 0.02867900

C -2.86052600 -0.24967000 1.82518800

C -4.55888700 -2.08448100 0.64241100

C -4.04185200 -0.60555400 2.47917000

C -4.89636900 -1.53368300 1.88110900

C -1.19742600 0.89581900 -1.17301400

C -2.35054700 1.65010300 -1.41437600

C -0.04007000 1.38892500 -1.78233700

C -2.35263100 2.82043700 -2.18426100

C 0.01131800 2.55457700 -2.53946600

C -1.16126200 3.28260400 -2.74307700

N 2.89430300 0.48630700 2.83971900

H 2.84065200 -0.44596900 3.26288800

H 2.77158100 1.13954000 3.62107100

C 4.29581700 0.66107600 2.26232400

C 5.31516300 0.14308300 3.29261200

H 5.16874600 -0.92132200 3.52870000

H 6.33500100 0.25759200 2.89701900

H 5.24525000 0.71506000 4.23207200

C 4.34533700 -0.15581600 0.96203800

C 4.34476200 0.45426300 -0.30041100

C 4.22118100 -0.30708300 -1.46770000

H 4.40634400 1.53825300 -0.39341700

C 4.10886800 -1.69590000 -1.39444100

H 4.18568800 0.19700500 -2.43594300

H 3.97562700 -2.29267400 -2.29982800

H 1.78137800 -0.35802000 0.70312900

H 0.43712300 -0.05203900 2.89622600

F 1.16563000 -3.15709200 -3.72047400

F 2.34310300 -4.72482100 -1.82569300

F 1.76919400 -4.32811500 0.81125000

F 0.11368000 -2.35190800 1.55045800

F -0.51258700 -1.19654300 -3.00938800

F 1.13624200 0.74991200 -1.59598000

F 1.17720700 3.00927000 -3.01269100

F -1.13786400 4.41167300 -3.44826900

F -3.48063400 3.50400400 -2.37503500

F -3.53301300 1.29042900 -0.89765600

F -2.08730100 0.67010400 2.43954400

F -4.36103200 -0.06965400 3.65906600

F -6.02866400 -1.88763800 2.48570300

F -5.37713100 -2.96207200 0.06212200

F -3.09655300 -2.23028400 -1.17357200

C 4.14065100 -2.32158600 -0.14413400

H 4.03817800 -3.40586800 -0.07195300

C 4.25974900 -1.55870300 1.01812400

H 4.24240500 -2.07861700 1.97923500

C 4.52463800 2.16231100 2.05769600

H 4.49676300 2.68607800 3.02662600

H 3.76563000 2.61843000 1.41251900

H 5.51488500 2.33397600 1.61336700

**
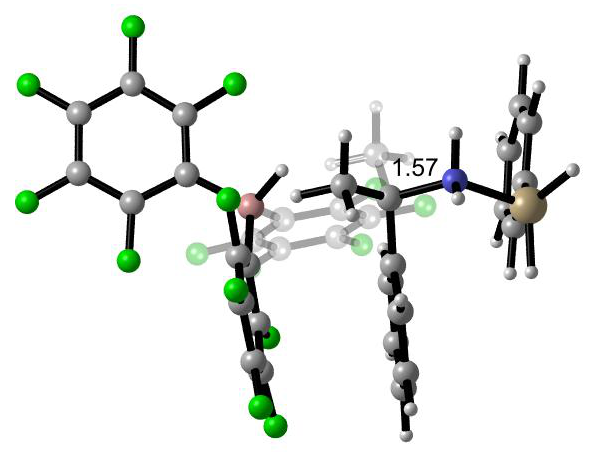
**

**Int3d**

**G_gas_ = -3134.270366 a.u.**

**E_gas_ = -3134.66644808 a.u.**

C 2.96262700 1.38535300 -1.76885300

C 3.11604400 1.49843000 -0.25088300

C 3.88509200 2.53834000 0.30507700

C 2.52881900 0.56376200 0.61387100

C 4.07626800 2.63229900 1.68403500

C 2.71703300 0.66288000 1.99627000

H 1.89836000 -0.23606700 0.23057000

C 3.49349200 1.68898700 2.53628800

H 2.21139500 -0.04764300 2.65397100

H 3.61048600 1.77721700 3.61827700

C 2.00607900 0.28738800 -2.22922500

H 2.26378600 -0.70525500 -1.84504000

H 0.98801700 0.51570500 -1.88439100

H 1.97814100 0.24246500 -3.33017000

N 4.38808100 1.02503300 -2.32212600

H 4.27850200 0.79456900 -3.31814000

Si 5.49773300 -0.29931000 -1.49963500

H 6.56830400 -0.31713700 -2.53164800

H 5.93110400 0.34648300 -0.24509000

C 4.58224700 -1.88281500 -1.31101700

C 4.26384600 -2.67226900 -2.43570800

C 4.11256800 -2.27822400 -0.04164700

C 3.46049400 -3.80495900 -2.29697300

H 4.63046900 -2.39871200 -3.43059600

C 3.31446400 -3.41295700 0.09242300

H 4.34251900 -1.67769400 0.84144400

C 2.97462700 -4.16553400 -1.03552800

H 3.20156400 -4.40121000 -3.17505000

H 2.91730000 -3.69659400 1.06743700

H 2.31125200 -5.02499600 -0.92433600

B -1.43246000 -0.00704100 -0.22423100

C -0.77437600 1.23349700 0.62120600

C -0.50589700 1.26500900 1.99153900

C -0.32774800 2.35680800 -0.07920400

C 0.19077600 2.30357100 2.61681100

C 0.38132800 3.41028800 0.49670800

C 0.65469600 3.37836400 1.86242900

C -2.90189700 0.34746300 -0.83978800

C -3.79723600 1.27502500 -0.30315000

C -3.33823800 -0.30304100 -1.99767800

C -5.04026800 1.56182000 -0.87527100

C -4.57223300 -0.05288400 -2.60365000

C -5.43006000 0.89070100 -2.03537200

C -1.44560000 -1.42834300 0.58879300

C -0.42073700 -2.36264900 0.44283000

C -2.47826400 -1.81607400 1.44866800

C -0.41069400 -3.60578000 1.08059300

C -2.51482800 -3.04820700 2.10676500

C -1.46845600 -3.95400800 1.91915000

F -2.55671000 -1.22668000 -2.58469100

F -4.93897200 -0.70055600 -3.71483300

F -6.61351600 1.14783400 -2.59673500

F -5.85957300 2.46203000 -0.32388200

F -3.49060500 1.94296100 0.82134800

F 0.65428000 -2.08945200 -0.33917500

F 0.60545400 -4.47238500 0.90641800

F -1.47530500 -5.13543500 2.53839500

F -3.52480000 -3.36686500 2.91777300

F -3.49240000 -0.97824900 1.69686400

F -0.51726300 2.43203400 -1.41660900

F 0.86569300 4.40992200 -0.25738300

F 1.40175800 4.33034500 2.42745700

F 0.50952500 2.22871800 3.91353400

F -0.85002500 0.24001000 2.78911900

H -0.69745600 -0.17451100 -1.18497600

H 4.94226000 1.89100900 -2.31488400

H 4.33296600 3.30324800 -0.33479500

H 4.66084700 3.45776500 2.09581200

C 2.58064600 2.71377600 -2.43398200

H 1.57801200 3.00783500 -2.10375800

H 3.26758400 3.53324700 -2.17460200

H 2.55650400 2.60639000 -3.53070100

**
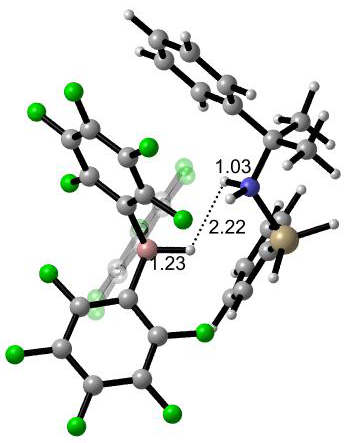
**

**Int4d**

**G_gas_ = -3134.300934 a.u.**

**E_gas_ = -3134.70100486 a.u.**

H -1.44867800 -1.28330600 -0.87948200

B 0.74782800 0.50329600 -0.02922200

C 1.35255900 -0.68100700 0.92353600

C 2.64717300 -0.75157100 1.44754000

C 0.57009300 -1.80094400 1.19711900

C 3.12644100 -1.85418700 2.16588400

C 0.98799900 -2.92109900 1.90594800

C 2.29409400 -2.94931700 2.39377600

C -0.58783700 1.22757200 0.59178400

C -1.51303800 1.84197700 -0.25109400

C -0.88554600 1.33690600 1.95438600

C -2.65074800 2.52252800 0.17744800

C -2.01572200 2.00176900 2.44037100

C -2.90103500 2.60371600 1.54586100

C 1.81334100 1.64712000 -0.49151400

C 2.36409100 1.68848200 -1.77166200

C 2.20978600 2.68254800 0.36004400

C 3.24837100 2.68268200 -2.20150200

C 3.08861200 3.69813100 -0.01889400

C 3.60964900 3.69824500 -1.31590800

F -0.71296400 -1.85877200 0.72177800

F 0.18196400 -3.97314100 2.07314900

F 2.75003200 -4.02699600 3.03249500

F 4.38178900 -1.87732400 2.61438900

F 3.52296800 0.24527900 1.26259700

F -0.07924300 0.79005300 2.87168500

F -2.27125200 2.04932300 3.74809300

F -4.00118500 3.20998900 1.99205100

F -3.50958500 3.05275200 -0.69702200

F -1.35205900 1.75886400 -1.60720300

F 2.06514500 0.73269300 -2.68225400

F 1.75500100 2.71249300 1.62216300

F 3.43790000 4.66130600 0.83455300

F 4.44968400 4.65773900 -1.70120600

F 3.74452300 2.67090500 -3.44117600

H 0.40085300 -0.07272300 -1.05670800

N -1.78343000 -1.06949000 -1.83235000

H -1.68571700 -0.04348600 -1.88392700

Si -0.44961900 -1.77368800 -2.99611600

H 0.15731200 -0.58209100 -3.61612500

H -1.24563200 -2.55357000 -3.97496000

C -3.27601900 -1.41293100 -1.94728700

C 0.70027900 -2.85619900 -2.02611700

C 2.03065200 -2.45548900 -1.79233800

C 0.26163800 -4.09367500 -1.51291900

C 2.89763500 -3.27627500 -1.06745400

H 2.38177400 -1.48951100 -2.15907300

C 1.12483200 -4.90333000 -0.77341600

H -0.76578000 -4.43071400 -1.67980100

C 2.44457800 -4.49394500 -0.55196800

H 3.92341700 -2.94998900 -0.88174200

H 0.76703400 -5.84782600 -0.35810800

H 3.11518600 -5.11797300 0.04385900

C -3.99833500 -0.70848100 -0.78355800

C -3.71138400 -1.07921000 0.54380700

C -4.95898400 0.29135100 -0.99647700

C -4.36042100 -0.46900400 1.61693700

C -5.61849400 0.89490500 0.07923600

H -5.20405600 0.62898900 -2.00247400

C -5.32492200 0.51697100 1.38883700

H -6.34964600 1.68270300 -0.11361500

H -5.82126800 1.00694700 2.22860900

C -3.74543700 -0.92572100 -3.32142400

H -4.81974300 -1.11941600 -3.44264400

H -3.57207100 0.15339000 -3.44946200

H -3.22130400 -1.45851600 -4.12666700

H -2.96393600 -1.84159400 0.76273700

H -4.10264500 -0.75969200 2.63792200

C -3.43856200 -2.93616400 -1.83011500

H -2.98259400 -3.45956500 -2.68345800

H -2.99007500 -3.32245200 -0.90362500

H -4.50939300 -3.18260900 -1.81308800

**
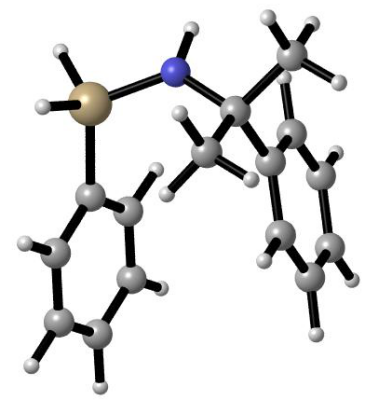
**

**Int5d**

**G_gas_ = -926.561519 a.u.**

**E_gas_ = -926.816493913 a.u.**

N 1.45181500 1.62805700 -0.72153000

H 2.00055100 1.53207300 -1.56906200

Si -0.13410800 2.32763300 -0.90501600

H -0.19395800 2.73660400 -2.33947200

H -0.33634500 3.53114200 -0.04126600

C 1.93978000 0.79535800 0.39190800

C -1.51889200 1.13127700 -0.45163600

C -1.64171600 -0.11488900 -1.09614700

C -2.41750300 1.42732200 0.58966200

C -2.62104100 -1.03272900 -0.71312400

H -0.94519000 -0.38881800 -1.89300000

C -3.40273800 0.51235300 0.97776100

H -2.34399500 2.38577300 1.11378700

C -3.50391200 -0.72046800 0.32702600

H -2.68716500 -1.99990600 -1.21778600

H -4.08949200 0.76062500 1.79165500

H -4.26805500 -1.44053000 0.63225400

C 1.42668800 -0.65830600 0.24718500

C 0.73114700 -1.33624900 1.25709800

C 1.63821200 -1.33583700 -0.96688200

C 0.25242300 -2.63687100 1.05890000

C 1.17129700 -2.63447300 -1.16864000

H 2.16080200 -0.83041500 -1.78300500

C 0.46881500 -3.29312800 -0.15284200

H 1.34953400 -3.13328000 -2.12517800

H 0.09263900 -4.30743600 -0.30852900

C 3.48114100 0.78723900 0.35311100

H 3.88841600 0.16978900 1.16820900

H 3.85404000 0.37058800 -0.59624600

H 3.86651800 1.81392300 0.45362400

H 0.53077000 -0.85128200 2.21234100

H -0.30239500 -3.13295600 1.85950800

C 1.49336700 1.43861800 1.71519200

H 1.85293400 2.47707800 1.75372000

H 0.39719000 1.44983400 1.81801500

H 1.90561300 0.89862100 2.57993600

**
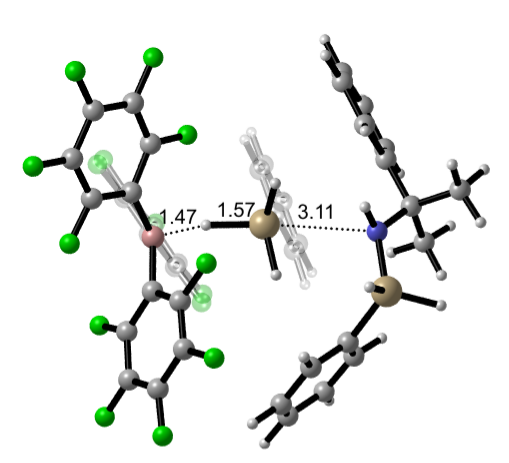
**

**Int6d**

**G_gas_ = -3655.748464 a.u.**

**E_gas_ = -3656.22961800 a.u.**

Si 0.94082800 -0.10517200 -0.71941800

H -0.59531500 -0.23115900 -0.41428400

C 1.71189300 -0.98947100 0.72197900

C 2.41622100 -0.24754100 1.69030800

C 1.49569200 -2.36176500 0.94444400

C 2.89900800 -0.86372600 2.84630900

H 2.56329100 0.82607800 1.55235300

C 1.96395300 -2.97279400 2.10954800

H 0.95586800 -2.95976500 0.20945700

C 2.66561700 -2.22628600 3.06119400

H 3.43313200 -0.27419600 3.59505200

H 1.78060100 -4.03728700 2.27476900

H 3.02119300 -2.70398800 3.97764500

B -1.96216000 -0.11873900 0.12027900

C -2.16849000 1.47084000 0.01658000

C -1.74270700 2.17747900 -1.11138200

C -2.73992200 2.23923600 1.03473800

C -1.81415100 3.56486800 -1.21625400

C -2.84153900 3.63072300 0.96796200

C -2.36599900 4.29474300 -0.16371700

C -2.71107800 -0.99496400 -0.99612900

C -3.94444400 -0.61933700 -1.53856000

C -2.16591200 -2.18003700 -1.49870300

C -4.59612800 -1.36468900 -2.52654000

C -2.77420600 -2.95075900 -2.48674400

C -4.00446300 -2.53514000 -3.00476500

C -1.68135200 -0.75875800 1.56261000

C -2.15764900 -2.00727400 1.98236000

C -0.81250100 -0.12009800 2.45945300

C -1.78148700 -2.59644400 3.19370700

C -0.41707700 -0.66683600 3.67648800

C -0.90001900 -1.92476600 4.04101400

H 1.10269300 1.35343800 -0.69042400

H 0.99181300 -0.75699400 -2.04444400

F -3.36980200 4.32621500 1.97185200

F -2.39203200 5.62458600 -0.22268200

F -1.31677400 4.20230000 -2.27765800

F -1.19113200 1.51597700 -2.14658000

F -3.20643800 1.65078900 2.14183000

F -0.27429100 1.06634800 2.13125900

F 0.43273100 -0.02283600 4.47360700

F -0.51483000 -2.48015200 5.18444000

F -2.25092100 -3.79342200 3.54017400

F -3.00770400 -2.71020200 1.22486600

F -0.99040300 -2.62538300 -1.01601900

F -2.20451200 -4.06773400 -2.93471300

F -4.60854800 -3.25397600 -3.94402500

F -5.77157000 -0.97116500 -3.00989600

F -4.56769000 0.48438500 -1.10849600

N 3.65911300 0.67013800 -2.02255200

H 3.15351200 0.26560700 -2.80749600

Si 3.65215800 2.43740000 -2.16192800

H 2.80761100 2.73427800 -3.35401200

H 5.03737400 2.95157500 -2.37583300

C 4.83461100 -0.17642100 -1.68018300

C 2.86667800 3.33174900 -0.70131200

C 1.68520100 4.07193200 -0.90351400

C 3.39469300 3.27788300 0.60376900

C 1.05348700 4.73156400 0.15642600

H 1.23054100 4.12396900 -1.89679200

C 2.75444600 3.91645200 1.66938300

H 4.31529100 2.72351600 0.80243500

C 1.58044400 4.64481400 1.44740600

H 0.14492600 5.30846600 -0.02749900

H 3.17432600 3.84981900 2.67656100

H 1.07929700 5.14599100 2.27940400

C 4.36432000 -1.63159300 -1.47614000

C 4.73984000 -2.40110700 -0.36580300

C 3.56775000 -2.25042700 -2.45649500

C 4.33146200 -3.73221400 -0.23662800

C 3.14487100 -3.57444600 -2.32655000

H 3.26656600 -1.69800300 -3.35093000

C 3.52896500 -4.32557600 -1.21172900

H 2.51656100 -4.02121200 -3.10122400

H 3.20177300 -5.36269800 -1.10496600

C 5.87072200 -0.16984900 -2.83079800

H 6.71911000 -0.83215400 -2.59793900

H 5.41532000 -0.52467600 -3.76819700

H 6.25931000 0.84681700 -2.99931300

H 5.34504700 -1.96839600 0.42979000

H 4.63124100 -4.30043300 0.64704700

C 5.49067300 0.39639100 -0.41707600

H 5.79621600 1.43997900 -0.58694700

H 4.80184900 0.36201400 0.43765900

H 6.40275000 -0.15879200 -0.15814600

**
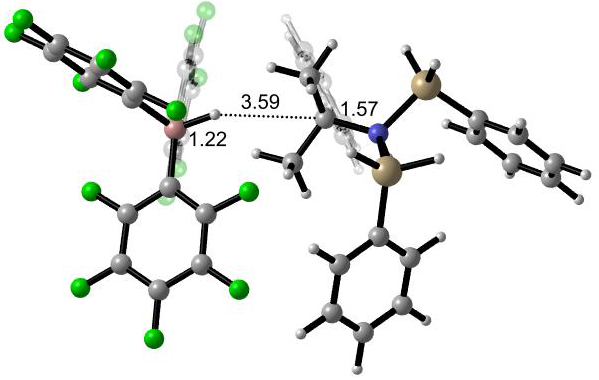
**

**Int7d**

**G_gas_ = -3655.724378 a.u.**

**E_gas_ = -3656.20821103 a.u.**

Si 4.40653400 -1.14350700 -2.27929100

H 4.08356100 -2.55584100 -1.99871500

C 6.10837300 -0.63472600 -1.71980500

C 6.60054300 -1.12451300 -0.49113600

C 6.88869500 0.29013500 -2.44077600

C 7.83235200 -0.69105100 0.00526700

H 6.01856000 -1.85063700 0.08567500

C 8.12287600 0.71977600 -1.94501300

H 6.53320200 0.68228700 -3.39777100

C 8.59258000 0.23270400 -0.72083500

H 8.20300000 -1.07848800 0.95741200

H 8.71903200 1.43662200 -2.51476600

H 9.55680100 0.57069200 -0.33300200

C 1.98083000 -0.81728000 -0.65603000

C 2.41919900 -2.03501000 0.16893500

C 3.32941300 -1.87353200 1.22983300

C 1.86349600 -3.30482300 -0.03665300

C 3.68787900 -2.94907300 2.04476400

C 2.20421800 -4.37983700 0.79061600

H 1.13069100 -3.47372800 -0.82369900

C 3.12007100 -4.20902300 1.82920200

H 1.71996300 -5.34482700 0.63163700

H 3.37708400 -5.04751200 2.48024600

C 1.13206800 -1.17197000 -1.87616700

H 1.63225000 -1.89915100 -2.53506000

H 0.17254700 -1.59813700 -1.56491000

H 0.88240400 -0.27387500 -2.45517900

N 3.28135800 -0.11019500 -1.17742100

H 3.85755300 0.02807900 -0.33678600

H 4.08759900 -0.74255600 -3.66870000

Si 3.10287500 1.67884700 -1.80031200

H 4.24657000 1.76967000 -2.74136800

H 1.80775500 1.77380600 -2.49786400

C 3.28303500 2.77904900 -0.32223000

C 4.47850300 2.78621400 0.42664300

C 2.20593600 3.58336300 0.09681200

C 4.58306900 3.56917500 1.57870500

H 5.33890100 2.18362400 0.11278400

C 2.31601200 4.37030700 1.24585000

H 1.26578400 3.58992800 -0.45684300

C 3.50116000 4.35900400 1.98821400

H 5.51034200 3.56793200 2.15708000

H 1.46392800 4.98035300 1.55343000

H 3.58477600 4.96883800 2.89135700

B -2.32110200 -0.02111600 -0.29497900

C -1.86466000 -1.25828500 0.68880700

C -1.65584400 -2.51399900 0.10551000

C -1.53336400 -1.19998100 2.04662800

C -1.16064300 -3.62454800 0.78531100

C -1.02692500 -2.28667000 2.77291500

C -0.83200200 -3.50926500 2.13521300

C -2.36063100 1.45932000 0.40633400

C -3.27137000 1.76730200 1.42278400

C -1.55020200 2.51987600 0.00549000

C -3.34603200 3.01384000 2.04690300

C -1.57996000 3.78264100 0.60463700

C -2.48144200 4.03284000 1.63660600

C -3.75798200 -0.27683300 -1.03330800

C -4.05280000 0.44189700 -2.19642500

C -4.76373600 -1.14236200 -0.59817500

C -5.25048300 0.31203900 -2.90283300

C -5.97792100 -1.30811100 -1.27317600

C -6.22333000 -0.57300900 -2.43333800

F -0.65265100 2.37635800 -1.00290200

F -0.73490300 4.75399600 0.20933500

F -2.51613100 5.22998700 2.22457900

F -4.21904800 3.24522300 3.02855500

F -4.11154900 0.82054000 1.85844300

F -3.15652700 1.31725000 -2.68615800

F -5.47735600 1.02188500 -4.01309500

F -7.37672100 -0.71481700 -3.09010600

F -6.90511500 -2.15564200 -0.81784000

F -4.60072500 -1.87688600 0.51480300

F -1.62174700 -0.04889000 2.73456700

F -0.69785700 -2.15074600 4.06010900

F -0.29450900 -4.54393100 2.78701900

F -0.90872200 -4.77162900 0.13669000

F -1.87669900 -2.68330700 -1.21416900

H -1.47562800 0.01937300 -1.17665100

H 3.75407700 -0.89005200 1.45804300

H 4.39206400 -2.79569400 2.86617300

C 1.24932800 0.16416300 0.26299900

H 0.39321200 -0.35300200 0.70581100

H 1.89375300 0.52235600 1.07800100

H 0.84496100 1.01914900 -0.28797300

**
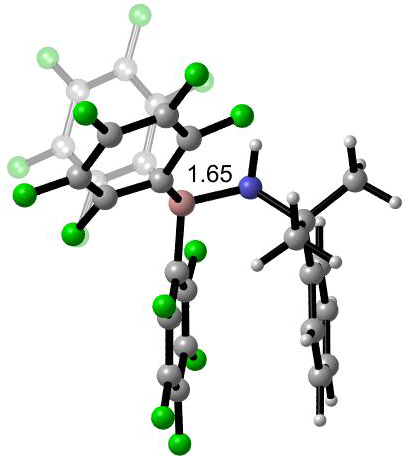
**

**int11d**

**G_gas_ = -2611.689019 a.u.**

**E_gas_ = -2611.98297692 a.u.**

C 1.25300400 -1.10861800 2.37836600

N 0.23092200 -0.09684800 1.83052000

H -0.60201100 -0.15026100 2.42097900

H 0.61631000 0.83666900 1.99874800

C -1.47603800 -1.15019600 -0.01561200

C -2.22570800 -1.83325100 0.94397700

C -1.91898200 -1.33336100 -1.33325600

C -3.28887000 -2.68907500 0.64275400

C -2.97192900 -2.17644900 -1.68840300

C -3.66465700 -2.86403300 -0.68830200

C 1.04248100 -0.09880200 -0.64039500

C 1.95163600 0.96212100 -0.57609400

C 1.45810300 -1.17520100 -1.42552400

C 3.17480200 0.98763000 -1.23784400

C 2.67425700 -1.19859400 -2.11523200

C 3.53962700 -0.11035400 -2.01767800

C -1.11895000 1.41639400 0.14000500

C -1.06984900 2.24093800 -0.99319400

C -2.01993900 1.84003500 1.12332500

C -1.83335600 3.40519700 -1.12808900

C -2.79967400 2.99423800 1.03774500

C -2.70420700 3.78690200 -0.10651000

F -3.94315500 -3.32520900 1.61264800

F -1.95234100 -1.69968400 2.26712000

F -4.67319600 -3.67008400 -1.00214100

F -3.33131700 -2.31924000 -2.96106000

F -1.32373800 -0.66330100 -2.32836800

F -2.19122600 1.09875600 2.24685400

F -3.62873400 3.33389200 2.02250000

F -3.43343900 4.89121300 -0.22139500

F -1.73580600 4.15255300 -2.22418900

F -0.27009100 1.95213800 -2.02452000

F 1.67191100 2.02294900 0.21086400

F 4.01288600 2.01253100 -1.09462900

F 4.72246400 -0.13608700 -2.62420500

F 3.03001600 -2.26736100 -2.82666600

F 0.71990600 -2.29580000 -1.51030900

B -0.31408800 -0.02781900 0.26951200

C 2.66866300 -0.62788800 2.01610300

C 3.56257200 -1.41614800 1.27712600

C 3.10830600 0.63648500 2.44647900

C 4.84007700 -0.94609900 0.95757500

C 4.37780500 1.11385400 2.12015200

H 2.45020000 1.28351300 3.03339300

C 5.25129400 0.32239100 1.36980000

H 4.68142100 2.11107400 2.44628700

H 6.24064600 0.69730000 1.09897900

C 1.07821900 -1.09858600 3.91047300

H 0.07408900 -1.45802300 4.18646800

H 1.21893800 -0.09418000 4.33685300

H 1.82214700 -1.76371900 4.37142700

H 3.26581300 -2.39895100 0.91386300

H 5.50773800 -1.57119300 0.35983200

C 0.94486300 -2.51582600 1.86013300

H 1.68099700 -3.21844500 2.27393000

H 0.97326600 -2.58565300 0.76812600

H -0.04410300 -2.83867400 2.20010600

**
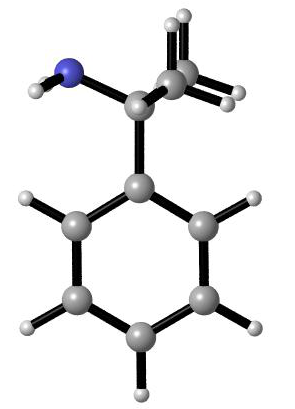
**

**1d**

**G_gas_ = -405.099864 a.u.**

**E_gas_ = -405.267567590 a.u.**

C 1.47934700 0.02987100 -0.00004800

C -0.06886400 0.02379900 -0.00002900

C -0.77993800 -1.19093700 0.00015300

C -0.81439100 1.21105100 -0.00005200

C -2.17552100 -1.21666200 0.00010400

C -2.21439800 1.19276800 -0.00007900

H -0.29974500 2.17334900 0.00000700

C -2.90263900 -0.02115000 -0.00003000

H -2.76641200 2.13662300 -0.00017200

H -3.99547500 -0.03848100 -0.00010300

C 1.99309400 -0.68947600 1.26303400

H 1.63900500 -0.17486100 2.17135900

H 1.64112000 -1.73012900 1.31188400

H 3.09348900 -0.68614600 1.26824100

N 2.08464300 1.36370600 -0.00084100

H 1.79957900 1.89825400 -0.82113600

H 1.80003300 1.89898100 0.81913800

H -2.69924500 -2.17637900 0.00021100

H -0.23636900 -2.13882100 0.00031000

C 1.99301000 -0.69111700 -1.26221800

H 3.09340700 -0.68755100 -1.26784200

H 1.63871900 -0.17778300 -2.17125900

H 1.64118900 -1.73187400 -1.30976200

**
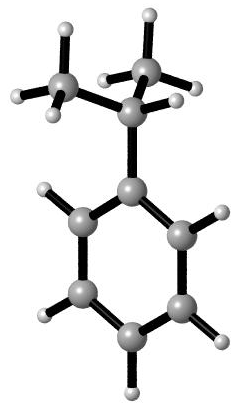
**

**1d’**

**G_gas_ = -349.806993 a.u.**

**E_gas_ = -349.958127539 a.u.**

C 1.64554300 -0.43075200 0.00003100

C 0.14060300 -0.20046200 -0.00008900

C -0.39530000 1.09908900 0.00017400

C -0.75331600 -1.28164800 -0.00042300

C -1.77607900 1.30945600 0.00010500

C -2.13734900 -1.07747300 -0.00049300

H -0.35834700 -2.30206700 -0.00063400

C -2.65451000 0.22033700 -0.00022900

H -2.81284500 -1.93722000 -0.00075800

H -3.73525500 0.38396700 -0.00028300

C 2.30824900 0.13227100 -1.26861700

H 1.85539600 -0.29300600 -2.17787600

H 2.19906800 1.22805000 -1.32157500

H 3.38663700 -0.09617000 -1.28144000

H -2.16962600 2.32959500 0.00031200

H 0.27626900 1.96200700 0.00043900

C 2.30783700 0.13132300 1.26932000

H 3.38623200 -0.09707900 1.28231200

H 1.85471300 -0.29466800 2.17811000

H 2.19857500 1.22705600 1.32307400

H 1.80511700 -1.52330600 -0.00035500

**
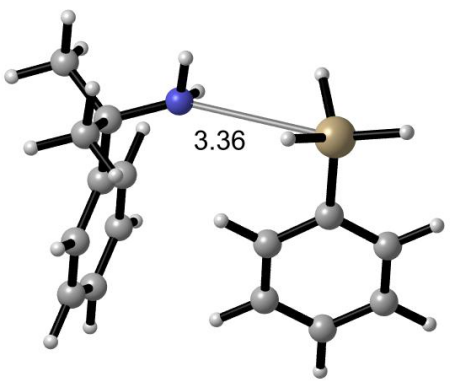
**

**TS1d**

**G_gas_ = -927.719514 a.u.**

**E_gas_ = -927.990968604 a.u.**

Si 2.38370800 -2.45395800 -0.01294400

H 3.75062200 -3.06071800 -0.05456700

C 2.59527800 -0.58190400 -0.07427200

C 3.84516900 -0.00169600 0.21808200

C 1.52584800 0.27570000 -0.39665700

C 4.01891900 1.38618800 0.19704500

H 4.70014900 -0.63956100 0.46317800

C 1.69553200 1.66279900 -0.41909100

H 0.54501400 -0.14699700 -0.61514200

C 2.94291900 2.22103200 -0.12065800

H 4.99765000 1.81618600 0.42736000

H 0.84424700 2.30282000 -0.66307000

H 3.07741500 3.30620000 -0.13667600

N -0.88272200 -1.89706600 0.52218600

H -0.68275000 -1.55167800 1.46040800

H -0.92393200 -2.91468100 0.59454600

C -2.16505300 -1.37086000 0.01187800

C -3.36811800 -1.82086900 0.87537700

H -3.25921500 -1.48745000 1.91878200

H -4.31521200 -1.41217400 0.48826900

H -3.44307300 -2.92118800 0.87746100

C -2.08087100 0.16711000 0.02679200

C -2.20902000 0.94966200 -1.12987700

C -1.85771800 0.83593900 1.24424900

C -2.11733900 2.34570600 -1.07393900

C -1.75933900 2.22646000 1.30574600

H -1.75314500 0.26521700 2.17084600

C -1.89170500 2.99154500 0.14268200

H -1.57714200 2.71461800 2.26650400

H -1.81613300 4.08091900 0.18584700

H 1.73892500 -2.92011900 1.25218700

H 1.60049300 -2.97396200 -1.16867900

H -2.37811100 0.47952500 -2.09847600

H -2.22035500 2.92832600 -1.99316800

C -2.33372000 -1.94102500 -1.40355500

H -3.27726600 -1.61454000 -1.86526700

H -1.49411200 -1.64882000 -2.05025800

H -2.35162600 -3.04177300 -1.35699000

**
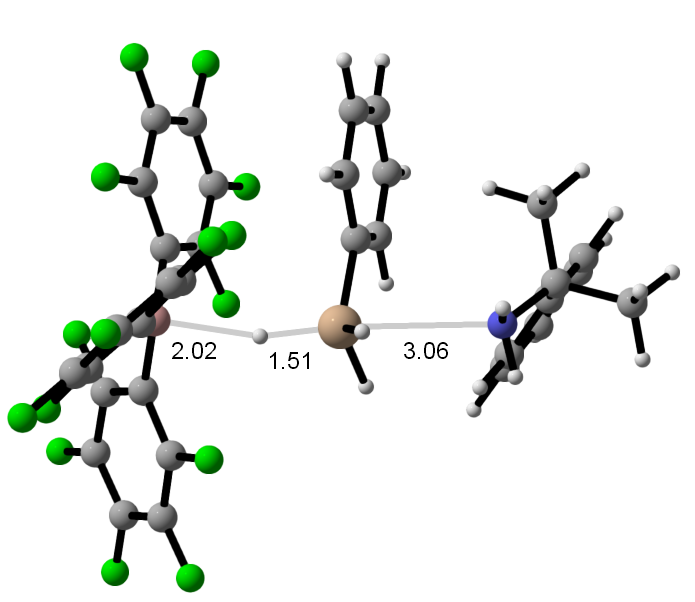
**

**TS2d**

**G_gas_ = -3134.275596 a.u.**

**E_gas_ = -3134.66687364 a.u.**

Si 0.99681700 0.06226200 1.38446300

H -0.38948500 -0.11801100 0.80204100

C 2.03606500 0.80922600 0.01306200

C 2.93971800 0.01003000 -0.71138700

C 1.90696400 2.16455000 -0.34851700

C 3.69731500 0.54624900 -1.75585200

H 3.06503200 -1.04379300 -0.45783400

C 2.65414000 2.69961000 -1.40133100

H 1.21548000 2.81351000 0.19189400

C 3.55124500 1.89089500 -2.10829500

H 4.39516900 -0.09348300 -2.30046500

H 2.53393300 3.75149200 -1.67373500

H 4.13061800 2.30830800 -2.93553900

B -2.02141300 -0.04657400 -0.39246300

C -2.28806100 -1.59851000 -0.23646900

C -2.32104800 -2.21566800 1.02189700

C -2.53098100 -2.43356300 -1.33499800

C -2.55184400 -3.58026600 1.19246300

C -2.78116100 -3.80044300 -1.20594200

C -2.79016100 -4.37518700 0.06764600

C -2.81342000 0.93736100 0.56335400

C -4.11401400 0.65412700 1.00741000

C -2.25939400 2.13221900 1.04324900

C -4.82065700 1.49254400 1.87279900

C -2.92200200 2.98813000 1.91986600

C -4.21764900 2.66465800 2.33393500

C -1.18643300 0.49471100 -1.61412400

C -1.40267900 1.75200100 -2.20311500

C -0.12138500 -0.24614900 -2.15660100

C -0.61206400 2.25300500 -3.23777100

C 0.67507200 0.20918900 -3.20339600

C 0.43984900 1.47898400 -3.73196800

N 3.58507400 0.43178300 2.97693300

H 3.51453200 -0.29838200 3.68787800

H 3.27208200 1.28872800 3.43387800

C 4.98696600 0.59917900 2.54479600

C 5.93103600 0.71332700 3.76523800

H 5.87608300 -0.19737000 4.38353900

H 6.97977900 0.84476400 3.45685500

H 5.64607500 1.57686500 4.38943500

C 5.42002300 -0.60932300 1.69850800

C 6.62006600 -0.56938900 0.96800000

C 7.03901200 -1.65868000 0.20100100

H 7.24123700 0.32862400 0.98827200

C 6.26913400 -2.82523500 0.15386500

H 7.97256600 -1.59451300 -0.36421800

H 6.59380600 -3.67842900 -0.44698400

H 1.43064300 -1.30682300 1.75658900

H 0.78338400 0.94912400 2.55691600

F -3.01451700 -4.55553300 -2.27545200

F -3.02274300 -5.67322700 0.20929800

F -2.55611000 -4.12723500 2.40499200

F -2.10472500 -1.49353500 2.12831700

F -2.54213900 -1.93009200 -2.57371800

F 0.20327600 -1.43555500 -1.63749800

F 1.67116000 -0.53272200 -3.67569900

F 1.21514600 1.94895100 -4.69864800

F -0.84439800 3.45899800 -3.74977100

F -2.40656300 2.53268700 -1.79181300

F -1.02227200 2.49079100 0.67367400

F -2.34236700 4.10215100 2.35758800

F -4.87152400 3.46960500 3.15966600

F -6.05598800 1.18712500 2.25743400

F -4.74826600 -0.44750600 0.59364800

C 5.08209700 -2.88619500 0.88663400

H 4.46772000 -3.79018800 0.86199900

C 4.66409400 -1.78966100 1.64849100

H 3.71774600 -1.85274600 2.18426000

C 5.05118900 1.89768400 1.71923800

H 4.68689000 2.74631100 2.32238200

H 4.43316600 1.82203000 0.81667900

H 6.08174900 2.13209700 1.41918600

**
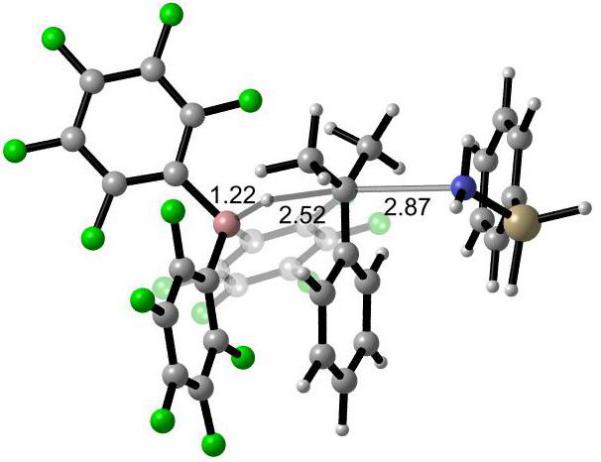
**

**TS3d**

**G_gas_ = -3134.253234 a.u.**

**E_gas_ = -3134.64070545 a.u.**

C 1.82778500 1.00660700 -1.91025900

C 1.90208500 1.67870900 -0.64888800

C 1.58531700 3.06255900 -0.53622800

C 2.22763800 0.96966500 0.53817900

C 1.62861200 3.70214900 0.69309300

C 2.24655700 1.61009000 1.76836700

H 2.41170600 -0.09786700 0.49074100

C 1.95323500 2.97702700 1.84928800

H 2.45742200 1.03919800 2.67404300

H 1.93722000 3.47498600 2.82145900

C 2.18018000 -0.41292900 -2.12065000

H 2.74531000 -0.87810700 -1.31474900

H 1.22931100 -0.96681400 -2.23655900

H 2.72519700 -0.51740100 -3.06940700

N 4.56710400 1.63271300 -2.51237300

H 4.63538900 1.18203800 -3.42466000

Si 5.76180200 1.10880900 -1.31062800

H 7.15844100 1.20485200 -1.83675300

H 5.60062500 2.02518800 -0.14880500

C 5.36220900 -0.65110100 -0.79713400

C 5.37917900 -1.70585900 -1.73132300

C 4.89857500 -0.92967800 0.50401800

C 4.89479600 -2.97362700 -1.39587500

H 5.75298300 -1.53746700 -2.74722000

C 4.40672500 -2.19396900 0.84210200

H 4.88788900 -0.13671400 1.25684200

C 4.38727000 -3.21289700 -0.11413200

H 4.89769400 -3.77356100 -2.14072900

H 4.00495700 -2.38531500 1.83936500

H 3.96562900 -4.18701600 0.14297600

B -1.11866800 -0.16142500 -0.07109700

C -1.33757000 1.37772000 0.43851400

C -1.11980300 1.87346400 1.72605300

C -1.66370700 2.36289700 -0.50211100

C -1.24582600 3.22326800 2.06930200

C -1.79828500 3.72178300 -0.21344400

C -1.58835900 4.15825400 1.09404400

C -2.34084300 -0.73831900 -0.99171200

C -3.68332800 -0.36626100 -0.88772200

C -2.06515800 -1.70663100 -1.96018900

C -4.69281200 -0.91190200 -1.68756900

C -3.03647600 -2.28100500 -2.78230100

C -4.36633500 -1.87868500 -2.64025300

C -0.79584300 -1.26172500 1.09913600

C 0.34274200 -2.06482700 1.11633700

C -1.69992800 -1.50776900 2.13815900

C 0.61561400 -3.00935900 2.11007700

C -1.48212800 -2.44500600 3.14938700

C -0.30745800 -3.20439200 3.13587300

F -0.79621500 -2.13887300 -2.14222600

F -2.71185100 -3.20064500 -3.69560400

F -5.31443900 -2.41032700 -3.41223300

F -5.96062700 -0.51842900 -1.55347900

F -4.06279600 0.56823900 -0.00331500

F 1.28367300 -1.95785900 0.14985900

F 1.75526800 -3.71863700 2.08574600

F -0.07300500 -4.10005100 4.09426400

F -2.37207100 -2.62530100 4.12569700

F -2.83334700 -0.79651100 2.20112900

F -1.84153100 2.01804000 -1.79660300

F -2.07489000 4.60428200 -1.17782800

F -1.64882000 5.45743300 1.39328400

F -0.95891900 3.63651200 3.30817700

F -0.69760200 1.07048500 2.71859800

H -0.14965700 -0.10014900 -0.80966200

H 4.53602800 2.64072300 -2.66535800

H 1.30214100 3.63936700 -1.41587900

H 1.37011500 4.75998800 0.76811000

C 1.23322100 1.70110200 -3.08844400

H 0.16563900 1.87835100 -2.86795400

H 1.69492300 2.68254400 -3.26586500

H 1.30666900 1.09664900 -4.00041000

**
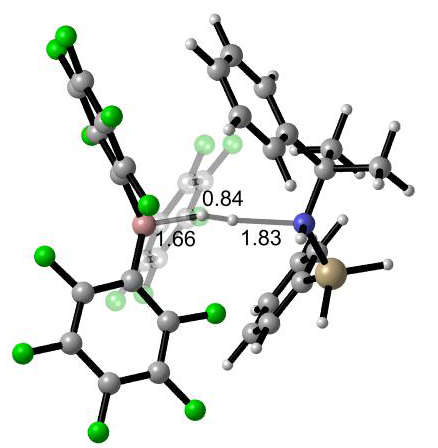
**

**TS4d**

**G_gas_ = -3134.277416 a.u.**

**E_gas_ = -3134.66346779 a.u.**

H -0.04115600 0.50692900 -1.22292400

B 0.60821500 -0.80669600 0.54672100

C -0.67445400 -0.27750700 1.32019400

C -1.66411800 -1.11605600 1.84648900

C -0.89235500 1.09476200 1.49005000

C -2.79164700 -0.62679900 2.51046900

C -2.01679900 1.62274500 2.11502800

C -2.96710600 0.74934600 2.64622000

C 2.04947500 -0.23894500 0.90885800

C 3.09944800 -0.32483400 -0.01559800

C 2.36306200 0.38029700 2.12601800

C 4.37546200 0.17238800 0.22899700

C 3.62987700 0.89815500 2.40735700

C 4.63779800 0.80124000 1.44670100

C 0.52571700 -2.23668000 -0.15363400

C -0.44203900 -2.53054000 -1.11606400

C 1.38185400 -3.28920100 0.18655200

C -0.57167800 -3.77758300 -1.72325900

C 1.28986300 -4.55681300 -0.39473700

C 0.30521600 -4.80168300 -1.35520800

F 0.00369500 1.97006500 1.01308600

F -2.20327600 2.94053100 2.18614600

F -4.04497800 1.22795000 3.25540800

F -3.72010200 -1.45939500 2.97630400

F -1.56577100 -2.44624900 1.73627700

F 1.44596100 0.49067000 3.09267300

F 3.88008300 1.48716800 3.57424500

F 5.83559700 1.32697800 1.67603700

F 5.32176200 0.11996100 -0.70777600

F 2.87927500 -0.86397400 -1.22195700

F -1.31011800 -1.57750800 -1.49779300

F 2.32880800 -3.11542600 1.11572200

F 2.12172400 -5.53086100 -0.03705200

F 0.20223700 -6.00090000 -1.91467500

F -1.51803100 -3.99732600 -2.63386700

H 0.58975700 0.09980300 -0.84717100

N -0.93307600 1.48642600 -2.48102200

H -0.37488700 0.94849300 -3.14490800

Si -2.65110600 1.05774200 -2.75346500

H -2.58003000 -0.21808600 -3.51212800

H -3.29733200 2.11452200 -3.58727000

C -0.41521900 2.89066700 -2.42380300

C -3.73838300 0.79435200 -1.24143400

C -4.08904700 -0.51555900 -0.85645300

C -4.35153900 1.86590600 -0.56057400

C -5.01907900 -0.74528500 0.16235900

H -3.63396900 -1.37087900 -1.35948800

C -5.27750900 1.63993300 0.46031500

H -4.11537500 2.89827400 -0.83074000

C -5.61791800 0.33171900 0.81988100

H -5.26618700 -1.76924800 0.45232600

H -5.72963600 2.48618200 0.98288600

H -6.33255000 0.15336100 1.62652900

C 1.06162000 2.86018300 -1.97648200

C 1.53862000 3.59036500 -0.87909600

C 1.99301800 2.10466800 -2.71194900

C 2.89438800 3.57372800 -0.53373100

C 3.34502100 2.07782200 -2.36881400

H 1.66736300 1.51560300 -3.57402200

C 3.80413000 2.82152200 -1.27715000

H 4.04058600 1.46096700 -2.94164600

H 4.85970100 2.79150300 -0.99796000

C -0.47607000 3.55043100 -3.82244100

H -0.04782800 4.56388400 -3.78533300

H 0.09909300 2.97264800 -4.56187800

H -1.51486100 3.62606500 -4.17818000

H 0.85473700 4.17062100 -0.26165200

H 3.23552500 4.14743100 0.33179800

C -1.30578300 3.69266900 -1.46686900

H -2.33431600 3.73076700 -1.85582500

H -1.32591700 3.26009800 -0.45932100

H -0.96151400 4.73353800 -1.39520100

**
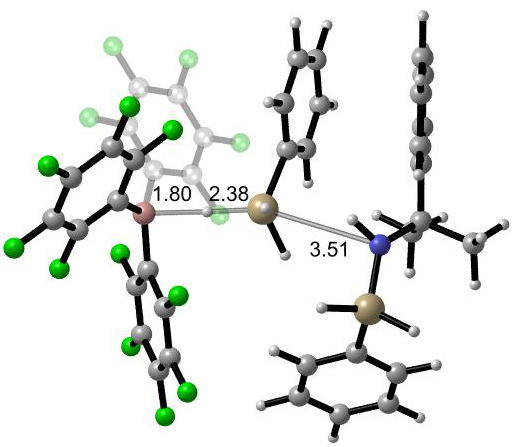
**

**TS5d**

**G_gas_ = -3655.736680 a.u.**

**E_gas_ = -3656.21526703 a.u.**

Si -0.83682500 -0.18758100 0.37664000

H 0.66714400 -0.06111800 0.27108100

C -1.31921500 -1.76888400 -0.49527000

C -1.63933400 -1.78200100 -1.86652400

C -1.27639000 -2.99602900 0.19455800

C -1.90328300 -2.98281200 -2.53008100

H -1.65897800 -0.84828200 -2.43365000

C -1.54120300 -4.19680300 -0.46646500

H -1.02512700 -3.01858900 1.25794000

C -1.85096700 -4.19221000 -1.83039500

H -2.13324800 -2.97365600 -3.59789800

H -1.50945500 -5.13922800 0.08540100

H -2.05383200 -5.13315600 -2.34846000

B 2.42934800 0.05292800 -0.09253400

C 2.34584700 1.58373400 -0.50102700

C 1.72283100 2.52257000 0.33247800

C 2.85007400 2.08257600 -1.70905300

C 1.59673700 3.86871400 0.00223400

C 2.73549600 3.42406700 -2.08241200

C 2.09167000 4.31818000 -1.22388700

C 2.88076100 -0.31002200 1.38410500

C 3.80466100 0.46529300 2.09724700

C 2.36488300 -1.42112500 2.06467500

C 4.19700800 0.16380500 3.40378600

C 2.71693400 -1.74904600 3.37177700

C 3.64694600 -0.94927900 4.04364800

C 2.38185900 -1.07447000 -1.20294900

C 3.12561300 -2.26195000 -1.12840000

C 1.51629500 -0.97442300 -2.30284700

C 3.00693400 -3.29071700 -2.06535500

C 1.36025100 -1.98027400 -3.25187900

C 2.11314300 -3.15020200 -3.12971600

H -1.38345200 1.03532400 -0.25458800

H -1.11004000 -0.25499900 1.83626400

F 3.22129200 3.85171400 -3.24320600

F 1.93706500 5.58960200 -1.57109600

F 0.97133300 4.71559100 0.81727700

F 1.17565400 2.13256800 1.49385400

F 3.47619900 1.26910100 -2.56465000

F 0.74061100 0.11141800 -2.44343900

F 0.49107100 -1.84851800 -4.25035000

F 1.97772700 -4.12360600 -4.01887900

F 3.73410000 -4.39803400 -1.95394800

F 4.00048600 -2.45563300 -0.13716700

F 1.46595500 -2.21325000 1.46062200

F 2.18522100 -2.80557700 3.97930900

F 4.00457900 -1.24738900 5.28480500

F 5.08554000 0.92136300 4.03881600

F 4.36784100 1.53799300 1.53128000

N -4.08892600 0.60712600 1.42335900

H -3.38488100 -0.03752000 1.76295300

Si -3.91137200 2.26224700 1.94404800

H -2.79545700 2.21658200 2.93105200

H -5.16903200 2.75612200 2.58080700

C -5.05040600 -0.02119600 0.49426700

C -3.47074100 3.50261400 0.58922300

C -2.13953300 3.89959700 0.35409100

C -4.47599200 4.05648000 -0.22805900

C -1.82526700 4.80515200 -0.66584100

H -1.33118600 3.51234100 0.98133900

C -4.16667100 4.94958200 -1.25767400

H -5.52471100 3.79066100 -0.05893800

C -2.83729100 5.32391500 -1.47868700

H -0.79115200 5.12028600 -0.81603600

H -4.96373200 5.36186100 -1.88225300

H -2.59062500 6.02923300 -2.27668400

C -5.03023200 -1.53906900 0.75477400

C -4.94009800 -2.48702100 -0.27421400

C -5.12288800 -2.00960500 2.07796800

C -4.94404500 -3.85737200 0.00726100

C -5.12388300 -3.37621900 2.36229200

H -5.18872900 -1.28841700 2.89583800

C -5.03570400 -4.30980300 1.32421300

H -5.19594200 -3.71381300 3.39970200

H -5.03447300 -5.38078600 1.54265400

C -6.47596500 0.50327800 0.77926800

H -7.20412300 0.00555800 0.12160200

H -6.76397800 0.31718900 1.82391700

H -6.54269500 1.58790000 0.59345700

H -4.84686500 -2.17087500 -1.31244800

H -4.85953900 -4.57239500 -0.81444500

C -4.68702100 0.32573500 -0.96325000

H -5.42814100 -0.06592700 -1.67696000

H -3.70094300 -0.07601400 -1.23033000

H -4.64926900 1.41722400 -1.08411500

**
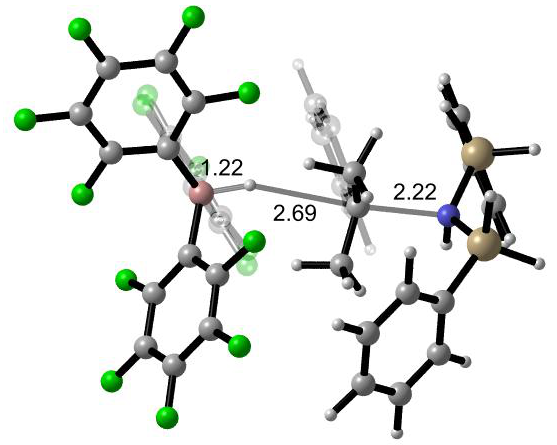
**

**TS6d**

**G_gas_ = -3655.712698 a.u.**

**E_gas_ = -3656.19517709 a.u.**

Si 4.71478900 0.06459400 -2.09042900

H 3.72364800 -0.50485500 -3.03688800

C 5.40350900 -1.25835200 -0.96811200

C 5.18323500 -2.61684200 -1.25660700

C 6.12850200 -0.92805000 0.19394100

C 5.66681500 -3.61579900 -0.40700500

H 4.60597200 -2.90318000 -2.13977600

C 6.60555900 -1.92375500 1.04886900

H 6.32928200 0.11956400 0.44419700

C 6.37337400 -3.27086600 0.74829700

H 5.47905700 -4.66653000 -0.64104400

H 7.16196100 -1.65109700 1.94915400

H 6.74413000 -4.05160900 1.41717200

C 1.79076200 0.71903900 -0.71753100

C 1.99298400 -0.70899400 -0.42995100

C 2.59801500 -1.12908800 0.77725200

C 1.46291200 -1.69270600 -1.29331400

C 2.67685900 -2.47482000 1.10666000

C 1.51727900 -3.04161700 -0.94424600

H 0.93016000 -1.40560900 -2.19844400

C 2.12367200 -3.43537300 0.25126900

H 1.05799600 -3.78528600 -1.59858600

H 2.14975800 -4.49102600 0.53118900

C 1.21638900 1.13143500 -2.02897200

H 1.67949700 0.62456200 -2.88368800

H 0.15089900 0.84903900 -2.00275300

H 1.25384100 2.21762800 -2.15676400

N 3.89269800 1.33650000 -1.09355500

H 4.15664900 1.21587300 -0.11250300

H 5.77557400 0.76995700 -2.86638400

Si 4.19702100 3.05107900 -1.62342000

H 5.63558400 3.32652000 -1.34783500

H 3.91954100 3.05486600 -3.08258200

C 3.08098900 4.22864700 -0.71084800

C 3.30691300 4.51545500 0.64965600

C 1.89218900 4.69717800 -1.30579900

C 2.34887200 5.19722100 1.40484500

H 4.22943600 4.18364500 1.13800000

C 0.92768300 5.36748400 -0.55059100

H 1.69751900 4.50929600 -2.36576900

C 1.14855100 5.60179200 0.81061500

H 2.52911900 5.39766200 2.46394500

H -0.01295900 5.67586100 -1.00966000

H 0.37494400 6.09015900 1.40660300

B -1.82233400 -0.32835300 0.02273000

C -1.50109000 -1.69299500 0.86793500

C -1.96165000 -2.97460400 0.54746200

C -0.57138100 -1.66209100 1.91114600

C -1.52847500 -4.13751500 1.19715600

C -0.11613100 -2.78684100 2.59384000

C -0.59184800 -4.04487600 2.22509200

C -2.16896000 0.98943800 0.93520400

C -2.76045800 0.96206100 2.20167200

C -1.86170100 2.26664400 0.45691500

C -3.01686100 2.11139600 2.95638200

C -2.10380800 3.44205700 1.17015000

C -2.68406000 3.36368000 2.43581700

C -2.93671500 -0.49499500 -1.15650500

C -2.59102100 -0.62249300 -2.50044000

C -4.30853300 -0.51172000 -0.89262900

C -3.52428700 -0.74823500 -3.53271900

C -5.28258100 -0.63621700 -1.88540600

C -4.88399500 -0.75360900 -3.21952100

F -1.27487000 2.41950500 -0.75327800

F -1.77502700 4.64135200 0.66310000

F -2.91424700 4.47363900 3.13931300

F -3.57329300 2.02781900 4.16585200

F -3.10084100 -0.20465100 2.76232500

F -1.28863900 -0.63928600 -2.88096000

F -3.13227400 -0.86400000 -4.80548000

F -5.79543600 -0.87223400 -4.18555200

F -6.58132100 -0.64861300 -1.57998800

F -4.74124400 -0.42750400 0.37501900

F -0.01619500 -0.48582200 2.28552500

F 0.82114100 -2.68116500 3.54490200

F -0.13174000 -5.14430500 2.82846900

F -1.98831200 -5.33563800 0.82806400

F -2.84838400 -3.16391800 -0.44266900

H -0.76553500 -0.07995700 -0.52542200

H 3.01060300 -0.39639200 1.47101800

H 3.13863400 -2.77814800 2.04656900

C 1.47518800 1.59283500 0.45004000

H 0.53462700 1.18418000 0.85570700

H 2.20625700 1.52790200 1.26570500

H 1.30721200 2.63377300 0.17205800

**
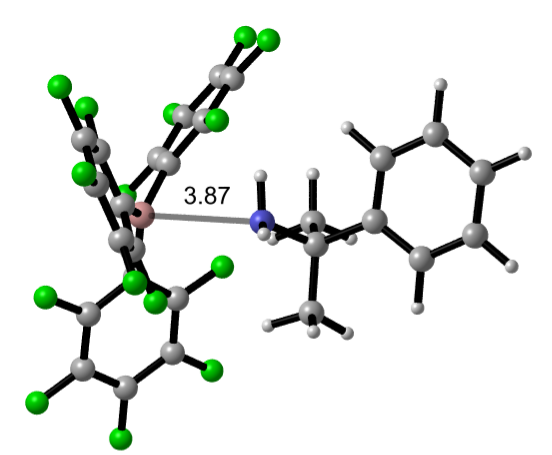
**

**TS10d**

**G_gas_ = -2611.651097 a.u.**

**E_gas_ = -2611.93436100 a.u.**

C -2.48677100 -0.52704600 1.75085500

N -2.27272600 -1.11624100 0.41453000

H -2.61428100 -0.49597100 -0.31761400

C 2.28469000 -0.36537100 0.42150800

C 2.40790000 0.29844300 1.65352300

C 3.14528900 -1.46004000 0.23040700

C 3.30334000 -0.09942400 2.64530300

C 4.06797600 -1.87653700 1.18953900

C 4.14260700 -1.19163000 2.40592500

C 0.90417300 1.58954400 -0.87444400

C -0.39236200 2.04120400 -1.17171200

C 1.87079900 2.59633200 -0.70802400

C -0.73038300 3.39069300 -1.24748900

C 1.58022000 3.95649200 -0.81022700

C 0.26643700 4.35481900 -1.07461500

C 0.50799900 -1.02248300 -1.52158600

C 0.13059300 -0.80776800 -2.85708300

C 0.09841800 -2.23933600 -0.95217900

C -0.61539300 -1.72745900 -3.59085600

C -0.67573300 -3.16772000 -1.64418700

C -1.02604400 -2.91329800 -2.97202800

F 3.37157500 0.54774400 3.80366500

F 1.63158700 1.35027900 1.93580100

F 5.00732900 -1.57724400 3.33125300

F 4.87093900 -2.90997000 0.96122900

F 3.11694100 -2.14542800 -0.91465800

F 0.41048700 -2.54075300 0.30741700

F -1.10163300 -4.27774700 -1.04671300

F -1.75327300 -3.79226600 -3.64595800

F -0.94231500 -1.49248500 -4.85836900

F 0.48958400 0.31914600 -3.48028900

F -1.39480200 1.17419500 -1.35973100

F -1.98582900 3.76219300 -1.47783800

F -0.03256900 5.64142400 -1.15913100

F 2.53083100 4.87113000 -0.65501100

F 3.13929400 2.27116100 -0.44629400

B 1.24282300 0.06641300 -0.67375400

C -3.98488000 -0.27223100 2.02578100

C -4.66375800 -0.78633900 3.14044300

C -4.72194000 0.51167800 1.11746400

C -6.02691100 -0.53283400 3.33888100

C -6.07920400 0.76919600 1.31107200

H -4.22753600 0.93384600 0.23765700

C -6.74145000 0.24484300 2.42697200

H -6.62329700 1.38294100 0.58819700

H -7.80505000 0.44277600 2.58192100

C -1.85025000 -1.49583700 2.75745800

H -0.80300700 -1.66489100 2.47068000

H -2.35998400 -2.47270300 2.75256800

H -1.87837400 -1.09877200 3.78288600

H -4.13568400 -1.39557400 3.87465100

H -6.52976100 -0.94929100 4.21587100

H -2.80641900 -1.98312300 0.33291900

C -1.73435200 0.81547000 1.80988400

H -1.85817500 1.28299300 2.79800300

H -0.66024800 0.66594900 1.63170200

H -2.11259800 1.52086500 1.05575400

**
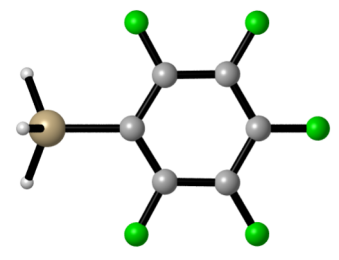
**

**C_6_F_5_SiH_3_**

**G_gas_ = -1018.443909 a.u.**

**E_gas_ = -1018.48244000 a.u.**

Si -2.99116300 0.00016300 -0.01063600

H -3.46489100 -1.21854700 -0.71506100

H -3.46492600 1.22098800 -0.71133400

H -3.48974900 -0.00203600 1.39275800

C -1.09260200 0.00019300 -0.00605300

C -0.35976600 -1.19142300 0.00129500

C -0.35947400 1.19163200 0.00128800

C 1.03710100 -1.21178900 -0.00308300

C 1.03741100 1.21159400 -0.00309200

C 1.73501200 -0.00018400 -0.00548400

F -0.99562300 2.36729300 0.01751900

F -0.99631600 -2.36688200 0.01754200

F 1.70456900 -2.36140700 0.00074400

F 3.06104400 -0.00035400 -0.00616300

F 1.70518600 2.36103600 0.00072500


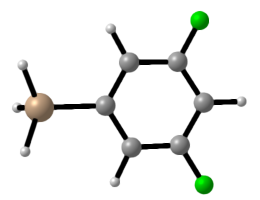


**1,3,5-C_6_H_3_F_2_SiH_3_**

**G_gas_ = -720.967829a.u.**

**E_gas_ = -721.031845632 a.u.**

C -1.14008300 1.19015300 0.00106000

C 0.25355600 1.21208600 -0.01316500

C 0.96696100 0.00000600 -0.01835800

C 0.25352300 -1.21212600 -0.01315900

C -1.14003300 -1.19019000 0.00106300

C -1.86417000 0.00001000 0.00859800

H 0.75501400 2.18237600 -0.02492500

H 0.75486100 -2.18250700 -0.02492200

Si 2.85568600 0.00002200 0.00774900

H 3.36288500 -0.01711300 1.41153700

H 3.36038800 -1.21372300 -0.69521100

H 3.35865700 1.23101300 -0.66574400

F -1.81085900 2.34857700 0.00436000

F -1.81088300 -2.34857300 0.00436200

H -2.95424500 -0.00001700 0.01605000

**
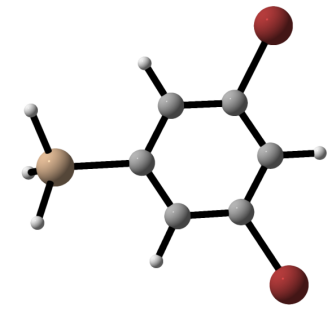
**

**1,3,5-C_6_H_3_Br_2_SiH_3_**

**G_gas_ = -5669.196167 a.u.**

**E_gas_ = -5669.25207170 a.u.**

C -1.19902000 -0.23590000 -0.00119300

C -1.21177600 1.16156300 -0.01376100

C -0.00002600 1.87480800 -0.01752400

C 1.21178400 1.16154000 -0.01375500

C 1.19905800 -0.23584100 -0.00119300

C -0.00000800 -0.95373600 0.00576600

H -2.17053100 1.68444000 -0.02504900

H 2.17062000 1.68432500 -0.02504600

Si -0.00001000 3.76505100 0.00845200

H 0.01717900 4.27155300 1.41219000

H 1.21417300 4.26656200 -0.69552200

H -1.23150300 4.26484300 -0.66595900

Br -2.84951100 -1.19247400 0.00168800

Br 2.84951500 -1.19248200 0.00168900

H 0.00002100 -2.04361300 0.01282600

**_
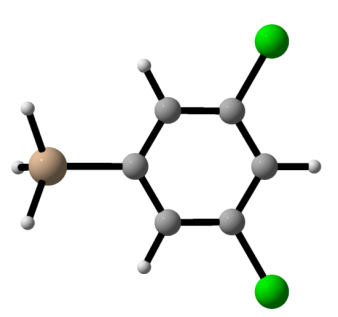
_**

**1,3,5-C_6_H_3_Cl_2_SiH_3_**

**G_gas_ = -1441.589141 a.u.**

**E_gas_ = -1441.64835102 a.u.**

C 0.74333500 -1.19945800 -0.00021800

C -0.65453100 -1.21129900 -0.01335500

C -1.36770800 -0.00004400 -0.01757300

C -0.65454400 1.21129300 -0.01335000

C 0.74323800 1.19952000 -0.00021700

C 1.45988600 0.00001500 0.00694000

H -1.17118800 -2.17334100 -0.02469700

H -1.17113500 2.17340000 -0.02469400

Si -3.25723900 -0.00006400 0.00806900

H -3.76473600 0.01711400 1.41157100

H -3.75989400 1.21386200 -0.69573600

H -3.75814300 -1.23130600 -0.66618300

H 2.54949400 0.00006200 0.01419700

Cl 1.61941900 -2.71200600 0.00291800

Cl 1.61934000 2.71206100 0.00291900


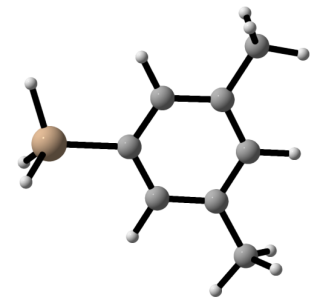


**1,3,5-C_6_H_3_Me_2_SiH_3_**

**G_gas_ = -601.167174 a.u.**

**E_gas_ = -601.296211063 a.u.**

C -1.11587600 1.22488100 0.00018300

C 0.28255300 1.21266600 -0.00149100

C 1.00889500 0.00463700 -0.00203900

C 0.29606300 -1.20275300 -0.00144200

C -1.11027100 -1.22666400 0.00021600

C -1.79389000 -0.00718000 0.00113200

H 0.81383500 2.17059400 -0.00292300

H 0.83597800 -2.15540900 -0.00276900

Si 2.89178600 0.01441800 0.00088600

H 3.42482600 0.67225400 1.23198500

H 3.38983300 -1.39111000 -0.05075500

H 3.42508400 0.76452900 -1.17588300

H -2.88894300 -0.00950300 0.00222200

C -1.89550300 2.51762000 0.00037500

H -2.54800500 2.58879500 -0.88606300

H -2.54909400 2.58787700 0.88606400

H -1.23158900 3.39482400 0.00119300

C -1.84702400 -2.54453300 0.00034400

H -1.58759400 -3.14483200 -0.88780000

H -1.58091000 -3.14852300 0.88396400

H -2.93810100 -2.40339100 0.00468600

**
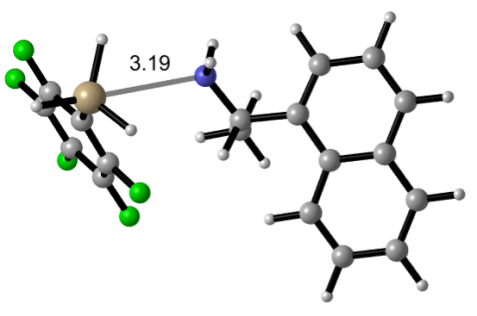
**

**TS1 (C_6_F_5_-SiH_3_)**

**G_gas_ = -1537.770890 a.u.**

**E_gas_ = -1538.01443500 a.u.**

Si -1.92838000 -2.07138900 -1.90217600

H -2.85410700 -2.37068300 -3.03483900

C -2.70471900 -0.66108600 -0.89928700

C -2.15169600 0.61809800 -0.82251000

C -3.87710300 -0.88105100 -0.17236000

C -2.71191300 1.62974300 -0.03992900

C -4.47481700 0.10653000 0.61418400

C -3.88232100 1.37092900 0.67851000

N 0.27607500 -1.66025300 0.36183200

H 0.68591000 -2.42967900 -0.16735100

H 0.13868800 -2.00053400 1.31400100

C 1.15210500 -0.48736700 0.33312600

H 1.02185200 -0.04262700 -0.66491400

C 0.66010200 0.53512500 1.36787700

H 0.73827100 0.11435000 2.38439100

H 1.26866100 1.44970900 1.34075900

H -0.38684200 0.80984200 1.18144800

C 2.64467200 -0.76551900 0.52965600

C 3.62796000 0.15553900 0.02432900

C 3.07078700 -1.88072900 1.22787800

C 3.28890600 1.33135300 -0.70927600

C 5.02208500 -0.11031200 0.25957900

C 4.44393100 -2.14021300 1.46088400

H 2.33298900 -2.58836600 1.61287900

C 4.26185100 2.19056800 -1.17859700

H 2.24261700 1.56487500 -0.90973200

C 5.99921600 0.79634800 -0.23934300

C 5.40119600 -1.27325200 0.98689100

H 4.73519200 -3.03362900 2.01913600

C 5.63225300 1.92392900 -0.94198600

H 3.97223200 3.08313700 -1.73896300

H 7.05498200 0.58041000 -0.05305200

H 6.46351000 -1.46469800 1.16069700

H 6.39385400 2.61156200 -1.31818000

H -0.61978800 -1.65055500 -2.45627300

H -1.80592700 -3.29223100 -1.06626300

F -5.58933600 -0.14099000 1.29672000

F -4.46279500 -2.08468600 -0.21204900

F -4.42644900 2.32266700 1.42699500

F -2.13081000 2.82445600 0.04465100

F -1.02753100 0.91505700 -1.48785600

**
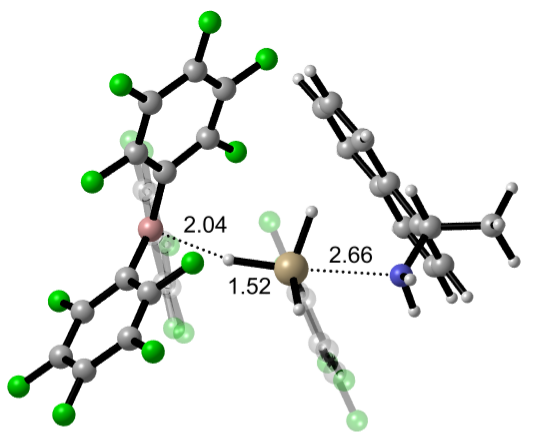
**

**TS2(C_6_F_5_-SiH_3_)**

**G_gas_ = -3744.330906 a.u.**

**E_gas_ = -3744.69901100 a.u.**

Si 0.23351500 0.10539400 1.83743600

H -0.87427500 -0.13096600 0.82586900

C 1.61388600 -0.99006600 1.13516700

C 2.17025900 -2.04452700 1.86272100

C 2.10797300 -0.80708400 -0.15688100

C 3.15673300 -2.88390300 1.34175900

C 3.08842800 -1.62467800 -0.71439400

C 3.61243900 -2.67441100 0.03798500

B -2.28774400 0.00751200 -0.63933900

C -3.24672400 -0.98061900 0.13805400

C -3.60720000 -0.76001200 1.47556200

C -3.78786600 -2.12738700 -0.45928700

C -4.43137700 -1.62452100 2.19365000

C -4.62933500 -3.01010600 0.22013000

C -4.94993100 -2.75563300 1.55582100

C -2.50857100 1.56776300 -0.47315200

C -3.76917500 2.12761100 -0.21183800

C -1.44418200 2.48163500 -0.52663300

C -3.96594700 3.49327500 0.00520200

C -1.59491400 3.84546400 -0.28594900

C -2.86893700 4.35770000 -0.02829800

C -1.27321300 -0.55761700 -1.70740300

C -0.91157200 0.14523900 -2.86707900

C -0.60429700 -1.77705800 -1.50776900

C 0.06953200 -0.30082000 -3.75275000

C 0.37918000 -2.25547500 -2.37010300

C 0.72678500 -1.50546700 -3.49524900

N 2.15950600 0.73129100 3.56808400

H 2.70529800 -0.09823700 3.80768500

H 1.64669100 0.98728800 4.41433000

C 3.03373900 1.84626700 3.14665200

H 2.35272500 2.64762500 2.83016400

C 3.89129000 2.40862200 4.29103800

H 4.57405600 1.64552600 4.69721600

H 4.50127000 3.25644300 3.94221300

H 3.25112600 2.76407600 5.11565000

C 3.87797900 1.41540600 1.94933900

C 3.68727400 1.96181400 0.63297700

C 4.84161400 0.43794600 2.13984500

C 2.70921900 2.94892100 0.31370900

C 4.50089500 1.46074000 -0.44684900

C 5.64389700 -0.04372900 1.07986200

H 4.99233600 0.01286800 3.13621200

C 2.52931700 3.39749000 -0.97757200

H 2.05993500 3.34928400 1.09161000

C 4.28684500 1.94184100 -1.76827700

C 5.47828100 0.46179200 -0.18960100

H 6.38467800 -0.82320300 1.27362500

C 3.32188400 2.88794400 -2.03303800

H 1.74819100 4.13072500 -1.18497400

H 4.89867600 1.53235900 -2.57614900

H 6.07718200 0.08399200 -1.02193700

H 3.15955400 3.23961500 -3.05477500

H -0.31931500 -0.41571800 3.10645300

H 0.41487100 1.56932900 1.75285800

F -5.12737600 -4.08205000 -0.38854700

F -5.74451400 -3.58480800 2.21826600

F -4.73300100 -1.38333700 3.46626200

F -3.13968400 0.31206400 2.12875400

F -3.51745600 -2.41405500 -1.73661100

F -0.84722400 -2.50894100 -0.41482800

F 1.02636600 -3.38940500 -2.10477000

F 1.67567700 -1.93527900 -4.31180300

F 0.38701300 0.41038900 -4.83049900

F -1.50987100 1.30077000 -3.17177100

F -0.20780300 2.05561600 -0.78607000

F -0.53984700 4.66266700 -0.30678700

F -3.03498500 5.65490800 0.18680900

F -5.18190600 3.97603000 0.24157900

F -4.85894100 1.35582900 -0.16829500

F 1.78892800 -2.26356600 3.13143400

F 3.67454100 -3.86646400 2.07595700

F 4.56548800 -3.44679600 -0.46793500

F 3.52923400 -1.39926900 -1.95052600

F 1.64810900 0.19355800 -0.91552300

**
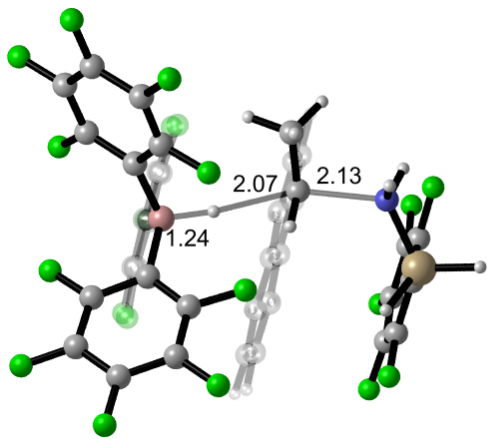
**

**TS3(C_6_F_5_-SiH_3_)**

**G_gas_ = -3744.311316 a.u.**

**E_gas_ = -3744.67895200 a.u.**

C 0.49633700 0.34186500 2.10567400

H 0.16217900 -0.68091100 1.98104500

C 1.37328700 0.90438100 1.09110700

C 1.79432100 0.12571100 -0.05272800

C 1.78203300 2.23685700 1.19552600

C 1.42699800 -1.22884900 -0.26292500

C 2.60258600 0.76429300 -1.05377600

C 2.59606300 2.84216700 0.22429800

H 1.45720300 2.83771700 2.04509600

C 1.82554100 -1.90994000 -1.39605100

H 0.80098600 -1.74607400 0.45829400

C 2.99469600 0.03661500 -2.21171500

C 2.98707000 2.12045600 -0.88675400

H 2.89084500 3.88629400 0.33671100

C 2.61355700 -1.27558500 -2.38389900

H 1.51161100 -2.94618600 -1.53502900

H 3.59088100 0.54597700 -2.97230600

H 3.59456100 2.59233200 -1.66290700

H 2.90683600 -1.82118700 -3.28241700

C -0.35026400 1.20768300 2.99115200

H 0.24301100 1.92965500 3.57088100

H -1.02698400 1.78875000 2.34561400

H -0.96967500 0.60232200 3.66352600

N 1.82020800 -0.34021800 3.63482700

H 2.29962300 0.52398400 3.89440600

Si 2.94907700 -1.68680600 3.25476800

H 3.68151700 -2.09633400 4.48263300

H 2.12290400 -2.79268000 2.72665300

C 4.12896300 -1.02456500 1.94877200

C 4.59805500 0.29091900 1.98297200

C 4.48657300 -1.79581400 0.83816000

C 5.35639900 0.84494800 0.95510700

C 5.24932400 -1.27888800 -0.20974300

C 5.67778700 0.04900700 -0.14804700

B -1.91815300 0.04712400 -0.10755600

C -1.14549000 0.86048300 -1.28217000

C -0.76398700 2.18645000 -1.04677400

C -0.64224000 0.31590300 -2.46785800

C 0.03288700 2.93711400 -1.90680800

C 0.16167100 1.02918400 -3.36334900

C 0.50553200 2.34834700 -3.08029900

C -2.34072700 -1.49554200 -0.41533400

C -3.25371200 -1.79393200 -1.43125500

C -1.91627800 -2.58738100 0.33759700

C -3.69628300 -3.08633400 -1.71467800

C -2.32704400 -3.90190600 0.09688700

C -3.22833200 -4.15103000 -0.93802300

C -3.19528900 0.81003300 0.56099900

C -3.57386400 0.48674900 1.86717400

C -3.99274200 1.76981600 -0.06886100

C -4.64987100 1.07506900 2.53385300

C -5.08391100 2.38363000 0.55458800

C -5.41519700 2.03269800 1.86527200

F -1.04519400 -2.43212000 1.37058700

F -1.87315300 -4.91065600 0.84630200

F -3.63754000 -5.39427500 -1.18726400

F -4.55761900 -3.31964700 -2.70475000

F -3.72749300 -0.79986200 -2.19494000

F -2.87471200 -0.44192700 2.55709800

F -4.95158500 0.73425100 3.78989300

F -6.45018900 2.60956200 2.47459400

F -5.81199000 3.29835000 -0.08653300

F -3.72849600 2.15447700 -1.32424900

F -0.85925100 -0.96994500 -2.78594900

F 0.66488800 0.43305200 -4.44736400

F 1.34258000 3.01521800 -3.87893900

F 0.41178100 4.17770600 -1.58483300

F -1.12289300 2.79253600 0.10653900

H -1.06910600 0.01038000 0.78889800

H 1.15269500 -0.56475100 4.37616600

F 4.27493900 1.08757300 3.02004100

F 5.73299600 2.11752300 0.98989800

F 6.34102200 0.57548000 -1.16612200

F 5.53330100 -2.01764500 -1.27341800

F 4.06377000 -3.05762700 0.73956800

**
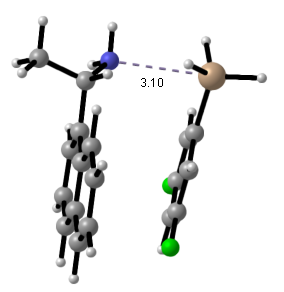
**

**TS1(1,3,5-C_6_H_3_F_2_SiH_3_)**

**G_gas_ = -1240.291346 a.u.**

**E_gas_ = -1240.56379727 a.u.**

Si -2.08663000 2.03673200 -1.91872000

H -2.63531600 1.85493200 -3.30223600

C -2.02238800 0.34358600 -1.09030500

C -2.99454500 -0.02008200 -0.14367500

C -1.02190600 -0.58307200 -1.43124800

C -2.95450200 -1.28834900 0.43097800

H -3.78365600 0.66703600 0.16871900

C -1.01755400 -1.83918700 -0.83138200

H -0.22499800 -0.34885100 -2.14012200

C -1.97859600 -2.22349800 0.10000400

N -0.77206500 2.78954100 0.78298900

H -1.13455700 2.62273400 1.72205500

H -0.88834500 3.78959500 0.61589600

C 0.64933800 2.44214200 0.68970900

H 0.92420300 2.65648900 -0.35374900

C 1.55885500 3.29083000 1.59943500

H 1.30415300 3.12110400 2.65857400

H 2.61970900 3.02751300 1.46267700

H 1.43929500 4.36531400 1.38008000

C 0.89087000 0.95875200 0.94575200

C 1.91158700 0.24634200 0.22583400

C 0.16824400 0.28837300 1.91577900

C 2.71470400 0.84298400 -0.79105300

C 2.13532200 -1.14356900 0.52191000

C 0.40196500 -1.07444000 2.21678200

H -0.62045100 0.80456700 2.46697300

C 3.66013500 0.11465400 -1.48380900

H 2.58446600 1.89740900 -1.03620000

C 3.11225200 -1.86774700 -0.21645100

C 1.36593400 -1.77824600 1.53386800

H -0.20194100 -1.56434200 2.98421000

C 3.86051300 -1.25817100 -1.19930200

H 4.25660800 0.60014900 -2.26048400

H 3.25464400 -2.92777300 0.00988300

H 1.54068000 -2.83629800 1.74437100

H 4.60549100 -1.82813300 -1.76000100

H -3.00901800 2.95269800 -1.19558100

H -0.72893900 2.62644000 -2.08291200

F -3.88193000 -1.62375100 1.34198400

F -0.04849000 -2.71202100 -1.14479300

H -1.95235200 -3.20748900 0.56733300

**
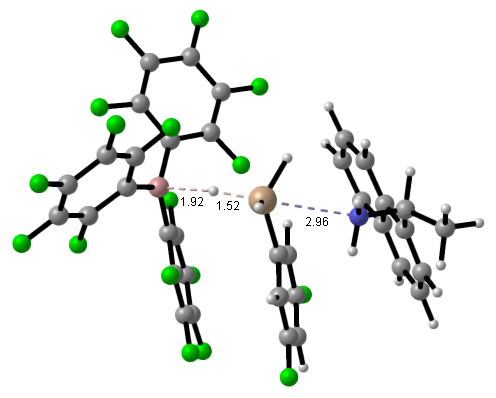
**

**TS2(1,3,5-C_6_H_3_F_2_SiH_3_)**

**G_gas_ = -3446.851087 a.u.**

**E_gas_ = -3447.24568365 a.u.**

Si 0.43032400 -0.07003700 1.81679500

H -0.84364700 -0.03142000 0.98193600

C 1.63785100 -1.04233100 0.75135000

C 1.91240800 -2.38647900 1.06546500

C 2.21501000 -0.47923300 -0.39776100

C 2.75460000 -3.12883700 0.24082500

H 1.46795900 -2.88324300 1.93050600

C 3.03800900 -1.26231100 -1.20029800

H 2.04410600 0.55412200 -0.69069800

C 3.33239100 -2.59026800 -0.90615300

B -2.11784200 0.02779200 -0.44839200

C -3.23155000 -0.68776600 0.42341800

C -3.55309900 -0.24003000 1.71182800

C -3.97146800 -1.77951000 -0.04779400

C -4.52735700 -0.84336000 2.50575600

C -4.96456600 -2.40352200 0.70933700

C -5.24198700 -1.93068300 1.99441100

C -2.17394300 1.61135500 -0.56031700

C -3.38458700 2.31886400 -0.50722700

C -1.02421900 2.39957100 -0.70967100

C -3.45633700 3.71204000 -0.59275100

C -1.05080200 3.78934700 -0.78961300

C -2.28016800 4.45177500 -0.72810500

C -1.20455100 -0.82594100 -1.41504900

C -0.70688500 -0.35145500 -2.63963100

C -0.77962600 -2.11672600 -1.05526700

C 0.17005200 -1.08466800 -3.44166600

C 0.07949200 -2.88568000 -1.83458000

C 0.57010200 -2.35702200 -3.02960800

N 2.86705900 -0.12596200 3.50288800

H 3.04413500 -1.09632200 3.23546500

H 2.46358000 -0.15386000 4.44151800

C 4.15014500 0.60738900 3.52791200

H 3.90989600 1.64581100 3.80747200

C 5.11000000 0.07661500 4.60186100

H 5.32170900 -0.99596300 4.46482000

H 6.06804100 0.61946600 4.59390100

H 4.65803200 0.19546000 5.59991500

C 4.73691800 0.63147000 2.11876700

C 4.23608200 1.55937400 1.14031800

C 5.71190000 -0.27380400 1.73974100

C 3.20823700 2.50428400 1.42080200

C 4.75889500 1.51822800 -0.19840900

C 6.23176000 -0.30696800 0.42168800

H 6.10074600 -0.99194100 2.46339100

C 2.69957100 3.32799600 0.43884100

H 2.78824700 2.55978900 2.42406500

C 4.21531200 2.38364800 -1.18906500

C 5.76919300 0.57357700 -0.52753300

H 6.99923600 -1.04192100 0.16691700

C 3.19703500 3.26134700 -0.88498700

H 1.89148900 4.02314900 0.67186000

H 4.60518400 2.31818400 -2.20800900

H 6.14944800 0.54283300 -1.55128900

H 2.76176100 3.90033100 -1.65641500

H 0.06177500 -0.83406300 3.03653700

H 0.72563600 1.35423000 2.06137300

F -5.64859200 -3.43360600 0.22026200

F -6.18046700 -2.51284200 2.72896600

F -4.78735300 -0.39416100 3.73052500

F -2.89776100 0.80323300 2.23917400

F -3.75156600 -2.26343100 -1.27492700

F -1.15831800 -2.64564800 0.11421100

F 0.47296300 -4.09051000 -1.43144500

F 1.42852100 -3.05574500 -3.75862200

F 0.62823900 -0.58215900 -4.58358000

F -1.06816100 0.84941800 -3.10208300

F 0.17926300 1.81513900 -0.77302700

F 0.07429900 4.49357600 -0.92054500

F -2.32778900 5.77393300 -0.80639200

F -4.62939900 4.33517600 -0.54677300

F -4.54798600 1.67158600 -0.39268200

F 3.00984900 -4.40595000 0.54953100

F 3.56022700 -0.72191900 -2.31171400

H 3.97129500 -3.18787400 -1.55556000

**
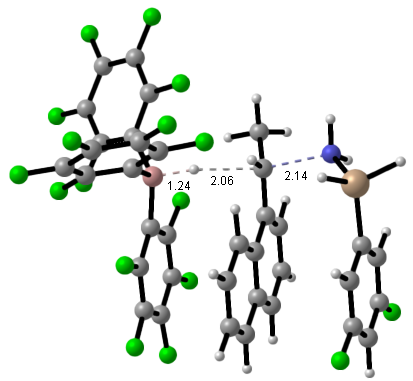
**

**TS3(1,3,5-C_6_H_3_F_2_SiH_3_)**

**G_gas_ = -3446.828453 a.u.**

**E_gas_ = -3447.22173478 a.u.**

C -0.70327600 -0.39975300 2.12859300

H -0.36275700 0.62666600 2.06980200

C -1.60367700 -0.88554300 1.09775500

C -2.05373400 -0.02148400 0.03071100

C -2.03061700 -2.21607800 1.12610000

C -1.63994900 1.32833700 -0.11699100

C -2.93293800 -0.56729800 -0.96444200

C -2.90560700 -2.73399700 0.15768200

H -1.67060100 -2.88401500 1.90922500

C -2.06375100 2.09120500 -1.18717400

H -0.95164100 1.77295400 0.59571000

C -3.35608300 0.24672100 -2.05095400

C -3.34374900 -1.92244700 -0.87065200

H -3.21225200 -3.77982200 0.20524400

C -2.93061600 1.55132600 -2.16496800

H -1.70352400 3.11714100 -1.28869000

H -4.00766300 -0.19194500 -2.81022900

H -4.00095300 -2.32505700 -1.64530700

H -3.24606900 2.16164900 -3.01308900

C 0.13015300 -1.32519500 2.96473900

H -0.47395300 -2.06246800 3.51335600

H 0.79041100 -1.88905300 2.28741200

H 0.76593400 -0.76662000 3.66172300

N -2.05420500 0.20534500 3.67708400

H -2.51010600 -0.68505200 3.88596300

Si -3.21026900 1.55092000 3.34052100

H -3.86278800 1.93679300 4.62413600

H -2.40317400 2.67847800 2.81632800

C -4.44377500 0.93508200 2.07402200

C -4.96767900 -0.36720000 2.14497800

C -4.82756200 1.77429200 1.01450500

C -5.84213300 -0.80826600 1.15485600

C -5.71235900 1.29639800 0.05027600

C -6.23927200 0.00844000 0.09985400

B 1.68301000 -0.04167000 -0.08979100

C 0.85691800 -0.74339700 -1.29913000

C 0.43612600 -2.06999300 -1.15068300

C 0.33838300 -0.09447900 -2.42418300

C -0.41421900 -2.72576300 -2.03681600

C -0.52161400 -0.70886500 -3.34046400

C -0.90415400 -2.03324000 -3.14454000

C 2.15478800 1.50247100 -0.30414400

C 3.06570800 1.83301500 -1.31187300

C 1.77473400 2.55877600 0.51954200

C 3.54866400 3.12517500 -1.52058500

C 2.22684500 3.87146200 0.35490400

C 3.12521200 4.15441400 -0.67379900

C 2.94440500 -0.89062800 0.49935200

C 3.36304700 -0.66381500 1.81367400

C 3.69265200 -1.83553600 -0.20862500

C 4.43141900 -1.33056000 2.41595300

C 4.77390100 -2.52553600 0.34877200

C 5.14626300 -2.27023500 1.67049100

F 0.90836400 2.36812800 1.54996900

F 1.81449400 4.84660900 1.17016400

F 3.57333300 5.39671600 -0.85113300

F 4.40658200 3.39108600 -2.50551500

F 3.49610800 0.87341200 -2.14248700

F 2.71426300 0.24404800 2.57609800

F 4.77359300 -1.08033200 3.68290000

F 6.17262300 -2.92063200 2.21736700

F 5.45417800 -3.42263600 -0.36573900

F 3.38810000 -2.12952600 -1.47919600

F 0.59579000 1.20276600 -2.65501300

F -1.03546200 -0.01610200 -4.36021500

F -1.78999900 -2.60915800 -3.96133100

F -0.82539900 -3.97488700 -1.79724600

F 0.80693500 -2.77207000 -0.05729000

H 0.85148600 -0.03579400 0.82656600

H -1.41363200 0.40147600 4.45025500

H -4.42352100 2.78020200 0.89077000

H -4.70325400 -1.07331700 2.93492500

F -6.30162000 -2.06293200 1.20783400

F -6.04908600 2.08833200 -0.97111900

H -6.91367700 -0.35636400 -0.67486200

**
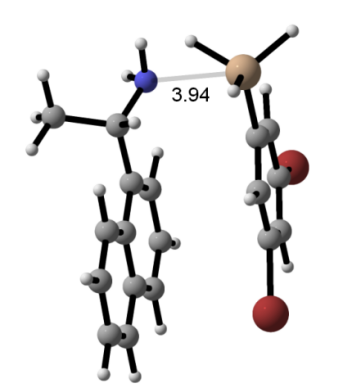
**

**TS1(1,3,5-C_6_H_3_Br_2_SiH_3_)**

**G_gas_ = -6188.523158 a.u.**

**E_gas_ = -6188.78665118 a.u.**

Si 1.39236100 1.03655500 3.61519500

H 0.17978000 1.22661900 4.46172800

C 0.95408800 -0.09285000 2.17062500

C 1.92884800 -0.44028600 1.21775300

C -0.34821700 -0.60264700 2.03532800

C 1.59341100 -1.29135300 0.16560300

H 2.93625300 -0.02722000 1.26692700

C -0.65819800 -1.44703900 0.96539800

H -1.12965100 -0.34311800 2.75183100

C 0.30226000 -1.80481900 0.01837200

N 2.37774400 2.56969300 0.12681200

H 2.85330500 2.91690900 -0.70801400

H 2.73956000 3.12915600 0.89901700

C 0.93343800 2.78723100 0.01511200

H 0.52405500 2.57873100 1.01655100

C 0.57844700 4.24303500 -0.35537000

H 1.00549100 4.49168600 -1.34118200

H -0.50786400 4.40825700 -0.41380800

H 0.99553800 4.94216900 0.38856900

C 0.27660000 1.81014400 -0.95391600

C -1.13597300 1.54801500 -0.87606900

C 1.01781100 1.18894000 -1.94116700

C -1.97860600 2.09456000 0.13644200

C -1.74052700 0.67673700 -1.84715800

C 0.42291900 0.32116400 -2.88843200

H 2.09812000 1.33075000 -1.97527400

C -3.33067300 1.82138700 0.17542600

H -1.55272500 2.73687400 0.90781800

C -3.13933600 0.42352200 -1.78430900

C -0.92955300 0.07417600 -2.84869200

H 1.05245000 -0.15943300 -3.64069200

C -3.92323800 0.98522700 -0.80104900

H -3.94835400 2.25089900 0.96832400

H -3.58176300 -0.24154200 -2.53091800

H -1.39872900 -0.59205700 -3.57769400

H -4.99481700 0.77515000 -0.76147000

H 2.48785900 0.44630300 4.43797200

H 1.85615900 2.37078900 3.13166100

Br -2.42961100 -2.13168200 0.79260000

H 0.04928600 -2.45359500 -0.81899500

Br 2.92269100 -1.75568500 -1.12363300

**
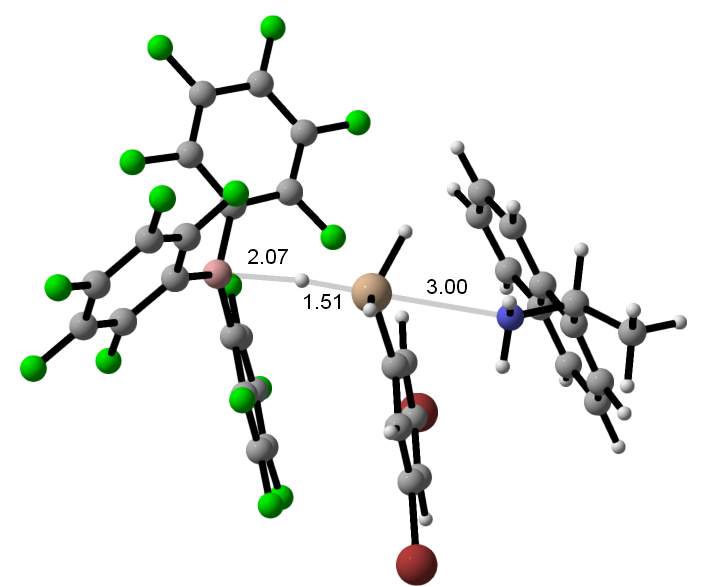
**

**TS2(1,3,5-C_6_H_3_Br_2_SiH_3_)**

**G_gas_ = -8395.081474 a.u.**

**E_gas_ = -8395.46918847 a.u.**

Si -0.03135800 0.11933900 1.98030900

H -1.26697700 -0.00251600 1.12063900

C 1.36548200 -0.54457000 0.89829800

C 1.85626100 -1.83771600 1.15259800

C 1.85420900 0.16268900 -0.21420500

C 2.82288600 -2.39959800 0.31539600

H 1.47932300 -2.42205400 1.99452400

C 2.78607400 -0.44176600 -1.06010500

H 1.50813100 1.16927500 -0.43866200

C 3.29259100 -1.71940000 -0.80992400

B -2.55954700 -0.06072500 -0.49369900

C -3.55444400 -0.99670700 0.30229600

C -4.00050500 -0.67052200 1.59113100

C -4.05892900 -2.18766200 -0.23751700

C -4.87216600 -1.47832200 2.32002900

C -4.94503500 -3.01585600 0.45290200

C -5.35167300 -2.65688000 1.74063600

C -2.84402200 1.49718100 -0.49769500

C -4.14922300 2.01197000 -0.45376500

C -1.81964100 2.45490100 -0.52454400

C -4.42729100 3.38080500 -0.43420900

C -2.05365500 3.82731200 -0.49162000

C -3.37063300 4.29367500 -0.44618600

C -1.45783900 -0.69433500 -1.42334000

C -1.01299800 -0.09196300 -2.61245900

C -0.82968000 -1.90842300 -1.09224900

C -0.01615300 -0.64298900 -3.41893700

C 0.16296200 -2.49277200 -1.87319000

C 0.57707900 -1.84916700 -3.04110800

N 2.34651800 0.32216100 3.80020100

H 2.69945500 -0.57361000 3.45783400

H 1.91526000 0.13511700 4.70760100

C 3.47987600 1.25506600 3.96077700

H 3.05343300 2.20365400 4.32612200

C 4.48302800 0.78785300 5.02442500

H 4.88420700 -0.21214200 4.79376200

H 5.33048700 1.48471400 5.11870200

H 3.98435700 0.72163600 6.00494000

C 4.09512600 1.52474500 2.59036300

C 3.44929900 2.42506500 1.67233100

C 5.24498200 0.87153900 2.18486800

C 2.24552300 3.11982000 1.98291400

C 4.01709000 2.62286800 0.36685400

C 5.80721600 1.07337800 0.89969400

H 5.74606800 0.17751700 2.86137300

C 1.61785200 3.92444100 1.05504700

H 1.78931400 2.99014700 2.96302100

C 3.35141600 3.46878800 -0.56456600

C 5.21013300 1.93557300 0.01086400

H 6.71649600 0.53495800 0.62154200

C 2.16866600 4.09615900 -0.23763600

H 0.67924900 4.42155000 1.30656200

H 3.78569800 3.58996400 -1.55960700

H 5.62789600 2.08653700 -0.98684900

H 1.64564700 4.71668600 -0.96830500

H -0.26105000 -0.79188800 3.13198800

H 0.01950700 1.54591100 2.35153700

F -5.40644300 -4.13308600 -0.10053300

F -6.19004600 -3.43318000 2.41309600

F -5.25490100 -1.13866200 3.54752600

F -3.57081600 0.44976400 2.18509300

F -3.70722300 -2.57086700 -1.46911900

F -1.14411800 -2.54072900 0.04410200

F 0.74194600 -3.63024400 -1.50339100

F 1.54836700 -2.37278600 -3.77310100

F 0.37467400 -0.03040400 -4.53068700

F -1.55438300 1.05462300 -3.03512500

F -0.54014600 2.06370600 -0.56563900

F -1.04428300 4.69836100 -0.49886900

F -3.61373500 5.59580700 -0.42073000

F -5.68137700 3.81917600 -0.40335200

F -5.20311800 1.19082000 -0.44963400

H 4.01592400 -2.17858800 -1.48272400

Br 3.48887000 -4.14666400 0.68844800

Br 3.33758500 0.45949600 -2.65036200

**
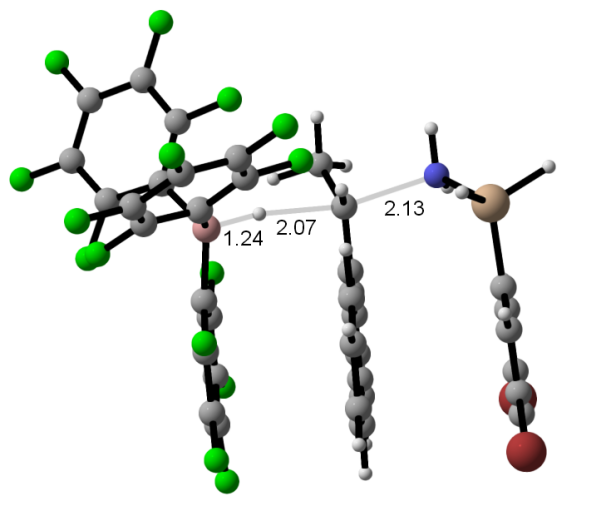
**

**TS3(****1,3,5-C_6_H_3_Br_2_SiH_3_)**

**G_gas_ = -8395.058621 a.u.**

**E_gas_ = -8395.44438273 a.u.**

C -0.03179300 -0.94661700 -1.96506400

H -0.38238700 0.05768700 -2.16936600

C 0.87350200 -1.14322100 -0.84705700

C 1.32241800 -0.02913000 -0.04415000

C 1.30606300 -2.43388700 -0.52852000

C 0.90296500 1.31079500 -0.25220400

C 2.20662500 -0.29354600 1.05614100

C 2.18586100 -2.67764700 0.53760500

H 0.94720300 -3.28442800 -1.10890600

C 1.31990200 2.32662400 0.58530100

H 0.21307200 1.55215500 -1.05538600

C 2.62304100 0.77594400 1.89532100

C 2.62449900 -1.62424600 1.31615700

H 2.50131500 -3.69742900 0.76065400

C 2.18588600 2.06209500 1.67078100

H 0.95432800 3.34198400 0.41814000

H 3.27911900 0.55356800 2.73992200

H 3.29029500 -1.80871400 2.16258500

H 2.49574000 2.87225300 2.33273200

C -0.86459400 -2.06086800 -2.52629600

H -0.26003500 -2.91526200 -2.86396900

H -1.52094500 -2.42770600 -1.72172200

H -1.50449300 -1.70640400 -3.34298900

N 1.30488500 -0.75379200 -3.61855700

H 1.76534500 -1.66593700 -3.60119100

Si 2.44789300 0.64307100 -3.63522800

H 3.09371100 0.70133100 -4.97746800

H 1.63140100 1.85878000 -3.40636800

C 3.70037100 0.38331300 -2.26547200

C 4.22307500 -0.89054400 -1.98622300

C 4.12163800 1.48306200 -1.49822300

C 5.13997500 -1.05309300 -0.94535100

C 5.05405700 1.29952400 -0.47417900

C 5.57118400 0.03445400 -0.18200700

B -2.41433900 -0.02249000 0.09804500

C -1.58248300 -0.38978000 1.44386200

C -1.16118800 -1.71021600 1.63839300

C -1.05986100 0.52539300 2.36300000

C -0.30581200 -2.11735800 2.65866100

C -0.19584600 0.16582700 3.40263300

C 0.18776600 -1.16448400 3.55031700

C -2.88626000 1.52505100 -0.09062200

C -3.78922700 2.10709800 0.80404900

C -2.51336100 2.33106100 -1.16307200

C -4.27084200 3.41009400 0.67450000

C -2.96439500 3.64244300 -1.34062200

C -3.85447100 4.18398500 -0.41333300

C -3.67799100 -0.99426500 -0.24597900

C -4.09792000 -1.12032600 -1.57330800

C -4.42590400 -1.71948600 0.68592300

C -5.16742900 -1.92082800 -1.97834500

C -5.50827200 -2.53067800 0.33033500

C -5.88207100 -2.63106300 -1.01165100

F -1.65549000 1.87836500 -2.11598000

F -2.55887000 4.37231100 -2.38403000

F -4.30142900 5.43072500 -0.56045100

F -5.12093900 3.92381100 1.56354500

F -4.21287800 1.39642200 1.85825700

F -3.44931000 -0.44553000 -2.54836400

F -5.51077800 -2.01196100 -3.26632700

F -6.90956500 -3.40131200 -1.36762700

F -6.18819900 -3.20826600 1.25598900

F -4.11954800 -1.67025000 1.98870200

F -1.31730600 1.83865700 2.25503400

F 0.32072000 1.09642800 4.20958500

F 1.07728200 -1.51221800 4.48382500

F 0.10609200 -3.38614100 2.74453500

F -1.53675600 -2.66907100 0.76320000

H -1.58871000 -0.25344700 -0.79240300

H 0.66065800 -0.76047200 -4.41334300

H 3.71339800 2.47987200 -1.67288400

H 3.91943800 -1.77223400 -2.55406800

H 6.28497100 -0.10207100 0.63016100

Br 5.80041700 -2.79287400 -0.53900500

Br 5.61991700 2.79235500 0.56077300

**
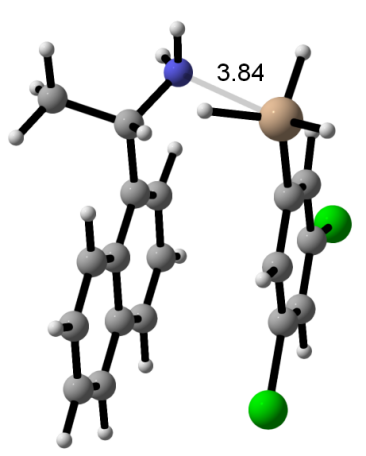
**

**TS1(1,3,5-C_6_H_3_Cl_2_SiH_3_)**

**G_gas_ = -1960.917329 a.u.**

**E_gas_ = -1961.18374811 a.u.**

Si 2.28480700 -1.60830800 2.53209000

H 2.07844400 -2.72616300 3.49876600

C 1.03176300 -1.71004600 1.12794000

C 1.46694300 -1.69911400 -0.20687200

C -0.34587500 -1.78862500 1.39983800

C 0.53129700 -1.77947000 -1.24118400

H 2.52199900 -1.58404700 -0.45401500

C -1.26034500 -1.85158700 0.34762100

H -0.72573400 -1.79112100 2.42368400

C -0.83784200 -1.85088800 -0.98440400

N 3.21671000 1.11735600 -0.01322500

H 3.59841100 1.67130300 -0.78196000

H 3.94495200 1.11076600 0.70130900

C 2.01608100 1.75769900 0.53250000

H 1.77814700 1.19857900 1.44967800

C 2.23771200 3.23371300 0.92216400

H 2.50532300 3.82228600 0.02890800

H 1.33334200 3.68438700 1.35922700

H 3.05739900 3.32252400 1.65475500

C 0.81782200 1.62907500 -0.40036400

C -0.51882800 1.65839600 0.13043100

C 0.99249700 1.52007600 -1.76693100

C -0.80392200 1.73865300 1.52510600

C -1.63282700 1.57710100 -0.77508200

C -0.10584700 1.44918100 -2.65741700

H 1.99858600 1.44615300 -2.18169400

C -2.10062900 1.74017000 1.99739300

H 0.01435600 1.78973700 2.24387900

C -2.95707600 1.58371100 -0.25548200

C -1.39268100 1.47831800 -2.17386600

H 0.08028800 1.35015300 -3.72903700

C -3.19178700 1.66400100 1.09948600

H -2.28637800 1.79429400 3.07313700

H -3.79248200 1.51032100 -0.95703100

H -2.24707400 1.41662700 -2.85328500

H -4.21456200 1.65606100 1.48372500

H 3.66377700 -1.67775400 1.97402800

H 2.13337000 -0.32738500 3.28710900

H -1.55877300 -1.89338700 -1.79943600

Cl -2.97290300 -1.93637200 0.69425700

Cl 1.08664400 -1.78014000 -2.90035700

**
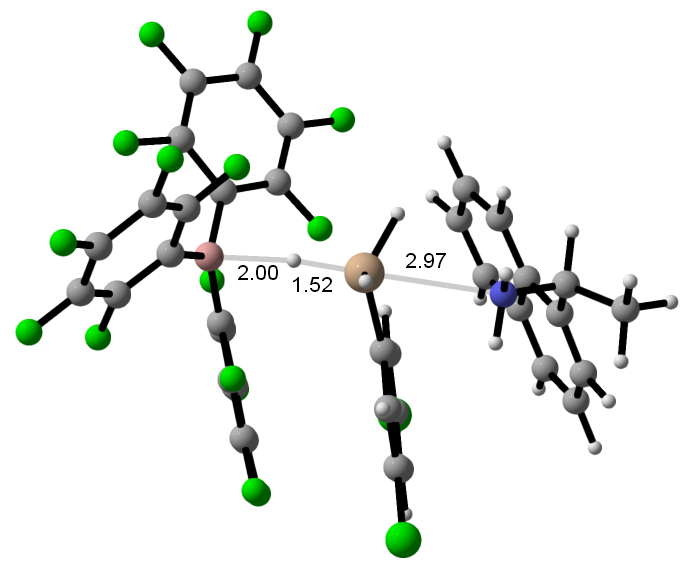
**

**TS2(1,3,5-C_6_H_3_Cl_2_SiH_3_)**

**G_gas_ = -4167.474934 a.u.**

**E_gas_ = -4167.86565512 a.u.**

Si 0.31261900 0.04372100 1.87581500

H -0.94950800 0.01432000 1.03589800

C 1.58791000 -0.84267300 0.80781700

C 1.94694400 -2.16330100 1.12959300

C 2.10697600 -0.26181400 -0.36115000

C 2.81615500 -2.87733000 0.30097100

H 1.54308000 -2.65826200 2.01511300

C 2.94160700 -1.01106300 -1.19162400

H 1.86096400 0.75808900 -0.64747100

C 3.31793300 -2.31758700 -0.87564100

B -2.26203100 0.00368000 -0.46812800

C -3.32758600 -0.77448000 0.40639900

C -3.68360600 -0.33814100 1.69017900

C -3.98895200 -1.91956200 -0.05655200

C -4.61624500 -1.00089500 2.48673300

C -4.93938700 -2.60349600 0.70320900

C -5.25259000 -2.13935800 1.98331600

C -2.39454000 1.58099700 -0.56617700

C -3.64052500 2.22520800 -0.51727900

C -1.28428200 2.42823200 -0.69227000

C -3.78280800 3.61355400 -0.58506800

C -1.38165600 3.81609100 -0.75093700

C -2.64406000 4.41379400 -0.69580200

C -1.28246200 -0.79549200 -1.41084100

C -0.81511200 -0.30243700 -2.64073400

C -0.78304200 -2.05686800 -1.04075500

C 0.09001300 -0.99610400 -3.44592700

C 0.11053800 -2.78454200 -1.82090800

C 0.55728900 -2.24345600 -3.02794700

N 2.72052400 0.10009500 3.61551700

H 2.97125100 -0.85191300 3.34131300

H 2.30303200 0.03206100 4.54590700

C 3.94708600 0.92191000 3.67069200

H 3.63005100 1.93467100 3.96741900

C 4.93185700 0.43981200 4.74498900

H 5.22156400 -0.61182200 4.58943300

H 5.84847300 1.04993100 4.75885400

H 4.46222700 0.50549300 5.73980400

C 4.54290200 1.01560700 2.26858900

C 3.98407400 1.92336500 1.30232600

C 5.58492100 0.19138300 1.88350600

C 2.89049100 2.78900800 1.59047100

C 4.52281400 1.94862200 -0.03028000

C 6.11984000 0.22362400 0.57161800

H 6.01800400 -0.51062800 2.59779600

C 2.33568400 3.59677900 0.62015300

H 2.45868300 2.79424600 2.59013100

C 3.93336300 2.79955900 -1.00738500

C 5.60392200 1.08773200 -0.36497700

H 6.94189900 -0.44752000 0.31116100

C 2.85298500 3.59708400 -0.69754800

H 1.47835200 4.22878900 0.85799200

H 4.33943900 2.78671000 -2.02157100

H 5.99701300 1.10643200 -1.38379300

H 2.38411800 4.22263700 -1.46007000

H -0.00690300 -0.76196900 3.08281800

H 0.52595700 1.47731200 2.14787300

F -5.54951100 -3.68226600 0.22177500

F -6.15083100 -2.77817700 2.72057200

F -4.91020800 -0.56059000 3.70688400

F -3.10349900 0.75088000 2.21141400

F -3.73216000 -2.39826300 -1.27835400

F -1.12512600 -2.59624600 0.13516500

F 0.57262600 -3.96193200 -1.41204000

F 1.44177400 -2.90451000 -3.75945600

F 0.51068500 -0.48049300 -4.59557700

F -1.24093100 0.87612800 -3.10582400

F -0.05172900 1.90826300 -0.74788600

F -0.29318700 4.57941600 -0.85504000

F -2.75816200 5.73263100 -0.75555900

F -4.98640000 4.17564200 -0.54487300

F -4.76945200 1.51665300 -0.42344100

H 3.96399600 -2.89136100 -1.53866000

Cl 3.26074300 -4.51298800 0.72247500

Cl 3.48998700 -0.32261000 -2.70504800

**
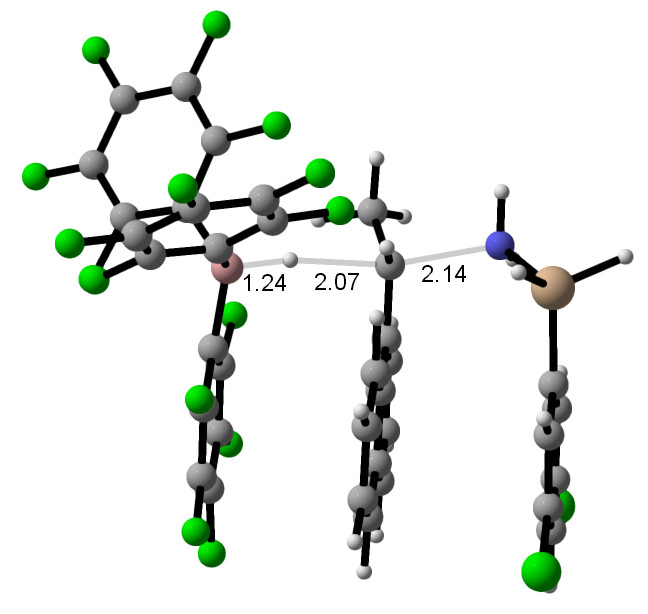
**

**TS3(1,3,5-C_6_H_3_Cl_2_SiH_3_)**

**G_gas_ = -4167.451342 a.u.**

**E_gas_ = -4167.84085985 a.u.**

C 0.45736500 -0.68266300 -2.06730300

H 0.11101400 0.34118200 -2.13826800

C 1.36109500 -1.02765200 -0.98435100

C 1.81206000 -0.03089500 -0.04063900

C 1.79029400 -2.35023600 -0.83929400

C 1.39724900 1.32615500 -0.07018200

C 2.69405500 -0.44124800 1.01574500

C 2.66874800 -2.73551400 0.18542500

H 1.43026100 -3.11524800 -1.52790900

C 1.81809900 2.22155900 0.89325000

H 0.70870000 1.67346600 -0.83476500

C 3.11418200 0.50715900 1.98823800

C 3.10808500 -1.79585800 1.09758200

H 2.98263600 -3.77647500 0.27055600

C 2.68362700 1.81374400 1.93388400

H 1.45636000 3.25144400 0.86094300

H 3.76928800 0.17298200 2.79599600

H 3.77346800 -2.09220700 1.91197500

H 2.99772400 2.52922500 2.69557600

C -0.37914200 -1.71073600 -2.76959700

H 0.22258200 -2.51607600 -3.21553200

H -1.03784100 -2.17693100 -2.02030900

H -1.01668200 -1.25016900 -3.53343900

N 1.79254800 -0.27946600 -3.68508700

H 2.25058900 -1.18722100 -3.78750000

Si 2.93981200 1.10493400 -3.52522700

H 3.58600400 1.33156200 -4.84932000

H 2.12604600 2.28377200 -3.14365700

C 4.18630800 0.66653700 -2.19706000

C 4.69640300 -0.63664600 -2.07838500

C 4.60306400 1.65328400 -1.28802700

C 5.59468500 -0.94167400 -1.05334100

C 5.51603900 1.32975100 -0.28102600

C 6.02068600 0.03378900 -0.14967400

B -1.92641700 -0.02860800 0.09511500

C -1.09764600 -0.57243100 1.38175500

C -0.67972000 -1.90799600 1.40167600

C -0.57399600 0.21276200 2.41365000

C 0.17281900 -2.44791000 2.36087600

C 0.28761000 -0.28239800 3.39809000

C 0.66742000 -1.62169400 3.37051200

C -2.39497700 1.53127000 0.11214400

C -3.30008700 1.99124100 1.07324200

C -2.01588800 2.47207500 -0.84180100

C -3.77826000 3.30117300 1.11695800

C -2.46339100 3.79662700 -0.84448800

C -3.35596900 4.21204900 0.14350100

C -3.19132900 -0.94375200 -0.37619400

C -3.60883300 -0.89348100 -1.70930500

C -3.94263800 -1.78336900 0.45112000

C -4.67942700 -1.63107300 -2.21772300

C -5.02623800 -2.53796900 -0.00969500

C -5.39765000 -2.46022600 -1.35385300

F -1.15467900 2.14826000 -1.84301600

F -2.05212500 4.65813600 -1.77964300

F -3.79967200 5.46844900 0.16230200

F -4.63060000 3.69402000 2.06361300

F -3.72913100 1.14749200 2.02186500

F -2.95676000 -0.09830900 -2.58639900

F -5.02046400 -1.55133200 -3.50708700

F -6.42630900 -3.17430000 -1.80955700

F -5.70953600 -3.32956100 0.81779400

F -3.63844300 -1.90681200 1.74949300

F -0.82800800 1.52954000 2.47860500

F 0.80543800 0.53291300 4.32064600

F 1.55462500 -2.09137300 4.25137000

F 0.58130900 -3.71812400 2.27991800

F -1.05578400 -2.74273100 0.40760700

H -1.09942300 -0.14068500 -0.81638800

H 1.14560700 -0.18200400 -4.47164600

H 4.20561300 2.66854900 -1.32960700

H 4.39702200 -1.44023800 -2.75395500

H 6.71572200 -0.21456100 0.65186700

Cl 6.17198400 -2.57940400 -0.87532500

Cl 6.01361900 2.55416400 0.85572000

**
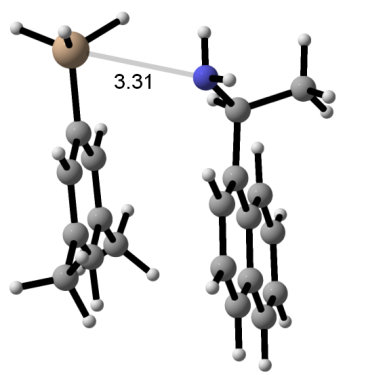
**

**TS1(1,3,5-C_6_H_3_Me_2_SiH_3_)**

**G_gas_ = -1120.488723 a.u.**

**E_gas_ = -1120.82999783 a.u.**

Si -3.79470700 -0.62498400 1.06953000

H -4.70060200 -0.10633700 2.14318500

C -2.38159100 0.57328400 0.76273900

C -2.31034400 1.30178600 -0.43270300

C -1.36506400 0.75180100 1.71913000

C -1.25649500 2.19328800 -0.68579500

H -3.07481900 1.15869900 -1.20257400

C -0.29798600 1.63030100 1.49646800

H -1.38923700 0.18723900 2.65772700

C -0.26165300 2.33976600 0.28619400

N -1.81824300 -2.29842000 -0.98679300

H -1.74459500 -2.61420100 -1.95557500

H -2.41700100 -2.98075100 -0.52262600

C -0.50082900 -2.30479000 -0.34750600

H -0.68806400 -2.09541600 0.71925000

C 0.19637100 -3.67787300 -0.46928300

H 0.34960300 -3.92259600 -1.53362200

H 1.18128400 -3.70122800 0.01974600

H -0.43010300 -4.46674100 -0.02080000

C 0.40219800 -1.19505700 -0.87707500

C 1.57582300 -0.79913100 -0.14466700

C 0.13687800 -0.58327100 -2.08790400

C 1.90904400 -1.32168700 1.13971900

C 2.45773200 0.18500400 -0.71182700

C 1.00625100 0.38588900 -2.64408000

H -0.77847900 -0.83181800 -2.62553900

C 3.05021900 -0.92727500 1.80775400

H 1.24478200 -2.04001100 1.62030600

C 3.62680400 0.57141000 0.00326900

C 2.14816200 0.75938600 -1.97555900

H 0.75670200 0.83939800 -3.60636900

C 3.92703700 0.02391800 1.23220600

H 3.27352600 -1.34406700 2.79311400

H 4.28863300 1.31762200 -0.44536900

H 2.82710100 1.50455100 -2.39886700

H 4.82919300 0.32945200 1.76795600

H -4.60645400 -0.81439400 -0.16484900

H -3.29002800 -1.94669300 1.55390800

H 0.58087600 3.01038900 0.09077500

C 0.79773700 1.81972100 2.51526800

H 1.78921300 1.70394500 2.05153500

H 0.75561500 2.82978800 2.95804900

H 0.72365700 1.08929300 3.33418400

C -1.20191000 2.97211900 -1.97694900

H -0.17494100 3.28744200 -2.21410200

H -1.57588200 2.37279300 -2.82187400

H -1.82668500 3.88081600 -1.91984000

**
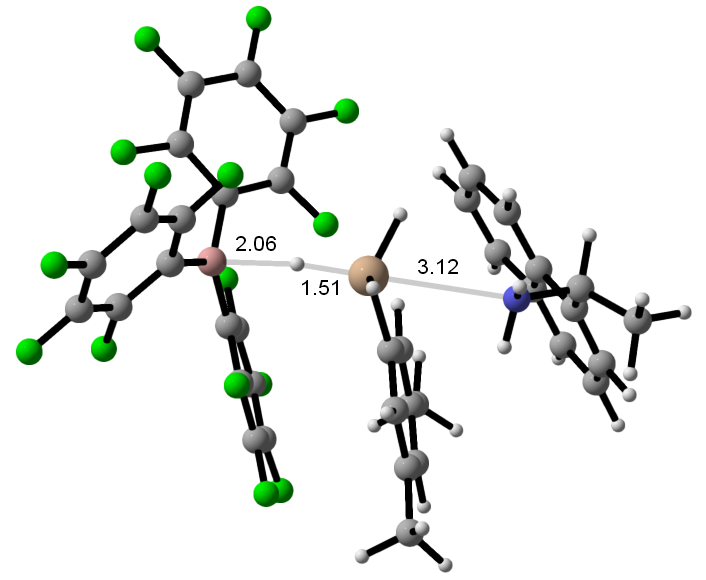
**

**TS2(1,3,5-C_6_H_3_Me_2_SiH_3_)**

**G_gas_ = -3327.049851 a.u.**

**E_gas_ = -3327.51399756 a.u.**

Si 0.45216800 -0.07250100 1.84762400

H -0.86909200 -0.02942200 1.10894500

C 1.62046400 -1.07496000 0.77194900

C 1.85093400 -2.42459300 1.09449800

C 2.21626400 -0.55136600 -0.38890800

C 2.65753400 -3.24477900 0.29154600

H 1.38446200 -2.85980500 1.98437300

C 3.00203600 -1.35365100 -1.22730900

H 2.07222400 0.49563600 -0.65580100

C 3.21843300 -2.69183400 -0.86663400

B -2.19839200 0.04996500 -0.46618200

C -3.33148700 -0.61259000 0.41709700

C -3.66255000 -0.11232900 1.68438300

C -4.08288600 -1.70991700 -0.02386800

C -4.65587700 -0.67341800 2.48612400

C -5.09496200 -2.29099200 0.74126100

C -5.38060300 -1.76766000 2.00494200

C -2.19316700 1.62972600 -0.59364600

C -3.38244400 2.37540300 -0.57202900

C -1.01410200 2.38047300 -0.71220200

C -3.40928200 3.76962500 -0.65460600

C -0.99743700 3.77145400 -0.78197700

C -2.20664300 4.47182100 -0.75324700

C -1.27107700 -0.85212900 -1.36507300

C -0.72501700 -0.42458900 -2.58714800

C -0.89893400 -2.14781800 -0.96120900

C 0.13319700 -1.21465600 -3.35206100

C -0.06215100 -2.97209100 -1.70748100

C 0.47103500 -2.49178500 -2.90326200

N 3.14676300 -0.15427800 3.42625600

H 3.23316800 -1.08571900 3.01633000

H 2.80303000 -0.28043500 4.37963700

C 4.46640900 0.50112000 3.44220700

H 4.30461800 1.52683500 3.81743300

C 5.44811200 -0.17002900 4.41275900

H 5.59724600 -1.23362200 4.16598500

H 6.43131900 0.32641100 4.41235900

H 5.04551700 -0.12523200 5.43761800

C 4.98100500 0.62366700 2.00948800

C 4.37908200 1.57008800 1.10807900

C 6.00123500 -0.18418000 1.54096000

C 3.30340100 2.42269400 1.48671800

C 4.85991400 1.65835700 -0.24307700

C 6.47683900 -0.09348300 0.20886700

H 6.46758300 -0.91504200 2.20276800

C 2.71535300 3.28322400 0.58356300

H 2.90870700 2.36567100 2.49952800

C 4.23989600 2.56622800 -1.14751300

C 5.92453800 0.81444000 -0.66323900

H 7.28743800 -0.75031700 -0.11649800

C 3.18023300 3.35373600 -0.75159000

H 1.87132600 3.90433400 0.88852100

H 4.61404100 2.61761400 -2.17383100

H 6.28487800 0.89118500 -1.69232100

H 2.68867300 4.02538000 -1.45832100

H 0.16970000 -0.79768400 3.11685700

H 0.79006600 1.34791800 2.07298800

F -5.78924400 -3.32747800 0.28051700

F -6.33699800 -2.30874000 2.74746000

F -4.92502900 -0.17684400 3.69025300

F -3.00105500 0.93877100 2.18296100

F -3.85505600 -2.24095600 -1.23003900

F -1.31975700 -2.63474100 0.21000200

F 0.26094500 -4.18803700 -1.27646900

F 1.32138600 -3.23468200 -3.59937500

F 0.65781700 -0.75453800 -4.48631800

F -1.02522400 0.77894600 -3.08430000

F 0.17127400 1.76163300 -0.74757000

F 0.15102200 4.44306400 -0.87394900

F -2.21014100 5.79498100 -0.82605300

F -4.56306900 4.42931300 -0.64022600

F -4.56755700 1.76323300 -0.49035400

C 2.85723500 -4.70252700 0.62250600

H 2.12711400 -5.31802800 0.07043900

H 3.86314800 -5.04953700 0.33932100

H 2.71351400 -4.90145300 1.69550900

H 3.82935600 -3.32529300 -1.51758400

C 3.56136200 -0.81843800 -2.52171500

H 3.03619200 -1.26223100 -3.38354400

H 3.45145500 0.27241800 -2.58669400

H 4.62942800 -1.06320200 -2.62740500

**
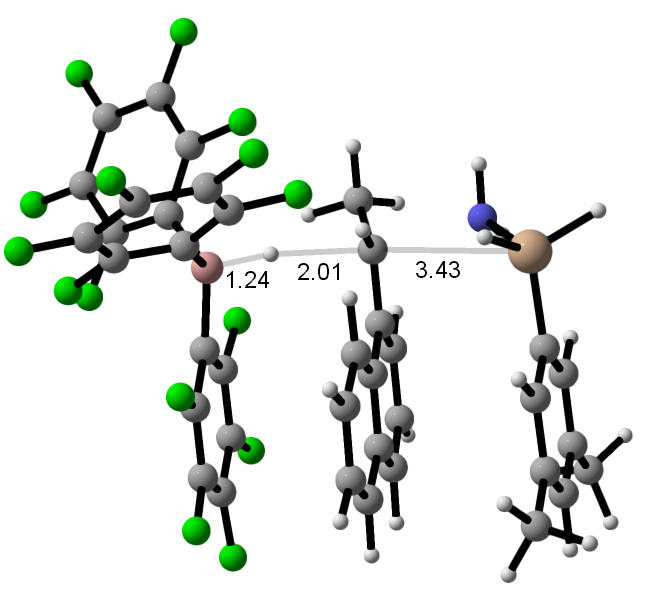
**

**TS3(1,3,5-C_6_H_3_Me_2_SiH_3_)**

**Ggas = -3327.028902 a.u.**

**Egas = -3327.48984186 a.u.**

C -0.67376100 -0.44696800 2.08687500

H -0.36527100 0.59046600 2.08691200

C -1.58158800 -0.90105700 1.05178700

C -2.04189700 -0.00612800 0.01468700

C -1.99722400 -2.23563600 1.03647500

C -1.62989400 1.34761900 -0.09440300

C -2.91667400 -0.52619300 -0.99732900

C -2.87133300 -2.72715100 0.05409100

H -1.62617500 -2.92696100 1.79350700

C -2.03751700 2.13382100 -1.15362100

H -0.95275000 1.77625900 0.63839400

C -3.32703900 0.31220200 -2.06970300

C -3.32111000 -1.88489400 -0.94455000

H -3.16172100 -3.77897400 0.06295700

C -2.88959700 1.61501200 -2.15547000

H -1.67449000 3.16107900 -1.22816300

H -3.97011100 -0.10949800 -2.84577600

H -3.97571800 -2.26662800 -1.73190800

H -3.18382500 2.24075300 -3.00036300

C 0.14732300 -1.39292900 2.91160800

H -0.46921000 -2.12799700 3.44910900

H 0.80460100 -1.95895500 2.23337500

H 0.78349300 -0.84959600 3.61973800

N -2.07336000 0.10152800 3.68595800

H -2.53461300 -0.79835000 3.82942800

Si -3.22028700 1.45947200 3.38057200

H -3.84535600 1.83809700 4.68243700

H -2.40306200 2.59273400 2.87847000

C -4.46474600 0.88009800 2.11614600

C -5.01818600 -0.40882100 2.17193800

C -4.85410600 1.73830900 1.07230000

C -5.93153100 -0.85465600 1.20510800

C -5.77609600 1.33082500 0.10042300

C -6.29812500 0.03098000 0.18391600

B 1.69113500 -0.04266700 -0.08774700

C 0.87185000 -0.71050300 -1.31945600

C 0.45347300 -2.04205000 -1.21365300

C 0.35967000 -0.02936200 -2.42866300

C -0.38921500 -2.67268600 -2.12483900

C -0.49179300 -0.61833400 -3.36902700

C -0.87371800 -1.94830300 -3.21431000

C 2.16755900 1.50494700 -0.26032600

C 3.08373300 1.85399700 -1.25703400

C 1.78925300 2.54354200 0.58644600

C 3.57326500 3.14820500 -1.43477900

C 2.24826200 3.85748400 0.45280900

C 3.15143500 4.15967100 -0.56618300

C 2.94661400 -0.90679000 0.49073700

C 3.36613900 -0.69909300 1.80812000

C 3.69304800 -1.84339100 -0.23039900

C 4.43195800 -1.37777200 2.40146800

C 4.77183500 -2.54441700 0.31781700

C 5.14419600 -2.30910600 1.64318800

F 0.91992200 2.33250500 1.60846300

F 1.83812200 4.81576800 1.28851300

F 3.60575100 5.40368800 -0.71373700

F 4.43581200 3.43237900 -2.41073000

F 3.51253700 0.91139400 -2.10812900

F 2.72081300 0.20060000 2.58178600

F 4.77455700 -1.14614800 3.67163800

F 6.16821200 -2.97039100 2.18121900

F 5.45022400 -3.43284100 -0.40934300

F 3.39056900 -2.11717900 -1.50595300

F 0.61725600 1.27388300 -2.62072100

F -0.99853100 0.10264700 -4.37282900

F -1.75193500 -2.50169800 -4.05459000

F -0.79825700 -3.92970800 -1.92611300

F 0.82105200 -2.77657000 -0.14116900

H 0.84763600 -0.05999300 0.82584200

H -1.44516800 0.25126400 4.47841300

H -4.41526700 2.73691500 0.99337000

H -4.73820800 -1.10325300 2.97185600

H -7.00636100 -0.30511400 -0.58011800

C -6.46263200 -2.26559600 1.24505300

H -5.71934300 -2.96654300 0.82741800

H -6.67338500 -2.59047800 2.27607100

H -7.38666400 -2.37176200 0.65757700

C -6.20614300 2.26092400 -1.00590600

H -7.19367800 2.70261200 -0.78705000

H -5.49154900 3.08588900 -1.13960500

H -6.29100800 1.72829000 -1.96563300

**int12**

**G_gas_ = -1999.274130 a.u.**

**E_gas_ = -1999.55079631 a.u.**

F 0.65998900 -1.57427900 -2.08058700

F -0.95128000 -3.74829000 -2.19039300

F -1.27960100 -0.36926400 2.06838200

F 2.24308400 -2.09287400 0.67340200

F 2.61571100 2.56355700 -0.21155800

F -2.90292500 -2.49646400 1.92536700

F 5.27261900 2.40192500 0.15553000

F -2.75337800 -4.20944300 -0.18415600

F 4.88115700 -2.22222700 1.03601000

F 6.43361100 0.00602700 0.78587900

C -0.21696800 -0.90459700 0.01544800

C 2.29514400 0.24354200 0.20898900

C -0.19173700 -1.80398300 -1.04989100

C -1.13794000 -1.18993700 1.02457800

C -1.99091000 -2.29699500 0.97501300

C -1.92965600 -3.16613400 -0.11407900

C -1.01417700 -2.92512400 -1.14407700

C 2.93830000 -0.95701600 0.53040700

C 4.52472600 1.30679600 0.28195900

C 4.32060000 -1.05404900 0.73047100

C 3.14317300 1.35056900 0.09723200

C 5.11886100 0.08388100 0.60355600

H 0.27746000 1.21767900 0.86585600

N 0.47719200 1.18702600 -1.41607600

H 0.98490600 2.07415500 -1.34894000

H 0.96186800 0.63339600 -2.12928800

C -0.92739000 1.44055000 -1.89381100

H -1.34671900 0.44320300 -2.05506500

C -1.72696800 2.16793100 -0.82598900

C -2.87546900 1.57103900 -0.20542100

C -1.30781700 3.42365300 -0.42127100

C -3.40068000 0.29441500 -0.56270400

C -3.53368400 2.29008700 0.85417300

C -1.96648100 4.13311100 0.60748600

H -0.42912500 3.88246200 -0.88441700

C -4.47935500 -0.25206300 0.09934600

H -2.93025300 -0.28850900 -1.35459200

C -4.64979100 1.69981400 1.51182700

C -3.05507400 3.57195800 1.23694200

H -1.59963600 5.11801800 0.90505300

C -5.11179000 0.45379000 1.15111100

H -4.83786400 -1.24560100 -0.17983000

H -5.13160300 2.25574000 2.32049800

H -3.56505700 4.10454600 2.04392700

H -5.96049200 0.00607000 1.67322600

C -0.90009500 2.16948700 -3.23885900

H -1.92661900 2.30203500 -3.61006000

H -0.44583400 3.16901500 -3.14945600

H -0.33545000 1.59543400 -3.99215000

B 0.68485800 0.43815700 0.03445400

**int13**

**G_gas_ = -1249.275577 a.u.**

**E_gas_ = -1249.38902395 a.u.**

F -4.18491700 0.50890500 -1.09399200

F -3.26703000 -2.05140400 -0.97962400

F -0.89594400 -2.58838700 0.18681300

F -0.33638100 2.01626700 1.14486300

F -2.71567700 2.54193300 -0.02887000

C -0.54088300 -0.30686100 0.72926800

C -3.02146400 0.24915000 -0.51184900

C -1.04567900 0.99351100 0.64146900

C -1.31906500 -1.32066800 0.16244500

C -2.54849400 -1.06478700 -0.45202800

C -2.26710400 1.29186100 0.03589700

Si 1.14664100 -0.64181900 1.54529500

H 1.18837400 0.15977800 2.79866100

H 1.23533100 -2.08894200 1.86497000

C 2.53277300 -0.16951000 0.36846200

C 2.62312900 1.13265000 -0.16372900

C 3.50567800 -1.11417200 -0.00785400

C 3.65443100 1.47735700 -1.04035800

H 1.87882700 1.88675900 0.10691600

C 4.53963500 -0.77023600 -0.88516200

H 3.45821200 -2.13440800 0.38456900

C 4.61506900 0.52600400 -1.40204800

H 3.70957800 2.49195800 -1.44360400

H 5.28630500 -1.51734500 -1.16684700

H 5.42178600 0.79604500 -2.08859400

**TS11**

**G_gas_ = -3248.459521 a.u.**

**E_gas_ = -3248.87394476 a.u.**

F -0.01039200 -2.18754700 1.13349600

F 5.68062100 1.90243200 2.71884600

F 5.98710500 0.87396500 0.21892800

F 3.89537600 0.62237300 -1.38507100

F 1.13134200 2.51900400 1.99247500

F -2.10713000 -2.22527900 2.72292300

F -0.74824500 2.43467400 0.32088000

F 3.22935600 2.70297600 3.57799200

F 2.58624000 -1.10559700 1.76410200

F 1.48218100 -1.24901600 -2.84075600

F -2.83680800 2.37626500 1.96387600

F 2.93611700 -3.48100800 -3.11674500

F -3.54959300 0.03913100 3.21002500

F 4.07010600 -3.34708500 1.46885900

F 4.24883300 -4.54849200 -0.97553300

C 2.35222400 1.51001000 0.20656400

C 4.63421800 1.78384600 1.92488500

C 2.27823100 2.06735600 1.50790300

C 3.67226200 1.14599400 -0.18281300

C 4.78734300 1.25721700 0.63841400

C -0.18725400 0.16291000 0.70797900

C 3.37200200 2.18874300 2.36285500

C 1.93756200 -1.11428000 -0.50788700

C -0.61752900 -1.01012700 1.34591100

C -1.01168100 1.26607600 0.94355400

C -2.11586300 1.27064800 1.79188500

C -2.48095700 0.08480800 2.42523900

C -1.72839900 -1.06452200 2.18990700

C 2.64540900 -1.68531000 0.55281400

C 2.84007400 -2.90479900 -1.91955000

C 3.42076900 -2.83514000 0.42606600

C 2.07794600 -1.74591200 -1.74281900

C 3.51600900 -3.45047800 -0.82715700

Si 1.38267000 2.48294000 -1.43547800

H 0.69534100 0.59057700 -1.41179400

N -1.27436600 -1.37942300 -1.94410600

H -1.47034600 -0.86928300 -2.80610800

H -0.65050300 -2.13849400 -2.21006000

C -2.49838100 -1.91891300 -1.35556100

H -2.16804200 -2.52590000 -0.49568300

C -3.40622800 -0.81427400 -0.82401500

C -4.41637000 -1.11090300 0.15670900

C -3.29065200 0.48274500 -1.29169200

C -4.56626700 -2.39429700 0.76036900

C -5.29431700 -0.05991300 0.59810400

C -4.15586700 1.51458500 -0.85386400

H -2.50411800 0.74178400 -1.99856800

C -5.52643200 -2.63069500 1.72221900

H -3.89798800 -3.20778600 0.47840400

C -6.28136800 -0.34085100 1.58335100

C -5.14264100 1.24989500 0.06586300

H -4.01354200 2.52389200 -1.24404800

C -6.40062500 -1.59701500 2.13562900

H -5.60572700 -3.62191800 2.17548300

H -6.93832200 0.47071400 1.90792900

H -5.80662200 2.04338200 0.41811500

H -7.15720300 -1.79541600 2.89875900

C -3.27253900 -2.83423300 -2.32793500

H -4.18128800 -3.25654800 -1.87352100

H -3.58014600 -2.25622500 -3.21501200

H -2.63338100 -3.66813400 -2.66252900

H 1.47225100 3.82194600 -0.78427300

H 2.50003300 2.21450000 -2.36551700

C -0.17445300 2.54048400 -2.49756700

C -1.14863700 3.53669800 -2.31886800

C -0.32355800 1.63680000 -3.56804100

C -2.24625300 3.62593200 -3.18129100

H -1.06156600 4.24219600 -1.48885600

C -1.41865700 1.72399700 -4.43016300

H 0.41673600 0.84666300 -3.72238500

C -2.38510500 2.71809200 -4.23486500

H -2.99900100 4.40301900 -3.02564000

H -1.52082700 1.01571800 -5.25662500

H -3.24755500 2.78216000 -4.90301300

B 1.05898400 0.21314900 -0.31421300

**int2-4**

**G_gas_ = -3248.510808 a.u.**

**E_gas_ = -3248.92541987 a.u.**

Si -2.35565700 1.64187100 1.66339800

H 0.09819500 -0.18413800 0.47692500

C -2.93173400 1.50422300 -0.08679900

C -3.68989600 2.54481000 -0.66103300

C -2.52187300 0.42852800 -0.89555700

C -4.03445000 2.50349500 -2.01345600

H -3.98162500 3.41513300 -0.06533400

C -2.85671600 0.39575400 -2.24991200

H -1.90350900 -0.36884700 -0.48575200

C -3.61201900 1.43147000 -2.80828600

H -4.60724900 3.32210800 -2.45556500

H -2.49789300 -0.43179400 -2.86447000

H -3.85938800 1.41428200 -3.87279800

B 1.14485700 0.11383000 -0.08712000

C 2.14078000 0.74540600 1.04282000

C 1.89900600 0.64337600 2.41123700

C 3.27995400 1.47670200 0.69504400

C 2.71319300 1.22672000 3.38667100

C 4.12798200 2.07527700 1.62827700

C 3.83778000 1.94988700 2.98982000

C 1.66904900 -1.28047600 -0.76680600

C 2.90077000 -1.90303800 -0.54448200

C 0.79959300 -2.00746900 -1.58444600

C 3.24468600 -3.15249200 -1.07959600

C 1.09062000 -3.25050400 -2.13781800

C 2.33071300 -3.83646000 -1.88006000

C 0.72878700 1.28213500 -1.16818600

C 0.93794000 1.26496300 -2.55131800

C 0.01657200 2.39289800 -0.72214600

C 0.41316500 2.22793500 -3.42152400

C -0.52646800 3.38264600 -1.53626500

C -0.32946100 3.29503400 -2.91314000

N -3.86017300 0.90698500 2.70152000

H -4.70440700 1.00906900 2.12544900

H -4.01625900 1.49632600 3.52764500

C -3.73649000 -0.54404300 3.14305400

H -2.80105900 -0.57635100 3.71272600

C -4.88873800 -0.90669700 4.07961100

H -5.86383300 -0.84040000 3.57245000

H -4.76243700 -1.94364400 4.42249700

H -4.90851200 -0.25463000 4.96886400

C -3.63086100 -1.43949500 1.91961200

C -2.44560800 -2.20537900 1.64747800

C -4.70855900 -1.50460400 1.04986100

C -1.27732000 -2.18999900 2.46138600

C -2.42477900 -3.02529800 0.46186700

C -4.67264000 -2.29325400 -0.12058700

H -5.61732200 -0.92909400 1.25426100

C -0.16541700 -2.93522200 2.13319800

H -1.21939500 -1.55375700 3.34382600

C -1.26465500 -3.78855100 0.15864300

C -3.55007500 -3.03907700 -0.40467900

H -5.53209900 -2.30230700 -0.79389500

C -0.15860100 -3.74997900 0.97782100

H 0.72746400 -2.86728700 2.75708400

H -1.24984200 -4.37401000 -0.76319800

H -3.50446500 -3.64964600 -1.30987800

H 0.73831800 -4.31563800 0.71768100

H -2.42071600 3.01631500 2.21178700

H -1.29655500 0.77899300 2.19480700

F 5.20623100 2.76051600 1.24298800

F 4.63032100 2.51522700 3.90168500

F 2.42434400 1.10079900 4.68766700

F 0.82738500 -0.04321100 2.87723900

F 3.60927000 1.60959200 -0.59880400

F -0.25831000 2.52611200 0.61597700

F -1.25515600 4.37792000 -1.01709800

F -0.85302200 4.20962500 -3.73030000

F 0.61821100 2.13982800 -4.73664500

F 1.64706000 0.28472700 -3.12678200

F -0.43333500 -1.52188300 -1.85133900

F 0.17311900 -3.91283600 -2.85745200

F 2.63220600 -5.03317400 -2.38664000

F 4.43834000 -3.69446200 -0.82777500

F 3.84148300 -1.32769500 0.22066000

**Int6-8**

**G_gas_ = -3769.969512 a.u.**

**E_gas_ = -3770.47547016 a.u.**

Si 1.96060700 -1.59828200 0.59575300

H -0.53316800 0.16294400 -0.14072100

C 0.61413900 -2.82259400 0.93058600

C 0.79880500 -3.80313500 1.92530200

C -0.65096400 -2.69224600 0.32833600

C -0.25519400 -4.63943800 2.29973600

H 1.75796100 -3.89453300 2.44456300

C -1.70662500 -3.51866300 0.71347800

H -0.83249000 -1.91809500 -0.41509600

C -1.50989300 -4.49249200 1.69770800

H -0.10693000 -5.38572400 3.08385300

H -2.68654300 -3.37743400 0.25280100

H -2.34135100 -5.12867600 2.01177400

B -1.13758400 1.00162100 0.52239900

C -0.03079700 2.18350500 0.75639900

C 0.72429900 2.62557500 -0.33378800

C 0.30449700 2.77650400 1.97608500

C 1.74446100 3.57090900 -0.24637900

C 1.34414000 3.70230200 2.12291500

C 2.06480100 4.10619000 1.00026700

C -2.37308100 1.44699800 -0.44500500

C -2.76719900 2.75998200 -0.70720400

C -3.07678400 0.47000000 -1.15325700

C -3.76319000 3.09471300 -1.63298300

C -4.07574300 0.75053900 -2.08165600

C -4.41832200 2.08064200 -2.33155500

C -1.58608500 0.18176500 1.87010800

C -2.89413300 -0.13134500 2.25411600

C -0.61011100 -0.43734900 2.64912500

C -3.20086800 -1.03529100 3.27994600

C -0.85198300 -1.33931000 3.67893900

C -2.17416700 -1.65135000 3.99481400

N 3.23355800 -2.38506000 -0.66712800

H 3.16365100 -3.40125100 -0.52999700

C 2.82630100 -2.09160200 -2.11554700

H 2.79769600 -0.99744800 -2.17056400

C 3.84635200 -2.60219100 -3.13836300

H 4.01830600 -3.68474200 -3.03198900

H 3.43945000 -2.42785300 -4.14470800

H 4.81537300 -2.08705000 -3.07581400

C 1.44662900 -2.66680500 -2.40744900

C 0.38562600 -1.84970900 -2.92625700

C 1.24581900 -4.02817600 -2.24040100

C 0.48446900 -0.44209000 -3.12171600

C -0.86356300 -2.48728900 -3.26133700

C 0.00992800 -4.64103400 -2.53628000

H 2.05556000 -4.66239500 -1.86416100

C -0.56192000 0.28120900 -3.65360200

H 1.37893500 0.10209900 -2.82014800

C -1.92371000 -1.70827300 -3.80110500

C -1.02244200 -3.88114400 -3.04065100

H -0.11890800 -5.71160400 -2.36471100

C -1.77164100 -0.35702500 -4.01372000

H -0.46433500 1.36263900 -3.75887800

H -2.87370300 -2.19682700 -4.02817500

H -1.98387600 -4.34277300 -3.27960700

H -2.59575200 0.22694300 -4.42638400

H 2.87526100 -1.46563700 1.74816500

H 1.65896500 -0.35952300 -0.12981000

F 1.65894600 4.19971800 3.32203100

F 3.08721200 4.96426900 1.12420900

F 2.43777500 3.94114200 -1.33218500

F 0.48762000 2.12442300 -1.56424100

F -0.36369400 2.46030400 3.09582600

F 0.70819500 -0.21184600 2.36591000

F 0.15635000 -1.92889500 4.33107400

F -2.44886100 -2.53183700 4.95839400

F -4.47081400 -1.31584200 3.57930000

F -3.94885800 0.41183900 1.62798100

F -2.79392000 -0.83610800 -0.95985800

F -4.67619000 -0.23173400 -2.76749500

F -5.35690000 2.37513400 -3.23363300

F -4.09111900 4.37001900 -1.85413000

F -2.18646700 3.78799200 -0.06615500

Si 4.96430600 -1.86871200 -0.14829200

H 5.89353700 -2.51757200 -1.10176700

H 5.10913100 -2.46280100 1.20059600

C 5.00402000 -0.01482900 -0.17989400

C 5.14850500 0.67803600 -1.40128900

C 4.82530000 0.73329500 1.00266500

C 5.10337200 2.07256500 -1.43882500

H 5.29934200 0.12949900 -2.33590800

C 4.78081600 2.12849000 0.96189200

H 4.70598100 0.22688100 1.96403200

C 4.92120800 2.79749900 -0.25703900

H 5.19871500 2.59798900 -2.39155600

H 4.61789500 2.70156300 1.87728100

H 4.85160400 3.88537600 -0.28556800

**TS_d_****_issociation-1_**

**G_gas_ = -3248.505885 a.u.**

**E_gas_ = -3248.92058489 a.u.**

Si -1.40459900 1.36899600 1.93110600

H 0.05472700 0.15627700 0.23859100

C -1.45221400 2.52154000 0.48548200

C -0.84934000 3.79016600 0.59699800

C -2.01313100 2.13599600 -0.74733300

C -0.81144700 4.65551700 -0.49891700

H -0.34895000 4.08688900 1.52193400

C -1.97813000 3.00496500 -1.83780000

H -2.44683900 1.14488900 -0.87090400

C -1.37673100 4.26314600 -1.71581400

H -0.30592800 5.61967300 -0.40833100

H -2.40193600 2.68651400 -2.79298200

H -1.33132100 4.93258500 -2.57864300

B 1.19179400 -0.22279900 -0.04246800

C 1.76505200 -0.90291800 1.33200500

C 0.95533300 -1.78918800 2.04633100

C 3.02159900 -0.67313800 1.89870000

C 1.32308700 -2.39150600 3.25057600

C 3.43938600 -1.25205300 3.10183600

C 2.58423000 -2.12051800 3.78318200

C 1.02488300 -1.33783900 -1.21989400

C 2.00988500 -2.28845400 -1.49931100

C -0.11679700 -1.41169600 -2.01758700

C 1.87783200 -3.26836300 -2.48639000

C -0.29080900 -2.36577000 -3.02292600

C 0.71001300 -3.30988600 -3.25124000

C 1.94108200 1.15123600 -0.50698400

C 2.48056800 1.42284800 -1.76733100

C 1.93992400 2.23976400 0.36610100

C 2.94781700 2.68830800 -2.14566100

C 2.37888900 3.51851100 0.03785800

C 2.89026100 3.74756700 -1.23970500

N -3.20677800 1.56097900 2.72434800

H -3.63421200 2.40305400 2.32081400

H -3.10432600 1.75540600 3.72711400

C -4.15461900 0.38494900 2.53783700

H -3.59724000 -0.48038900 2.91369600

C -5.39896300 0.57003000 3.40616900

H -5.97280800 1.46437400 3.11830100

H -6.05799900 -0.30176200 3.28582300

H -5.13560200 0.65169200 4.47386300

C -4.45006000 0.20959400 1.05601900

C -3.97792600 -0.93076800 0.31801800

C -5.17512000 1.19771000 0.40699500

C -3.21535000 -1.99330000 0.88250000

C -4.26873400 -0.99446200 -1.09291800

C -5.45589800 1.12901400 -0.97513300

H -5.54238500 2.06560700 0.96363000

C -2.76060300 -3.04020000 0.10923600

H -2.94302700 -1.98629400 1.93744800

C -3.78703500 -2.09068700 -1.85880700

C -5.00786000 0.05168900 -1.70741200

H -6.01926500 1.93260100 -1.45378900

C -3.04768000 -3.09331500 -1.27425400

H -2.14427900 -3.81484600 0.56893500

H -3.98273700 -2.10752100 -2.93259900

H -5.21021800 -0.01134000 -2.77963600

H -2.66127100 -3.91283800 -1.88256100

H -0.62988700 1.88123700 3.08128900

H -1.42803500 -0.08717900 1.80046100

F 4.64779500 -0.99041700 3.60331000

F 2.96543900 -2.68099700 4.93198500

F 0.48662100 -3.21914400 3.88889400

F -0.27596200 -2.10570600 1.57820200

F 3.90129300 0.13800900 1.29538900

F 1.43115800 2.08453400 1.61505800

F 2.26478100 4.53126500 0.90838100

F 3.30273000 4.96677900 -1.59217700

F 3.44030700 2.89375700 -3.36897700

F 2.55923300 0.46693700 -2.70578600

F -1.12555600 -0.53159700 -1.85177300

F -1.41023200 -2.39904200 -3.76185300

F 0.55431100 -4.23775000 -4.19732400

F 2.84765600 -4.15728700 -2.71027100

F 3.16465100 -2.27360400 -0.81623700

**TS _association-1_**

**G_gas_ = -3248.531179 a.u.**

**E_gas_ = -3248.94897826 a.u.**

H -0.11027900 1.26951700 -1.27330900

B -0.37026500 -0.85472300 0.00254700

C -1.03805500 -0.79927200 1.48988900

C -1.30025500 -1.94756700 2.24400100

C -1.47411400 0.39266300 2.06567100

C -1.95149200 -1.92504500 3.47921000

C -2.14441300 0.46994800 3.28883700

C -2.38437900 -0.70310300 4.00368500

C 1.24050900 -1.09276400 -0.08111700

C 1.89042900 -0.90269000 -1.30182000

C 2.07414000 -1.49595300 0.96439200

C 3.25451200 -1.09117600 -1.50807300

C 3.44610700 -1.71261600 0.80764500

C 4.04096200 -1.50819600 -0.43550600

C -1.19133200 -1.89302900 -0.95885300

C -2.38268600 -1.49929500 -1.56854500

C -0.79400800 -3.20581800 -1.23220100

C -3.14258100 -2.31752800 -2.40566100

C -1.52106300 -4.06784500 -2.05980700

C -2.70445000 -3.62016400 -2.65085500

F -1.26892800 1.57990400 1.44130700

F -2.56826100 1.64777400 3.76214000

F -3.01935100 -0.66075100 5.17366900

F -2.17275500 -3.04906600 4.15976700

F -0.91128500 -3.14298300 1.78505600

F 1.59146600 -1.67530300 2.20179100

F 4.20396600 -2.05700500 1.85034200

F 5.36243700 -1.62840800 -0.57671800

F 3.81528600 -0.83201400 -2.69238800

F 1.17788300 -0.47942500 -2.38363500

F -2.86140500 -0.24621200 -1.37057700

F 0.33203600 -3.69948300 -0.70234500

F -1.09803000 -5.31052200 -2.29087100

F -3.40766400 -4.42681500 -3.44376300

F -4.26717000 -1.87048300 -2.97023500

H -0.59588000 0.26313700 -0.45949700

N 0.11166900 2.10505200 -1.88680900

H 0.72920800 1.68978200 -2.59681400

Si -1.51542100 2.60213800 -2.69809500

H -1.99622000 1.30002100 -3.20237800

H -1.10321500 3.51000100 -3.79586600

C 0.91353700 3.09056700 -1.04188900

H 0.34969700 3.15691700 -0.10475500

C -2.56608300 3.46380400 -1.43582300

C -3.12333600 2.75840200 -0.35096100

C -2.77107100 4.85811400 -1.51548300

C -3.82193400 3.43489100 0.65159500

H -2.99953100 1.67941100 -0.27503200

C -3.49230200 5.52867100 -0.52576700

H -2.36615100 5.42874600 -2.35692700

C -4.00629900 4.81777600 0.56506000

H -4.20493900 2.87908700 1.51041100

H -3.64719500 6.60782000 -0.60031600

H -4.55403200 5.34548400 1.35001900

C 2.30620900 2.52566200 -0.79082400

C 2.71066800 2.04009500 0.49885900

C 3.20743700 2.49593800 -1.84465900

C 1.84308600 1.96117300 1.62576000

C 4.06332300 1.57297100 0.65975800

C 4.52726200 2.01854400 -1.68515600

H 2.90580600 2.85299100 -2.83361300

C 2.27505500 1.44183500 2.82795000

H 0.80623800 2.27671300 1.54509200

C 4.48028300 1.06100600 1.92005500

C 4.94943300 1.57954200 -0.45095400

H 5.20066000 1.99367000 -2.54386800

C 3.60636300 0.98748600 2.98167300

H 1.57434000 1.36345700 3.66206400

H 5.50195400 0.68640200 2.01799000

H 5.96292000 1.19465000 -0.31688500

H 3.93042700 0.55983000 3.93270900

C 0.95039400 4.47985100 -1.67979500

H 1.58869300 5.13185100 -1.06713000

H 1.36918000 4.46071600 -2.69689100

H -0.04932400 4.93398200 -1.72032700

**TS _dissociation-2_**

**G_gas_ = -3769.965731 a.u.**

**E_gas_ = -3770.47331486 a.u.**

Si 1.65333600 -0.79211400 1.16747600

H -0.60162000 -0.10871800 0.10674900

C 0.44881000 -1.82538900 2.12162500

C 0.40489200 -1.69499000 3.52393600

C -0.50120200 -2.64168000 1.48122400

C -0.56259800 -2.37176200 4.26993100

H 1.09154000 -1.01751100 4.03920400

C -1.46893200 -3.31428600 2.22800000

H -0.51168100 -2.72411100 0.39559000

C -1.50144800 -3.17951600 3.62054700

H -0.60748300 -2.23579700 5.35268400

H -2.21408800 -3.92728400 1.71569900

H -2.27404900 -3.69036600 4.20093000

B -1.35396700 0.85674400 -0.01538400

C -0.34454100 2.12043100 -0.27669800

C 0.62495700 2.00135700 -1.27741400

C -0.31252500 3.32712800 0.42731400

C 1.57556800 2.97922600 -1.56794200

C 0.63689400 4.32843900 0.19110600

C 1.58251600 4.15477200 -0.81941200

C -2.25408900 0.55475300 -1.34085600

C -2.84571100 1.56509800 -2.10159600

C -2.46335300 -0.74232400 -1.80909000

C -3.57333800 1.32409400 -3.27031400

C -3.18551700 -1.03445600 -2.96750900

C -3.73728100 0.00920800 -3.71077300

C -2.18397100 0.85213000 1.39070400

C -3.55240000 0.60559400 1.53563900

C -1.47946300 0.91197200 2.59516700

C -4.16817000 0.39327200 2.77617600

C -2.03865800 0.69520700 3.85063800

C -3.40597800 0.43201700 3.94337300

N 3.32263500 -1.82926700 1.09939400

H 3.17013400 -2.60545700 1.75569400

C 3.52532800 -2.44544000 -0.29079200

H 3.57595900 -1.58294700 -0.96428500

C 4.83865700 -3.22416400 -0.40636900

H 4.90902400 -4.02245400 0.34865300

H 4.87473100 -3.69916200 -1.39729400

H 5.72574900 -2.58172400 -0.31014500

C 2.32796100 -3.31693000 -0.65228000

C 1.48210700 -3.01930000 -1.77622700

C 2.07300400 -4.44108000 0.11835700

C 1.65419100 -1.89209500 -2.63015900

C 0.37013600 -3.89694200 -2.04343400

C 0.98712700 -5.30150600 -0.15300900

H 2.71776600 -4.68036200 0.97007100

C 0.78011800 -1.63703000 -3.66529600

H 2.46145900 -1.18082700 -2.45607400

C -0.51245400 -3.60143900 -3.11821800

C 0.15219300 -5.02965600 -1.21419000

H 0.81082400 -6.16861400 0.48668700

C -0.31618600 -2.49544900 -3.91325200

H 0.91819900 -0.74131400 -4.27384000

H -1.37582300 -4.24913900 -3.28251300

H -0.69983300 -5.67946700 -1.42945600

H -1.02089700 -2.26777300 -4.71482100

H 2.11856300 0.36532400 1.95200900

H 1.50181300 -0.52729200 -0.26149500

F 0.65192400 5.44774600 0.91916100

F 2.51541600 5.09105300 -1.04088200

F 2.48664700 2.79048100 -2.53218200

F 0.68478900 0.87945900 -2.02707200

F -1.20871200 3.57860100 1.39046200

F -0.14675700 1.15255700 2.57018300

F -1.27939600 0.70334700 4.95379600

F -3.97156600 0.21155900 5.13196400

F -5.47844700 0.14785200 2.85174200

F -4.35953400 0.53327500 0.46529100

F -1.96604900 -1.79975200 -1.13593300

F -3.34194100 -2.29924700 -3.38638600

F -4.41922500 -0.24943800 -4.82835000

F -4.11159100 2.32782700 -3.96677100

F -2.73750300 2.84591500 -1.71252900

Si 4.71288600 -0.78349200 1.79395200

H 5.91120900 -1.65594400 1.81688700

H 4.25632300 -0.49166100 3.17294600

C 4.86726600 0.72653400 0.72736800

C 5.40904000 0.65740500 -0.57409400

C 4.38343900 1.96768700 1.18968500

C 5.44351400 1.78824200 -1.39195300

H 5.80975200 -0.28468700 -0.96004400

C 4.42526900 3.09888400 0.37259900

H 3.95376200 2.05387600 2.19129000

C 4.94809600 3.00809300 -0.92021000

H 5.84943000 1.71742600 -2.40367700

H 4.02902300 4.05077100 0.73026400

H 4.94239800 3.88707400 -1.56681100

**TS _association-2_**

**G_gas_ = -3769.987693 a.u.**

**E_gas_ = -3770.49646653 a.u.**

H -0.11140500 1.41561000 0.52698100

B 1.26181800 -0.91364100 -0.02093400

C 0.18378400 -1.97923100 -0.62539200

C 0.05168800 -2.30569100 -1.97746900

C -0.75910800 -2.59190400 0.20435100

C -0.91064800 -3.19215200 -2.47126700

C -1.73092900 -3.48724800 -0.23750400

C -1.82509700 -3.77624300 -1.59819600

C 1.94880300 -1.35893800 1.39661200

C 2.38593400 -0.37135500 2.27862100

C 2.17577000 -2.66927500 1.82656700

C 2.97533100 -0.62362500 3.51783600

C 2.76585200 -2.97994200 3.05560700

C 3.17121700 -1.94942400 3.90754300

C 2.44495900 -0.45413400 -1.04702800

C 2.45463900 0.79697800 -1.65709800

C 3.55511200 -1.25638300 -1.32298100

C 3.49944500 1.26709600 -2.45417200

C 4.62183200 -0.84184600 -2.12477100

C 4.59603500 0.43667600 -2.68902200

F -0.78865500 -2.29491200 1.52214600

F -2.61631500 -4.01153100 0.61795700

F -2.78546800 -4.58075200 -2.05100600

F -1.01324700 -3.41241300 -3.78578500

F 0.83028200 -1.72414700 -2.90726200

F 1.81026700 -3.70542000 1.06147600

F 2.95077500 -4.24760100 3.42267700

F 3.73397200 -2.22902300 5.08178100

F 3.34545500 0.37787600 4.32011600

F 2.23052600 0.93889000 1.95240700

F 1.40174900 1.63844400 -1.49991300

F 3.62448500 -2.49240700 -0.80946100

F 5.66094700 -1.64362500 -2.35434100

F 5.60702600 0.85825600 -3.44691400

F 3.46535800 2.49556900 -2.98356800

H 0.64217900 0.11776200 0.24903100

N -0.90987600 2.00843300 0.86376000

Si -1.54269600 2.78519200 -0.75489900

H -0.31701200 3.30369300 -1.38325700

H -2.43837600 3.88230600 -0.32551900

C -1.84000400 0.92199200 1.45519100

H -1.72024300 0.10507100 0.73433300

C -1.34204400 0.41956100 2.81008900

H -1.46899000 1.17712100 3.59837900

H -1.94087300 -0.45772600 3.09066500

H -0.29213800 0.10063800 2.75989300

C -3.30999400 1.31142600 1.48722900

C -4.30474900 0.33033300 1.13941500

C -3.71941300 2.58032800 1.85622600

C -3.99481100 -1.02220600 0.82327400

C -5.68435300 0.73222500 1.08444700

C -5.08269300 2.96310900 1.83414300

H -2.98368400 3.32580000 2.15415900

C -4.97148500 -1.91030800 0.42186900

H -2.97470000 -1.38439600 0.91696800

C -6.66844800 -0.20586500 0.66472200

C -6.04380400 2.06253900 1.43759400

H -5.35849600 3.98035800 2.12111700

C -6.32145300 -1.49638200 0.32654800

H -4.69156000 -2.93748500 0.18302800

H -7.71156200 0.11835600 0.61703800

H -7.09651800 2.35414800 1.39480200

H -7.08591800 -2.20593700 0.00093000

C -2.38846000 1.48899800 -1.77549100

C -1.62433500 0.59670900 -2.55546300

C -3.79408200 1.39451700 -1.81349000

C -2.25386200 -0.36691200 -3.34740900

H -0.53329200 0.64907300 -2.54605900

C -4.41983500 0.42084700 -2.59327800

H -4.40620800 2.07411600 -1.21575100

C -3.64907100 -0.46029700 -3.35925800

H -1.65306300 -1.05180500 -3.94731000

H -5.50946000 0.34346800 -2.59342500

H -4.13579600 -1.22768400 -3.96664100

Si -0.05166700 3.25542900 1.99581100

H -1.13676100 4.09402500 2.55598800

H 0.63711700 2.47113600 3.03395600

C 1.05593500 4.27215700 0.90547100

C 2.26212600 3.75564400 0.38996100

C 0.64526700 5.56165600 0.50887700

C 3.01754200 4.49651500 -0.52052300

H 2.60831800 2.76416200 0.68564300

C 1.40965100 6.30697900 -0.39142400

H -0.28492100 5.98710000 0.89762300

C 2.59048400 5.76901700 -0.91371800

H 3.93155200 4.07230100 -0.94005100

H 1.08063200 7.30478800 -0.69208300

H 3.18152500 6.34399600 -1.63107800

**3. Energies and coordinates for the conformations for each TS with a free energy difference less than 3.0 kcal/mol compared to the most stable conformation**

**TS3 (conformation 1)**

**G_gas_ = -3248.497059 a.u.**

**E_gas_ = -3248.90833589 a.u.**

C -0.82929600 -0.32945600 2.34202300

H -1.14870300 0.52449800 1.76295400

C -1.12595600 -1.64889100 1.83607400

C -1.88393300 -1.81730700 0.61437200

C -0.65035300 -2.77986400 2.50917900

C -2.36733900 -0.73362700 -0.16281200

C -2.11046000 -3.15025500 0.12982600

C -0.89616300 -4.07831500 2.03603500

H -0.05118300 -2.65951700 3.41203400

C -3.01481000 -0.95273200 -1.36291200

H -2.21047900 0.29164300 0.15933400

C -2.78606300 -3.34254900 -1.10699500

C -1.60727500 -4.25702700 0.86350800

H -0.49107900 -4.93774100 2.57248200

C -3.22418500 -2.26494100 -1.84540500

H -3.35205600 -0.09536100 -1.94749900

H -2.91782700 -4.36142200 -1.47911300

H -1.76487600 -5.26303000 0.46611300

H -3.71412300 -2.42056100 -2.80873000

C 0.17719100 -0.06407000 3.41581100

H 0.00640100 -0.67389100 4.31459700

H 1.17179300 -0.33402500 3.02535000

H 0.19839900 0.99926300 3.68101500

N -2.69090800 0.02089300 3.54592600

H -3.26384600 -0.71827800 3.13568800

Si -3.41586700 1.66132200 3.39268000

H -4.34656800 1.87934100 4.53859600

H -2.29559400 2.63450700 3.45603500

C -4.27566900 1.70416000 1.73634600

C -5.32041900 0.80302500 1.44542300

C -3.86250500 2.59814400 0.73084100

C -5.92271500 0.78651700 0.18612000

H -5.67398700 0.09862500 2.20638500

C -4.46178000 2.58079400 -0.53260900

H -3.04722000 3.29948500 0.91149100

C -5.49116600 1.67551300 -0.80517300

H -6.72668700 0.07725700 -0.02543600

H -4.10610100 3.27220900 -1.29938200

H -5.95811700 1.65906900 -1.79343900

B 1.17950400 0.20337800 -0.16305300

C 1.06161600 -1.29514000 -0.77393400

C 1.50488300 -2.37468900 -0.00045400

C 0.38778100 -1.66000200 -1.94467300

C 1.33072700 -3.71101500 -0.35002500

C 0.18811400 -2.98719200 -2.33747500

C 0.65795400 -4.02145100 -1.53230900

C 0.66176700 1.42918800 -1.10416600

C 1.25380100 1.68940200 -2.34364300

C -0.33399000 2.32471800 -0.72672000

C 0.86158400 2.73593500 -3.18009500

C -0.76656200 3.38521600 -1.52642400

C -0.16367300 3.59266400 -2.76613900

C 2.62452300 0.60649200 0.47392700

C 2.68242300 1.59394100 1.46221100

C 3.85769700 0.06630900 0.09815400

C 3.86690000 2.01892400 2.06690100

C 5.07027400 0.46149800 0.67180900

C 5.07433500 1.44564400 1.66281700

F -0.95788500 2.20197300 0.47142200

F -1.75362500 4.19834600 -1.11711000

F -0.56042700 4.59587300 -3.54773200

F 1.44562600 2.92990300 -4.36213600

F 2.23541300 0.88988000 -2.77940300

F 1.54551700 2.19146800 1.87891300

F 3.85741600 2.95954500 3.01504300

F 6.22075500 1.83298600 2.22040100

F 6.22170000 -0.08747300 0.28326200

F 3.92451100 -0.88087700 -0.84677000

F -0.16487100 -0.73385800 -2.74438400

F -0.51360400 -3.27236600 -3.43761200

F 0.40127900 -5.29334300 -1.84759100

F 1.72502100 -4.69152900 0.46810200

F 2.09128200 -2.14566000 1.19351100

H 0.41237100 0.14968700 0.81334100

H -2.42610000 -0.27460000 4.48672200

**TS3 (conformation 2)**

**G_gas_ = -3248.491936 a.u.**

**E_gas_ = -3248.90902867 a.u.**

C -1.67623000 -0.40408200 1.14258600

H -0.89155500 -0.26850500 1.87262700

C -2.06734600 0.73216500 0.33497900

C -1.54745800 2.05324100 0.60789900

C -2.93944000 0.54791200 -0.74076100

C -0.60871700 2.32915200 1.63635800

C -1.95531400 3.13683600 -0.24149600

C -3.34529700 1.61889800 -1.55444300

H -3.30814200 -0.44992500 -0.97285500

C -0.08432500 3.59699100 1.79964100

H -0.24272500 1.53061500 2.27751700

C -1.40386600 4.43236600 -0.04126100

C -2.85976400 2.88863600 -1.30864400

H -4.01047800 1.43540000 -2.39981500

C -0.48038100 4.66004300 0.95547400

H 0.66626200 3.77091200 2.57374200

H -1.69852300 5.23729000 -0.71893200

H -3.13898300 3.71968000 -1.96133800

H -0.03762100 5.65014400 1.08086800

C -1.97341000 -1.81605300 0.74688900

H -3.04635300 -1.98652800 0.58575500

H -1.45448200 -2.01636400 -0.20443800

H -1.58508300 -2.52453300 1.48803900

N -2.99576300 -0.20754900 2.87050100

H -2.90395300 -1.12309800 3.31531500

Si -4.69018000 0.31857500 2.56479300

H -5.38525600 0.45477800 3.87718700

H -4.60186500 1.63201500 1.88305600

C -5.48857700 -0.97543300 1.47812800

C -5.61016500 -2.30971100 1.91557900

C -5.94279600 -0.65248000 0.18513600

C -6.14797800 -3.29312300 1.08184900

H -5.28078500 -2.59597700 2.92026100

C -6.48686700 -1.63352000 -0.64952900

H -5.85931100 0.37384900 -0.18186600

C -6.58422000 -2.95527800 -0.20414500

H -6.22891900 -4.32415200 1.43483700

H -6.83202400 -1.36645100 -1.65150800

H -7.00357700 -3.72381100 -0.85822300

B 1.32829100 -0.29470200 -0.13548600

C 1.00579700 0.90377200 -1.18184900

C 0.05367300 0.69513300 -2.18637300

C 1.45319100 2.22470100 -1.07274900

C -0.41412200 1.69182600 -3.03882000

C 1.00868400 3.26027600 -1.90102800

C 0.06508700 2.99351300 -2.88957400

C 2.47639100 -0.00375700 0.98376700

C 3.80536000 0.19096200 0.59506900

C 2.25717400 -0.00090000 2.35805400

C 4.84929500 0.40275600 1.49602500

C 3.26502700 0.20630300 3.30451200

C 4.57400500 0.40687000 2.86706800

C 1.59575300 -1.76668700 -0.78227800

C 1.34645200 -2.90185800 -0.00534600

C 2.08876300 -2.01936000 -2.06526200

C 1.54214600 -4.20920800 -0.45417800

C 2.30473600 -3.30993600 -2.55872800

C 2.03046200 -4.41248100 -1.74644400

F 1.01162700 -0.19323100 2.87086600

F 2.98821900 0.20966900 4.61195100

F 5.55378900 0.60519300 3.74823200

F 6.09840700 0.59613500 1.07258200

F 4.10915700 0.19676700 -0.71039000

F 0.88694800 -2.76155600 1.25765700

F 1.27275900 -5.25564800 0.33180100

F 2.22941200 -5.64919800 -2.20188500

F 2.77089500 -3.50111700 -3.79365300

F 2.37100300 -1.00718900 -2.89567000

F 2.31134900 2.58717800 -0.10557200

F 1.42712400 4.51398800 -1.70707700

F -0.43435800 3.98477000 -3.63272000

F -1.37041800 1.43399500 -3.93705800

F -0.51472900 -0.52088000 -2.32849900

H 0.25434700 -0.41649000 0.46603900

H -2.45455700 0.45666700 3.42885000

**TS3 (conformation 3)**

**G_gas_ = -3248.497337 a.u.**

**E_gas_ = -3248.90902867 a.u.**

C -0.02920500 -1.71776200 -1.71859100

H 0.65241500 -1.87022000 -0.89201400

C -1.44665900 -1.94462100 -1.52325300

C -1.97208400 -2.34683000 -0.23710700

C -2.33224500 -1.73238500 -2.58523200

C -1.16200100 -2.54822800 0.91080300

C -3.39454500 -2.48521100 -0.09199800

C -3.71817000 -1.90717300 -2.44117100

H -1.94989200 -1.39894800 -3.55003700

C -1.72741800 -2.83382200 2.13876900

H -0.08312000 -2.43266500 0.85104800

C -3.94428100 -2.78560600 1.18493200

C -4.23903400 -2.26574900 -1.21244500

H -4.37990600 -1.71505000 -3.28717200

C -3.12937000 -2.94770000 2.28383600

H -1.08137300 -2.94764600 3.01209100

H -5.03044700 -2.84989700 1.28521600

H -5.32017100 -2.35456900 -1.07870400

H -3.56221000 -3.14605900 3.26643100

C 0.50751200 -0.98985800 -2.91169500

H 0.23111400 -1.48066800 -3.85552700

H 0.07586300 0.02317900 -2.92624700

H 1.59553400 -0.89451400 -2.85225000

N 0.54607100 -3.80701000 -2.26701000

H 0.19585800 -4.25504100 -1.41797000

Si 2.27946400 -4.12328500 -2.63620700

H 2.51415200 -5.59651400 -2.65231200

H 2.48537800 -3.54438900 -3.98959500

C 3.31735400 -3.27884700 -1.34099500

C 3.49704400 -3.86157400 -0.06873200

C 3.89525100 -2.01888000 -1.58580200

C 4.21450100 -3.19715000 0.92605500

H 3.07041500 -4.84573000 0.15251900

C 4.61512200 -1.35289100 -0.59074500

H 3.78399800 -1.53929900 -2.56207300

C 4.77179800 -1.94081200 0.66539000

H 4.32543900 -3.64647200 1.91522500

H 5.02634100 -0.36224400 -0.79556200

H 5.31094300 -1.41403900 1.45547300

B -0.11298400 0.89887900 0.13816700

C -1.72472100 0.91503000 0.30458600

C -2.53228400 1.06281700 -0.82884500

C -2.42107300 0.59732900 1.47629500

C -3.91863000 0.93518700 -0.82235500

C -3.81103100 0.45493500 1.53154300

C -4.56585400 0.62015300 0.37300600

C 0.76361200 0.79767600 1.50728900

C 0.73824300 1.84402400 2.43377900

C 1.63052600 -0.24757300 1.81306400

C 1.49783200 1.85531600 3.60420400

C 2.41149600 -0.28563600 2.97192300

C 2.34909100 0.77874100 3.87098700

C 0.54477800 2.06043400 -0.79664400

C 1.79620900 1.82975700 -1.37397800

C -0.00283800 3.32059100 -1.05402900

C 2.47164000 2.75304600 -2.17199700

C 0.63518400 4.28140600 -1.84575500

C 1.88220500 3.99651900 -2.40791500

F 1.75350700 -1.31021200 0.98269700

F 3.22463900 -1.31960500 3.22512400

F 3.08901200 0.76500000 4.97912500

F 1.43001700 2.87312800 4.46205600

F -0.06565500 2.89333000 2.21172600

F 2.41970500 0.64863600 -1.16871500

F 3.66398000 2.45898400 -2.70053200

F 2.50147800 4.90169800 -3.16390000

F 0.06978000 5.46824000 -2.06731100

F -1.19139700 3.66188500 -0.54184800

F -1.76922300 0.35347500 2.62414100

F -4.41312900 0.09209500 2.66698600

F -5.88351300 0.40659100 0.38917500

F -4.62164100 1.02830200 -1.95482600

F -1.96248100 1.28308200 -2.03290300

H 0.07284100 -0.15752600 -0.50114700

H -0.11668600 -4.02065100 -3.01428900

**TS3 (conformation 4)**

**G_gas_ = -3248.497059 a.u.**

**E_gas_ =** **-3248.90833590 a.u.**

C -0.82937500 0.32943900 -2.34212200

H -1.14863500 -0.52460500 -1.76309900

C -1.12619900 1.64879200 -1.83606500

C -1.88405600 1.81701800 -0.61426000

C -0.65084800 2.77988400 -2.50915400

C -2.36717100 0.73321800 0.16293300

C -2.11071400 3.14990000 -0.12959500

C -0.89678700 4.07826600 -2.03589000

H -0.05178800 2.65969000 -3.41210200

C -3.01451500 0.95214700 1.36313300

H -2.21017600 -0.29200200 -0.15928800

C -2.78618100 3.34201400 1.10733000

C -1.60778800 4.25679500 -0.86326600

H -0.49190000 4.93778700 -2.57233200

C -3.22403600 2.26429300 1.84573300

H -3.35152200 0.09468300 1.94772300

H -2.91804300 4.36084200 1.47953300

H -1.76549200 5.26274900 -0.46578700

H -3.71386000 2.41977700 2.80913800

C 0.17713800 0.06423600 -3.41592100

H 0.00651400 0.67434200 -4.31454400

H 1.17174100 0.33394900 -3.02529100

H 0.19822100 -0.99902400 -3.68143800

N -2.69091700 -0.02105700 -3.54616200

H -3.26387900 0.71822700 -3.13616200

Si -3.41599900 -1.66141100 -3.39274900

H -4.34689600 -1.87940800 -4.53851000

H -2.29580300 -2.63467700 -3.45624400

C -4.27558400 -1.70413500 -1.73630400

C -5.32033000 -0.80302000 -1.44530300

C -3.86230000 -2.59807700 -0.73081500

C -5.92249200 -0.78648500 -0.18593700

H -5.67400400 -0.09866500 -2.20625800

C -4.46144000 -2.58069900 0.53269800

H -3.04702500 -3.29941200 -0.91152800

C -5.49081600 -1.67543500 0.80534300

H -6.72646000 -0.07723800 0.02567700

H -4.10566700 -3.27208500 1.29945400

H -5.95766100 -1.65896400 1.79365800

B 1.17949200 -0.20333100 0.16302200

C 1.06154900 1.29519100 0.77388900

C 1.50465900 2.37474600 0.00033000

C 0.38781500 1.66004500 1.94468900

C 1.33047300 3.71107100 0.34988800

C 0.18813000 2.98723300 2.33748700

C 0.65783200 4.02150000 1.53224900

C 0.66184500 -1.42917000 1.10414900

C 1.25386700 -1.68932800 2.34364400

C -0.33381600 -2.32479900 0.72667700

C 0.86170700 -2.73588300 3.18009600

C -0.76633200 -3.38531800 1.52638200

C -0.16347400 -3.59269500 2.76612500

C 2.62452600 -0.60635300 -0.47398200

C 2.68247100 -1.59375300 -1.46231100

C 3.85767400 -0.06613800 -0.09817200

C 3.86697100 -2.01866600 -2.06700400

C 5.07027300 -0.46125900 -0.67182800

C 5.07438100 -1.44536200 -1.66287800

F -0.95767800 -2.20213800 -0.47149000

F -1.75331900 -4.19852800 1.11704300

F -0.56018300 -4.59591500 3.54772600

F 1.44573000 -2.92979200 4.36215700

F 2.23541500 -0.88973600 2.77941800

F 1.54559000 -2.19129800 -1.87905500

F 3.85753500 -2.95924200 -3.01519200

F 6.22082100 -1.83263800 -2.22046700

F 6.22167500 0.08773800 -0.28324500

F 3.92443800 0.88101500 0.84679000

F -0.16472600 0.73389200 2.74446700

F -0.51348500 3.27239600 3.43769400

F 0.40114500 5.29338600 1.84754400

F 1.72461900 4.69158700 -0.46830900

F 2.09093100 2.14571900 -1.19369900

H 0.41233400 -0.14965900 -0.81334100

H -2.42595900 0.27423000 -4.48698200

**TS3 (conformation 5)**

**G_gas_ = -3248.491198 a.u.**

**E_gas_ =** **-3248.90267832 a.u.**

C 0.93488500 -0.09345600 -2.13829900

H 0.62628400 0.92756200 -1.95353900

C 1.83824700 -0.72588400 -1.19640300

C 2.29856000 -0.02938000 -0.01686500

C 2.24853700 -2.04459400 -1.41423800

C 1.89428200 1.28720100 0.32558700

C 3.16531300 -0.72439600 0.89203900

C 3.11728700 -2.70514900 -0.53162100

H 1.87745600 -2.59048300 -2.28211000

C 2.30134900 1.87328800 1.50776800

H 1.22207800 1.84129700 -0.32283000

C 3.57407400 -0.08980300 2.09704700

C 3.56440400 -2.05478400 0.60244200

H 3.40667000 -3.73899400 -0.72658400

C 3.14481200 1.18155900 2.40712500

H 1.94331200 2.87380000 1.75969300

H 4.20872000 -0.64600100 2.79095200

H 4.21223900 -2.57303100 1.31370900

H 3.43625100 1.64728900 3.35071300

C 0.10606300 -0.87861200 -3.11086100

H 0.71603900 -1.51930600 -3.76414500

H -0.55795100 -1.54345700 -2.53688400

H -0.52383100 -0.21767400 -3.71735800

N 2.32739900 0.71356700 -3.61434200

H 2.79406000 -0.14816000 -3.90236400

Si 3.46469400 2.00965500 -3.08609300

H 4.08031900 2.60977700 -4.30603400

H 2.64508800 3.03144600 -2.38804600

C 4.72495400 1.23159900 -1.94839400

C 5.32206000 -0.00753900 -2.24772600

C 5.09214000 1.87882800 -0.75358300

C 6.24833200 -0.58713400 -1.37747400

H 5.06663700 -0.54351000 -3.16778100

C 6.02475600 1.30581600 0.11412300

H 4.62788100 2.82964100 -0.47963400

C 6.60229900 0.07144900 -0.19605500

H 6.69272500 -1.55528100 -1.62066900

H 6.28734200 1.81693000 1.04289100

H 7.32389100 -0.38264100 0.48772800

B -1.43387700 -0.05151400 0.08060700

C -0.62258200 -0.93362300 1.17542200

C -0.21526800 -2.22879100 0.83529300

C -0.10782200 -0.46494500 2.38854900

C 0.61943100 -3.01890400 1.62086400

C 0.73534400 -1.21946700 3.21070400

C 1.10619300 -2.50428400 2.82281900

C -1.89784500 1.44544300 0.52323100

C -2.81127400 1.62209900 1.56684600

C -1.51130900 2.61305100 -0.12954800

C -3.29045000 2.86915000 1.96869800

C -1.95983400 3.88700400 0.23223500

C -2.86051700 4.01352500 1.28967600

C -2.69624300 -0.78984400 -0.63998800

C -3.10763000 -0.35564000 -1.90359600

C -3.45553900 -1.82761500 -0.09150300

C -4.17783700 -0.91103900 -2.60700000

C -4.53928700 -2.41255800 -0.75421300

C -4.90328900 -1.95014100 -2.02084100

F -0.64356800 2.57720400 -1.17416700

F -1.54212100 4.97338500 -0.42381500

F -3.30484500 5.21614100 1.65302800

F -4.15083900 2.98520100 2.98034500

F -3.24804700 0.54863400 2.24025800

F -2.44935700 0.65572000 -2.51087100

F -4.51211400 -0.46212500 -3.81994800

F -5.93185300 -2.49825700 -2.66662800

F -5.23020700 -3.40459600 -0.19141800

F -3.16096100 -2.31903100 1.11894700

F -0.35391700 0.78619100 2.80808800

F 1.24529900 -0.69284900 4.32733200

F 1.97647000 -3.20629900 3.55309800

F 1.01874800 -4.22387700 1.20214100

F -0.58629900 -2.75772800 -0.35120200

H -0.59120800 0.08552100 -0.82139300

H 1.70081000 0.98872000 -4.37390900

**TS3 (conformation 6)**

**G_gas_ = -3248.510976 a.u.**

**E_gas_ = -3248.92330581 a.u.**

Si 0.56536900 -0.21245800 1.76511900

H -0.74262100 -0.09028000 0.99948800

C 1.67451900 -1.28741300 0.70039600

C 1.80548500 -2.65852600 0.99770400

C 2.34945700 -0.78361200 -0.42665500

C 2.58441200 -3.49865600 0.19711500

H 1.27991100 -3.08384600 1.85800500

C 3.12235600 -1.62395300 -1.23123500

H 2.28398500 0.27405600 -0.68085700

C 3.23954000 -2.98301100 -0.92506200

H 2.66290500 -4.56161700 0.43761500

H 3.64041800 -1.21055900 -2.09935400

H 3.83703000 -3.63963500 -1.56215200

B -2.03694200 0.04793600 -0.44320200

C -3.19235000 -0.55252300 0.46019700

C -3.45965400 -0.04797300 1.74020800

C -4.02455900 -1.59211600 0.02596100

C -4.46883500 -0.55073800 2.56033100

C -5.05435700 -2.11469600 0.81020200

C -5.27540600 -1.58918800 2.08566100

C -1.95674100 1.62751800 -0.58372100

C -3.10166400 2.43686100 -0.52841700

C -0.74438500 2.31084100 -0.75692200

C -3.05527900 3.82976900 -0.63045700

C -0.65418200 3.69733500 -0.85088400

C -1.82143600 4.46357900 -0.78626800

C -1.20843300 -0.89874500 -1.39630700

C -0.68943500 -0.49312000 -2.63684700

C -0.89130400 -2.21450100 -1.01560100

C 0.10697000 -1.31654100 -3.43368700

C -0.11873300 -3.07333000 -1.79198900

C 0.40102400 -2.61107800 -3.00154100

N 3.22702800 -0.42448600 3.34042500

H 3.27710000 -1.37343000 2.96565300

H 2.87638800 -0.50424400 4.29653800

C 4.57658500 0.17114800 3.34355400

H 4.46047400 1.21133600 3.69343100

C 5.52427300 -0.52009600 4.33350500

H 5.62246800 -1.59597100 4.11643100

H 6.53012100 -0.07167600 4.31946800

H 5.12513100 -0.42786500 5.35653600

C 5.09871500 0.23193300 1.90957800

C 4.56292900 1.19954200 0.98946000

C 6.05662600 -0.65724500 1.45663800

C 3.55547900 2.13768400 1.35312500

C 5.03456700 1.21523500 -0.36818700

C 6.52774800 -0.63424400 0.12018000

H 6.47300000 -1.40381900 2.13416000

C 3.02043900 3.01208100 0.43066000

H 3.16962700 2.13997100 2.37115300

C 4.46527500 2.13362400 -1.29502600

C 6.03107100 0.28513700 -0.77323900

H 7.28656900 -1.35566600 -0.19283300

C 3.46980300 3.00668000 -0.91175700

H 2.22846700 3.70252300 0.72552800

H 4.82470000 2.12341900 -2.32788800

H 6.38420600 0.30579600 -1.80791700

H 3.01557400 3.68792800 -1.63404700

H 0.21006900 -0.91320200 3.02869800

H 0.98402100 1.18698700 1.98049200

F -5.82587500 -3.09805300 0.35565300

F -6.24795400 -2.07517400 2.84567500

F -4.67595200 -0.05133200 3.77574500

F -2.71681700 0.95193400 2.23272500

F -3.86187300 -2.12269000 -1.19082800

F -1.29859700 -2.68678300 0.16813200

F 0.15588700 -4.30652200 -1.37895500

F 1.17654400 -3.39785000 -3.73428800

F 0.59406900 -0.87820900 -4.59198800

F -0.95211800 0.72540400 -3.12030800

F 0.40298000 1.62432200 -0.82752200

F 0.52455700 4.30390900 -0.99937500

F -1.75559800 5.78414600 -0.87968100

F -4.17018200 4.55190200 -0.58143200

F -4.31488300 1.89276900 -0.39364400

**TS4 (conformation 1)**

**G_gas_ = -3248.512391 a.u.**

**E_gas_ =** **-3248.91940730 a.u.**

H 0.18065300 0.06121400 -1.10217700

B -0.97486600 0.76217200 0.99543600

C -1.49870400 2.10399900 0.31750200

C -2.85574800 2.45083300 0.26492600

C -0.62553200 3.00365200 -0.30555700

C -3.32291000 3.59667400 -0.38447500

C -1.04854600 4.14419800 -0.98425600

C -2.41312400 4.44413600 -1.02031500

C 0.30042100 0.81381900 1.94259300

C 1.13190500 -0.31010700 2.07513200

C 0.76207700 1.98670100 2.55628800

C 2.37393200 -0.26790900 2.70371200

C 1.99571200 2.06707300 3.20724000

C 2.81204500 0.93593900 3.26083500

C -1.93785700 -0.49689600 0.97869100

C -2.63630900 -0.84803000 -0.18399800

C -2.13437400 -1.33401500 2.08281000

C -3.48648700 -1.94495600 -0.25899500

C -2.96347900 -2.45795400 2.04234100

C -3.63735000 -2.76576900 0.85984300

F 0.69573200 2.77473000 -0.28418200

F -0.17315300 4.93876200 -1.59468500

F -2.84065700 5.52945100 -1.65177900

F -4.62118500 3.88396200 -0.40466200

F -3.77774800 1.68233700 0.85430700

F 0.03072700 3.10479200 2.52395700

F 2.40371700 3.20730600 3.75656400

F 4.01379400 1.00347400 3.82163500

F 3.16712000 -1.33732100 2.74184100

F 0.76547500 -1.46737700 1.52580700

F -2.48033900 -0.12729900 -1.30812500

F -1.52109000 -1.07604000 3.24236000

F -3.10605400 -3.23886400 3.11021700

F -4.39505900 -3.85376800 0.78350400

F -4.10334100 -2.25539100 -1.39902400

H 0.04036700 0.12724100 -0.25898700

N 0.39523600 0.00202800 -2.85389200

H -0.59248700 0.24389600 -2.94568400

Si 1.55992300 1.30035500 -3.20101600

H 0.88454900 2.56334900 -2.81579500

H 1.91527900 1.33698800 -4.65252200

C 0.65346100 -1.37411100 -3.35203200

H 1.74726800 -1.47443500 -3.41550900

C 3.14348200 0.93206600 -2.25874900

C 3.21254100 0.94796900 -0.84932600

C 4.29064500 0.53454900 -2.97288600

C 4.38297800 0.57706400 -0.18363900

H 2.34256200 1.23625900 -0.25962700

C 5.46301300 0.15805600 -2.30844200

H 4.26997700 0.50976800 -4.06667900

C 5.50942900 0.17845300 -0.91189900

H 4.41446900 0.58040800 0.90828000

H 6.33963300 -0.15336800 -2.88221600

H 6.42216600 -0.11804800 -0.38864700

C 0.15731800 -2.43454700 -2.36756800

C 1.00433900 -2.89861900 -1.29983400

C -1.11682900 -2.96169700 -2.48950800

C 2.32474600 -2.41234500 -1.07613800

C 0.50446200 -3.90415000 -0.39946500

C -1.60345900 -3.95301800 -1.60307800

H -1.78248200 -2.60958900 -3.27882000

C 3.09881500 -2.88172700 -0.03553400

H 2.73993000 -1.64042100 -1.71965100

C 1.33206500 -4.37198300 0.65930500

C -0.80975300 -4.41481300 -0.57932900

H -2.61986100 -4.33075900 -1.72921700

C 2.60241800 -3.87342300 0.84261600

H 4.09765600 -2.46795800 0.11685400

H 0.93256900 -5.13342400 1.33476000

H -1.18293800 -5.17373800 0.11378600

H 3.22172500 -4.22730400 1.66990900

C 0.11081600 -1.55237100 -4.77647700

H 0.29225400 -2.57388300 -5.14335600

H -0.97327000 -1.36256000 -4.82323700

H 0.60036800 -0.84289100 -5.46081200

**TS5 (conformation 1)**

**G_gas_ = -3769.979994 a.u.**

**E_gas_ =** **-3770.48232102 a.u.**

Si -0.56929900 0.42190200 -1.25468500

H 0.80841200 -0.10989700 -0.61767800

C -0.10160800 2.16262800 -1.72270600

C -0.24036800 3.24181900 -0.82962200

C 0.45132800 2.40845400 -2.99580900

C 0.16602400 4.52735500 -1.19394600

H -0.66399700 3.08412600 0.16077800

C 0.84840300 3.69676700 -3.36388400

H 0.59122300 1.58523000 -3.70102400

C 0.70948000 4.75735300 -2.46192500

H 0.06600600 5.34631400 -0.47698900

H 1.27764700 3.87132600 -4.35367700

H 1.03030600 5.76277400 -2.74668400

B 1.80088900 -0.48703000 0.29447200

C 0.97854900 -1.36160200 1.37314600

C 0.03979000 -2.31190300 0.96292500

C 1.15331100 -1.24672500 2.75574300

C -0.70770300 -3.08548300 1.84865300

C 0.42185800 -1.99718100 3.67878500

C -0.51193500 -2.92443000 3.21880100

C 2.74975200 -1.34604900 -0.68283200

C 3.48549200 -2.43944600 -0.21682300

C 2.91385600 -1.03922300 -2.03535200

C 4.31940600 -3.20064500 -1.04086200

C 3.72995500 -1.77192200 -2.89659400

C 4.43914000 -2.86442700 -2.39088600

C 2.31782700 0.96714200 0.75483100

C 3.58497200 1.48998200 0.47302700

C 1.45001500 1.84080100 1.41883900

C 3.95900500 2.79657400 0.80933200

C 1.77158700 3.15081600 1.75683900

C 3.04381500 3.63514200 1.44735300

H -1.41667300 0.14974600 -0.09577700

H -0.61339900 -0.49588500 -2.40681800

F 0.58935800 -1.82286600 4.98786900

F -1.26121600 -3.60593000 4.08341100

F -1.63141000 -3.94667100 1.41177400

F -0.19679600 -2.48903900 -0.35410800

F 2.04749300 -0.38742300 3.25804100

F 0.19572000 1.43864000 1.70067000

F 0.86255000 3.95298400 2.31586400

F 3.37417100 4.88708100 1.74592700

F 5.17642100 3.24829800 0.51714900

F 4.51333700 0.75095100 -0.14453700

F 2.27062600 0.02023700 -2.56276900

F 3.84288400 -1.44021000 -4.18166700

F 5.22678900 -3.57651200 -3.19009000

F 5.00244800 -4.23313200 -0.55181000

F 3.42909100 -2.78902400 1.07571400

N -3.10913100 1.14499700 -2.21785000

H -2.63697500 1.97779000 -2.57533800

Si -3.48833700 -0.06969400 -3.46344600

H -2.26221900 -0.21848000 -4.29054600

H -4.62938200 0.34704000 -4.33510100

C -4.21435600 1.54677400 -1.29666600

H -4.81790200 0.64293200 -1.13418600

C -3.99197000 -1.63320200 -2.56040700

C -3.04166900 -2.49145900 -1.97324500

C -5.35679400 -1.90171900 -2.33574900

C -3.43742600 -3.56809400 -1.17639200

H -1.97388000 -2.32006200 -2.11861000

C -5.75803300 -2.97611100 -1.53528100

H -6.12082600 -1.25869500 -2.78390600

C -4.79716000 -3.80747300 -0.95106700

H -2.68072900 -4.20405900 -0.71574900

H -6.82148800 -3.16297400 -1.36534100

H -5.10752800 -4.64386800 -0.31937700

C -3.68321700 1.98671100 0.06643600

C -3.46440900 1.02789900 1.11810500

C -3.37574400 3.31578700 0.29892500

C -3.75028300 -0.36386600 0.98491600

C -2.89083900 1.47619600 2.35939100

C -2.82146100 3.75461400 1.52593800

H -3.53702200 4.05618600 -0.48614600

C -3.49036000 -1.25197400 2.00819300

H -4.15725200 -0.75420700 0.05368700

C -2.63579000 0.53340200 3.39458200

C -2.57218700 2.85108900 2.53219200

H -2.57510200 4.81084800 1.65834600

C -2.93188800 -0.80163200 3.22968200

H -3.69515700 -2.31483500 1.86335100

H -2.19106900 0.88981600 4.32749300

H -2.11522500 3.17431100 3.47061100

H -2.72825900 -1.51690900 4.02917500

C -5.14128900 2.58168100 -1.95117400

H -5.93759400 2.89087200 -1.25729100

H -4.58975500 3.48447500 -2.25883200

H -5.61144600 2.16235200 -2.85335700

**TS6 (conformation 1)**

**G_gas_ =** **-3769.953321 a.u.**

**E_gas_ =** **-3770.45395394 a.u.**

Si 2.69936100 -3.96092100 0.15622700

H 3.11192800 -4.12111600 -1.26218500

C 0.84505800 -4.07950800 0.31962200

C 0.04620300 -4.38845300 -0.80111600

C 0.20245600 -3.82922200 1.54842400

C -1.34597400 -4.42963100 -0.69674500

H 0.51355700 -4.58243500 -1.77139800

C -1.18967500 -3.85995100 1.65197200

H 0.78643100 -3.58337200 2.44124900

C -1.96469200 -4.15933100 0.52827500

H -1.95840100 -4.64635300 -1.57471700

H -1.66667500 -3.61322500 2.60232500

H -3.05360200 -4.14336400 0.59093400

C 1.81827000 -0.68235400 0.70502400

H 1.05986300 -1.42058800 0.45661800

C 2.20290000 0.23503800 -0.33539600

C 1.96721200 -0.07693200 -1.72817200

C 2.73737200 1.48369700 0.00516200

C 1.42985900 -1.30927200 -2.16944200

C 2.29087000 0.91002200 -2.71710500

C 3.00263900 2.46009200 -0.96727700

H 2.90113400 1.73942600 1.04939400

C 1.23916400 -1.56393200 -3.51375500

H 1.11645000 -2.05958500 -1.44850400

C 2.07864000 0.62162100 -4.09306800

C 2.78987300 2.17347200 -2.30305000

H 3.35192100 3.44587900 -0.65578100

C 1.56643400 -0.59474200 -4.48805200

H 0.79733000 -2.51333200 -3.82420700

H 2.31681700 1.39011900 -4.83271800

H 2.99460500 2.93041200 -3.06504800

H 1.38612700 -0.80390900 -5.54406300

C 1.93832100 -0.36168000 2.15472200

H 2.92723700 0.03346500 2.42394500

H 1.18427200 0.40905400 2.37296900

H 1.68735800 -1.22908500 2.77732000

N 3.30646200 -2.32880700 0.67000700

H 3.38561300 -2.31305300 1.68966600

H 3.40979500 -4.94289700 1.02513700

Si 4.86893600 -1.74551600 -0.02319700

H 5.77863100 -2.92517600 -0.11097800

H 4.60476200 -1.20857800 -1.37774300

C 5.54213000 -0.42912500 1.12384100

C 5.73246900 -0.69504800 2.49467200

C 5.83824500 0.86191500 0.64842400

C 6.18382500 0.30016400 3.36536900

H 5.52905400 -1.69265000 2.89981900

C 6.29596200 1.85875900 1.51529500

H 5.69233600 1.10158800 -0.40780000

C 6.46340700 1.58077000 2.87522300

H 6.32012900 0.07744000 4.42655100

H 6.51488600 2.85744300 1.12916200

H 6.81411100 2.36147300 3.55481000

B -1.52489800 0.66537400 0.21347600

C -2.14473500 -0.25594600 -0.99536100

C -1.66312300 -0.15512400 -2.30730600

C -3.04379600 -1.31006600 -0.79953700

C -2.02281300 -1.01569400 -3.34426500

C -3.42663300 -2.20239100 -1.80546800

C -2.89863300 -2.07018900 -3.08583600

C -2.34953200 0.55437800 1.61614400

C -3.52376900 1.27042500 1.85841500

C -1.95998000 -0.30507300 2.64099600

C -4.27095000 1.14742200 3.03363400

C -2.67818600 -0.47927200 3.82397400

C -3.84433500 0.26042600 4.02526400

C -1.22528600 2.22774800 -0.16696000

C -0.19905900 2.90487500 0.49181600

C -1.87112000 2.97223000 -1.15843100

C 0.24697400 4.18057300 0.14650500

C -1.47886200 4.26207300 -1.52880500

C -0.40306100 4.86952900 -0.87666600

F -0.85006700 -1.07039200 2.51134800

F -2.26815800 -1.35667000 4.74963900

F -4.54697900 0.11751500 5.14934600

F -5.38838200 1.85313100 3.21561300

F -4.00159400 2.10338300 0.92167600

F 0.45791500 2.31196500 1.51739200

F 1.30103500 4.73045100 0.76764300

F 0.00158300 6.09070900 -1.23148500

F -2.11474600 4.91916700 -2.50098100

F -2.90110900 2.44691900 -1.83370700

F -3.57266400 -1.56233100 0.41196100

F -4.23072300 -3.23754500 -1.52604400

F -3.20633100 -2.95102600 -4.04070600

F -1.50055900 -0.87517200 -4.56902600

F -0.76693500 0.79695200 -2.62526000

H -0.41754900 0.17011000 0.41295000

**TS6 (conformation 2)**

**G_gas_ = -3769.950637 a.u.**

**E_gas_ =** **-3770.44851876 a.u.**

Si -2.88585900 2.93403900 -1.56981900

H -1.73161400 2.37717300 -2.31660200

C -2.40572000 4.46506300 -0.62166400

C -1.08241900 4.94137000 -0.66415500

C -3.33785000 5.13135100 0.20144600

C -0.69539700 6.04351400 0.10408300

H -0.33449700 4.44435700 -1.28045300

C -2.95251400 6.23405600 0.96682600

H -4.37952200 4.79422500 0.24665600

C -1.62843900 6.68860600 0.92037900

H 0.34246700 6.38199400 0.06448400

H -3.68439000 6.74263700 1.59962700

H -1.32613400 7.54837400 1.52387400

C -1.64530300 0.65911700 0.41568300

H -1.10382100 1.00256000 -0.45957800

C -1.99820200 -0.73647800 0.44970100

C -2.09172800 -1.50420100 -0.77301400

C -2.23254800 -1.36852600 1.67603700

C -1.81032400 -0.97348200 -2.05843000

C -2.48973100 -2.88006100 -0.68561500

C -2.54115400 -2.73285100 1.75122900

H -2.15253500 -0.79920500 2.60144800

C -1.94446300 -1.74834900 -3.19399200

H -1.46009400 0.05196000 -2.16360300

C -2.64527600 -3.64504700 -1.87539400

C -2.68068800 -3.46938400 0.59003400

H -2.68662900 -3.20239900 2.72519900

C -2.38280900 -3.09140800 -3.10853800

H -1.69076800 -1.32439600 -4.16822700

H -2.93923200 -4.69335300 -1.78944700

H -2.93973100 -4.52992800 0.64009800

H -2.47119500 -3.69311700 -4.01503800

C -1.45270600 1.49304600 1.63703600

H -2.26739800 1.37529500 2.36590900

H -0.51358200 1.17320200 2.11502000

H -1.32626500 2.55135700 1.37537400

N -3.42032600 1.70208600 -0.34358000

H -3.56678000 2.20437900 0.53629100

H -4.03484300 3.13313100 -2.49830000

Si -4.89051900 0.71094100 -0.68864000

H -5.99217700 1.68194800 -0.95742600

H -4.62551100 -0.09517500 -1.90238600

C -5.29791800 -0.36597000 0.78405700

C -5.28417600 0.14979700 2.09523300

C -5.65590100 -1.71319900 0.60125400

C -5.60679000 -0.65650200 3.18860900

H -5.01923100 1.19683700 2.27861100

C -5.99109400 -2.52033000 1.69289100

H -5.65652300 -2.14803700 -0.40218900

C -5.96331800 -1.99510900 2.98694200

H -5.58574400 -0.24102400 4.19928500

H -6.26233600 -3.56667500 1.53207600

H -6.21842100 -2.62735000 3.84113200

B 1.78228900 -0.27108800 0.07465100

C 1.53619600 -1.44329800 -1.03207700

C 0.91015300 -2.63189800 -0.63947900

C 1.79910400 -1.35080900 -2.40097600

C 0.59578500 -3.67220100 -1.50951900

C 1.47820100 -2.35827800 -3.31878200

C 0.86779200 -3.52708900 -2.86944700

C 2.57782700 1.04855300 -0.46998600

C 3.95434400 1.02546100 -0.71467900

C 1.96527100 2.28051000 -0.67562000

C 4.68516500 2.14284100 -1.12360700

C 2.65026700 3.42968600 -1.07753300

C 4.02377000 3.36075000 -1.30583100

C 2.50735700 -0.77922300 1.45367600

C 2.32763000 -0.05109300 2.63120700

C 3.34488800 -1.89175800 1.57504900

C 2.89654200 -0.39271500 3.85937800

C 3.93918800 -2.27758100 2.78088400

C 3.71587200 -1.52041100 3.93209300

F 0.62517300 2.42295100 -0.49940600

F 2.00355800 4.59412400 -1.26098300

F 4.69649900 4.44365900 -1.69447800

F 5.99732700 2.06531800 -1.34638100

F 4.63026100 -0.12258000 -0.57247300

F 1.55973200 1.06497300 2.62374100

F 2.67274900 0.34389400 4.95240200

F 4.27547400 -1.87235500 5.09044900

F 4.72297100 -3.35601300 2.84265500

F 3.61368400 -2.66197000 0.51152800

F 2.36452100 -0.24849000 -2.92375600

F 1.71804200 -2.19591300 -4.62349800

F 0.50173000 -4.47801900 -3.73274000

F -0.02838900 -4.77211600 -1.06857400

F 0.54886000 -2.80419000 0.64626900

H 0.65362500 0.08407500 0.40325700

**TS7 (conformation 1)**

**G_gas_ = -3769.966066 a.u.**

**E_gas_ =** **-3770.46857302 a.u.**

H 0.27429000 0.60026100 0.34879600

B 1.61723900 -1.50581000 -0.14622700

C 2.77691800 -0.69797300 -0.87670100

C 4.11768100 -0.75215000 -0.47746200

C 2.48438800 0.20226100 -1.90917800

C 5.10568400 0.06557000 -1.03164600

C 3.43398800 1.04513600 -2.48020900

C 4.75468600 0.97335600 -2.03201700

C 0.53419600 -2.27326800 -1.02306300

C -0.76417800 -2.49605300 -0.54511600

C 0.77320200 -2.69477500 -2.33819600

C -1.77845000 -3.05954700 -1.31235500

C -0.21569700 -3.26824000 -3.14264100

C -1.50014800 -3.44141400 -2.62607700

C 1.78141200 -1.86487200 1.39359200

C 2.25298600 -0.90937000 2.30328800

C 1.41503500 -3.09678900 1.95071900

C 2.31584100 -1.11964000 3.67844000

C 1.48005500 -3.35862400 3.32181400

C 1.92849600 -2.36128300 4.19096300

F 1.22568400 0.31722800 -2.35705700

F 3.09436800 1.93655700 -3.40555800

F 5.66781300 1.80182100 -2.53033500

F 6.36490200 -0.00433600 -0.61059000

F 4.50433000 -1.59748700 0.48394800

F 1.98297600 -2.55692600 -2.88776600

F 0.05151400 -3.63617600 -4.39143300

F -2.46823500 -3.92999800 -3.39318600

F -3.01234500 -3.19330900 -0.82852700

F -1.09285400 -2.11092500 0.69860900

F 2.63827100 0.29777500 1.86248000

F 0.97773000 -4.09128800 1.17138200

F 1.12145400 -4.54456900 3.80448800

F 1.98906600 -2.59143600 5.49560400

F 2.73283600 -0.15780000 4.49700700

H 0.64306300 -0.15399700 0.07919000

N -0.75000100 1.86087700 0.83196200

Si -1.08470200 2.56757000 -0.81044100

H 0.19236200 2.54395800 -1.55970300

H -1.55794100 3.97048500 -0.65180600

C -1.80531000 0.93742600 1.36979100

H -1.86288900 0.12518300 0.63643900

C -1.40235500 0.28938800 2.69966700

H -1.34275200 1.02856800 3.51384200

H -2.16477500 -0.45050500 2.98287400

H -0.43822900 -0.23199700 2.61411700

C -3.20152600 1.54916400 1.46228700

C -4.35416700 0.73586900 1.17719300

C -3.38333200 2.87500100 1.81024900

C -4.26999400 -0.63534200 0.79874700

C -5.65959300 1.33613200 1.23272500

C -4.67202800 3.45967500 1.87726000

H -2.51750600 3.50065800 2.02511500

C -5.39711400 -1.36453500 0.48090000

H -3.30460900 -1.13322500 0.74164800

C -6.80289400 0.55367100 0.90883700

C -5.78743800 2.70779100 1.58985500

H -4.76931900 4.51274600 2.15228800

C -6.67902100 -0.76784400 0.53636200

H -5.28955900 -2.40730500 0.17494600

H -7.78832300 1.02562800 0.95536900

H -6.78557600 3.15235400 1.62902000

H -7.56548100 -1.35486200 0.28326700

C -2.41600500 1.57499800 -1.68201700

C -2.15701500 0.32686100 -2.28355600

C -3.73337700 2.07142500 -1.71948700

C -3.18681100 -0.40161200 -2.88804600

H -1.14363700 -0.08250500 -2.28266900

C -4.76226000 1.34509500 -2.32199300

H -3.96802000 3.02673800 -1.24238500

C -4.48990900 0.10419800 -2.90287300

H -2.98298100 -1.37209700 -3.34382100

H -5.78103000 1.73944900 -2.31973200

H -5.29451700 -0.47425900 -3.36362200

Si 0.32788300 2.81365300 1.91078300

H -0.45838700 3.79877100 2.70842600

H 1.03275100 1.89766800 2.83589100

C 1.51202500 3.77577100 0.81923600

C 2.63437100 3.17884700 0.20846700

C 1.25963600 5.13757800 0.56205500

C 3.46558500 3.91820600 -0.63695100

H 2.86034000 2.12545800 0.38830900

C 2.08939500 5.87862100 -0.28432800

H 0.39843500 5.62896500 1.02443600

C 3.19181700 5.26731400 -0.88681100

H 4.32731600 3.44707400 -1.11300600

H 1.87399100 6.93320300 -0.47438200

H 3.84202000 5.84107200 -1.55215300

**TS1b (conformation 2)**

**G_gas_ = -849.192941 a.u.**

**E_gas_ =** **-849.411197172 a.u.**

Si -2.64070800 -1.86840800 0.48100900

H -3.95433700 -1.95351200 1.19427700

C -2.30427100 -0.06562100 0.05196600

C -2.39788600 0.93166400 1.04101600

C -1.89931000 0.31902800 -1.23909400

C -2.08340700 2.26429200 0.75569600

H -2.71316500 0.66951300 2.05622000

C -1.59063900 1.65011900 -1.53152300

H -1.80144700 -0.43625500 -2.02264400

C -1.67725900 2.62533300 -0.53292200

H -2.15509200 3.02192500 1.54069100

H -1.27098200 1.92711000 -2.53957600

H -1.42766500 3.66550200 -0.75854400

N 0.29262800 -2.25801100 -0.82849900

H 0.57144800 -1.93982100 -1.75742200

H -0.07342700 -3.20401200 -0.93674800

C 1.44581700 -2.25568700 0.07489900

C 2.09788400 -0.89016500 0.14830700

C 1.32964800 0.25165400 0.42932600

C 3.47525000 -0.73712800 -0.06472200

C 1.92642400 1.51195000 0.49721900

C 4.07725100 0.52410400 0.00816200

H 4.08640500 -1.61599000 -0.29175800

C 3.30323400 1.65324400 0.28837100

H 5.15294500 0.62352100 -0.16022000

H 3.77013400 2.64036800 0.34159100

H -2.72862700 -2.70034000 -0.75199000

H -1.61373500 -2.42812700 1.40721100

H 0.25578400 0.14979800 0.58358800

H 1.30691400 2.38639800 0.71176100

H 1.07775700 -2.53650800 1.07883100

H 2.22218500 -3.00251600 -0.19010300

**TS2b (conformation 1)**

**G_gas_ = -3055.750428 a.u.**

**E_gas_ =** **-3056.08930267 a.u.**

Si 0.94186600 -0.22628500 2.23104800

H -0.37097700 -0.09443500 1.51793400

C 2.05326700 -1.37331500 1.23296500

C 2.10359400 -2.74753900 1.54085500

C 2.84932800 -0.90191800 0.17195400

C 2.91542200 -3.62202300 0.81190300

H 1.49625600 -3.14612900 2.35887800

C 3.65398300 -1.77607200 -0.56366700

H 2.86163700 0.15936600 -0.07740900

C 3.68779900 -3.13834300 -0.24884800

H 2.93108700 -4.68585100 1.06068200

H 4.26206900 -1.38512700 -1.38301800

H 4.31051500 -3.82201900 -0.83102700

B -1.60502600 0.09805900 -0.55212700

C -2.87637600 -0.40850400 0.22942200

C -3.30643000 0.20371100 1.41675400

C -3.65822200 -1.48031400 -0.22612700

C -4.42424200 -0.22829700 2.12981700

C -4.79262100 -1.93084800 0.44872300

C -5.17440600 -1.29959600 1.63595100

C -1.38637300 1.65403700 -0.69868800

C -2.46879000 2.53665300 -0.84869400

C -0.11592100 2.25087100 -0.68302200

C -2.31268600 3.91753600 -0.98128200

C 0.08029700 3.62457400 -0.80517800

C -1.02793700 4.46417800 -0.95116200

C -0.64091700 -0.92908300 -1.24252400

C 0.09584900 -0.62557700 -2.40237900

C -0.44743200 -2.22636800 -0.73099100

C 0.96601500 -1.52928100 -3.00960500

C 0.39178300 -3.16455600 -1.32549200

C 1.11860800 -2.80434500 -2.46063200

N 4.05742600 -0.45157600 3.59836100

H 4.00623300 -1.22474800 2.93482900

H 4.11932600 -0.86363500 4.52961100

C 5.23226100 0.37890300 3.32096600

H 5.30580000 1.13610800 4.12061600

C 5.08804600 1.08112400 1.98737400

C 5.83973600 0.69179500 0.87195300

C 5.64551000 1.30307500 -0.37254900

H 6.57763400 -0.10934500 0.97369000

C 4.68746800 2.30996800 -0.51517500

H 6.23890600 0.98417300 -1.23364100

H 4.52156600 2.78204200 -1.48690600

H 0.64970800 -0.84319700 3.55448500

H 1.48100600 1.14523400 2.37010200

F -5.51134300 -2.94441600 -0.02391300

F -6.24651200 -1.71696200 2.29361200

F -4.78609000 0.36856000 3.26122200

F -2.62517300 1.23484500 1.92557700

F -3.33869400 -2.11037300 -1.36097300

F -1.05422200 -2.60744800 0.39584200

F 0.53229800 -4.37883400 -0.80532600

F 1.95646800 -3.66836500 -3.01292600

F 1.65730300 -1.18817500 -4.09355300

F -0.02937900 0.56640300 -2.99318200

F 0.97870000 1.49610300 -0.53774400

F 1.30439300 4.15040500 -0.78474600

F -0.85754200 5.77195600 -1.07150200

F -3.36769200 4.71067800 -1.13374700

F -3.72011300 2.07145700 -0.89813100

C 3.93501500 2.71318500 0.59533600

H 3.18076600 3.49359800 0.48744600

C 4.13831000 2.10344100 1.83337300

H 3.54291000 2.40771200 2.69737000

H 6.19132600 -0.17994000 3.33123700

**TS3b (conformation 1)**

**G_gas_ = -3055.726609 a.u.**

**E_gas_ =** **-3056.06526206 a.u.**

C 1.14572600 1.16035200 1.32978200

H 1.76567500 0.41475800 0.85204300

C 1.08346000 2.50263700 0.76077000

C 1.99595000 2.87662700 -0.24741000

C 0.11946500 3.43031400 1.20403900

C 1.93966200 4.15472600 -0.79898300

C 0.06571500 4.70594300 0.64316400

H -0.59958800 3.13757700 1.97063100

C 0.97449900 5.06933700 -0.35748300

H -0.69411600 5.41491000 0.98009500

H 0.92734300 6.06777000 -0.79952400

N 2.54329400 1.40796500 2.85966900

H 2.86765100 2.36483300 2.70284900

Si 3.85970100 0.16188900 2.74150800

H 4.85661400 0.43101500 3.81508500

H 3.17780600 -1.13984000 2.93492300

C 4.58065500 0.38729600 1.03634000

C 5.44930500 1.46821500 0.77962200

C 4.17727900 -0.42313600 -0.04309000

C 5.88341400 1.74250200 -0.51909500

H 5.79184400 2.10894800 1.59912800

C 4.60723700 -0.14694100 -1.34460500

H 3.49948800 -1.26301900 0.12356800

C 5.45590500 0.93785100 -1.58212500

H 6.55390300 2.58556300 -0.70422300

H 4.25318200 -0.75980000 -2.17510700

H 5.78568700 1.15799600 -2.60062600

B -0.92253100 -0.35070100 -0.17450900

C -2.14769400 0.70247800 -0.29338300

C -2.51295200 1.45145600 0.82923400

C -2.81725200 1.05151000 -1.47044300

C -3.44244400 2.48746700 0.81548100

C -3.77342200 2.07283400 -1.53313400

C -4.08534900 2.80048900 -0.38403800

C -0.41149600 -1.07355800 -1.53404800

C -1.14146200 -2.11443300 -2.11287100

C 0.80443000 -0.77912400 -2.14486600

C -0.70749200 -2.82317900 -3.23455900

C 1.28870600 -1.46792500 -3.25953000

C 0.52735600 -2.50091500 -3.80679900

C -1.01205900 -1.44403700 1.03330400

C 0.15332700 -2.05999000 1.49744800

C -2.18423000 -1.84368500 1.68313700

C 0.19520600 -2.96726100 2.55579000

C -2.19833400 -2.75962300 2.74107100

C -0.99970700 -3.32415700 3.18352000

F 1.59271000 0.20482500 -1.65935700

F 2.48053000 -1.16182500 -3.79208800

F 0.97011400 -3.17073500 -4.86906700

F -1.44165500 -3.80497300 -3.75682400

F -2.32376900 -2.45791100 -1.58387600

F 1.34560600 -1.77223000 0.91727800

F 1.35679300 -3.48272300 2.97071100

F -0.99536100 -4.18926800 4.19522300

F -3.34260300 -3.09945100 3.33224100

F -3.36914500 -1.34462400 1.31431000

F -2.55723700 0.42018500 -2.62335800

F -4.38079000 2.36417800 -2.68307800

F -4.97783300 3.78768100 -0.43119800

F -3.69600100 3.19364700 1.92127200

F -1.91653200 1.20371700 2.02126600

H 0.01740700 0.42428300 0.16560900

H 2.00858600 1.40450200 3.73163400

H 2.73600200 2.15591300 -0.59966700

H 2.64480600 4.43795200 -1.58385200

H 0.39762400 0.82905600 2.04267700

**TS3b (conformation 2)**

**G_gas_ = -3055.724250 a.u.**

**E_gas_ =** **-3056.06256242 a.u.**

C -1.05988400 -0.15666600 2.02289800

C -1.51615200 -1.44645000 1.51737200

C -2.44265400 -1.51596800 0.45769100

C -1.02609800 -2.63998400 2.08478900

C -2.85700200 -2.75541300 -0.02644100

C -1.44832400 -3.87574500 1.60125900

H -0.28044600 -2.59055200 2.88221300

C -2.36095700 -3.93442800 0.54072700

H -1.04035000 -4.79585900 2.02387200

H -2.66699500 -4.90377500 0.14053000

N -2.32980800 0.11914500 3.63000100

H -2.72501300 -0.82035000 3.71607000

Si -3.58027800 1.42380500 3.42818900

H -4.51969900 1.32701800 4.57999100

H -2.80252100 2.68742600 3.46343000

C -4.41681800 1.15419200 1.78926500

C -5.35324000 0.11370100 1.61702700

C -4.05872100 1.92918600 0.67041200

C -5.89705000 -0.15420000 0.35997900

H -5.65993700 -0.50237600 2.46858200

C -4.59918600 1.66007300 -0.59028600

H -3.33273800 2.74040600 0.76016900

C -5.51481100 0.61630100 -0.74568900

H -6.61635900 -0.96786500 0.23874300

H -4.27877400 2.25760100 -1.44548600

H -5.93361700 0.40020300 -1.73184000

B 1.08561500 0.16152100 -0.21009700

C 0.65306900 -1.09942200 -1.13920700

C 0.81599200 -2.39794100 -0.63871600

C -0.01693500 -1.02753800 -2.36540800

C 0.33372200 -3.54210600 -1.26939100

C -0.52254200 -2.14893000 -3.03331600

C -0.35024900 -3.41648400 -2.47888300

C 0.78162500 1.65330100 -0.79579100

C 1.75163900 2.59623800 -1.15212100

C -0.53362200 2.10282700 -0.92027400

C 1.44005300 3.89598300 -1.57386700

C -0.89667300 3.38394400 -1.32360800

C 0.10593400 4.29756600 -1.65489300

C 2.54819500 0.02799600 0.48319100

C 2.77901000 0.45451200 1.78993600

C 3.65704600 -0.51290400 -0.17202800

C 4.01503500 0.35322300 2.43236200

C 4.91399300 -0.63454000 0.42371200

C 5.09327900 -0.19815800 1.73903000

F -1.55147600 1.25517000 -0.64348100

F -2.18805400 3.74672600 -1.38357300

F -0.20773700 5.53114300 -2.04427100

F 2.40649300 4.75397500 -1.89792800

F 3.05442400 2.29067000 -1.11252700

F 1.77301800 1.00293400 2.51533300

F 4.17250700 0.77242300 3.69106000

F 6.28409400 -0.30668500 2.32642500

F 5.94256000 -1.15749800 -0.24415400

F 3.54357500 -0.93461800 -1.43951800

F -0.22176200 0.15132000 -2.97315700

F -1.17149900 -2.01550300 -4.19203900

F -0.84608600 -4.49493400 -3.08598500

F 0.48020000 -4.74740700 -0.71081000

F 1.43516000 -2.58742100 0.54213700

H 0.26648700 0.03627900 0.75035800

H -1.71561300 0.26226000 4.43615400

H -2.81885700 -0.59882200 0.00683000

H -3.55976700 -2.80120700 -0.86110000

H -0.18570400 -0.09328000 2.66439100

H -1.38043700 0.75237000 1.52691400

**TS3b (conformation 3)**

**G_gas_ = -3055.726644 a.u.**

**E_gas_ =** **-3056.06453203 a.u.**

C -0.24956500 -1.45458800 -1.90595900

H -0.10099800 -0.65308900 -2.62384000

C 0.85674100 -2.33205000 -1.54225100

C 2.09145900 -2.23982000 -2.21205400

C 0.70752500 -3.25125400 -0.48505600

C 3.16191800 -3.04391800 -1.82469300

C 1.77752700 -4.05906900 -0.10745900

H -0.24340400 -3.30065000 0.05075200

C 3.00474200 -3.95323700 -0.77327100

H 1.66424800 -4.75906600 0.72354700

H 3.84900500 -4.57181500 -0.45971100

N -1.24547300 -2.63469400 -3.29230500

H -0.64436400 -2.66770100 -4.11910100

Si -2.95576300 -2.09476000 -3.59141600

H -2.83255000 -0.88067300 -4.43514600

H -3.64638200 -3.19236500 -4.32620500

C -3.71088500 -1.72832400 -1.92920000

C -4.04149500 -0.40576200 -1.57623600

C -3.88457200 -2.74602300 -0.96792500

C -4.52280300 -0.10941800 -0.29804700

H -3.89996500 0.41083200 -2.28634500

C -4.35190700 -2.44720300 0.31274600

H -3.64417200 -3.78683600 -1.21164800

C -4.67109100 -1.12684600 0.64744700

H -4.76573000 0.92468800 -0.04273000

H -4.45190800 -3.23931300 1.05824600

H -5.02452000 -0.89160000 1.65431400

B 0.52528900 0.62603800 0.15357900

C 2.02648500 0.99037200 -0.33206100

C 3.07095900 0.06772200 -0.18475200

C 2.35593000 2.13822200 -1.06231000

C 4.34172500 0.24010300 -0.73243900

C 3.62270600 2.36311900 -1.61269000

C 4.62256600 1.40229000 -1.45303300

C -0.60351400 1.79764700 0.08854600

C -0.75432600 2.71255000 1.13525500

C -1.54301000 1.91034700 -0.93407500

C -1.78818800 3.65083500 1.19569600

C -2.60408700 2.81590400 -0.91072300

C -2.73243700 3.69203800 0.16673200

C 0.37691000 -0.18323800 1.55963800

C -0.69179500 -1.05695700 1.75738200

C 1.23655700 -0.05047700 2.65486700

C -0.90284200 -1.79053900 2.92492900

C 1.06828100 -0.75796200 3.84863200

C -0.01058000 -1.63619200 3.98593100

F -1.47511800 1.11127600 -2.02401700

F -3.51519800 2.82780500 -1.89360400

F -3.74410000 4.55705200 0.21242200

F -1.89177000 4.49342100 2.22266100

F 0.12005400 2.70849700 2.15080300

F -1.60119400 -1.24881200 0.77342000

F -1.93827300 -2.63296100 3.02350300

F -0.18213500 -2.32021300 5.11586600

F 1.92101300 -0.60200400 4.86096200

F 2.28446200 0.77739900 2.59181600

F 1.43917300 3.09197900 -1.28837100

F 3.87909500 3.47691400 -2.29997000

F 5.82822900 1.58849500 -1.98775000

F 5.27551800 -0.70642300 -0.59790900

F 2.87184500 -1.07401400 0.49428800

H 0.18805600 -0.21633500 -0.73352300

H -1.17060000 -3.54914200 -2.84048300

H 2.21552300 -1.50836800 -3.01502700

H 4.12869300 -2.94389800 -2.32230000

H -1.14640900 -1.42071300 -1.30123300

**TS3b (conformation 4)**

**Ggas = -3055.727563 a.u.**

**Egas = -3056.06630550 a.u.**

C -0.17494000 1.40734200 -1.88573700

H -0.19515400 2.13098400 -1.07949400

C 1.06318900 1.18936600 -2.62443100

C 2.23492900 1.88088700 -2.26352300

C 1.11064000 0.22823400 -3.65246800

C 3.43690600 1.60266500 -2.91372000

C 2.31058900 -0.03645800 -4.30626400

H 0.21057100 -0.34387800 -3.89223300

C 3.47644300 0.64345600 -3.93106700

H 2.34957300 -0.79716300 -5.08923900

H 4.42427100 0.41177600 -4.42226200

N -1.19775000 2.70835000 -3.14557200

H -0.61754700 3.54839100 -3.20138700

Si -2.90495400 2.96470800 -2.57959800

H -2.78405500 3.96520100 -1.48937700

H -3.69862700 3.48817700 -3.72567900

C -3.52138700 1.31959000 -1.96068000

C -3.53591900 1.03679800 -0.58027100

C -3.86558000 0.29021200 -2.86106100

C -3.88444400 -0.23389800 -0.11358000

H -3.25726200 1.80751700 0.14406700

C -4.19194200 -0.98536800 -2.39644000

H -3.87109900 0.47813500 -3.94013000

C -4.20237500 -1.24629100 -1.02277000

H -3.87866100 -0.44128200 0.95706900

H -4.42727300 -1.78348000 -3.10407400

H -4.44426300 -2.24866300 -0.66252100

B 0.41714800 -0.23340500 0.53695600

C -0.44929300 0.77119600 1.48596300

C -0.17129900 2.14354500 1.48113200

C -1.56606200 0.41884600 2.25024400

C -0.93310900 3.10893500 2.13754300

C -2.36785100 1.34689000 2.92418300

C -2.05290500 2.70409200 2.86913000

C -0.16665900 -1.74313000 0.40809700

C 0.03845900 -2.67285300 1.43106900

C -0.91964500 -2.20132100 -0.67005900

C -0.49243700 -3.96360600 1.41444100

C -1.49115800 -3.47410500 -0.72542800

C -1.26923400 -4.36591500 0.32363800

C 2.02847100 -0.19315900 0.71836500

C 2.83066900 -0.69118900 -0.31336400

C 2.72331200 0.36260800 1.79625900

C 4.22197700 -0.59466100 -0.32933100

C 4.11756700 0.46473600 1.83417200

C 4.87239700 -0.01589600 0.76153600

F -1.16362000 -1.40572200 -1.73638200

F -2.25431700 -3.83478500 -1.76383200

F -1.80072100 -5.58696000 0.29112400

F -0.28140400 -4.80869200 2.42323500

F 0.73635600 -2.31001700 2.51650100

F 2.25550800 -1.26575900 -1.38386200

F 4.92676800 -1.02919700 -1.37821500

F 6.20078300 0.08690300 0.77485400

F 4.73432000 1.01150900 2.88260100

F 2.06149100 0.84513400 2.85752000

F -1.96998900 -0.85801900 2.35029600

F -3.44700700 0.94015900 3.59431100

F -2.80508400 3.60095500 3.50189000

F -0.61335600 4.40194400 2.06668800

F 0.88416200 2.60814800 0.77121400

H 0.27202300 0.27951000 -0.61465900

H -1.10666000 2.22451700 -4.04146000

H 2.20189100 2.59490400 -1.43734000

H 4.35193900 2.11604800 -2.61023800

H -0.99265600 0.70228400 -1.96740900

**TS3b (conformation 5)**

**G_gas_ = -3055.728093 a.u.**

**E_gas_ =** **-3056.0674358 a.u.**

C -0.80764400 -0.51269000 -2.12446600

H -0.24457200 0.25277700 -2.64848100

C -0.41489800 -1.92005900 -2.21726200

C 0.68328100 -2.30847000 -3.00846400

C -1.11804400 -2.89757400 -1.48577300

C 1.07009000 -3.64690400 -3.05974400

C -0.72686700 -4.23508500 -1.53961200

H -1.95476700 -2.59717400 -0.85331000

C 0.36796100 -4.61134000 -2.32530000

H -1.26374500 -4.97976600 -0.94733600

H 0.68255200 -5.65734400 -2.35794100

N -2.35042400 -0.43488000 -3.48027900

H -1.90448600 -0.30643600 -4.39174100

Si -3.57883000 0.82877500 -3.03708100

H -2.86755400 2.12705100 -3.13089300

H -4.68547500 0.75540000 -4.03261600

C -4.08414900 0.45382600 -1.28066000

C -4.15220100 1.49397100 -0.33471100

C -4.32194500 -0.86554600 -0.84331100

C -4.42924800 1.22335100 1.00912700

H -3.96447700 2.52654100 -0.63881800

C -4.58840900 -1.13855600 0.49899000

H -4.28052000 -1.70284900 -1.54807700

C -4.63651700 -0.09299200 1.42724900

H -4.45110400 2.04223600 1.73166700

H -4.73228400 -2.16985200 0.82927400

H -4.82355100 -0.30806900 2.48196500

B 0.89227600 0.25717600 0.18094700

C 0.79924300 -1.12404100 1.02505900

C -0.45761900 -1.68058300 1.28106300

C 1.87797600 -1.93087100 1.40135500

C -0.66409500 -2.93794800 1.83998400

C 1.72313200 -3.19545600 1.98316200

C 0.44261000 -3.70873100 2.19656000

C 2.36248400 0.84335600 -0.16300100

C 3.16119900 1.39419100 0.84185500

C 2.88964100 0.89867000 -1.45119800

C 4.41805600 1.95049700 0.60220300

C 4.14082000 1.44879600 -1.74447300

C 4.90897100 1.97887200 -0.70695900

C -0.10921500 1.45094200 0.65797900

C -0.58641400 2.37470800 -0.27537200

C -0.59944200 1.61205400 1.95767600

C -1.51820900 3.36759300 0.02439400

C -1.53615000 2.59029700 2.30340700

C -2.00299600 3.46837800 1.32642100

F 2.19182800 0.41023300 -2.50346300

F 4.60220000 1.47504100 -2.99718200

F 6.10432600 2.50810400 -0.96194600

F 5.15013900 2.45606900 1.59468700

F 2.71856500 1.38269000 2.10753300

F -0.18341600 2.30810700 -1.56357600

F -2.02530000 4.14985300 -0.93720100

F -2.97456400 4.33757100 1.61377000

F -2.03428100 2.65967300 3.53878000

F -0.21230700 0.78632300 2.93619000

F 3.13995300 -1.53003000 1.19795600

F 2.78827500 -3.92305400 2.31891300

F 0.27874300 -4.92404000 2.71725500

F -1.90300900 -3.43040400 1.96844900

F -1.57363100 -1.00870100 0.91527300

H 0.42272200 -0.11814100 -0.92992300

H -2.70863600 -1.39255800 -3.46182300

H 1.25507400 -1.54828600 -3.54384400

H 1.93293500 -3.93991300 -3.66236600

H -1.47300600 -0.20105100 -1.32540700

**TS6b (conformation 1)**

**Ggas = -3577.194118 a.u.**

**Egas = -3577.62526208 a.u.**

Si 2.89843300 2.38607400 -2.87805800

H 2.27311300 1.37373400 -3.76358500

C 4.19307800 1.63075600 -1.76770700

C 4.30749300 0.23222300 -1.64143500

C 5.01304100 2.44752000 -0.96356900

C 5.19823000 -0.33324100 -0.72293300

H 3.67869500 -0.43430200 -2.23527100

C 5.90546700 1.88535900 -0.04805800

C 5.99462000 0.49386200 0.07542100

H 5.24858100 -1.41898700 -0.62445000

H 6.53053300 2.53223500 0.57257100

H 6.68731800 0.05287600 0.79678300

C 0.85351700 1.41091100 -0.69990100

H -0.15646400 1.55074700 -1.05531600

C 1.19151700 1.80079800 0.66076400

C 0.28316600 2.57040000 1.41671800

C 2.40582200 1.39168600 1.24775100

C 0.58881300 2.92737400 2.72813100

C 2.70192000 1.74616000 2.56081700

H 3.10202500 0.77780400 0.67690500

C 1.79484500 2.51242000 3.30258100

H 3.63740100 1.41054800 3.01367400

H 2.02030900 2.77357800 4.33929200

N 1.54571800 3.01165300 -1.84056800

H 1.91841200 3.56304800 -1.06164900

H 3.43020000 3.53693100 -3.66181500

Si 0.17098500 3.89201300 -2.64891300

H 0.65949800 5.26721400 -2.94616200

H -0.08353100 3.11900800 -3.89000200

C -1.31743400 3.93710200 -1.52555100

C -1.47175100 4.96634100 -0.57566500

C -2.25231700 2.88036300 -1.53009600

C -2.50329100 4.92065300 0.36743800

H -0.77109600 5.80707200 -0.55611800

C -3.28132900 2.83334700 -0.58646000

H -2.16258600 2.06566500 -2.25351200

C -3.39882900 3.84549400 0.37234700

H -2.60141700 5.71893600 1.10741700

H -3.97685900 1.99365600 -0.58202500

H -4.18556000 3.78063700 1.12777000

B -0.63841200 -1.15104600 0.15784500

C -1.54862000 -0.46597900 1.31749500

C -0.99133300 -0.06801000 2.53983200

C -2.86068700 -0.02378900 1.11223000

C -1.66282200 0.70391600 3.48900600

C -3.56498400 0.76365800 2.02627900

C -2.95963100 1.14175300 3.22317100

C -1.44936300 -1.74443900 -1.12398800

C -2.14063400 -2.95503100 -1.02410100

C -1.51329600 -1.12756500 -2.37078400

C -2.85371500 -3.52400700 -2.08151200

C -2.21482200 -1.65032300 -3.45916100

C -2.88927600 -2.86311600 -3.31241400

C 0.51158000 -2.20727600 0.61416300

C 1.66238100 -2.33540800 -0.16572200

C 0.45353400 -3.04708800 1.72941300

C 2.70959700 -3.20780300 0.12977000

C 1.47845800 -3.93593400 2.06933200

C 2.61841100 -4.01393900 1.26524500

F -0.89764700 0.06263100 -2.58895600

F -2.24878000 -1.00187000 -4.62641800

F -3.56403100 -3.38434600 -4.33562600

F -3.49978800 -4.68015200 -1.93257300

F -2.14974400 -3.61391900 0.14406000

F 1.80387000 -1.58708100 -1.28711400

F 3.79583100 -3.27303800 -0.65497700

F 3.60629100 -4.85247500 1.57486600

F 1.38245700 -4.70911800 3.15080900

F -0.61079100 -3.02019500 2.53827700

F -3.51015300 -0.29214400 -0.03677000

F -4.79160000 1.21371200 1.73229500

F -3.60060600 1.93222500 4.08455700

F -1.04600700 1.09457000 4.60783100

F 0.28364600 -0.37035600 2.82971800

H 0.01536900 -0.16928100 -0.25374200

H 4.95535400 3.53823700 -1.04340000

H -0.13070800 3.49983500 3.31669200

H -0.67253600 2.86071400 0.98003000

H 1.45946900 0.67966000 -1.22464200

**TS6b (conformation 2)**

**Ggas = -3577.188782 a.u.**

**Egas = -3577.61725616 a.u.**

Si -3.42895000 1.26519300 1.09758100

H -3.43533900 1.87650100 2.45313600

C -5.10352400 1.30144400 0.27260800

C -6.28344800 1.03817700 0.99804900

C -5.20648000 1.55527200 -1.11013300

C -7.52666600 1.02459400 0.36292200

H -6.23600200 0.84348200 2.07441200

C -6.45133700 1.54157600 -1.74618300

H -4.31539400 1.76180200 -1.70690300

C -7.61042500 1.27548200 -1.01160000

H -8.43277200 0.82089000 0.93915400

H -6.51304800 1.74021600 -2.81889700

H -8.58328200 1.26560200 -1.50985000

C -0.75221200 -0.37845400 1.23070100

H -0.74508700 -1.14578500 0.46956800

C -0.29063500 -0.70392500 2.56964800

C 0.00125800 -2.04152200 2.90957300

C -0.11342900 0.31300600 3.53245500

C 0.45109900 -2.35266200 4.19065400

C 0.33740000 -0.00632300 4.81164200

H -0.30350900 1.35265000 3.25937400

C 0.61738300 -1.33848500 5.14227600

H 0.48364200 0.78528200 5.55013100

H 0.97597700 -1.58584400 6.14473800

N -2.86181400 -0.43956700 1.36094900

H -2.93605300 -0.70342600 2.34661300

H -2.43059200 1.91208000 0.21049700

Si -3.49025100 -1.77077300 0.29193600

H -4.91611200 -1.99352600 0.65295900

H -3.33731300 -1.26138300 -1.08867600

C -2.47747700 -3.30939900 0.60726000

C -2.73129700 -4.10101500 1.74574500

C -1.39448500 -3.65865600 -0.22369800

C -1.91660200 -5.19265200 2.05493600

H -3.57271400 -3.86439900 2.40546700

C -0.57209700 -4.74608200 0.08709400

H -1.17046000 -3.06303900 -1.11105900

C -0.83163500 -5.51010000 1.22886300

H -2.12389700 -5.79492700 2.94319900

H 0.28699000 -4.97439200 -0.54644500

H -0.18577100 -6.35649600 1.47615500

B 1.59592200 0.44277100 -0.42915700

C 2.33526100 -0.73854200 -1.25762700

C 2.19713800 -2.08579200 -0.93056400

C 3.07518700 -0.46862800 -2.41038600

C 2.74515900 -3.11842000 -1.69486900

C 3.65478900 -1.46457800 -3.19880800

C 3.47853700 -2.80469800 -2.83972300

C 0.49527500 1.20848300 -1.36911900

C 0.45694500 2.58123100 -1.63841200

C -0.57372700 0.48130700 -1.90367300

C -0.59387800 3.20238900 -2.32512000

C -1.63946200 1.05127500 -2.59791800

C -1.65486400 2.43074900 -2.80012900

C 2.50843600 1.38011300 0.53178200

C 1.88270100 2.27158400 1.40930900

C 3.89659300 1.29845900 0.67697800

C 2.54512000 3.02038500 2.37739800

C 4.61239600 2.03993900 1.62574000

C 3.93310800 2.90419500 2.48487600

F -0.63629100 -0.85963100 -1.72207300

F -2.66720900 0.30629400 -3.02493000

F -2.69193600 3.00051200 -3.41675800

F -0.59290500 4.51878300 -2.52600400

F 1.44029600 3.38478700 -1.22082700

F 0.53693600 2.43370000 1.35091800

F 1.86982400 3.82476700 3.20324100

F 4.59771000 3.60678400 3.40055400

F 5.93658600 1.92145800 1.72081600

F 4.62251800 0.47794500 -0.09473600

F 3.25244400 0.80408200 -2.79143700

F 4.35832300 -1.15977900 -4.28926700

F 4.01045800 -3.77392700 -3.58236900

F 2.55643800 -4.40097300 -1.35254800

F 1.48995600 -2.45771800 0.15830000

H 0.89760700 -0.15696600 0.42912600

H -0.11067500 -2.82472600 2.15967100

H 0.68079800 -3.38939100 4.44694600

H -0.80406400 0.65492300 0.91537900

**TS10b (conformation 1)**

**Ggas = -2533.125361 a.u.**

**Egas = -2533.35764910 a.u.**

C 1.21915900 -0.38792300 2.96444200

H 0.80953500 -1.32403500 2.55654100

N 0.28404700 0.71005300 2.71127400

H 0.55195100 1.24242600 1.88758300

C -1.39288600 -1.40392300 -0.34955500

C -0.75484800 -2.52028800 0.21374500

C -2.74088200 -1.59291000 -0.70053200

C -1.39851100 -3.73382400 0.44526600

C -3.41629000 -2.79804000 -0.50597800

C -2.74004200 -3.87282400 0.07731800

C 0.83305200 0.07395500 -0.96018800

C 1.65844200 1.16429700 -0.62140000

C 1.47168600 -0.94375100 -1.69663100

C 3.00533000 1.24673300 -0.96025100

C 2.82259000 -0.90789100 -2.03788300

C 3.59515700 0.19099800 -1.65644000

C -1.51855100 1.29410200 -0.30144700

C -1.38449500 2.43009600 -1.11493800

C -2.45021200 1.39170800 0.74674500

C -2.10860800 3.60263000 -0.90667400

C -3.17664800 2.55608100 0.99853200

C -3.00777100 3.66332700 0.16233800

F -0.75280500 -4.75347200 1.00162800

F 0.53136900 -2.45042100 0.57779100

F -3.36704500 -5.02159000 0.27772900

F -4.68818500 -2.93224300 -0.86584400

F -3.43642700 -0.60245500 -1.26555300

F -2.67150600 0.35879000 1.55602400

F -4.03510300 2.61840200 2.01178600

F -3.70179800 4.77045900 0.38118800

F -1.95677500 4.65332500 -1.70700600

F -0.53977200 2.41468600 -2.15105900

F 1.17535600 2.19278100 0.08979900

F 3.73334400 2.29977700 -0.61063600

F 4.88445000 0.22968600 -1.94945300

F 3.38237700 -1.90785200 -2.71109100

F 0.78418800 -2.00506500 -2.12339400

B -0.67930800 -0.01310000 -0.54118800

C 2.62365200 -0.20545000 2.40835500

C 3.26840100 -1.25331800 1.73343400

C 3.30197600 1.01593600 2.54859800

C 4.55847800 -1.08771200 1.21861000

C 4.59204600 1.18322900 2.04085600

H 2.80488700 1.84986500 3.05169900

C 5.22569000 0.13120900 1.37065500

H 5.10000300 2.14411800 2.15321500

H 6.22636400 0.26698100 0.95392200

H 2.74633100 -2.20299700 1.59571200

H 5.03819600 -1.91006100 0.68101600

H 0.25872100 1.37261500 3.48272000

H 1.28169600 -0.54573300 4.05558100

**TS10b (conformation 2)**

**Ggas = -2533.122631 a.u.**

**Egas = -2533.35279607 a.u.**

C 0.93774500 0.03930200 2.49509000

H 0.36598200 0.82739600 3.01211800

N 0.18463500 -1.21102000 2.61938400

H 0.05405300 -1.44571300 3.60368400

C 0.54481400 -0.49586500 -1.60893100

C 1.81355500 0.07741400 -1.43390800

C 0.49218100 -1.61006600 -2.46196000

C 2.96938700 -0.44004700 -2.01539300

C 1.61985800 -2.13659900 -3.09127100

C 2.86851800 -1.55139000 -2.85690000

C -0.88075100 1.60091100 -0.65159100

C -1.37172800 2.15456600 0.54274000

C -0.49291300 2.52098700 -1.63936100

C -1.45770000 3.52928800 0.75928700

C -0.58547700 3.90184100 -1.46884400

C -1.06778200 4.40629000 -0.25739900

C -1.78159500 -0.94035000 -0.27111800

C -3.13709300 -0.59419000 -0.12452700

C -1.41945100 -2.20337100 0.22837100

C -4.07217400 -1.42771300 0.48482100

C -2.31855400 -3.05207600 0.86814300

C -3.65401900 -2.66256500 0.99153300

F 4.15641300 0.10534100 -1.76669500

F 1.97166900 1.14399800 -0.63930600

F 3.95241900 -2.04999800 -3.43046200

F 1.52343200 -3.18689000 -3.89868500

F -0.67451700 -2.21056200 -2.70512400

F -0.16039700 -2.64072400 0.13091800

F -1.91729400 -4.21853700 1.36422300

F -4.52578400 -3.46224700 1.58615500

F -5.34654800 -1.06511900 0.59144300

F -3.58590300 0.57493700 -0.58910500

F -1.75292300 1.36379300 1.54823900

F -1.90630200 4.01071100 1.91370900

F -1.15442000 5.71447500 -0.07266600

F -0.22030800 4.73629600 -2.43617500

F -0.02233600 2.08622600 -2.81225900

B -0.72555100 0.05062400 -0.85964600

C 2.36063000 -0.00608500 3.02476100

C 2.59464700 -0.13955900 4.40387000

C 3.46563400 0.03550900 2.16148400

C 3.89333700 -0.23817900 4.90652000

C 4.76931700 -0.06257700 2.66067700

H 3.31015300 0.16374800 1.08839900

C 4.98730300 -0.20258400 4.03349700

H 5.61689900 -0.02622600 1.97113700

H 6.00507000 -0.27871300 4.42463300

H 1.74470500 -0.16417700 5.09381600

H 4.05532900 -0.33796000 5.98314900

H 0.72218600 -1.97926400 2.21650800

H 0.95999200 0.33228200 1.43365800

**TS10b (conformation 3)**

**Ggas = -2533.122190 a.u.**

**Egas = -2533.35258744 a.u.**

C 3.19793000 -1.21876900 -0.84407800

H 3.43513200 -2.28723900 -0.70672600

N 1.97273800 -1.12760600 -1.64543400

H 2.13852000 -1.50577200 -2.57908000

C -0.14681300 -1.06611500 1.04392100

C 0.09154000 -2.27462400 0.36678100

C 0.62408600 -0.84238100 2.19844400

C 1.05445900 -3.18990200 0.78624700

C 1.57405100 -1.74847500 2.66263100

C 1.80018200 -2.92187700 1.93479900

C -2.45221300 -0.42304000 -0.25183400

C -2.96130000 0.28201400 -1.35554300

C -3.19923200 -1.53998500 0.16045000

C -4.12884900 -0.09109000 -2.02002900

C -4.38032800 -1.93374400 -0.46756900

C -4.84289900 -1.20483200 -1.56750200

C -0.78121100 1.53786000 0.60860300

C -1.76215400 2.51078800 0.85998000

C 0.52802600 2.02106800 0.46915100

C -1.47050800 3.87068600 0.97368300

C 0.85965500 3.37132600 0.55499200

C -0.14992000 4.30272700 0.81482000

F 1.30952600 -4.28455200 0.07563700

F -0.58788100 -2.57751000 -0.73533900

F 2.73232200 -3.77566300 2.33096300

F 2.27553300 -1.50516000 3.76598200

F 0.45261000 0.27583000 2.91181700

F 1.53652400 1.17535900 0.21158100

F 2.11652600 3.77490500 0.39008500

F 0.14296200 5.59014600 0.90906400

F -2.42895200 4.75459500 1.22636400

F -3.03786200 2.15046500 1.02004900

F -2.31064700 1.34757800 -1.83247400

F -4.56658200 0.59463700 -3.07065000

F -5.95774500 -1.56943000 -2.18116100

F -5.06506900 -2.98744700 -0.03665600

F -2.80272400 -2.26433300 1.20847100

B -1.13142500 0.00703500 0.47737900

C 4.39708600 -0.49003700 -1.41946100

C 5.36566800 -1.17260600 -2.17021800

C 4.52617500 0.90086900 -1.26137600

C 6.43681700 -0.48745300 -2.75271800

C 5.59478500 1.58822600 -1.84218200

H 3.78233300 1.44945900 -0.67720800

C 6.55356500 0.89610300 -2.59066500

H 5.68320900 2.66937400 -1.70559800

H 7.39127200 1.43367900 -3.04232200

H 5.28186500 -2.25677000 -2.29542100

H 7.18457900 -1.03645500 -3.33128100

H 1.73180000 -0.14497000 -1.77871600

H 2.97079700 -0.81843000 0.15594300

**TS10b (conformation 4)**

**Ggas = -2533.125605 a.u.**

**Egas = -2533.35681935 a.u.**

C -1.74915900 0.90634000 -2.71404300

H -1.83154200 1.08106300 -3.80132100

N -0.56796100 0.08637800 -2.46011600

H -0.36107200 -0.53509500 -3.23903100

C 1.65218500 1.24295000 0.21415700

C 2.51241900 1.28897900 -0.89345500

C 1.65928600 2.36561500 1.05325300

C 3.30985900 2.39802300 -1.17735400

C 2.45743400 3.48271500 0.81434300

C 3.28576000 3.49585200 -0.31243000

C 1.40399900 -1.43477600 0.30903100

C 0.74446200 -2.53062500 -0.26691100

C 2.73445800 -1.66850200 0.69533900

C 1.34752900 -3.76927500 -0.47132900

C 3.37127600 -2.89955500 0.52690800

C 2.67305200 -3.95410300 -0.06589600

C -0.74610100 0.15524300 0.94406900

C -1.45712700 -0.85742100 1.62081800

C -1.49617400 1.31359000 0.65148500

C -2.80998300 -0.75886800 1.94033700

C -2.84293400 1.45541700 0.97641900

C -3.50902300 0.40378000 1.60699300

F 4.09909400 2.41571200 -2.24738200

F 2.58668500 0.25639300 -1.73476600

F 4.04879000 4.55088700 -0.56026500

F 2.44265300 4.52543900 1.63927900

F 0.88291900 2.38730200 2.14184100

F -0.94655600 2.33942300 -0.00120500

F -3.50126700 2.56607000 0.66545800

F -4.79438800 0.51063800 1.89636500

F -3.43837100 -1.75414500 2.55663100

F -0.84157900 -1.97758300 2.00522000

F -0.53026300 -2.41420500 -0.67271600

F 0.68056900 -4.76895600 -1.03857400

F 3.26254500 -5.12669900 -0.24157700

F 4.62724700 -3.07532700 0.92168600

F 3.44949300 -0.70041100 1.27442500

B 0.74730900 -0.00938100 0.49223600

C -3.07830400 0.37246100 -2.18875300

C -3.27160100 -0.97958500 -1.87184800

C -4.14030400 1.26531400 -1.96900300

C -4.48090700 -1.42253200 -1.32338100

C -5.34942800 0.82726700 -1.42696500

H -4.00221400 2.32716400 -2.19198700

C -5.52231100 -0.52074500 -1.09286700

H -6.15259400 1.54479000 -1.24176800

H -6.45992000 -0.86210000 -0.64789700

H -2.46644600 -1.69856100 -2.03328300

H -4.60324500 -2.47840800 -1.06803500

H -0.72268000 -0.51808300 -1.65945100

H -1.59941600 1.90528400 -2.27089200

**TS1c (conformation 1)**

**Ggas = -888.457175 a.u.**

**Egas = -888.703084653 a.u.**

Si 1.57933500 1.78651100 1.64584300

H 2.49797300 1.72006700 2.83277700

C 1.97072400 0.33116000 0.51173100

C 2.83168000 0.48367700 -0.59188300

C 1.41113300 -0.93845200 0.74111100

C 3.12369700 -0.59237600 -1.43631500

H 3.27847000 1.46037000 -0.80396400

C 1.70214400 -2.01783000 -0.09838100

H 0.71288000 -1.08982400 1.56894100

C 2.55926800 -1.84845900 -1.18957000

H 3.79376900 -0.45067300 -2.28897400

H 1.24326600 -2.99061300 0.09454000

H 2.78440900 -2.69116000 -1.84888000

N -0.34371000 2.02680200 -0.74126100

H 0.10797100 1.37358100 -1.38243600

H -0.23870500 2.96127200 -1.13814800

C -1.76155400 1.69260900 -0.55747000

H -2.12585400 2.32349500 0.27198300

C -2.63527200 1.99692100 -1.78621300

H -2.30234500 1.42411800 -2.66688600

H -3.69271100 1.74991500 -1.59904200

H -2.57438600 3.06770200 -2.04274200

C -1.87914600 0.24418600 -0.10153500

C -2.16431800 -0.06262300 1.23656500

C -1.67405200 -0.81571500 -0.99806200

C -2.24547000 -1.38954500 1.66955800

C -1.75337300 -2.14338300 -0.57141800

H -1.43913000 -0.60850600 -2.04530600

C -2.04049900 -2.43584300 0.76516800

H -1.58353600 -2.95311600 -1.28557600

H -2.10333600 -3.47424500 1.10063000

H 1.85851600 3.08337300 0.96820900

H 0.19415400 1.72513100 2.18371800

H -2.31723900 0.75050900 1.95091900

H -2.46866500 -1.60611700 2.71754800

**TS2c (conformation 1)**

**Ggas = -3095.015830 a.u.**

**Egas = -3095.38090579 a.u.**

Si 0.92677200 -0.05523200 2.08838800

H -0.44036400 0.01687500 1.47324600

C 1.91515400 -1.37459400 1.17476300

C 1.91493600 -2.70071700 1.65166400

C 2.66327800 -1.08781800 0.01729200

C 2.63401000 -3.70485000 0.99606800

H 1.34137200 -2.95887800 2.54696200

C 3.37938600 -2.09065800 -0.64170000

H 2.70633100 -0.07078800 -0.37121500

C 3.36529100 -3.40229900 -0.15675000

H 2.61030400 -4.72879800 1.37686500

H 3.95231500 -1.84061100 -1.53779100

H 3.91686500 -4.18772300 -0.67921900

B -1.76261600 0.09591400 -0.56117500

C -3.01623100 -0.29824600 0.30958000

C -3.36820000 0.41466000 1.46606000

C -3.85755300 -1.36773500 -0.03110500

C -4.46726100 0.08058500 2.25670200

C -4.97564900 -1.72231300 0.72351400

C -5.27860600 -0.99239000 1.87637200

C -1.48847100 1.62739100 -0.82624700

C -2.53954800 2.54733800 -0.97585200

C -0.19536500 2.16911100 -0.89880700

C -2.33386400 3.91319800 -1.17714000

C 0.05026700 3.52851100 -1.07899600

C -1.02805700 4.40628400 -1.22364500

C -0.87010400 -1.02030400 -1.20699200

C -0.17541900 -0.83732100 -2.41681100

C -0.70400100 -2.28138000 -0.60300600

C 0.62936600 -1.82134800 -2.98822500

C 0.07023800 -3.29690600 -1.15717600

C 0.75674100 -3.05576700 -2.34763300

N 3.99664100 -0.13940100 3.27949300

H 3.93625200 -1.00220700 2.73898300

H 4.02534600 -0.40853700 4.26402800

C 5.20950600 0.60747200 2.91672400

C 5.13763900 0.98376900 1.44216500

C 5.94708700 0.32972600 0.50434000

C 5.85301600 0.62515700 -0.86026200

H 6.65573400 -0.43086900 0.84537300

C 4.93993600 1.58387600 -1.30607200

H 6.49205100 0.10047100 -1.57549400

H 4.85890400 1.81432700 -2.37153100

H 0.73104200 -0.47137500 3.50462700

H 1.49992800 1.30543800 1.98659200

F -5.75234000 -2.73760800 0.35849500

F -6.33403600 -1.31763300 2.60884400

F -4.75414600 0.77163400 3.35557100

F -2.62722800 1.45195500 1.86782900

F -3.61403100 -2.09081500 -1.12878000

F -1.27434200 -2.54886900 0.57406600

F 0.18972900 -4.47184200 -0.54904400

F 1.53635500 -3.99324700 -2.86427100

F 1.28525000 -1.59369400 -4.12287500

F -0.28022100 0.31133900 -3.09142100

F 0.87458100 1.37595600 -0.77324400

F 1.29395700 4.00474200 -1.12319100

F -0.81029700 5.69952600 -1.40705800

F -3.36148000 4.74232900 -1.32478600

F -3.80957800 2.13415600 -0.94873300

C 4.12702200 2.24796800 -0.37939800

H 3.39827300 2.98538800 -0.71884300

C 4.22683200 1.94737100 0.98041200

H 3.57420500 2.45633400 1.69149100

C 5.35354900 1.82140900 3.83900800

H 5.47171800 1.49757100 4.88670500

H 4.46136600 2.46367900 3.78726000

H 6.23524700 2.42030300 3.56564900

H 6.12580600 -0.00759300 3.04259800

**TS2c (conformation 2)**

**Ggas =** **-3095.011430 a.u.**

**Egas =** **-3095.37620957 a.u.**

Si 1.09044000 0.13111600 -1.30695500

H -0.29552700 0.24910300 -0.69345500

C 2.08810300 -0.88726300 -0.09106700

C 2.97000200 -0.25746900 0.80711600

C 1.93947800 -2.28520700 -0.00985600

C 3.68409400 -0.99774900 1.75246600

H 3.10567500 0.82545300 0.77489600

C 2.64565800 -3.02634300 0.94135900

H 1.26259100 -2.80583800 -0.68984400

C 3.51827400 -2.38386900 1.82640800

H 4.36098100 -0.48810000 2.44168100

H 2.51174600 -4.10991400 0.99503000

H 4.06389000 -2.96276900 2.57560700

B -1.89744100 0.03372500 0.34281700

C -2.11959400 1.59680600 0.48021900

C -2.07703000 2.44353700 -0.63579000

C -2.39713500 2.21370500 1.70665800

C -2.26969000 3.82191700 -0.55241000

C -2.61022700 3.58762500 1.83119300

C -2.54499900 4.39482500 0.69264300

C -2.69661900 -0.72554500 -0.79838800

C -3.96807000 -0.31048200 -1.22129900

C -2.17403700 -1.84066300 -1.46705000

C -4.67512500 -0.95070800 -2.24233000

C -2.83692100 -2.49885800 -2.49992100

C -4.10307200 -2.04932300 -2.88691700

C -1.15604500 -0.76377300 1.48633200

C -1.45269300 -2.09423200 1.82452300

C -0.09091000 -0.18747200 2.19945600

C -0.73301900 -2.81445500 2.77917400

C 0.63781900 -0.86538700 3.17246800

C 0.32580400 -2.19731700 3.44829600

N 3.62483200 -0.10352100 -2.97426500

H 3.53897200 0.71122600 -3.58422100

H 3.20374100 -0.88451400 -3.47714600

C 5.03862100 -0.39617100 -2.70523600

C 5.72027900 0.75614700 -1.97838500

C 7.12333300 0.80486700 -1.93248700

C 7.78559000 1.81991900 -1.23972900

H 7.70535400 0.03520100 -2.44887300

C 7.05280700 2.81511200 -0.58288200

H 8.87839500 1.83826100 -1.21609900

H 7.56866500 3.61279300 -0.04234100

H 1.56792800 1.53151500 -1.41901900

H 0.83568600 -0.51430200 -2.61918800

F -2.87836200 4.13165500 3.01468400

F -2.74216600 5.70268900 0.79270100

F -2.20319600 4.58939500 -1.63679200

F -1.82253600 1.93707600 -1.84993100

F -2.48022900 1.48415300 2.82434400

F 0.30323900 1.06156700 1.92263000

F 1.64114100 -0.27198800 3.81123400

F 1.03387000 -2.87314500 4.34222600

F -1.04125700 -4.07981300 3.05168700

F -2.46641400 -2.73718700 1.23659300

F -0.96538000 -2.31372400 -1.12929100

F -2.28572900 -3.54367000 -3.11113400

F -4.75798100 -2.66584800 -3.86093700

F -5.88266500 -0.52648300 -2.60165600

F -4.57422400 0.72785200 -0.63694500

C 5.65731300 2.78361600 -0.63165300

H 5.07403600 3.55963600 -0.12854900

C 4.99679700 1.76183700 -1.32335300

H 3.90813000 1.74218600 -1.35613600

H 5.60253900 -0.54978100 -3.65002500

C 5.14382400 -1.69838900 -1.89820100

H 4.66267000 -2.53187900 -2.43714400

H 6.19796400 -1.96579900 -1.73364200

H 4.65512200 -1.59025500 -0.92121800

**TS3c (conformation 1)**

**Ggas =** **-3094.996056 a.u.**

**Egas =** **-3095.36246224 a.u.**

C 0.23353100 1.97709300 1.47421400

H 1.05760500 1.83220800 0.78347700

C -0.96559300 2.62949200 0.96678200

C -1.04131700 2.96981000 -0.40221700

C -2.07297400 2.89897500 1.79879400

C -2.19469000 3.55124700 -0.92136700

C -3.22005000 3.49186600 1.27759100

H -2.04981800 2.61802100 2.85226700

C -3.28693900 3.81039300 -0.08423700

H -4.08005500 3.67418800 1.92452000

H -4.20106700 4.24279800 -0.49791700

C 0.27220900 1.28020400 2.79690400

H -0.04557600 1.94015300 3.61646000

H -0.43331800 0.43580800 2.76939800

H 1.27130800 0.88576400 3.00798200

N 1.32322000 3.83276900 2.07325400

H 1.17788000 4.34956200 1.20416600

Si 3.05088100 3.61020400 2.53044900

H 3.72499100 4.93970100 2.57713200

H 3.01173800 2.99070900 3.88042400

C 3.80778800 2.49260900 1.24828000

C 4.19842400 3.00382400 -0.00703600

C 3.91309700 1.10651300 1.46933100

C 4.66397600 2.15391100 -1.01054900

H 4.13563400 4.07838300 -0.20919500

C 4.38446000 0.25501600 0.46639300

H 3.61386500 0.67076000 2.42569800

C 4.75858000 0.77860600 -0.77241200

H 4.94027700 2.55659000 -1.98713500

H 4.43327600 -0.81980500 0.65142400

H 5.10920900 0.11558900 -1.56598900

B -0.42712100 -0.67087700 -0.16183400

C -1.92880900 -0.25427800 -0.61027900

C -2.86892200 0.07211300 0.37376500

C -2.35762200 -0.03104000 -1.92367000

C -4.13307700 0.58749600 0.10681000

C -3.61988800 0.48284000 -2.24281500

C -4.50972600 0.80630600 -1.21958700

C 0.62189300 -0.92542000 -1.38007800

C 0.67311900 -2.16057300 -2.03323300

C 1.50697600 0.03651900 -1.86050300

C 1.54624300 -2.43878600 -3.08704100

C 2.38824100 -0.18621000 -2.92031700

C 2.41792700 -1.44062100 -3.52930700

C -0.30066000 -1.83609800 0.96554600

C 0.85700500 -1.90498900 1.74336600

C -1.25837100 -2.81609700 1.24224700

C 1.07181100 -2.85125500 2.74575700

C -1.09050400 -3.78809900 2.23322500

C 0.08381700 -3.80656400 2.99052100

F 1.53952000 1.27721800 -1.31434300

F 3.21227900 0.77670800 -3.35033500

F 3.26415900 -1.67916200 -4.53031300

F 1.55620000 -3.63443600 -3.67531100

F -0.16906700 -3.13847800 -1.66989300

F 1.84827500 -1.00632400 1.54610600

F 2.19946200 -2.84716200 3.46259300

F 0.25739300 -4.72527100 3.93875700

F -2.03425800 -4.70130700 2.46098100

F -2.40087100 -2.86228800 0.54639100

F -1.55273700 -0.28597500 -2.96631300

F -3.96773000 0.69339300 -3.51360800

F -5.69870400 1.33954000 -1.49979700

F -4.95780600 0.93415200 1.09888300

F -2.54312900 -0.06532000 1.67668300

H -0.03996700 0.36212400 0.43896900

H 0.71488100 4.25275800 2.77839600

H -0.20146400 2.72978400 -1.05614300

H -2.25526300 3.78063300 -1.98742100

**TS3c (conformation 2)**

**Ggas =** **-3094.984770 a.u.**

**Egas =** **-3095.34667958 a.u.**

C -1.92396200 0.43235900 -0.19812400

H -1.29609000 1.01584300 -0.85466600

C -2.12689200 -0.95105900 -0.57695000

C -1.73914000 -1.36779200 -1.86998100

C -2.65519300 -1.89744800 0.32391900

C -1.88640100 -2.69653400 -2.25320000

C -2.78324100 -3.22918800 -0.05955100

H -2.92960200 -1.59577500 1.33481200

C -2.39888700 -3.63036100 -1.34503000

H -3.16575900 -3.96552400 0.65081100

H -2.48126600 -4.68001900 -1.63602200

C -2.27466200 0.98999900 1.14065100

H -3.31642600 0.76668900 1.40967200

H -1.62858800 0.52339200 1.89834500

H -2.10803000 2.07477000 1.17332900

N -3.52775600 1.44757300 -1.37919800

H -3.57010200 2.36638900 -0.93375900

Si -5.11386600 0.60880900 -1.50222100

H -6.02601900 1.42488500 -2.35421800

H -4.83999300 -0.70442300 -2.13385100

C -5.77166900 0.42245600 0.23686900

C -6.15858200 1.55289100 0.98454500

C -5.83250500 -0.84108400 0.85536000

C -6.57194200 1.42810900 2.31319100

H -6.13974100 2.54950500 0.53049000

C -6.25083000 -0.96925400 2.18342600

H -5.53923700 -1.73511500 0.29957600

C -6.61468800 0.16542700 2.91494800

H -6.86406600 2.31546100 2.88024700

H -6.29025500 -1.95705400 2.64947000

H -6.93675300 0.06631900 3.95458000

B 1.21793600 0.20906500 0.14344200

C 1.34317600 1.79351800 -0.23462400

C 0.78482900 2.28577300 -1.41958800

C 1.90683600 2.78177000 0.58190100

C 0.77105200 3.62657900 -1.79702400

C 1.93134400 4.14154900 0.24535400

C 1.35974000 4.56984100 -0.95252400

C 1.91965700 -0.27615400 1.52845700

C 3.31102000 -0.27611500 1.65652200

C 1.21796400 -0.80175600 2.61013400

C 3.97867100 -0.74312100 2.78870000

C 1.83858700 -1.28618200 3.76577600

C 3.23069100 -1.25725200 3.85250400

C 1.62875100 -0.83533300 -1.03982600

C 1.14381600 -2.14562200 -0.97078000

C 2.44055400 -0.56252700 -2.14494500

C 1.38147800 -3.11338600 -1.94725400

C 2.71714700 -1.50331500 -3.14240400

C 2.17906300 -2.78872800 -3.04484600

F -0.13322800 -0.87138600 2.59105500

F 1.11749600 -1.77783600 4.77724300

F 3.84420300 -1.71313500 4.94415800

F 5.30941600 -0.71147100 2.86910900

F 4.05860600 0.21003700 0.65400200

F 0.37051200 -2.51920600 0.06398400

F 0.82590200 -4.32718100 -1.85720700

F 2.41695000 -3.69285700 -3.99509300

F 3.48945700 -1.18909400 -4.18349900

F 2.99097200 0.64880200 -2.30423000

F 2.45321300 2.47202600 1.76500900

F 2.48590000 5.03269700 1.06631100

F 1.36220200 5.86041400 -1.28133300

F 0.19498200 4.01353300 -2.93917700

F 0.17575500 1.43053100 -2.28645300

H -0.00630500 0.02440300 0.30021100

H -3.04180300 1.56779800 -2.27110600

H -1.29049200 -0.64221000 -2.55206200

H -1.56646300 -3.01822500 -3.24613900

**TS3c (conformation 3)**

**Ggas =** **-3094.992486 a.u.**

**Egas =** **-3095.35705439 a.u.**

C 0.93069000 -1.30846200 2.01169900

C 0.05026900 -2.45239400 1.83686100

C -1.06030300 -2.66818900 2.68018000

C 0.28502500 -3.35020700 0.77225900

C -1.92289000 -3.73567300 2.44194400

C -0.57379900 -4.42160200 0.54380000

H 1.15180200 -3.20143400 0.12843000

C -1.68460400 -4.61110900 1.37434200

H -0.38941900 -5.09492800 -0.29630500

H -2.37123600 -5.43996100 1.18564400

N 2.78308600 -2.44697600 2.68557200

H 3.02595200 -1.97759600 3.55913500

Si 4.10936600 -2.48702100 1.47119900

H 5.32913000 -3.06669300 2.10528500

H 3.62456600 -3.37031700 0.38063300

C 4.37967800 -0.74238200 0.87186900

C 4.78111200 0.27432900 1.76355900

C 4.17530200 -0.40743000 -0.48104100

C 4.97041500 1.58411000 1.31826400

H 4.95411200 0.04801500 2.82158600

C 4.37866200 0.90064600 -0.92914800

H 3.83183400 -1.16429000 -1.18988800

C 4.77804600 1.89487300 -0.03206700

H 5.26336400 2.36695000 2.02161200

H 4.20113800 1.15224200 -1.97744700

H 4.90953900 2.92138200 -0.37964700

B -0.76908700 0.39252800 -0.08505100

C -0.98391700 -0.84957700 -1.11039800

C 0.12662200 -1.53714600 -1.60613200

C -2.21503700 -1.40704500 -1.47484900

C 0.06237600 -2.67898500 -2.39916500

C -2.33470800 -2.55386200 -2.26923200

C -1.18717300 -3.20110100 -2.72959800

C -2.12669100 1.14529200 0.40276900

C -2.80087000 2.01980000 -0.45453900

C -2.68990700 1.00126800 1.66710000

C -3.95752200 2.71192000 -0.09267200

C -3.84331100 1.67251800 2.08169700

C -4.48076700 2.53752900 1.19179900

C 0.33668700 1.50134700 -0.54400300

C 0.96917000 2.28195300 0.42663500

C 0.70683900 1.77996900 -1.86307800

C 1.91956800 3.26073700 0.13776200

C 1.65795100 2.74661300 -2.20131400

C 2.25959800 3.49773400 -1.19269700

F -2.12919600 0.17029600 2.57795400

F -4.33653400 1.49467800 3.30996000

F -5.58210600 3.18899600 1.56175300

F -4.56708100 3.53064800 -0.95001000

F -2.34611800 2.19915400 -1.70403500

F 0.68407600 2.09172100 1.73160300

F 2.54431900 3.92753000 1.11292900

F 3.21927200 4.37876900 -1.49041700

F 2.02053400 2.93751700 -3.47162100

F 0.16403300 1.10042100 -2.88165300

F -3.36827800 -0.86787100 -1.05736100

F -3.53520200 -3.04563500 -2.57497800

F -1.28210900 -4.32092500 -3.44584900

F 1.17991000 -3.32661400 -2.74957600

F 1.37163000 -1.14030800 -1.24809600

H -0.27542100 -0.16374700 0.92947000

H 2.37870900 -3.35452400 2.92297400

H -1.28003500 -1.96861600 3.48621500

H -2.79631900 -3.87932500 3.08181600

C 0.89408400 -0.41112200 3.20539000

H -0.01045800 0.20943400 3.16915600

H 0.85231900 -0.99521700 4.13668800

H 1.75833600 0.26393100 3.21129100

H 1.53688200 -1.01232000 1.15942800

**TS3c (conformation 4)**

**Ggas =** **-3094.984263 a.u.**

**Egas =** **-3095.34725079 a.u.**

C 1.63275500 -1.17928800 -0.76640700

C 2.30763400 -0.20077700 0.06857000

C 2.78416500 1.01663200 -0.46054800

C 2.45181700 -0.44630800 1.45199900

C 3.36115400 1.96534600 0.37792500

C 3.04338500 0.49843500 2.28513400

H 2.08740000 -1.38454600 1.87296400

C 3.49065800 1.71137100 1.74898600

H 3.12503500 0.30100100 3.35633500

H 3.93671700 2.46449200 2.40316100

N 3.08833100 -2.87953700 -0.62685800

H 3.22899300 -2.86201700 0.38457800

Si 4.61482200 -2.81683900 -1.57601400

H 5.53407300 -3.87879900 -1.07524200

H 4.21818800 -3.08508400 -2.98172300

C 5.37465600 -1.11951700 -1.40028500

C 5.93093400 -0.69879500 -0.17641500

C 5.39726800 -0.22489100 -2.48748300

C 6.48163700 0.57661000 -0.04069600

H 5.93401400 -1.36806100 0.68971400

C 5.95368200 1.05114400 -2.35571300

H 4.98066400 -0.52467200 -3.45384900

C 6.49527300 1.45237300 -1.13162800

H 6.89985300 0.88975500 0.91884500

H 5.96454600 1.73200900 -3.21044000

H 6.92863700 2.45012700 -1.02592000

B -1.26850800 0.04692800 0.00472500

C -0.89206600 0.33613000 1.55947500

C -0.38702000 -0.70166700 2.34728400

C -0.90358000 1.58620900 2.18812000

C 0.08493600 -0.54535200 3.64778400

C -0.44383300 1.79451600 3.49409100

C 0.06435600 0.72191300 4.22776600

C -1.83481200 1.32290700 -0.83291100

C -3.15716700 1.75346600 -0.69006000

C -1.06844500 2.05292000 -1.73749300

C -3.69539900 2.83114800 -1.39563000

C -1.55794100 3.13671100 -2.47094500

C -2.88619900 3.52689100 -2.29816000

C -2.21729500 -1.25801400 -0.24184500

C -2.15561300 -1.94214500 -1.45767300

C -3.14930600 -1.75873200 0.67229900

C -2.93795200 -3.05707400 -1.76573800

C -3.95656800 -2.86997100 0.41138700

C -3.85098100 -3.52335800 -0.81837600

F 0.23295100 1.73799800 -1.94706800

F -0.77305700 3.79694400 -3.32717800

F -3.37806200 4.55724400 -2.98478300

F -4.96266600 3.20565200 -1.21922400

F -3.96411800 1.13362400 0.18367800

F -1.29402900 -1.53353200 -2.41541000

F -2.82147700 -3.67554800 -2.94440000

F -4.61087700 -4.58523100 -1.08359300

F -4.82731500 -3.31117900 1.31991600

F -3.30515700 -1.17843100 1.86947000

F -1.35020300 2.67623900 1.54916600

F -0.46782600 3.01170300 4.03734600

F 0.55654400 0.90991200 5.45261600

F 0.65669300 -1.57008200 4.29278200

F -0.25574300 -1.94254300 1.81767400

H -0.16131900 -0.24230200 -0.49889700

H 2.50581700 -3.69384200 -0.83583100

H 2.66448700 1.23828400 -1.51956500

H 3.70932200 2.91253800 -0.03821900

H 0.97620600 -1.88019700 -0.25748100

C 1.57631800 -1.08745400 -2.25383100

H 1.20451800 -2.01837600 -2.69677300

H 0.88053100 -0.28627200 -2.53556600

H 2.55941000 -0.82936600 -2.67035200

**TS3c (conformation 5)**

**Ggas = -3094.994025 a.u.**

**Egas =** **-3095.35876661 a.u.**

C 1.10538200 -0.51195800 -2.17258800

C 1.69965700 -1.54881700 -1.34785800

C 2.38728300 -1.18136700 -0.16967100

C 1.57526800 -2.91897300 -1.66897600

C 2.91629400 -2.15740100 0.66877300

C 2.11958800 -3.89049700 -0.83447400

H 1.02431200 -3.22793700 -2.55807600

C 2.78027200 -3.51179300 0.34171700

H 1.99621300 -4.94761300 -1.07627800

H 3.17422000 -4.27786600 1.01393800

N 2.80306700 -0.21742000 -3.61518700

H 3.27249600 -1.10829900 -3.44201700

Si 3.86414000 1.22560000 -3.40730800

H 4.98214900 1.13520300 -4.39166100

H 2.99725700 2.39382200 -3.70593000

C 4.47519100 1.25395300 -1.64618400

C 5.36391600 0.27070700 -1.16484000

C 3.99544300 2.22206500 -0.74505600

C 5.74023300 0.24423800 0.17907500

H 5.76467100 -0.49290800 -1.83978600

C 4.36379300 2.19360400 0.60338300

H 3.29475900 2.99052200 -1.07623700

C 5.23368400 1.20273000 1.06611600

H 6.42380800 -0.52870800 0.53910000

H 3.94849300 2.93901600 1.28499500

H 5.51991500 1.17562700 2.12071700

B -1.06621100 0.10492400 0.16732200

C -0.75771300 -1.20924300 1.06767100

C -0.85773800 -2.47380300 0.47661400

C -0.23261700 -1.21310700 2.36499900

C -0.47323900 -3.65914600 1.09517300

C 0.16662400 -2.37827900 3.02957800

C 0.05691400 -3.60978700 2.38553700

C -0.81286900 1.53468700 0.90113600

C -1.72080000 2.06468100 1.82173500

C 0.31262300 2.31500200 0.66777500

C -1.52261500 3.28067700 2.47993100

C 0.56512000 3.53218500 1.30158000

C -0.36532900 4.02199900 2.21767300

C -2.47435100 0.12120200 -0.64263700

C -2.57777700 0.89286000 -1.80352700

C -3.63053400 -0.57572300 -0.28253600

C -3.73313100 0.97128900 -2.58301100

C -4.81274300 -0.52530100 -1.02728000

C -4.86387700 0.25490300 -2.18518400

F 1.26007300 1.90193300 -0.20996900

F 1.68934300 4.21799700 1.04317700

F -0.15372500 5.18071800 2.83907200

F -2.41360700 3.74089000 3.35755100

F -2.82815800 1.37531200 2.12671200

F -1.51219700 1.60635000 -2.23008000

F -3.76640000 1.71337700 -3.69304500

F -5.98184100 0.31175800 -2.90732300

F -5.89171700 -1.20913200 -0.64580600

F -3.64497800 -1.33958900 0.81716500

F -0.05907000 -0.06895400 3.04454000

F 0.68039100 -2.31846700 4.25990500

F 0.47549300 -4.72608200 2.98260600

F -0.53842300 -4.82563400 0.44654200

F -1.30411600 -2.58181700 -0.79330100

H -0.20872400 0.01064600 -0.73319000

H 2.36086800 -0.27779600 -4.53455500

H 2.47533300 -0.12609600 0.08977900

H 3.42281900 -1.86139800 1.58938100

C 0.06108200 -0.78910300 -3.20372300

H 0.35880800 -1.59345800 -3.89184600

H -0.84520700 -1.13795600 -2.68346900

H -0.19709200 0.11691000 -3.76406200

H 1.24871300 0.51437200 -1.85403500

**TS6c (conformation 1)**

**Ggas =** **-3616.455357 a.u.**

**Egas =** **-3616.90738946 a.u.**

Si 4.59604800 -0.67153000 -2.30140800

H 4.08828400 -2.06788100 -2.39589200

C 6.38228000 -0.70298100 -1.74598400

C 6.88012600 -1.80404800 -1.02167800

C 7.24314000 0.38775200 -1.98155500

C 8.19390300 -1.81427300 -0.54396300

H 6.23787900 -2.66970600 -0.83012700

C 8.55697300 0.37904800 -1.50628900

C 9.03280400 -0.72184400 -0.78571300

H 8.56539500 -2.67764300 0.01395000

H 9.21276200 1.23161800 -1.70018600

H 10.06105200 -0.72944000 -0.41509200

C 1.37047200 -0.56978100 -1.05751700

H 1.13928600 0.41010800 -0.65102800

C 1.55304000 -1.62375500 -0.09533800

C 1.70814400 -1.27267800 1.26889200

C 1.59157500 -2.98752600 -0.46467700

C 1.91697700 -2.25706500 2.22691500

C 1.76456400 -3.96860000 0.50555000

H 1.45619800 -3.27493900 -1.50827300

C 1.92931400 -3.60497000 1.84906500

H 1.76424900 -5.02299500 0.22115300

H 2.05528200 -4.37846000 2.61021600

C 1.16301500 -0.75581300 -2.51362500

H 1.75801900 -1.57790500 -2.93184300

H 0.09887700 -0.99146500 -2.67447600

H 1.36154600 0.17793200 -3.05427800

N 3.58427800 0.18171700 -1.10034000

H 3.74798000 -0.21374100 -0.17083300

H 4.41755000 0.06586400 -3.57974100

Si 3.49741000 1.97396600 -0.95697200

H 4.86365600 2.54136400 -1.14604000

H 2.58770100 2.46295800 -2.02256200

C 2.81815400 2.27879000 0.75739000

C 3.55568200 1.86016700 1.88473400

C 1.52223100 2.79259000 0.95598500

C 3.00213400 1.91991500 3.16634900

H 4.57206300 1.46845100 1.76582200

C 0.97194100 2.86207600 2.23987200

H 0.92406800 3.11311300 0.10057700

C 1.70358100 2.41208300 3.34287700

H 3.58126700 1.57865900 4.02831100

H -0.04158700 3.23931500 2.37606800

H 1.24749200 2.43455600 4.33523500

B -1.91486000 -0.07466900 -0.20351700

C -1.67279100 -0.15290400 1.40814800

C -1.29175400 -1.37051900 1.98605200

C -1.69081100 0.92564400 2.29600100

C -0.94936700 -1.52367400 3.32906600

C -1.32562900 0.82636300 3.64199700

C -0.93852900 -0.40589700 4.16382300

C -2.30374100 1.40992300 -0.76148500

C -3.59054400 1.93152200 -0.59091200

C -1.41392400 2.25639400 -1.41553900

C -3.97231900 3.20253000 -1.02408500

C -1.73710900 3.54167200 -1.85618000

C -3.03335900 4.01763400 -1.66357100

C -2.96042800 -1.18154800 -0.79671500

C -2.90992900 -1.51795000 -2.15065200

C -3.96220800 -1.82840600 -0.06757600

C -3.75741000 -2.44429300 -2.76022800

C -4.83645000 -2.76433300 -0.62920500

C -4.73493900 -3.07324600 -1.98675000

F -0.12798200 1.87727700 -1.64068500

F -0.81350200 4.31273600 -2.43995300

F -3.37143300 5.23846700 -2.07565300

F -5.21084000 3.65370000 -0.82594800

F -4.51482700 1.20973600 0.05878200

F -1.99487900 -0.92746000 -2.95563300

F -3.64784700 -2.72824000 -4.06079000

F -5.55988500 -3.96247700 -2.53899200

F -5.77041900 -3.35896300 0.11487700

F -4.12993400 -1.57120200 1.23742000

F -2.03675100 2.16018300 1.88523700

F -1.29064700 1.91676300 4.41798600

F -0.54513200 -0.51224500 5.43402300

F -0.55287600 -2.71043400 3.80375200

F -1.20649800 -2.47284100 1.22152600

H -0.80753000 -0.37756700 -0.67258500

H 6.88855600 1.25582800 -2.54538200

H 2.02443000 -1.98158800 3.27712200

H 1.65968800 -0.22221100 1.56275200

**TS10c (conformation 1)**

**Ggas = -2572.388665 a.u.**

**Egas = -2572.64703884 a.u.**

C -1.61432400 0.52903400 2.87139400

N -0.72320100 -0.05976200 1.85661300

H -0.80969100 -1.07532400 1.87260100

C 1.29333800 1.43111300 -0.57004000

C 0.34198600 2.42740000 -0.29964100

C 2.62562300 1.87245800 -0.62388100

C 0.67258400 3.75504600 -0.04380700

C 2.99836900 3.19899400 -0.40293000

C 2.01426300 4.14416600 -0.10118200

C -0.46363100 -0.48066200 -1.40256400

C -1.23621500 -1.56871600 -0.95679500

C -1.04849000 0.29745400 -2.41665900

C -2.52666500 -1.82775500 -1.41564300

C -2.32595600 0.05634100 -2.92113400

C -3.07294900 -1.00687000 -2.40566400

C 1.87083100 -1.20106400 -0.18878700

C 2.01228200 -2.43748400 -0.84055400

C 2.62240800 -1.03659500 0.98870200

C 2.83814700 -3.45591100 -0.36727800

C 3.44293900 -2.04092400 1.50264700

C 3.55401100 -3.25376100 0.81629400

F -0.26957800 4.64464500 0.25250200

F -0.96533800 2.12642000 -0.24469200

F 2.34997000 5.40468300 0.12462200

F 4.27250000 3.56859000 -0.47102300

F 3.60809700 1.01229800 -0.90225600

F 2.56569000 0.10023900 1.67810100

F 4.12494700 -1.85438100 2.62818900

F 4.33956000 -4.21078500 1.28641100

F 2.95020200 -4.60684700 -1.02277200

F 1.34880900 -2.67684700 -1.97575800

F -0.77333800 -2.38595800 -0.00293600

F -3.23584500 -2.83574800 -0.92442300

F -4.29823000 -1.23370000 -2.85243200

F -2.83961500 0.82564100 -3.87452300

F -0.38405900 1.33267700 -2.93512600

B 0.90742500 -0.08739900 -0.72692000

C -3.07963000 0.20109900 2.58638600

C -3.66585100 0.57518000 1.36284800

C -3.85432000 -0.52870800 3.49719900

C -4.97784200 0.21251900 1.05412700

C -5.17418600 -0.88637100 3.19656600

H -3.41655800 -0.83018200 4.45371600

C -5.73842800 -0.52208100 1.97202100

H -5.75962000 -1.45948500 3.92041200

H -6.76503000 -0.80890200 1.73091500

H -3.08748400 1.15358700 0.63703900

H -5.41031500 0.50268300 0.09261800

H -1.07743700 0.22036600 0.94574800

C -1.35596000 2.03544200 2.98248500

H -1.59314800 2.55205400 2.04047900

H -1.97223500 2.48193700 3.77746600

H -0.29346700 2.21350600 3.20912800

H -1.34767100 0.07068400 3.83861300

**TS10c (conformation 2)**

**Ggas = -2572.386801 a.u.**

**Egas = -2572.64238536 a.u.**

C 3.34679500 0.27707300 0.72088900

N 2.69310700 1.57620800 0.83664900

H 2.42733500 1.77983500 1.79923300

C -1.22638800 -1.56931400 -0.54120700

C -0.01000700 -2.26350700 -0.64192700

C -2.38082100 -2.36701500 -0.47827000

C 0.07187600 -3.65491100 -0.66324700

C -2.34274700 -3.76069300 -0.51794400

C -1.10575800 -4.40638600 -0.60737900

C -0.30103300 0.85524900 -1.34421100

C 0.25388500 2.05082600 -0.86020400

C 0.13809700 0.44272200 -2.61610900

C 1.20377500 2.78091500 -1.57141100

C 1.06206400 1.16320400 -3.36696700

C 1.60851100 2.33450300 -2.82832600

C -2.34174000 0.70214500 0.44775700

C -2.98059100 1.89970500 0.08336400

C -2.70010600 0.17180700 1.69860900

C -3.92143500 2.53275300 0.89495700

C -3.62319300 0.78473800 2.54511500

C -4.23927800 1.97108000 2.13531400

F 1.24871800 -4.26800900 -0.73727600

F 1.14819400 -1.59491500 -0.69231700

F -1.04956300 -5.72881500 -0.63682400

F -3.46100400 -4.47617300 -0.47055000

F -3.58617900 -1.79764900 -0.39458300

F -2.13352300 -0.95505400 2.13951900

F -3.92143900 0.25753100 3.72779800

F -5.12394400 2.56310400 2.92250400

F -4.51375600 3.65662800 0.50597700

F -2.71653200 2.47207000 -1.09309900

F -0.09405800 2.51841500 0.33719800

F 1.76567800 3.86391500 -1.04139700

F 2.52261800 3.00943800 -3.51015600

F 1.44219600 0.74806300 -4.57173700

F -0.34925100 -0.67570500 -3.16306700

B -1.29277600 0.00142600 -0.48722400

C 4.50209700 0.01554900 1.68897000

C 5.25866600 1.05563100 2.24442500

C 4.84742600 -1.30670900 2.01347600

C 6.33638700 0.78537200 3.09519700

C 5.92105800 -1.58258900 2.86236400

H 4.26180300 -2.13099600 1.59356600

C 6.67225500 -0.53444900 3.40660300

H 6.17097200 -2.61891600 3.10503000

H 7.51149000 -0.74696200 4.07374700

H 5.00052700 2.09301300 2.01786800

H 6.91363000 1.61191800 3.51829100

H 3.30001400 2.33540000 0.52693100

C 3.79968500 0.07428600 -0.73145700

H 4.55980200 0.82512300 -1.00700600

H 4.24612200 -0.92083400 -0.87014700

H 2.94295900 0.17068600 -1.41295400

H 2.57717100 -0.48635100 0.92682000

**TS2d (conformation 1)**

**Ggas =** **-3134.275821 a.u.**

**Egas = -3134.67076983 a.u.**

Si 0.82488200 0.11533600 1.91088900

H -0.53573500 0.07734500 1.24759900

C 1.69101800 -1.45000600 1.32773000

C 1.51128300 -2.64896500 2.04561100

C 2.46915600 -1.48989700 0.15631700

C 2.08392900 -3.84642200 1.60734200

H 0.90563700 -2.65487400 2.95699300

C 3.03734400 -2.68751800 -0.28684100

H 2.64438000 -0.58073200 -0.41819400

C 2.84378800 -3.86997300 0.43359600

H 1.92039400 -4.76734600 2.17246100

H 3.63339500 -2.69257800 -1.20275600

H 3.27583100 -4.80843200 0.07734600

B -1.75110200 0.08514900 -0.59558100

C -3.04635000 -0.04857600 0.29986000

C -3.30274100 0.82264800 1.36911400

C -4.01328400 -1.03757900 0.07118500

C -4.42969500 0.71418200 2.18263500

C -5.16218700 -1.16934700 0.85220900

C -5.36792100 -0.28739700 1.91608500

C -1.30427600 1.53053400 -1.06100500

C -2.23089700 2.56690200 -1.26324900

C 0.04177900 1.88493600 -1.24672000

C -1.85828300 3.87013700 -1.59908400

C 0.45463700 3.17947500 -1.55257100

C -0.50372700 4.17946700 -1.73709200

C -1.02862300 -1.20859800 -1.12216200

C -0.36121100 -1.26618400 -2.35754200

C -0.98394400 -2.38916400 -0.35756200

C 0.32289900 -2.39759100 -2.80024400

C -0.33168600 -3.54541900 -0.77682100

C 0.34411200 -3.53994500 -1.99742700

N 3.50500100 0.41763900 3.09026100

H 3.86163700 -0.52329700 2.91967400

H 3.40590200 0.51742700 4.10248900

C 4.44410700 1.43801500 2.56714100

C 4.66655100 1.12439000 1.07402200

C 5.47610500 0.03610300 0.70279200

C 5.67137900 -0.30472500 -0.63691000

H 5.96151600 -0.57221800 1.46868100

C 5.05184000 0.43620500 -1.64704700

H 6.30380100 -1.15940700 -0.89015800

H 5.19763200 0.17088200 -2.69721200

H 0.54351000 0.07315500 3.37088600

H 1.39656400 1.39870400 1.45643900

F -6.05817900 -2.11739400 0.59374800

F -6.45164200 -0.39943200 2.67181600

F -4.62442500 1.55212900 3.19691100

F -2.43896600 1.80180800 1.65921300

F -3.86693100 -1.90189700 -0.93852400

F -1.55514800 -2.43448400 0.84872800

F -0.31798200 -4.63539000 -0.01689000

F 1.01169400 -4.61540000 -2.38986000

F 0.96142600 -2.39616300 -3.96791200

F -0.36947900 -0.21344600 -3.18154100

F 1.00540900 0.96918400 -1.10433200

F 1.74966800 3.47727700 -1.67454600

F -0.12789100 5.41245800 -2.04293400

F -2.77586100 4.81309300 -1.78634400

F -3.54183800 2.33771100 -1.15026300

C 4.23876400 1.51627200 -1.29788500

H 3.72852400 2.09679700 -2.06817400

C 4.05139700 1.85597300 0.04639900

H 3.39827500 2.69682500 0.27498500

C 3.79410400 2.81223200 2.79022100

H 3.66760100 2.99195600 3.87003600

H 4.42265400 3.61893700 2.38479300

H 2.80019900 2.86974100 2.32876100

C 5.78957700 1.41768000 3.32911300

H 6.26286300 0.42518100 3.30162900

H 6.50051900 2.14514800 2.90639800

H 5.62468700 1.67532000 4.38827200

**TS2d (conformation 2)**

**Ggas = -3134.278489 a.u.**

**Egas = -3134.66984704 a.u.**

Si 0.80950200 -0.12972400 2.05558300

H -0.58127200 -0.05638700 1.50672500

C 1.83094700 -1.34779700 1.04281100

C 1.96656800 -2.67936900 1.48465700

C 2.48426500 -0.97730100 -0.14834400

C 2.72816700 -3.60558500 0.76522400

H 1.46863800 -3.00298900 2.40355000

C 3.25172000 -1.89849300 -0.86519500

H 2.41200500 0.04282200 -0.52111900

C 3.37380100 -3.21616400 -0.41235000

H 2.80906700 -4.63618700 1.11945700

H 3.75241600 -1.58117400 -1.78293600

H 3.96369300 -3.93980000 -0.98021900

B -1.98163600 0.09819400 -0.52195400

C -3.19388500 -0.41960400 0.34138600

C -3.55724500 0.19283900 1.55069300

C -3.98542600 -1.50677500 -0.05800500

C -4.62146400 -0.25156800 2.33471700

C -5.06839200 -1.96990200 0.68874100

C -5.38454400 -1.33702200 1.89441800

C -1.79116100 1.65689100 -0.67802700

C -2.89102800 2.52818400 -0.74776300

C -0.52972800 2.27186500 -0.70949600

C -2.75962000 3.91500300 -0.83359400

C -0.35695900 3.65365500 -0.76932000

C -1.48226800 4.48042400 -0.83785000

C -1.03952700 -0.92504500 -1.24524100

C -0.37522400 -0.62830500 -2.44956900

C -0.79613600 -2.21076300 -0.72408400

C 0.46567700 -1.53222300 -3.09640700

C 0.02170100 -3.14625300 -1.35151800

C 0.66880900 -2.79570400 -2.53699600

N 4.25566600 -0.09367100 2.96885800

H 3.93212800 -0.91157300 2.45100600

H 4.41260900 -0.40211100 3.92950100

C 5.52321900 0.39313300 2.38885600

C 5.20956200 0.94977300 0.98845700

C 5.96794400 0.63782700 -0.14876800

C 5.62763900 1.15306800 -1.40605400

H 6.83129300 -0.02395500 -0.07470000

C 4.51566800 1.98485100 -1.54978400

H 6.23277800 0.88999500 -2.27794200

H 4.23654000 2.36981800 -2.53408400

H 0.70395600 -0.63927400 3.45173500

H 1.35481100 1.24806300 2.03129800

F -5.79933600 -2.99714900 0.26775700

F -6.40651300 -1.76642900 2.62017700

F -4.92015600 0.34605200 3.48380500

F -2.86017000 1.23607600 2.01173200

F -3.72643800 -2.13928800 -1.20675500

F -1.32823500 -2.57957300 0.44353100

F 0.22538000 -4.34344700 -0.81371900

F 1.49005000 -3.65380100 -3.12186800

F 1.09055600 -1.19907700 -4.22212900

F -0.54762100 0.55604800 -3.04307800

F 0.58102400 1.52704400 -0.65477600

F 0.85889000 4.19906400 -0.76786300

F -1.33613500 5.79421500 -0.90896600

F -3.83068600 4.69713000 -0.90944200

F -4.13638700 2.04535500 -0.75059500

C 3.75452200 2.31223100 -0.42159900

H 2.84537800 2.90476800 -0.53085900

C 4.10569600 1.80537000 0.82977800

H 3.48934100 2.02941600 1.70098200

C 6.03969900 1.53891900 3.28145600

H 6.25721700 1.16871400 4.29814000

H 6.96495700 1.96794100 2.86849300

H 5.28975400 2.33901500 3.36055500

C 6.57876700 -0.72959900 2.35112000

H 6.25078300 -1.55785600 1.70295700

H 7.55985600 -0.37816900 1.99479600

H 6.72095200 -1.12954300 3.36787900

**TS3d (conformation 1)**

**Ggas = -3134.252988 a.u.**

**Egas = -3134.64516182 a.u.**

C -1.60367300 0.40401300 2.09488200

C -1.43384000 1.65370800 1.38354000

C -0.58118800 2.66930900 1.88882600

C -2.09020800 1.88440800 0.15035200

C -0.39277300 3.85019800 1.18321800

C -1.92364500 3.08118400 -0.53550300

H -2.73523600 1.11827100 -0.27152600

C -1.06880400 4.06328500 -0.02581100

H -2.41688500 3.22780700 -1.49783400

H -0.91073600 4.98957600 -0.58311400

C -2.08965500 -0.83220100 1.43099200

H -2.64992400 -0.67605400 0.50688800

H -1.18428900 -1.41370000 1.18808100

H -2.68045100 -1.44757000 2.12012400

N -3.76383800 0.96463300 3.12675600

H -3.83239500 0.18704400 3.78431300

Si -5.22854600 1.25048500 2.13636100

H -6.43475800 1.27276100 3.01566000

H -5.02859400 2.57529600 1.49481800

C -5.32991900 -0.10264700 0.84869100

C -5.46656700 -1.45178600 1.23036200

C -5.12189700 0.18307900 -0.51509400

C -5.34812500 -2.48037700 0.29187100

H -5.64959400 -1.71403700 2.27816900

C -4.99626200 -0.84284200 -1.45679200

H -5.03373300 1.22105000 -0.84889100

C -5.09360300 -2.17733900 -1.04991800

H -5.44068400 -3.52213800 0.60899700

H -4.79635700 -0.60495300 -2.50317800

H -4.96249700 -2.97777900 -1.78047000

B 1.22673700 -0.24890600 -0.05839700

C 0.17230000 -0.26786400 -1.31219100

C -0.63080400 -1.39163700 -1.55756200

C -0.18082200 0.85655400 -2.07008800

C -1.68339000 -1.42136300 -2.47501700

C -1.22929700 0.87685700 -2.99606700

C -1.98741200 -0.27191400 -3.20442000

C 2.32166900 0.96182300 -0.01566400

C 3.03513300 1.46362600 -1.10988500

C 2.61849400 1.56996300 1.20673200

C 3.95918600 2.50781700 -1.00956200

C 3.52410600 2.62226200 1.35701500

C 4.20578500 3.09271700 0.23448300

C 1.97807900 -1.67659500 0.23230500

C 1.72070900 -2.43526000 1.37466100

C 2.94355000 -2.21340300 -0.62354500

C 2.36723500 -3.63622600 1.67430500

C 3.61898400 -3.41133200 -0.37208000

C 3.32770500 -4.12876300 0.78973300

F 2.00804300 1.16163500 2.34711800

F 3.73659100 3.18028800 2.55431200

F 5.07800400 4.09497000 0.34764200

F 4.60172100 2.95941800 -2.08799600

F 2.82431700 0.97540700 -2.33718100

F 0.78177200 -2.04070300 2.27038600

F 2.07192100 -4.31763100 2.78545300

F 3.95835600 -5.27347400 1.05110800

F 4.53253600 -3.87636700 -1.22555400

F 3.26003800 -1.57156100 -1.75611300

F 0.43262800 2.03645800 -1.88858000

F -1.54992600 2.01021300 -3.63174100

F -3.01791900 -0.26395700 -4.05750400

F -2.42873300 -2.52054800 -2.63178200

F -0.46055500 -2.51834900 -0.83905400

H 0.50107300 -0.04955500 0.89993300

H -3.45210500 1.78248000 3.65218300

H -0.00604400 2.50513500 2.79794600

H 0.29974400 4.60320200 1.56506900

C -0.94403000 0.19943400 3.41682600

H 0.12068900 0.00149100 3.20077500

H -0.99677100 1.07702200 4.07216600

H -1.33889700 -0.68435100 3.93331100

**TS3d (conformation 2)**

**Ggas = -3134.252863 a.u.**

**Egas = -3134.64268301 a.u.**

C 1.63830500 -0.32851200 2.15037200

C 1.53033400 -1.61081000 1.48003000

C 0.67826600 -2.62694400 1.98165600

C 2.24227200 -1.86634500 0.28422300

C 0.53893600 -3.83200200 1.30350500

C 2.13990900 -3.09369400 -0.36108100

H 2.88714900 -1.09993400 -0.13567100

C 1.27654100 -4.07475200 0.13696800

H 2.70366800 -3.27430300 -1.27799400

H 1.15739600 -5.01899200 -0.39940700

C 2.06096700 0.90615000 1.43965300

H 2.63492000 0.74588000 0.52558300

H 1.12433400 1.40810500 1.14545800

H 2.59899200 1.58863400 2.10859200

N 3.79696400 -0.72984500 3.19368300

H 3.81084500 0.06657200 3.83207100

Si 5.29959500 -0.95372400 2.23946000

H 6.48188900 -0.83761100 3.14297900

H 5.21024000 -2.32087700 1.66610700

C 5.33255500 0.33595900 0.88514100

C 5.41731200 1.70728500 1.19560800

C 5.11756900 -0.02833400 -0.45872600

C 5.24760100 2.67923200 0.20605600

H 5.60306300 2.03065700 2.22559600

C 4.93159500 0.93946200 -1.45015100

H 5.07372900 -1.08472500 -0.73848000

C 4.98649100 2.29562900 -1.11406600

H 5.30602000 3.73915200 0.46609400

H 4.71720300 0.63327500 -2.47557400

H 4.82677200 3.05510000 -1.88319100

B -1.17865400 0.13670100 -0.01551200

C -0.45359100 -0.65080600 -1.25042200

C 0.69743700 -0.14314000 -1.86677600

C -0.80641400 -1.93697100 -1.67291600

C 1.42900200 -0.81950500 -2.84366800

C -0.10258700 -2.65642200 -2.64305400

C 1.02688400 -2.09405000 -3.23632700

C -2.65491800 -0.41987900 0.40425100

C -3.75242300 -0.32359800 -0.45484300

C -2.92488800 -0.98319600 1.64952500

C -5.03499300 -0.76096000 -0.11965100

C -4.18986900 -1.43449500 2.03684600

C -5.25426100 -1.32086400 1.14215300

C -1.26802000 1.76221200 -0.18022200

C -1.23574300 2.57278100 0.95771700

C -1.42207100 2.44825700 -1.38914000

C -1.34526900 3.96492900 0.92841800

C -1.53608100 3.83896500 -1.47161300

C -1.50119200 4.60311500 -0.30273900

F -1.94229800 -1.13402000 2.57287700

F -4.38775200 -1.97152900 3.24489500

F -6.47013300 -1.74469100 1.48727600

F -6.04815600 -0.65305200 -0.98044400

F -3.58446400 0.19789400 -1.67934000

F -1.07152700 2.01693500 2.18224200

F -1.29463200 4.68454900 2.05331600

F -1.60593500 5.93063800 -0.36331100

F -1.67442400 4.44626000 -2.65113900

F -1.44433900 1.77960300 -2.54949400

F -1.83495400 -2.59019700 -1.10851000

F -0.45855600 -3.90580400 -2.95485000

F 1.75182200 -2.79299800 -4.11280100

F 2.55310400 -0.28972800 -3.34854600

F 1.19946400 1.05242700 -1.49391700

H -0.45826500 -0.07010500 0.94353900

H 3.52962900 -1.55109400 3.73828300

H 0.06263300 -2.44674100 2.86061700

H -0.15958600 -4.58407400 1.67560500

C 0.95568500 -0.10491900 3.45782600

H -0.11191100 0.05643800 3.22525400

H 1.02385300 -0.96125800 4.13975000

H 1.32059500 0.80420300 3.95254000

**TS4d (conformation 1)**

**Ggas = -3134.272734 a.u.**

**Egas = -3134.66264595 a.u.**

H 0.17691600 -0.75307400 0.93256600

B 0.13236800 0.84873700 -0.59681800

C -0.69974600 -0.06939100 -1.58512400

C -2.00070400 0.21987500 -2.02218100

C -0.17557500 -1.29229300 -2.03257000

C -2.71954300 -0.63200100 -2.86402700

C -0.88023000 -2.19188400 -2.82574300

C -2.17047600 -1.85524000 -3.24232200

C 1.67989400 1.11692600 -0.80955900

C 2.50037400 1.46311100 0.27555200

C 2.31348600 1.03695600 -2.05785600

C 3.86505500 1.70693300 0.14534300

C 3.67957300 1.27076700 -2.22595300

C 4.45836800 1.59796100 -1.11327100

C -0.64030000 1.89826600 0.32573400

C -1.07497300 1.74438700 1.64015900

C -0.93270300 3.12601000 -0.27465000

C -1.80787300 2.72593600 2.31115400

C -1.65207100 4.13791800 0.35942500

C -2.10011200 3.92926500 1.66746200

F 1.05677800 -1.66135600 -1.65928800

F -0.35008100 -3.36513200 -3.16089600

F -2.88258800 -2.70619300 -3.96939700

F -3.95737300 -0.32204800 -3.24157800

F -2.62630000 1.33715200 -1.64829600

F 1.61327000 0.73690800 -3.15434800

F 4.24413200 1.17808600 -3.42619000

F 5.76560000 1.78145400 -1.24465100

F 4.61549600 1.98084000 1.20943000

F 1.99132400 1.50570300 1.51070400

F -0.80602100 0.62771300 2.33432500

F -0.53698800 3.33332000 -1.54008000

F -1.92030400 5.28170400 -0.26266000

F -2.79619800 4.87084400 2.29319000

F -2.23945100 2.50804300 3.55282800

H 0.73025000 -0.53281200 0.43706400

N -0.02171900 -2.20744000 2.54849900

H 0.41792800 -1.44359100 3.05218900

Si -1.72159700 -2.40477200 2.93857500

H -1.97157400 -1.60740800 4.17203400

H -2.01154600 -3.85001500 3.17157900

C 0.95154200 -3.21654000 2.07098000

C -2.91558400 -1.77835500 1.61929900

C -3.52842000 -0.51749400 1.75936300

C -3.24430600 -2.54855500 0.48670600

C -4.42673200 -0.03847000 0.80137300

H -3.29708600 0.10580600 2.62724000

C -4.15026500 -2.07792200 -0.46812400

H -2.79241900 -3.53508900 0.34442200

C -4.74404100 -0.82063600 -0.31207400

H -4.87989200 0.94866300 0.92375700

H -4.38700300 -2.68559500 -1.34578200

H -5.43483400 -0.44569100 -1.06979600

C 2.26242800 -2.49580600 1.68940400

C 2.89898900 -2.67863200 0.45331700

C 2.87516100 -1.63186500 2.61595400

C 4.09475400 -2.01782300 0.14985900

C 4.06216000 -0.96235900 2.31583600

H 2.42151600 -1.47040200 3.59795500

C 4.68170800 -1.15598600 1.07699100

H 4.50046900 -0.27828700 3.04572700

H 5.60379700 -0.62271400 0.83406800

C 1.27403000 -4.24403900 3.18376500

H 2.02829900 -4.97086700 2.84307800

H 1.66742700 -3.73858200 4.07831700

H 0.36502400 -4.79463900 3.47300700

H 2.45924100 -3.32382500 -0.30514000

H 4.56030400 -2.17287400 -0.82691300

C 0.33137500 -3.95925700 0.87627400

H -0.61519200 -4.43172900 1.17889700

H 0.12855700 -3.27878100 0.03850800

H 0.98924000 -4.76520200 0.52096200

**TS4d (conformation 2)**

**Ggas = -3134.273363 a.u.**

**Egas = -3134.66080584 a.u.**

H -0.09825700 0.99793400 0.08527400

B -0.28695000 -1.39546200 -0.29248800

C 1.24524700 -1.37821800 -0.70937100

C 2.21348000 -2.21507700 -0.13645300

C 1.72830400 -0.47697000 -1.66716100

C 3.56756000 -2.15290700 -0.47432100

C 3.06799700 -0.37573900 -2.02647800

C 3.99567300 -1.22674600 -1.42442500

C -1.40648800 -1.26600100 -1.41408100

C -2.65634100 -0.70604700 -1.11946200

C -1.22389100 -1.66642700 -2.74360200

C -3.65285100 -0.50135400 -2.07017600

C -2.20203000 -1.49639100 -3.72707800

C -3.42231600 -0.90778500 -3.38793700

C -0.69864800 -1.96814500 1.13465600

C -0.03200300 -1.57433400 2.30213000

C -1.74572100 -2.88116700 1.31643100

C -0.37936800 -2.02059500 3.57451500

C -2.11937700 -3.36590800 2.57288800

C -1.43244100 -2.93064600 3.70806500

F 0.88422300 0.37955300 -2.27116000

F 3.47055800 0.53652800 -2.90838600

F 5.28176900 -1.13760700 -1.73831700

F 4.45258300 -2.95240400 0.11513000

F 1.87213900 -3.13197600 0.77518800

F -0.08030200 -2.24223500 -3.12924600

F -1.98368500 -1.89301700 -4.97757500

F -4.35907200 -0.73593800 -4.31116200

F -4.80972400 0.06411500 -1.73670600

F -2.92699600 -0.31030100 0.13520200

F 0.98845200 -0.70659900 2.22221300

F -2.43564200 -3.34193500 0.26751000

F -3.11662900 -4.23718100 2.69571300

F -1.77681800 -3.37832500 4.90836800

F 0.27927000 -1.59857300 4.65094000

H -0.47175300 0.24884600 0.28220600

N 0.63344100 2.58548100 -0.31076300

H 1.20956800 2.17194300 -1.04214700

Si -0.70861900 3.49119400 -1.05074500

H -1.23051100 2.54876700 -2.07808100

H -0.24946800 4.75088600 -1.70899400

C 1.47935400 3.03427700 0.84349100

C -2.00701000 3.93708100 0.22272400

C -2.85114800 2.95065900 0.76941700

C -2.14194200 5.26075800 0.68275700

C -3.78805300 3.27516500 1.75358400

H -2.78348600 1.91583000 0.42955200

C -3.07759600 5.58831500 1.66912500

H -1.50888100 6.05066700 0.26670900

C -3.89969100 4.59392600 2.20795600

H -4.43465300 2.49578900 2.16499000

H -3.16660800 6.62105200 2.01618000

H -4.63188200 4.84741300 2.97912200

C 2.82705800 2.29538200 0.73349000

C 3.25165000 1.34390800 1.67253100

C 3.67856100 2.56979400 -0.35377300

C 4.48599500 0.69860600 1.53541100

C 4.91223600 1.93167800 -0.49084700

H 3.37612800 3.29183800 -1.11626700

C 5.32527500 0.99362800 0.46001700

H 5.54504000 2.15513400 -1.35264900

H 6.28225300 0.47981600 0.34789400

C 1.73027800 4.55926600 0.79914600

H 2.41121400 4.85535600 1.61130100

H 2.17546900 4.87787800 -0.15394900

H 0.78644100 5.11014500 0.93586300

H 2.61958700 1.08013800 2.51810500

H 4.78602800 -0.04802800 2.27513100

C 0.73249300 2.71219600 2.14894200

H -0.22910000 3.24324500 2.16982000

H 0.52852800 1.63895100 2.24465900

H 1.31909900 3.03658000 3.02142600

**TS5d (conformation 1)**

**Ggas = -3655.735880 a.u.**

**Egas = -3656.21902862 a.u.**

Si 0.79821900 -0.41187900 -0.85628900

H -0.71892700 -0.31453900 -0.49559700

C 1.54628300 -1.24397700 0.62864000

C 2.41076300 -0.51917200 1.47056600

C 1.18308700 -2.55338000 0.99490100

C 2.90591000 -1.09046600 2.64427800

H 2.67743000 0.50932900 1.22334900

C 1.66642700 -3.11802900 2.17730100

H 0.51442600 -3.13709500 0.36064200

C 2.52855400 -2.38874400 3.00296900

H 3.56576500 -0.51023100 3.29346900

H 1.36865600 -4.13194900 2.45590800

H 2.89808900 -2.82920700 3.93254100

B -2.01274000 0.11534700 0.13487000

C -1.97650900 1.68214300 -0.20785000

C -1.54014400 2.13409500 -1.45584900

C -2.31494600 2.67699500 0.71405200

C -1.36779300 3.48231200 -1.76587300

C -2.17716000 4.03805700 0.43916600

C -1.69426600 4.44096000 -0.80716200

C -2.97873100 -0.78150200 -0.77592400

C -4.19066700 -0.29440500 -1.27681400

C -2.66067000 -2.09573200 -1.13141500

C -5.03588400 -1.05793800 -2.08827200

C -3.46728400 -2.89052300 -1.94259500

C -4.66861200 -2.36207300 -2.42489500

C -1.68041800 -0.34546400 1.63138700

C -2.27911600 -1.43849400 2.27199200

C -0.65124800 0.27720400 2.35374600

C -1.87219800 -1.90056800 3.52748400

C -0.21832300 -0.14637200 3.60646800

C -0.82952300 -1.25555800 4.19357900

H 1.14688800 1.00700400 -1.00069800

H 0.76076100 -1.19171700 -2.11286900

F -2.45392400 4.95465500 1.36387100

F -1.48813900 5.73086500 -1.05394900

F -0.85115200 3.85667300 -2.93622100

F -1.20340900 1.24399700 -2.40789500

F -2.76477500 2.34518600 1.92905900

F 0.01320700 1.31129100 1.81068500

F 0.78461000 0.46993500 4.22765500

F -0.41647600 -1.69546300 5.37626200

F -2.46496000 -2.95297500 4.08725800

F -3.28518000 -2.10657300 1.69672900

F -1.51978900 -2.64667800 -0.67715000

F -3.11211800 -4.13425800 -2.25686400

F -5.45830900 -3.10046800 -3.19574600

F -6.18298100 -0.55560800 -2.53717800

F -4.60157200 0.94278400 -0.97401800

N 3.71350200 0.27669700 -2.14491800

H 3.13694600 -0.03813000 -2.92153100

Si 3.82983700 2.03882400 -2.12657500

H 2.92848500 2.49620600 -3.22226600

H 5.23219000 2.48187500 -2.38896400

C 4.79578100 -0.70140000 -1.86416600

C 3.21414400 2.84903100 -0.53738100

C 2.00854700 3.57931700 -0.55040300

C 3.89777800 2.73457400 0.68918600

C 1.49585500 4.15196800 0.61835900

H 1.45352200 3.70067900 -1.48568100

C 3.37996200 3.28991900 1.86275200

H 4.84675300 2.19517500 0.74108100

C 2.17244000 3.99436500 1.83033400

H 0.56663200 4.72353100 0.58435900

H 3.92066700 3.17394300 2.80575900

H 1.76215300 4.42511900 2.74695700

C 4.16181700 -2.09396500 -1.66454100

C 4.38449500 -2.87818000 -0.52372200

C 3.33691300 -2.62903100 -2.67079800

C 3.80129900 -4.14214800 -0.38950800

C 2.74279500 -3.88521900 -2.53775900

H 3.14754200 -2.06045300 -3.58574200

C 2.97463200 -4.65165800 -1.39149500

H 2.09972500 -4.26787600 -3.33443400

H 2.51238000 -5.63577300 -1.28203500

C 5.78545800 -0.77685600 -3.05187300

H 6.56872300 -1.52856300 -2.86608400

H 5.26621300 -1.05502500 -3.98164600

H 6.26927000 0.19998700 -3.21031300

H 5.00209000 -2.50740400 0.29313700

H 3.98417300 -4.72171300 0.51830400

C 5.56205300 -0.23891600 -0.61803100

H 6.00306000 0.75459000 -0.79048600

H 4.90353400 -0.18614800 0.25912100

H 6.39493800 -0.91974900 -0.39360500

**TS6d (conformation 1)**

**Ggas = -3655.710725 a.u.**

**Egas = -3656.18905358 a.u.**

Si 5.14723700 -0.67089300 -1.18619900

H 4.49418300 -0.88249700 -2.50217000

C 5.15214000 -2.22714500 -0.15422200

C 4.62904700 -3.43622000 -0.65057700

C 5.62578800 -2.19705300 1.17270300

C 4.56705400 -4.57499500 0.15704700

H 4.24666200 -3.48957200 -1.67410800

C 5.56162000 -3.33314800 1.98355400

C 5.02812000 -4.52261400 1.47616800

H 4.14761200 -5.50246900 -0.23994800

H 5.92737800 -3.29155300 3.01247500

H 4.96923500 -5.40990000 2.11114200

C 2.04955800 0.24889300 -0.18917100

C 1.53430700 1.23707700 -1.15215800

C 1.78033600 1.09855400 -2.53632500

C 0.68936400 2.27926800 -0.71821600

C 1.21875300 1.98014800 -3.45214200

C 0.09949000 3.14022700 -1.64381300

H 0.43574100 2.38618200 0.33417800

C 0.36505900 2.99846400 -3.00797300

H -0.59097700 3.91103800 -1.29542600

H -0.11328200 3.66212300 -3.73205400

N 4.20252800 0.58892400 -0.28143500

H 4.31915300 0.39014700 0.71702400

H 6.51470000 -0.11944500 -1.40033800

Si 4.62673200 2.34080700 -0.54930100

H 6.11574200 2.40547100 -0.55395600

H 4.08940200 2.72620400 -1.87460500

C 3.91821700 3.35519300 0.84714800

C 4.38974900 3.16577500 2.16224200

C 2.87557500 4.27627500 0.63563700

C 3.81750700 3.85204700 3.23543600

H 5.21275400 2.47012500 2.36022600

C 2.30450100 4.96821300 1.70774600

H 2.49029400 4.44358900 -0.37252200

C 2.76843800 4.75029100 3.00837800

H 4.18785000 3.68750600 4.25027800

H 1.48839000 5.67239300 1.52849900

H 2.31365600 5.28241500 3.84745800

B -1.79334100 -0.29959700 0.11629600

C -2.19812900 0.35865200 -1.32559200

C -3.10829000 1.40110900 -1.52559200

C -1.49524200 -0.02040000 -2.47292300

C -3.30250000 2.03080700 -2.76158600

C -1.65830200 0.56676700 -3.72465300

C -2.56713500 1.61482800 -3.87007700

C -1.73411600 -1.93675900 0.11728500

C -2.51161700 -2.77452100 -0.68726800

C -0.82933400 -2.59620900 0.95296600

C -2.39213200 -4.16784200 -0.68408400

C -0.66476200 -3.98253900 0.98604800

C -1.46172600 -4.77692700 0.16099400

C -2.65168800 0.23923800 1.39434300

C -2.14648600 1.17304800 2.29707400

C -3.94384900 -0.21436600 1.67227900

C -2.85260100 1.63716600 3.41017900

C -4.69283100 0.21657400 2.76902700

C -4.13844300 1.15119900 3.64770600

F -0.01875300 -1.88614100 1.77331200

F 0.24658200 -4.54928400 1.78568300

F -1.32823200 -6.10444300 0.17220600

F -3.15287700 -4.92323200 -1.47840700

F -3.41150000 -2.25557600 -1.53208400

F -0.90617400 1.69849700 2.13065500

F -2.31326000 2.53435200 4.24267600

F -4.83361800 1.57732000 4.70303500

F -5.92511000 -0.24579500 2.98774500

F -4.52901900 -1.09409100 0.84586500

F -0.55359500 -0.98918800 -2.39682500

F -0.90677400 0.18695600 -4.76559400

F -2.70312100 2.23154500 -5.04717400

F -4.16890300 3.03984500 -2.88384500

F -3.84378200 1.88148900 -0.50992800

H -0.64564400 0.06568600 0.28592100

H 6.05489700 -1.27853300 1.58885800

H 1.40916400 1.85127800 -4.51875600

H 2.42438000 0.29703800 -2.89833200

C 1.90470800 0.50591800 1.27574500

H 0.83879700 0.34166500 1.50733800

H 2.47213400 -0.22508000 1.86803400

H 2.16276200 1.52828400 1.56991500

C 1.95259300 -1.18564400 -0.58378300

H 0.86695600 -1.38527100 -0.59059600

H 2.29493400 -1.39496600 -1.60376700

H 2.42335100 -1.86147000 0.13762800

**TS10d (conformation 1)**

**Ggas = -2611.652804 a.u.**

**Egas = -2611.93923338 a.u.**

C -1.61105600 -1.16806500 -2.67450200

N -0.49087500 -0.48731600 -1.98704500

H 0.36439500 -1.01996800 -2.14199900

H -0.31086400 0.41654100 -2.42526500

C 1.91938900 -0.93216500 0.34167500

C 1.64782200 -2.24587600 -0.07437300

C 3.27938500 -0.57411200 0.34378300

C 2.63299100 -3.13148700 -0.50859800

C 4.29827900 -1.44010700 -0.05785500

C 3.97041400 -2.72636200 -0.49267500

C -0.49778300 -0.32351200 1.52341300

C -1.68977200 0.42390600 1.45652800

C -0.57171800 -1.49501700 2.29998000

C -2.89375600 -0.00314500 2.01167200

C -1.75182200 -1.94406900 2.89247700

C -2.92279300 -1.19657500 2.73814500

C 1.05386900 1.62370500 0.40051100

C 0.82942400 2.63474500 1.34532300

C 1.57037000 2.04550300 -0.83126100

C 1.07417800 3.98355800 1.08606400

C 1.81138500 3.38380500 -1.13873800

C 1.56616700 4.35858900 -0.16714100

F 2.31154100 -4.35164000 -0.92817100

F 0.39430700 -2.69941600 -0.10279900

F 4.92190000 -3.55868400 -0.88710800

F 5.56939500 -1.05392200 -0.02928300

F 3.66268400 0.63810300 0.75487400

F 1.84800300 1.14847200 -1.79106400

F 2.28078200 3.73667800 -2.33174200

F 1.79915200 5.63549600 -0.43434300

F 0.84923700 4.90857900 2.01318200

F 0.36804000 2.32319700 2.55999100

F -1.72603400 1.60118700 0.82782800

F -3.99878500 0.72157800 1.87603700

F -4.05157700 -1.61062800 3.29160300

F -1.77085600 -3.06191400 3.61142600

F 0.51744300 -2.23586500 2.51615200

B 0.78944600 0.10114000 0.73200000

C -2.92307900 -0.40737700 -2.43081600

C -4.14246700 -0.91922600 -2.91006800

C -2.95345600 0.79948900 -1.72248400

C -5.34683200 -0.25470900 -2.67173700

C -4.15884000 1.46345700 -1.46908700

H -2.02928700 1.21990800 -1.33786600

C -5.36228500 0.93947700 -1.94231500

H -4.14851800 2.38798700 -0.88668900

H -6.30638100 1.45314100 -1.74425100

C -1.33516400 -1.24764800 -4.19441900

H -0.40135900 -1.80299000 -4.38406700

H -1.23226800 -0.23745900 -4.62239800

H -2.14735000 -1.75844000 -4.73367100

H -4.16055200 -1.85544500 -3.47290200

H -6.28100700 -0.67659400 -3.05186200

C -1.72435200 -2.58829500 -2.09249400

H -2.52152900 -3.15656900 -2.59173600

H -1.94352200 -2.54655400 -1.01705300

H -0.78096000 -3.13995800 -2.22621600

**TS10d (conformation 2)**

**Ggas = -2611.653891 a.u.**

**Egas = -2611.93926446 a.u.**

C 3.57514100 1.04610300 2.30102200

N 2.10778200 1.16037700 2.14835600

H 1.71602900 1.69080900 2.92819000

C 0.13408400 0.64165700 -0.79992600

C 0.54782100 1.89767300 -0.32531700

C 1.01382200 0.00673800 -1.69924300

C 1.72905300 2.50726800 -0.73484000

C 2.19299800 0.59580700 -2.14508700

C 2.55644000 1.84927700 -1.64327500

C -2.48280400 0.83887300 -0.04573600

C -3.40714500 0.47172500 0.94797700

C -2.78797000 2.00851500 -0.76415500

C -4.55345400 1.21459700 1.22910300

C -3.93263500 2.76747200 -0.52303500

C -4.81743800 2.36686900 0.48289300

C -1.27533900 -1.58538300 -0.18676100

C -2.34846800 -2.34530600 -0.67086600

C -0.24304300 -2.30217000 0.42838300

C -2.38453600 -3.73783400 -0.58218700

C -0.23979400 -3.69008000 0.54240500

C -1.32204700 -4.41196500 0.02927500

F 2.10671100 3.67740700 -0.22603800

F -0.19484300 2.55204100 0.56805700

F 3.69301600 2.41242200 -2.02758500

F 2.97644400 -0.01909400 -3.02533300

F 0.71013300 -1.19355500 -2.20478900

F 0.79808800 -1.64551500 0.95709600

F 0.77305700 -4.32328400 1.12886000

F -1.34267500 -5.73325300 0.12325000

F -3.41062800 -4.42663400 -1.06999400

F -3.37893900 -1.74086600 -1.26959600

F -3.19724400 -0.61519300 1.69599700

F -5.39143300 0.84106800 2.19036000

F -5.90499900 3.08002300 0.72961700

F -4.19117200 3.85885100 -1.23499200

F -1.98473800 2.42755300 -1.74310400

B -1.20434900 -0.02036800 -0.34326500

C 4.08033600 0.15443400 1.15189200

C 4.92559800 0.62038800 0.13431900

C 3.65092700 -1.18164000 1.07893300

C 5.30749700 -0.20960600 -0.92588800

C 4.01145500 -2.00920800 0.01444500

H 3.00050100 -1.59150500 1.85242100

C 4.84518600 -1.52468500 -0.99756500

H 3.62990000 -3.03314700 -0.02105900

H 5.12420400 -2.16329400 -1.83894800

C 4.13634100 2.47359500 2.23264800

H 3.73025200 3.06830400 3.06696100

H 3.84224200 2.97258100 1.30020800

H 5.23274700 2.48323000 2.32213700

H 5.28265700 1.64968400 0.14023800

H 5.95080600 0.18867600 -1.71413400

H 1.66553300 0.24184600 2.16846500

C 3.97528800 0.42121700 3.65811700

H 5.06932100 0.33145900 3.75273400

H 3.60924500 1.05018600 4.48671200

H 3.54296200 -0.58335000 3.78248500
